# Halogenated N-(1,3,4-oxadiazol-2-yl) benzamides are effective eradicators of methicillin-resistant *Staphylococcus aureus* biofilms

**DOI:** 10.1016/j.bmc.2025.118437

**Published:** 2025-10-09

**Authors:** George A. Naclerio, Christopher S. Vennard, Kenneth I. Onyedibe, Dielson da S. Vieira, Nader S. Abutaleb, Marxa L. Figueiredo, Mohamed N. Seleem, Herman O. Sintim

**Affiliations:** a Chemistry Department, Institute for Drug Discovery, Purdue University, West Lafayette, IN 47907, United States; b Purdue Institute of Inflammation, Immunology, and Infectious Disease, West Lafayette, IN 47907, United States; c Department of Biomedical Sciences and Pathobiology, Virginia-Maryland College of Veterinary Medicine, Virginia Polytechnic Institute and State University, Blacksburg, VA 24060, United States; d Basic Medical Sciences, Purdue University, College of Veterinary Medicine, West Lafayette, IN 47907, United States; e Center for Emerging, Zoonotic and Arthropod-borne Pathogens, Virginia Polytechnic Institute and State University, Blacksburg, VA 24061, United States; f Department of Chemistry and Biochemistry, University of Notre Dame, Notre Dame, IN 46556, United States

**Keywords:** *N*-(1,3,4-oxadiazol-2-yl) benzamides, Biofilm, MRSA, Drug resistance, Membrane depolarizers

## Abstract

Due to the ever-increasing threat of methicillin-resistant *Staphylococcus aureus* (MRSA), we have embarked on a campaign to discover novel antibacterial agents which are effective at eradicating both MRSA, as well as acting on pre-formed MRSA biofilms. Using the known scaffold of *N*-(1,3,4-oxadiazol-2-yl)benzamides, we performed a halogenation study which has led to the identification of **HSGN-2241**. This novel compound was found to effectively kill many multi-drug resistant Gram-positive clinical isolates in a bactericidal manner. The growth inhibition mechanism of **HSGN-2241** was found to be via potassium ion release and subsequent depolarization of the cell membrane. As an initial safety test, **HSGN-2241** was found to not lyse human red blood cells, so it is a promising lead compound for the treatment of bacterial infections caused by *Staphylococcus aureus*.

## 1 Introduction

Antimicrobial resistance (AMR) has continued to climb at an alarming rate in recent years, despite the increased awareness and research into the factors contributing to AMR.^1^ The WHO has routinely published a list of priority pathogens for which new treatments are urgently needed. While the worst pathogens with the lowest survival rates and few treatments are generally Gram-negative bacteria, there is one Gram-positive species that stands out as particularly concerning to the WHO. *Staphylococcus aureus* (*S. aureus*) has taken the lead as the only bacteria associated with over 1 million deaths in 2019 and is consistently in the WHO priority pathogens list, with methicillin-resistant *S. aureus* (MRSA) achieving the “HIGH” category in 2024, surpassed only by 4 organisms.^2,3^ Recent antibiotic developments such as Vancomycin, Daptomycin, and Linezolid have proven to be effective against MRSA, although their efficacy is waning quickly as new resistant strains are discovered.^4^ Therefore, novel antibiotics with distinct mechanisms of action from current drugs are urgently needed to combat MRSA infections.

One mechanism that MRSA can employ to persist or resist antibiotics is the formation of biofilms. Most drugs which are efficacious against planktonic MRSA are unable to penetrate the biofilm matrix, which results in recurring or persistent infections. Biofilms show approximately 10–10,000 times more antibiotic resistance than planktonic bacteria and cause about 80 % of chronic and recurrent bacterial infections.^5,6^ Bacteria in biofilms can be eradicated in one of two ways: (1) direct killing of bacteria without biofilm disruption, or (2) disruption of biofilm by chemical agents or abrasive force, then bacterial killing. While many other groups have employed various strategies to combat biofilms,^7–9^ our group as well as others have shown that oxadiazoles are a privileged scaffold which can be elaborated to afford specific activities.^10–18^ Our group has synthesized and tested many *N*-(1,3,4-oxadiazol-2-yl) benzamide derivatives in the past, we had yet to explore how various terminal halogenation patterns would affect antibacterial activity or killing mechanisms.

**Fig. 1.**
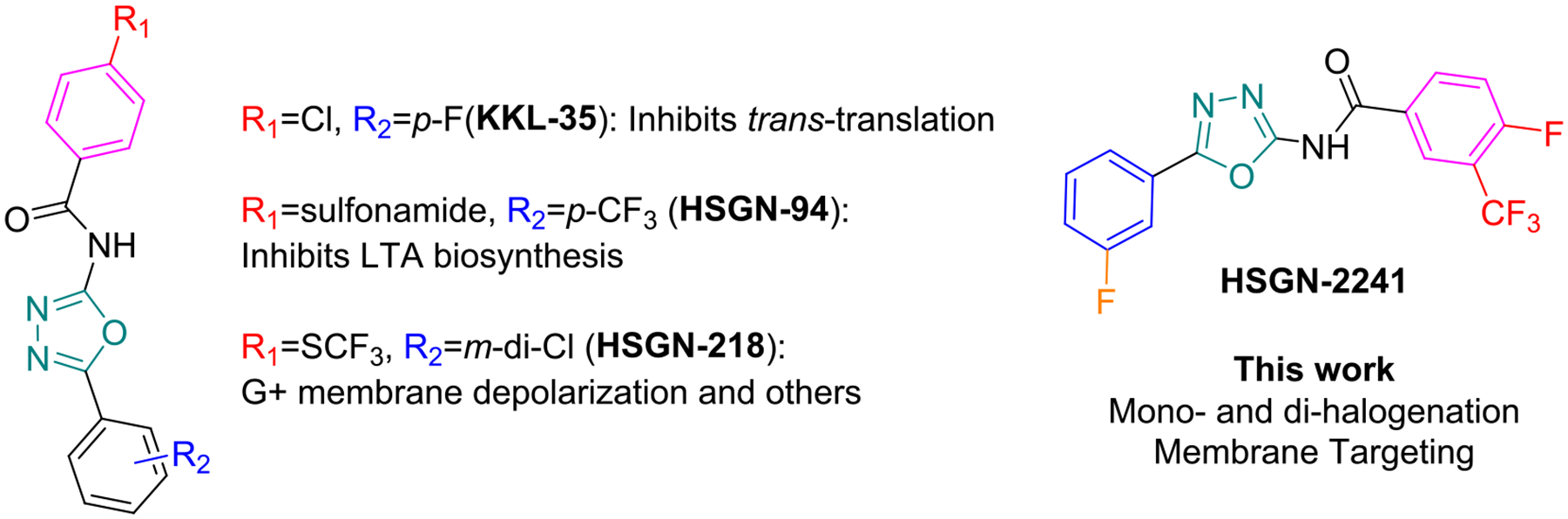
Previously reported *N*-(1,3,4-oxadiazol-2-yl) benzamides which have antibacterial activity. This study has focused on halogenation to increase potency.

After the survey of various halogenation patterns, we identified **HSGN-2241** (Fig. 1) as a potent antibacterial agent which was effective at inhibiting the growth of a panel of multidrug-resistant Gram-positive clinical isolates. Killing kinetics, biofilm eradication ability, resistance generation, and hemolysis were also investigated. Moreover, we performed several mechanistic studies for **HSGN-2241** against *S. aureus*, which has indicated that this compound acts by depolarizing *S. aureus* membranes.

## 2 Results and discussion

### 2.1. Synthesis of N-(1,3,4-oxadiazol-2-yl)benzamides and initial antibacterial screening

**Scheme 1.**
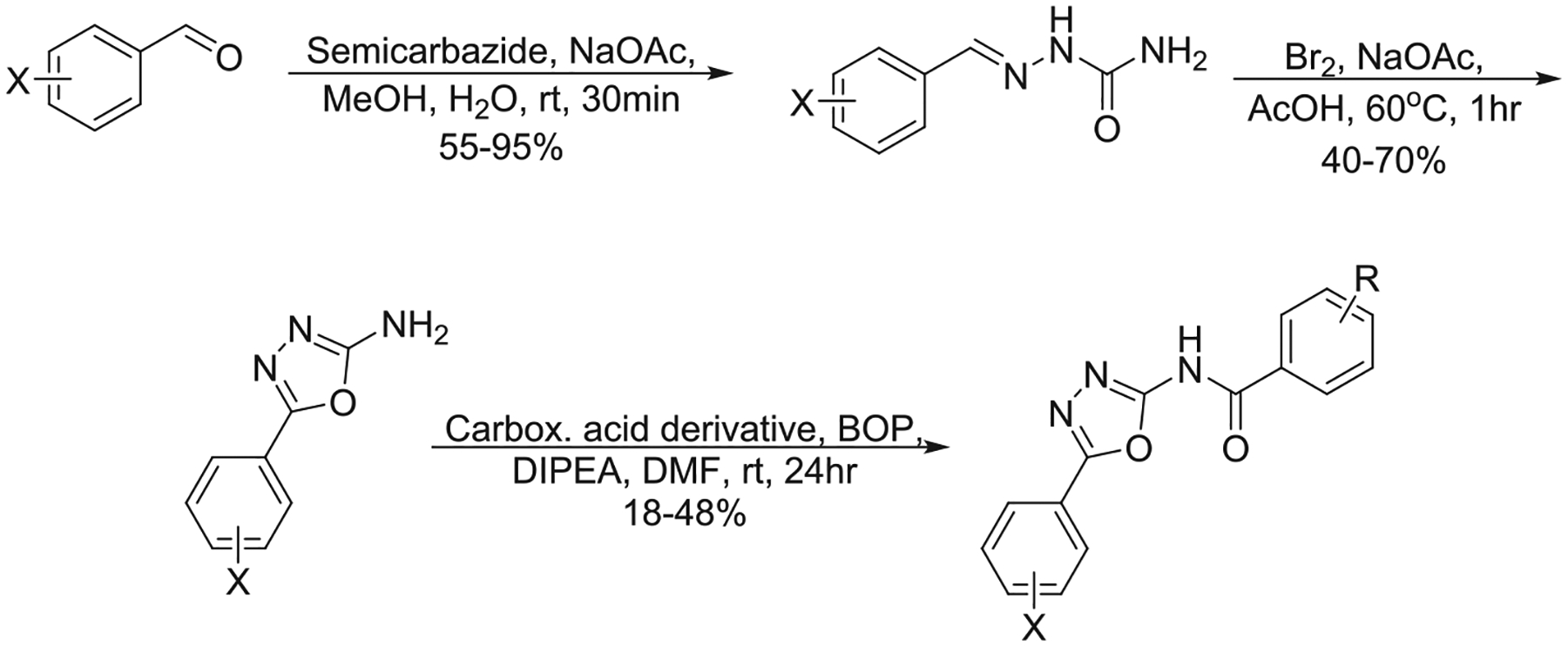
Synthetic scheme to access N-(1,3,4-oxadiazol-2-yl)benzamides. DIPEA = diisopropylethylamine, DMF = *N*, *N*-dimethylformamide, BOP = (Benzotriazol-1-yloxytris(dimethylamino)phosphonium hexafluorophosphate).

*N*-(1,3,4-oxadiazol-2-yl)benzamides were synthesized by first combining halogenated benzaldehydes and semicarbazides to afford the semicarbazone, then cyclized to form the amino-oxadiazole. Then, final products were obtained by using standard amide coupling procedures with halogenated benzoic acids (Scheme 1).

While the non-halogenated derivative (**1,** Table 1) had weak activity against both *S. aureus* and MRSA, *para*-substitution with halogens yielded increased inhibitory activity across the board. The most prominent increase in activity was the chloro-derivative **(3)** in which the MIC decreased 32-fold. In a similar fashion, the *meta*-substituted derivatives also showed increased activity across the board, with the chloro-derivative (**8**) once again becoming 32-fold more potent. Pleasingly, the combination of both *meta-* and *para*- halogenation compounded the increased potency, yielding the two lead compounds in this study **HSGN-2241** (**16**) and **HSGN-2263** (**17**) which were 64-fold and 128-fold more potent than the non-halogenated counterpart, respectively. Further substitutions which included the *ortho*- position decreased activity for all derivatives as compared to their counterparts which indicates *o*-halogenation is not tolerated regardless of halogen size or electronics.

### 2.2. HSGN-2241 and -2263 were effective at inhibiting growth of drug-resistant Gram-positive clinical isolates

While both lead compounds were active against the reference strains of *S. aureus* and MRSA, there can sometimes be a disparity between effectiveness against clinical strains of bacteria. Therefore, we moved forward to test both **HSGN-2241** and **-2263** against several MRSA isolates from the ARLG as well as other Gram-positive bacteria including vancomycin-resistant *enterococci* (VRE) and *Listeria monocytogenes*. Both lead compounds were able to inhibit the growth of all isolates tested and even outperformed vancomycin and linezolid in many cases (See Tables 2 and 3).

### 2.3. Halogenation pattern of HSGN-2241 and HSGN-2263 exhibits a difference in growth inhibition mechanism

**Fig. 2.**
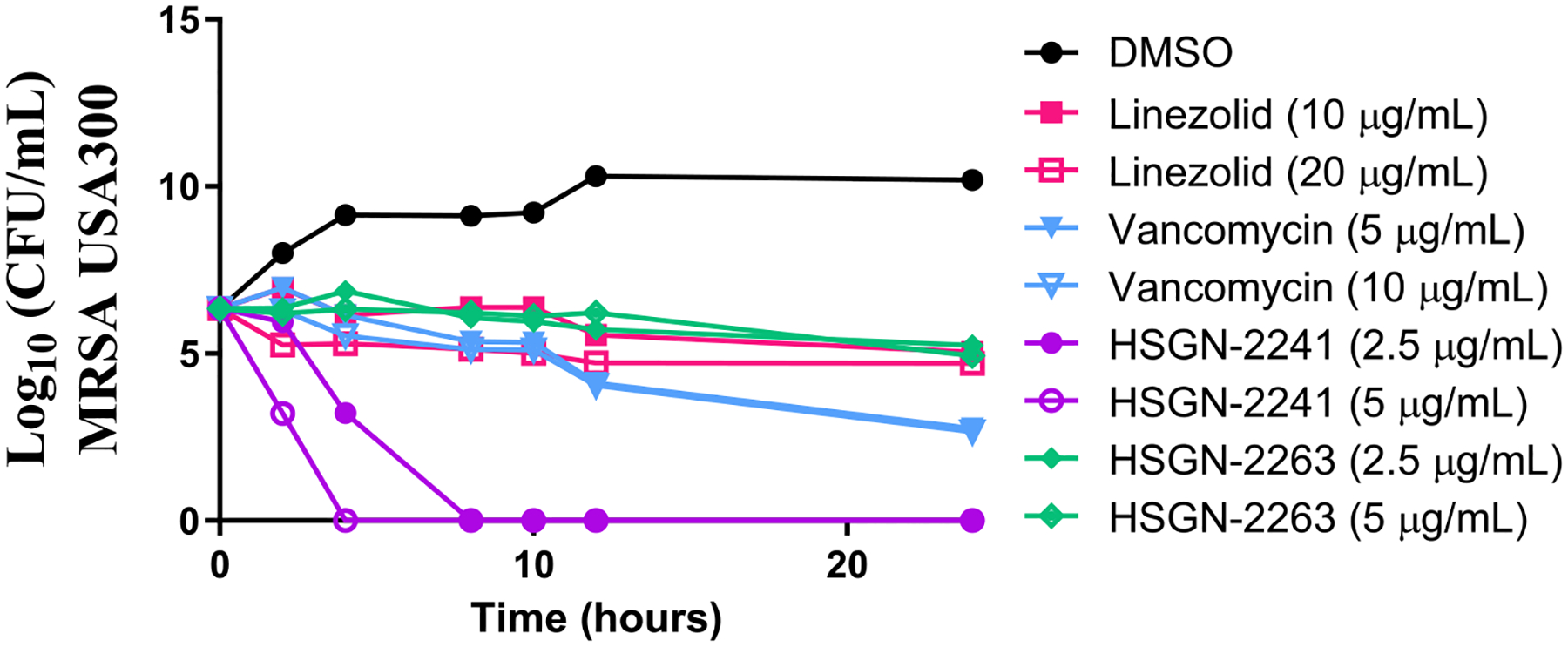
Time-kill kinetics assay using MRSA USA300. All compounds were tested at 5× MIC and 10× MIC.

To identify the mode of inhibition of **HSGN-2241** and **-2263,** we performed a time-kill kinetics assay (Fig. 2). Interestingly, the two lead compounds did not share the same growth inhibition mechanism. **HSGN-2263** was found to be bacteriostatic and **HSGN-2241** was found to be bactericidal. **HSGN-2263** only showed a modest 1.1- and 1.4-log_10_-reduction in bacterial count over 24 h, which was comparable to linezolid (a known bacteriostatic agent). In contrast, **HSGN-2241** at 10× and 5× MIC, was bactericidal with the bacterial load dropping below the detectable limit after just 4 h and 8 h respectively (Fig. 2).

### 2.4. Elaboration of HSGN-2241 via heterocycle scaffold-hopping

Because of the rapid bactericidal activity which was found for **HSGN-2241** we decided to further elaborate the molecule by replacing the 3-fluorophenyl substituent with various commercially available heterocycles. It has been a known medicinal chemistry principle for some time that replacement of unadorned phenyl rings with heterocycles increases water solubility while also giving the opportunity for the molecule to form additional interactions with the target protein. The synthetic route to access these derivatives was the same as stated above. Out of all the additional derivatives that were synthesized, none were more potent than **HSGN-2241**.

### 2.5. Potassium ion release and membrane depolarization due to HSGN-2241

**Fig. 3.**
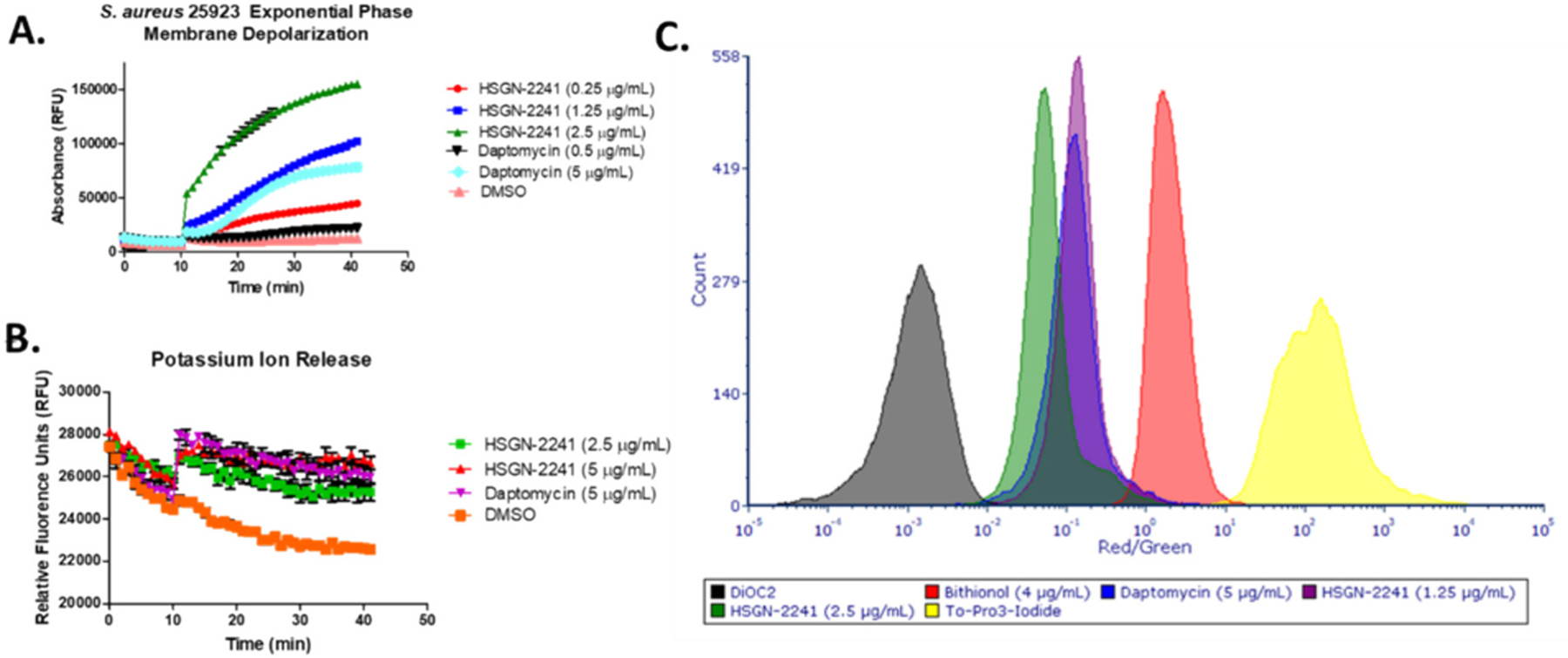
**(A)** Membrane depolarization assay using DiSC3(5) as the fluorescent probe. **B)** Potassium ion release assay using PBFI as the fluorescent probe. (**C**) Flow cytometry showing **HSGN-2241** shows a close red:green profile to a known membrane depolarizer (daptomycin). Charts were constructed using FCS Express software, version 7.0.

The bacterial membrane is considered an attractive target for antibacterial drugs due to its necessity and accessibility. In order to determine the mechanism of bacterial killing, we first measured membrane depolarization and permeabilization. DiSC3(5) is a fluorescent dye which can be used to quickly visualize membrane depolarization and Sytox green is a dye which was used to determine membrane permeability. We discovered that in all cases of **HSGN-2241** treatment, *S. aureus* membranes showed depolarization (Fig. 3A), but not increased permeability (Fig. S1). As a secondary method to confirm membrane depolarization, we opted to use flow cytometry (Fig. 3C). By using both permeable (To-PRO 3 iodide, red) and impermeable (DiOC_2_(3), green) dyes, we can assess the ratio of red:green to determine the relative level of depolarization. We opted to use both depolarization (daptomycin)^19^ and permeabilization (bithionol)^20^ controls to better match the profile of **HSGN-2241** to a control compound. It was confirmed that **HSGN-2241** showed depolarization activity and matched a red:green ratio of daptomycin which is a known membrane depolarizer.

Because of the observed depolarization, we investigated potassium ion release as a possible depolarization mechanism using the K^+^-sensitive fluorescent probe PBFI. Potassium ion release was observed at both 10× and 20× MIC (Fig. 3B).

### 2.6. HSGN-2241 alters S. aureus membrane morphology

**Fig. 4.**
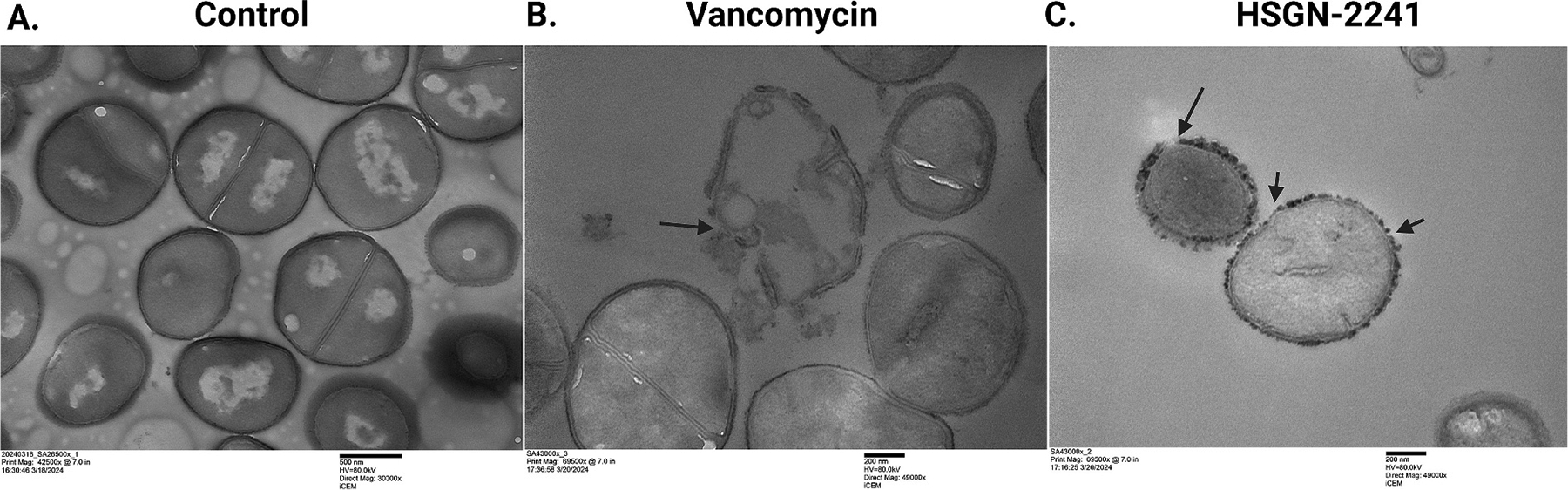
Transmission electron microscopy of MRSA USA300 with **A)** no treatment (26,500× magnification), **B)** Vancomycin (43,000× magnification), or **C)** HSGN-2241 (43,000× magnification). Arrows direct towards areas of membrane disruption and damage.

To directly visualize the effects that **HSGN-2241** has on bacterial membranes, we conducted transmission electron microscopy (TEM) of MRSA USA300 in the presence or absence of treatments. MRSA USA300 was grown for 4 h to show daughter cell formation in the control (Fig. 4A). In the Vancomycin treatment group, notable cell lysis occurred as indicated by the arrow (Fig. 4B) as well as loss of the separation between outer membrane and plasma membrane and alteration in the septum division and thickness of septa. In the **HSGN-2241** treatment group, the cells were not lysed, although the cell wall itself can be seen as porous and irregular in addition to aberrant division septa as compared to the untreated group, structures without specific form can be visualized at the cytoplasmatic area, and no division point is observed (Fig. 4C).

### 2.7. HSGN-2241 showed synergistic interaction with commercially available antibiotics daptomycin, vancomycin, and linezolid

As the threat of antimicrobial resistance increases, it has become more common to treat infections with a combination of agents. It is possible that by using multiple medications in a single treatment regimen, less of each drug is needed, antibacterial resistance generation is lowered, off-target effects can be reduced, and drugs which have shown high resistance can be reintroduced to the clinic.^21,22^ Studies have consistently highlighted that prolonged monotherapy with Vancomycin and Linezolid inevitably leads to decreased susceptibility or even the emergence of resistance in *S. aureus*. Synergy is typically sought after by combining multiple drugs that target different parts of the same biological pathway, although this is not always the case.^23^ Our findings reveal that **HSGN-2241** exhibits synergy with three antibiotics with distinct mechanisms of action (daptomycin, vancomycin and linezolid), which increases the importance of these findings (Table 4). We hypothesize that the synergistic effect of **HSGN-2241** with several drugs is due to membrane depolarization by **HSGN-2241** which causes increased uptake of additional agents, and thus a lower concentration of each agent is required for the same effect.

### 2.8. HSGN-2241 both prevents and disrupts MRSA biofilms

**Fig. 5.**
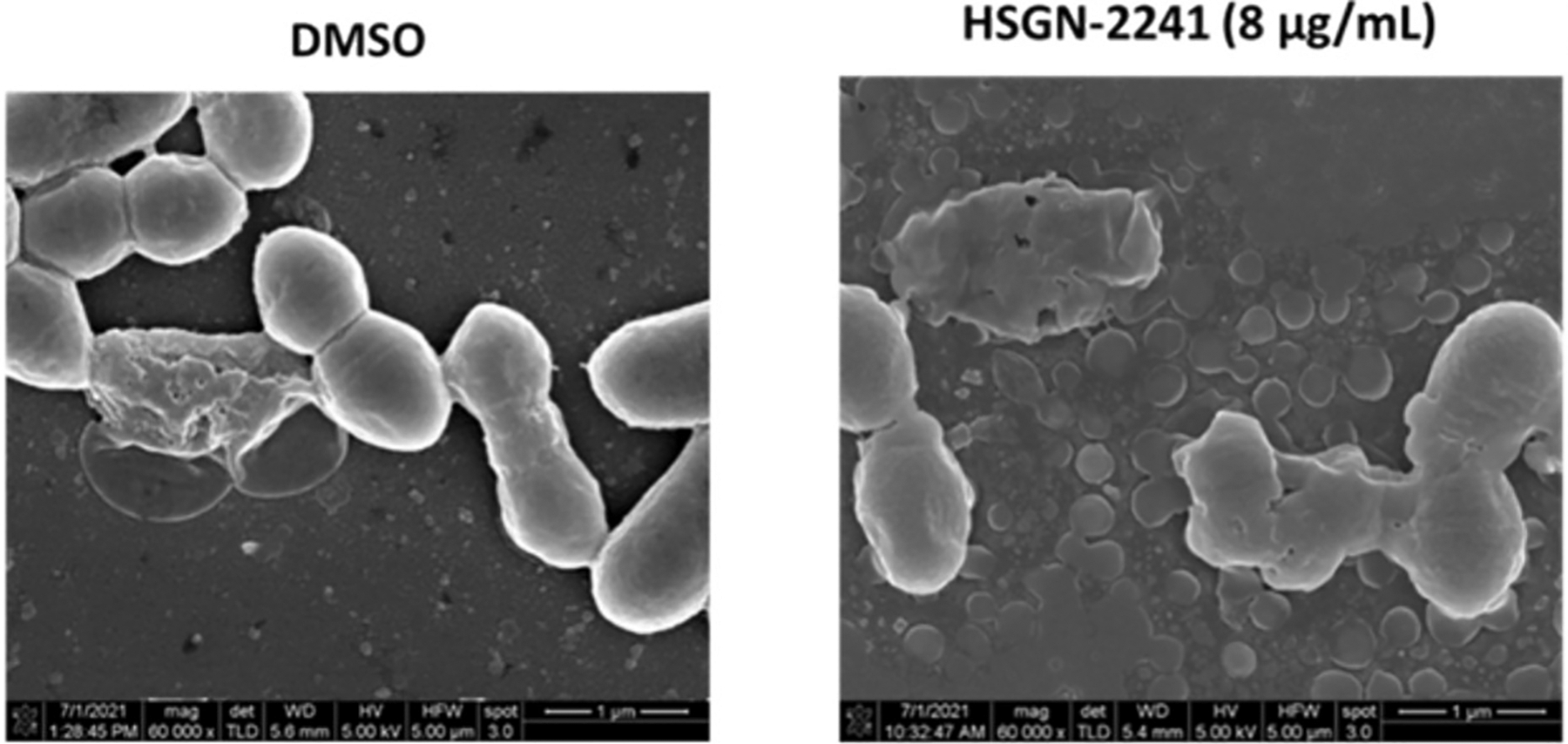
**A)** SEM images of MRSA USA300 biofilms treated with either DMSO or **HSGN-2241**. Images were captured at 60,000× magnification. Images were captured using the FEI Nova Nano-SEM instrument.

To directly visualize the effects of **HSGN-2241** on mature biofilms, we used SEM to image the biofilms. The images show that **HSGN-2241** could lyse bacteria in the biofilm whereas the untreated control did not show profound bacterial lysis (Fig. 5).

**Fig. 6.**
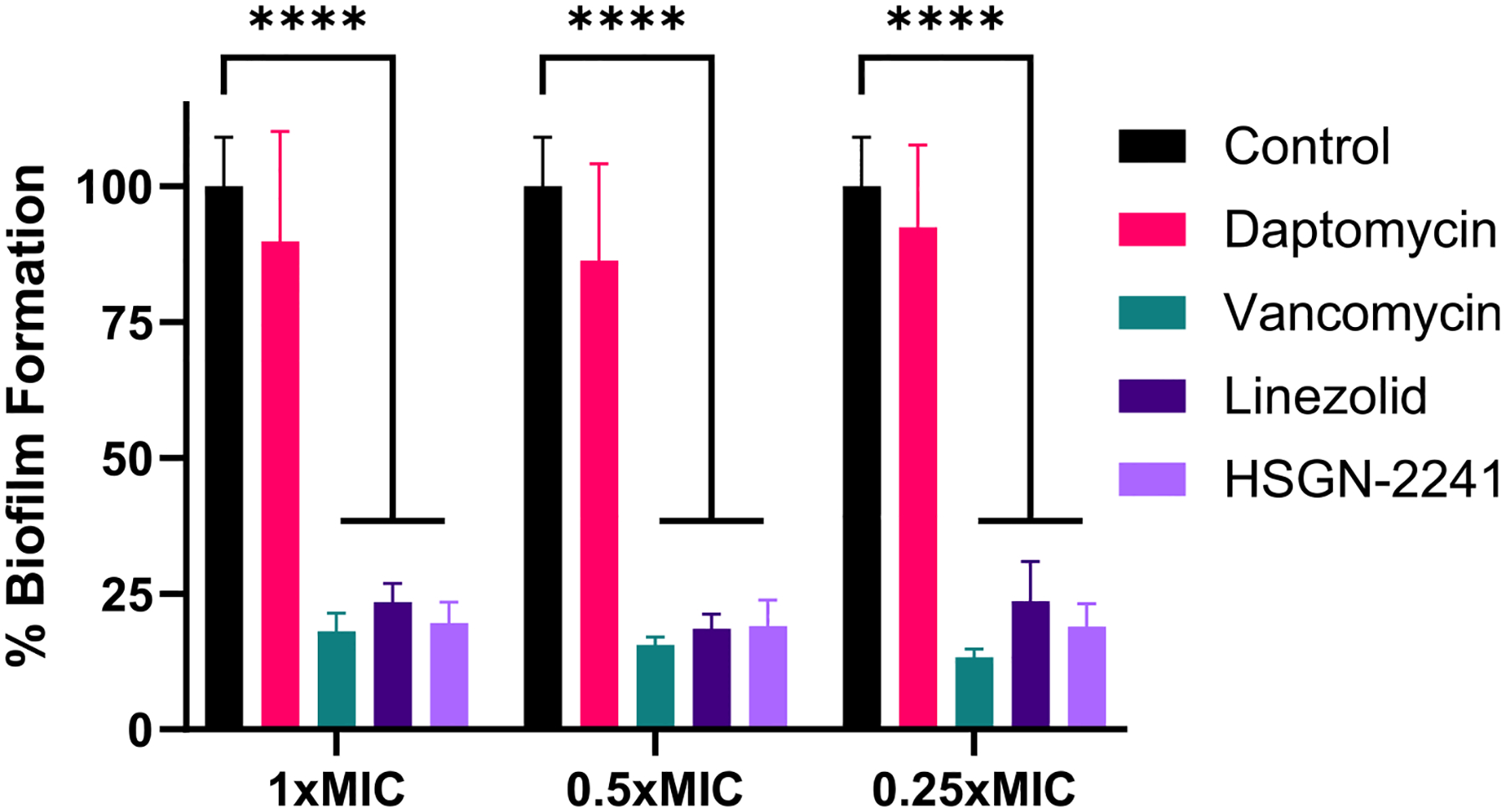
Representation of how much biofilm the bacteria was able to produce when treated with 1 × MIC, 1/2× MIC and 1/4× MIC. Two-way ANOVA was performed to compare the concentrations and the groups, and **** denotes *p* < 0.0001.

In addition to the disruption of pre-formed biofilms, **HSGN-2241** was able to prevent biofilm formation. When lower concentrations of the compounds were used (1 × MIC, 1/2 × MIC and 1/4 × MIC), they could prevent biofilm formation in 24 h incubation. Daptomycin was unable to prevent biofilm formation under the conditions tested, although **HSGN-2241**, Vancomycin, and Linezolid were able to prevent biofilm (Fig. 6).

### 2.9. MRSA does not develop resistance to HSGN-2241

**Fig. 7.**
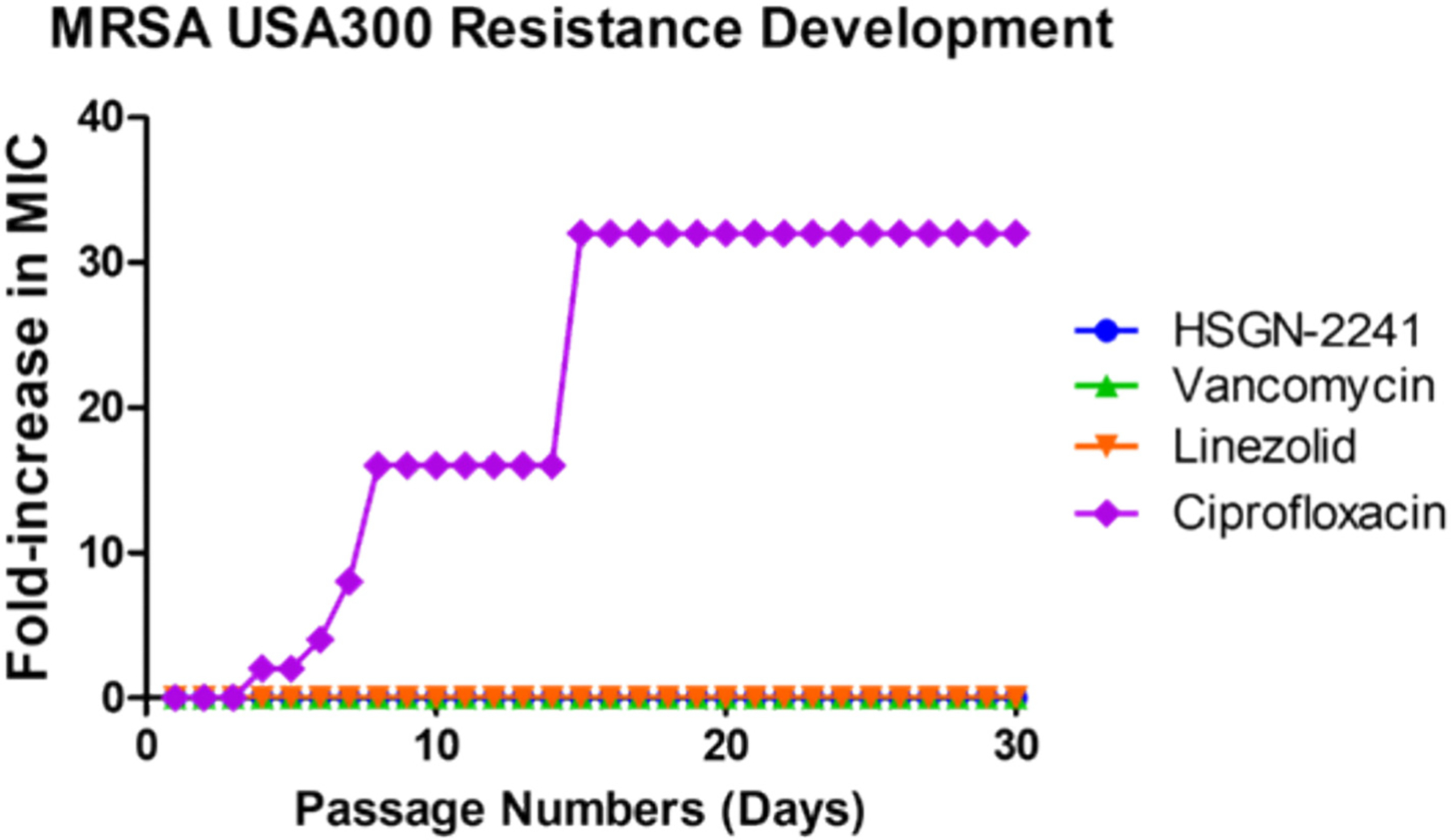
Serial passage assay using MRSA USA300. The cutoff for determining point of resistance was set to be a four-fold increase in the initial MIC.

Because rapid resistance generation remains a clinical challenge for novel chemical entities, we carried out a serial passage assay to determine if resistance to **HSGN-2241** could be seen over 30 days. Pleasingly, we did not observe any resistance to **HSGN-2241** over the time period tested (Fig. 7).

### 2.10. HSGN-2241 does not lyse human red blood cells

**Fig. 8.**
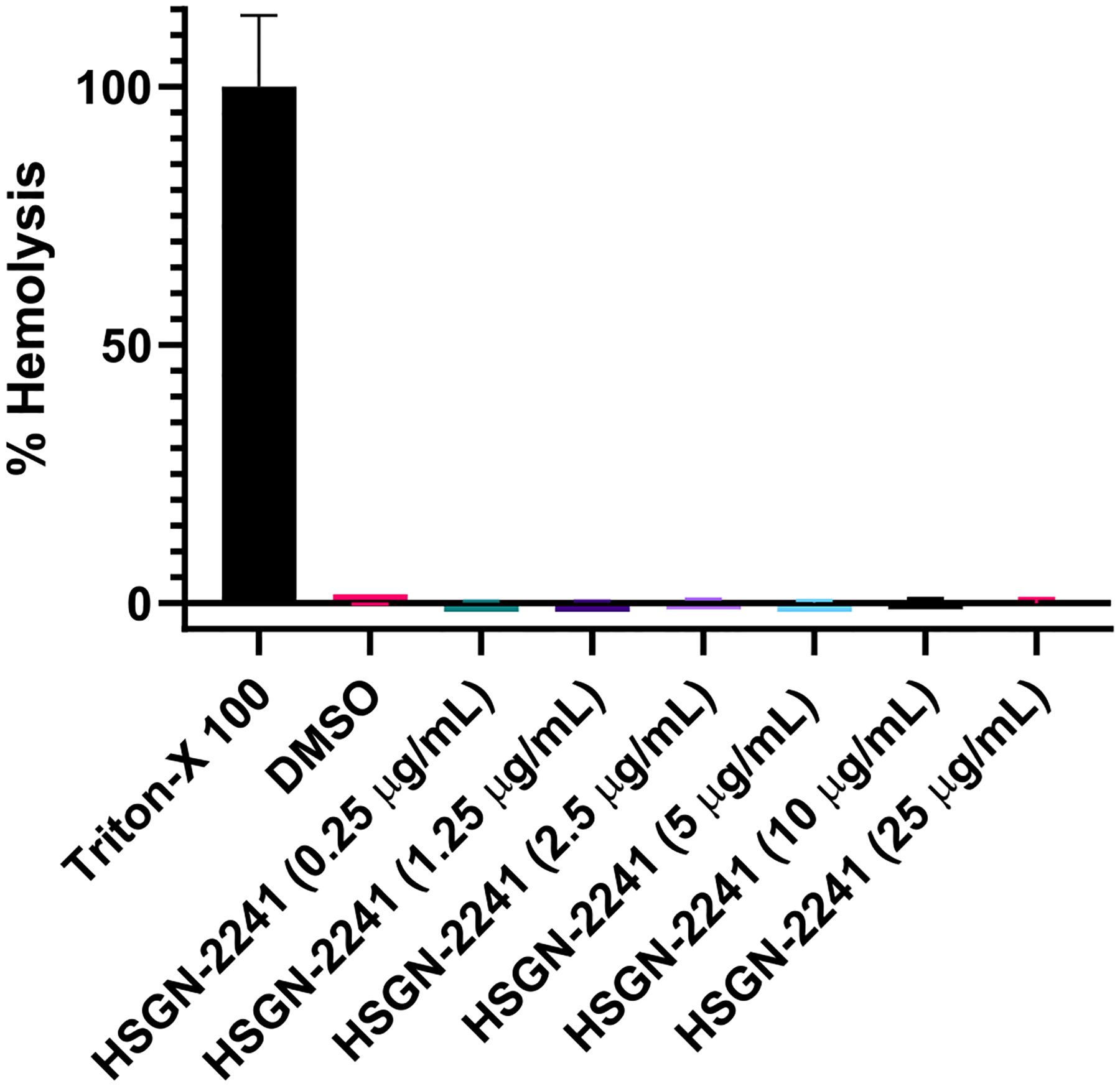
Hemolytic activity of **HSGN-2241** (in triplicate) against human RBCs. % Hemolysis was determined by normalizing samples to Triton-X100 (100 %) and DMSO (0 %). Error bars represent standard deviation of the individual normalized values.

Human RBCs were found to be hemolytically stable when exposed to HSGN-2241 at concentrations 25× to 100× MIC (Fig. 8).

## 3 Conclusion

Our exploration of the *N*-(1,3,4-oxadiazol-2-yl)benzamide scaffold with halogen substitution has led us to a new lead compound **HSGN-2241**. This compound was effective at killing MRSA clinical isolates with concentrations as low as 0.125 μg/mL. In addition, **HSGN-2241** was able to prevent and eradicate *S. aureus* biofilms, which remain a significant clinical challenge. A time-kill kinetics study has shown that this compound acts in a bactericidal manner, with further testing indicating that it acts by depolarizing the membrane via potassium ion release. A preliminary safety assessment has shown that **HSGN-2241** also did not lyse human RBCs even at a concentration 100 times higher than the MIC against *S. aureus*. Ultimately, we have used traditional medicinal chemistry principles on a known scaffold to improve the potency of a *N*-(1,3,4-oxadiazol-2-yl)benzamide derivative with the simple addition of several halogenated substituents. We hope this study will encourage others to realize the promise of both systematic halogenation of their own scaffolds, as well as the potential of the *N*-(1,3,4-oxadiazol-2-yl) benzamide scaffold as a powerful tool for antibiotic discovery.

## 4 Materials and methods

### 4.1. Bacterial strains, media, and reagents

Sources for each of the bacterial stains are listed in Tables S1 and S2. All media, broths, and agars were purchased from commercial sources and used without further alteration. The purchased biochemical reagents have sources listed in Table S4. Synthesized final compounds were prepared in stock solutions using DMSO as the solvent.

### 4.2. Determination of MICs

The MICs were determined using the broth microdilution method following the guidelines of the Clinical and Laboratory Standards Institute (CLSI).^24^ A bacterial suspension equivalent to a 0.5 McFarland standard was further concentration of approximately 5 × 10^5^ CFU/mL. *Listeria monocytogenes* was diluted in TSB as described. MICs reported are the minimum concentration of each compound in which bacterial growth could not be seen visually.

### 4.3. Checkerboard assay

Drug interactions (HSGN-2241 with Vancomycin, Linezolid, and Daptomycin) were evaluated using the Checkerboard assay described by Bellio et al. (2021).^25^ Drug A (HSGN-2241) was prepared at 2× MIC and 200 μL of this solution was added to wells A1–11. Then, drug A was diluted from top to bottom, from row B2—H. After that, 1 × MIC of the compounds (Vancomycin, Linezolid, and Daptomycin) were added to the first column from A1-H8 and serially diluted across the plate. Bacteria were then added into each well at a starting concentration of 5 × 10^5^ CFU/mL. After incubating at 37 °C for 24 h, the Fractional Inhibitory Concentration (FIC) Index (FICI) was calculated.

### 4.4. Time-kill analysis

A bacterial culture (~ 2.35 × 10^6^ CFU/mL) was treated with **HSGN-2241**, **−2263**, linezolid, or vancomycin at 5× and 10× MIC and incubated at 37 °C as previously described.^13,26^ An aliquot from each treatment was collected after the corresponding times, serially diluted in PBS, and plated onto TSA plates. Plates were incubated at 37 °C for 18–20 h before enumerating colonies to determine the CFU/mL counts.

### 4.5. Hemolysis assay

The hemolytic activity of **HSGN-2241** against human red blood cells (RBCs) was assayed following a procedure described previously.^13^

### 4.6. Biofilm formation

Biofilms were developed according to the modified microtiter plate test proposed by Stepanović et al.^27^ For USA300 (ATCC BAA-1756^™^) the quantification of biofilm was performed, where in each 200 μL TSB with 0.5 % of glucose was added 5 × 10^5^ CFU/mL of bacterial suspension. Negative control wells contained just media without adding any bacterial cells. The plates were incubated for 24 h at 37 °C.

### 4.7. Interference with biofilm formation

The antibiofilm activity of HSGN-2241, Vancomycin, Linezolid, and Daptomycin during biofilm formation was carried out according to adaptations of the protocol used by Stepanović et al. (2000). Initially, 200 μL TSB (plus 0.5 % of glucose) with the compounds were added in each well in three dilutions (1 × MIC, 1/2 × MIC and 1/4 × MIC). After that 5 × 10^5^ UFC/mL of bacterial suspension was added and the plates incubated for 24 h at 37 °C. After this period, the washing, fixing (200 μL, 95 % formalin), staining, destaining (200 μL, 33 % acetic acid solution) and reading (590 nm) steps took place. The assay was performed in technical and biological triplicate on independent days. Two-way ANOVA was performed for the analysis, and for multiple comparison Tukey test, significance was considered when *p* < 0.05.

### 4.8. Scanning electron microscopy for biofilm

*S. aureus* biofilms were first grown on glass slides (13 mm) then treated with each test agent for 24 h. After treatment, the media was removed and washed twice with PBS. Fixation of samples was carried out as previously described^28^ (chilled 2.5 % glutaraldehyde in 0.1M sodium cacodylate buffer). Post-fixation processing included treatment with 1 % osmium tetroxide as well as dehydration with a series of ethanol washes. The slides were then processed using three changes of hexamethyldisilazane (HMDS) and then dried overnight. Platinum coating was then carried out using a Cressington 208HR sputter coater then imaged with FEI Nova Nano-SEM 200 equipment at 5 kV.

### 4.9. Transmission electron microscopy for bacterial ultrastructure

This method was performed at Purdue University and Indiana University School of Medicine Center for Electron Microscopy. The bacteria USA 300 (ATCC BAA-1756^™^) was used as a MRSA strain. 1 mL of 10^6^ CFU/mL was incubated (37 °C, 150 rpm) with 4× the MIC of the compounds (HSGN-2241 and Vancomycin) for 4 h. A control without treatment was used for comparison. After 4 h, the tubes containing 1 mL of the bacterial cells treated were centrifuged at 5000 x*g* (rcf) for 5 min at 4 °C. The supernatant was carefully removed and then 500 μL of fixative added, and samples kept at 4 °C until analysis.

Centrifuged samples were immediately fixed in 2.5 % glutaraldehyde with 2 % paraformaldehyde in 0.1 M Cacodylate buffer (pH 7.4) for overnight at 4 °C. After that, the samples were washed from fixative with 0.1 M Cacodylate buffer (pH 7.4) and postfixed with 1 % osmium tetroxide and 1.5 % potassium ferrocyanide in 0.1 M Cacodylate buffer (pH 7.4) for one hour at room temperature. Samples were dehydrated using a graded ethanol series, incubated in Propylene Oxide for 30 min, and embedded in EMbed 812. All reagents for TEM processing were purchased from EMS (Hatfield, PA, USA). The resin-embedded samples were polymerized for 48 h at 60 °C. Ultrathin sections (80 nm) were cut using a Diatome 45° ultra knife (Diatome, Nidau, Switzerland) and placed on 200 mesh nickel grids (EMS) The sections were contrasted for 1 min with Uranyless (EMS), washed with water, blotted, and dried in a vacuum desiccator for 1 h before imaging. All images were captured on a Tecnai 12 transmission electron microscope operated at 80 kV with a Hamamatsu Orca HR CCD camera.

### 4.10. Multi-step resistance selection

To determine if MRSA could form resistance to **HSGN-2241**, a multi-step serial passage experiment was used, as described previously.^15,29,30^ The initial MIC was first found for each agent as described above, and for each subsequent passage, a portion of the bacterial culture which was just below the measured MIC was used for the inoculation of the next plate.^30^

### 4.11. Bacterial membrane depolarization assay

The fluorescent dye DiSC3(5) (3,3’-Dipropylthiadicarbocyanine Iodide) was used to determine *S. aureus* membrane depolarization according to a previously reported procedure.^28^

### 4.12. Potassium ion release assay

The fluorescent dye PBFI (Potassium-binding benzofuran isophthalate) was used to determine if treatment with **HSGN-2241** caused potassium ion release in *S. aureus* ATCC 25923. The assay was carried out according to a previously reported procedure.^20^

### 4.13. Bacterial membrane permeability assay

The fluorescent dye Sytox green was used to determine if treatment with **HSGN-2241** increased *S. aureus* membrane permeability. The assay was carried out according to a previously reported procedure.^19,28^

### 4.14. Flow cytometry for membrane depolarization activity

The membrane depolarization activity of **HSGN-2241** was confirmed using flow cytometry according to a previously reported procedure.^20,28^ To-PRO 3 iodide (red dye) served as the membrane-permeable dye, while DiOC2 (green dye) served as the membrane-impermeable dye. A BD Fortessa cell analyzer was used for this assay.

## Supplementary Material

1

## Figures and Tables

**Table 1:** Structures of all synthesized N-(1,3,4-oxadiazol-2-yl)benzamides used in this study and corresponding MICs against *S. aureus* and MRSA. MIC values shown are in μg/mL.

Base Structure/Series	Entry	R_1_	R_2_	R_3_	R_4_	*S. aureus* (ATCC 25923)	MRSA (ATCC 33592)
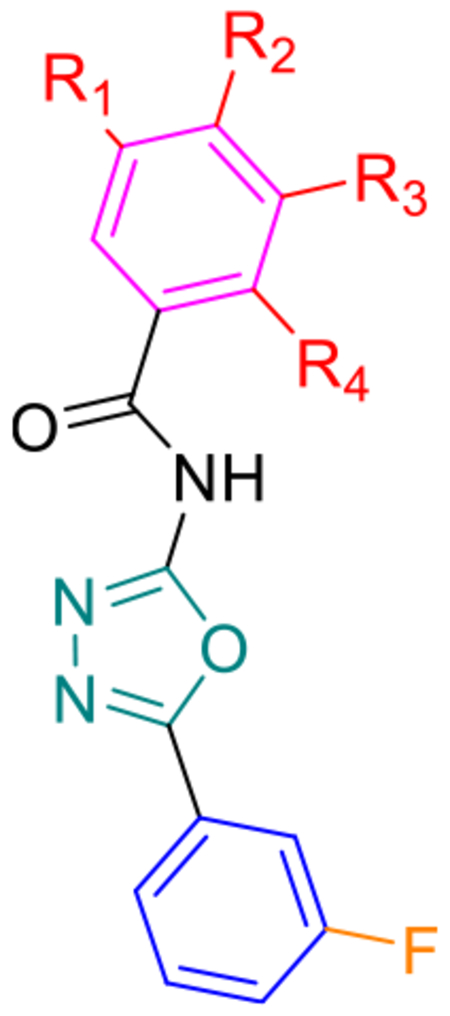	**1**	H	H	H	H	16	16
	**2**	H	F	H	H	4	4
	**3**	H	Cl	H	H	0.5	0.5
	**4**	H	Br	H	H	4	2
	**5**	H	I	H	H	2	1
	**6**	H	CF_3_	H	H	1	1
	**7**	H	H	F	H	2	2
	**8**	H	H	Cl	H	0.5	0.5
	**9**	H	H	Br	H	2	1
	**10**	H	H	CF_3_	H	1	1
	**11**	F	H	F	H	1	1
	**12**	H	F	Cl	H	0.5	0.5
	**13**	H	Cl	F	H	0.5	0.5
	**14**	H	F	Br	H	1	1
	**15**	H	Br	F	H	0.5	0.5
	**16, HSGN-2241**	H	F	CF_3_	H	0.25	0.25
	**17, HSGN-2263**	H	Cl	CF_3_	H	0.125	0.125
	**18**	F	H	CF_3_	H	0.5	0.5
	**19**	H	F	H	Cl	16	16
	**20**	H	Cl	H	F	8	8
	**21**	H	F	H	Br	32	16
	**22**	H	Br	H	F	16	16
	**23**	H	I	H	F	16	8
Linezolid	–	–	–	–		2	2
Vancomycin	**–**	–	–	–		1	1

**Table 2:** MICs of **HSGN-2241** and **-2263** against several clinically relevant Gram-positive bacteria. MIC values are displayed in μg/mL.

Bacterial Strain	HSGN-2241	HSGN-2263	Vancomycin	Linezolid
MRSA USA300	0.5	0.5	1	2
MRSA ARLG 1561	0.25	0.125	1	2
MRSA ARLG 1567	0.125	0.125	1	2
MRSA ARLG 1568	0.25	0.125	0.5	1
MRSA ARLG 1569	0.25	0.125	1	2
MRSA ARLG 1570	0.25	0.06	0.5	1
MRSA ARLG 1649	0.5	0.125	2	2
MRSA ARLG 1663	0.5	1	1	2
MRSA ARLG 1664	1	1	1	2
MRSA ARLG 1665	1	1	0.25	2
*E. faecalis* ATCC 29212	1	0.5	2	2
VRE faecalis ATCC 51575	1	0.25	>128	1
VRE faecium ATCC 700221	0.5	0.25	>128	1
*L. monocytogenes* ATCC 19115	0.25	0.125	2	2

VRE: vancomycin-resistant *Enterococcus*.

**Table 3:** Additional derivatives that were synthesized based on **HSGN-2241**. MIC values are displayed in μg/mL.

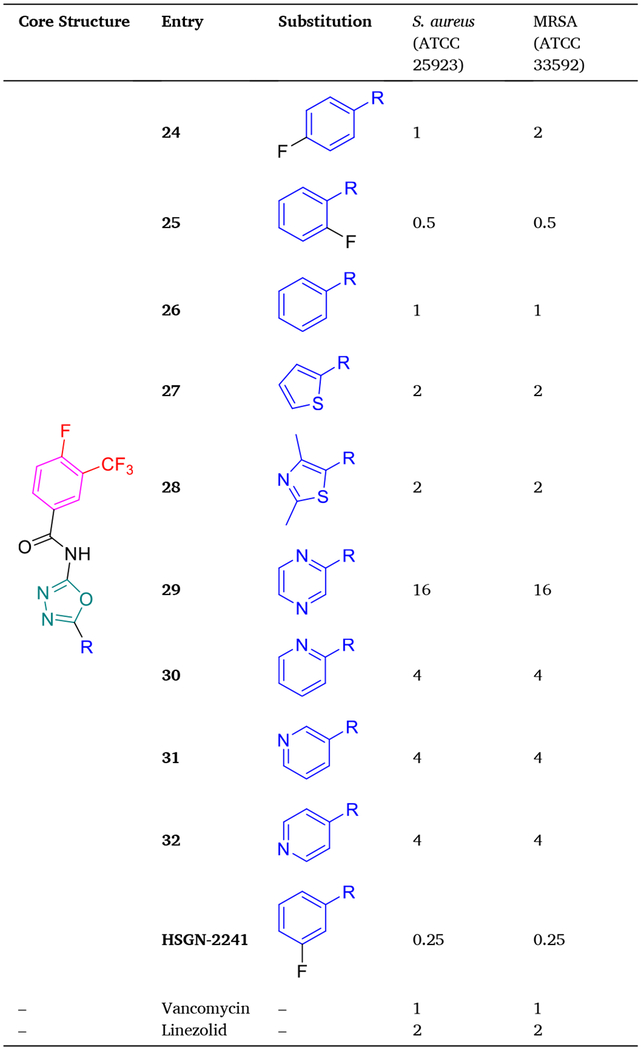

**Table 4:** Synergy of HSGN-2241 and Daptomycin, Vancomycin, or Linezolid against MRSA USA300. MIC values are displayed in μg/mL. Synergy is displayed by the letter “S”.

Drug in combination with HSGN-2241	HSGN-2241 MIC	Drug MIC	Combination MIC	FIC Index	Checkerboard Effect
Daptomycin	0.125	1	0.0157	0.141	S
Vancomycin		1	0.00393	0.0470	S
Linezolid		2	0.00785	0.0784	S

## Data Availability

Data will be made available on request.

## Appendix A. Supplementary data

Supplementary data to this article can be found online at https://doi.org/10.1016/j.bmc.2025.118437.
