# Supplementary material for: Halogenated N-(1,3,4-oxadiazol-2-yl) benzamides are effective eradicators of methicillin-resistant Staphylococcus aureus biofilms
Source: Bioorg Med Chem. Author manuscript; Available in PMC 2026 Jun 21. (PMC13283350; doi:10.1016/j.bmc.2025.118437)
Supplement: 1 [file NIHMS2184617-supplement-1.docx]

**Halogenated N-(1,3,4-oxadiazol-2-yl) benzamides are effective eradicators of methicillin-resistant Staphylococcus aureus biofilms**

George A. Naclerio^a^, Christopher S. Vennard^a^, Kenneth I. Onyedibe^a^, Dielson da S. Vieira ^a,d^, Nader S. Abutaleb^c^, Marxa L. Figueiredo^d^, Mohamed N. Seleem^c,e^, and Herman O. Sintim^a,b*^

^a^ Chemistry Department, Institute for Drug Discovery, Purdue University, West Lafayette, IN 47907, United States

^b^ Purdue Institute of Inflammation, Immunology, and Infectious Disease, West Lafayette, Indiana 47907, United States

^c^ Department of Biomedical Sciences and Pathobiology, Virginia-Maryland College of Veterinary Medicine, Virginia Polytechnic Institute and State University, Blacksburg, VA, 24060, United States

^d^ Basic Medical Sciences, Purdue University, College of Veterinary Medicine, West Lafayette, Indiana 47907, United States

^e^ Center for Emerging, Zoonotic and Arthropod-borne Pathogens, Virginia Polytechnic Institute and State University, Blacksburg, VA, 24061, USA.

Supplementary Information:

I. Chemistry:

i. Synthesis and Characterization Data of Analogs

II. Biological Analysis

i. Table S1: Bacterial strains

ii. Table S2: MRSA clinical isolates

iii. Table S3: Sequence of primers used in RT-PCR.

iv. Figure S1: Effects of **HSGN-2241** on membrane permeability in *S. aureus*

III. ^1^H, ^13^C, and ^19^F Spectra of Analogs**I. Chemistry:**

All reagents and solvents were purchased from commercial sources and utilized as collected. The ^1^H, ^13^C, and ^19^F NMR spectra were acquired in DMSO-*d*6 solvent using an 800 MHz NMR spectrometer. ^1^H NMR data are described as: chemical shift (δ ppm) (multiplicity, coupling constant (in Hz), integration). High resolution mass spectra (HRMS) were performed with electron spray ionization (ESI) technique and a TOF mass analyzer. Synthesized compounds were characterized using ^1^H NMR, ^13^C NMR, ^19^F NMR, and HRMS data.

**General Procedure for the Synthesis of Analogs 1-32.**

In a round-bottom flask was added the corresponding halogenated benzoic acid (1 eq), amine (1 eq), BOP reagent (2.7 eq) and diisopropylethylamine (22 eq) in DMF solvent (5 mL). The reaction mixture was stirred at room temperature for 24 h. After completion, the reaction mixture was concentrated under reduced pressure, diluted with ethyl acetate (20 mL), washed twice with water (10 mL), once with brine, dried over sodium sulfate, and concentrated to give a crude mixture. The crude mixture was purified by flash column chromatography (hexanes: ethyl acetate 80:20 to 70:30) to give the desired product.

***N*-(5-(3-fluorophenyl)-1,3,4-oxadiazol-2-yl)benzamide (1):**

Off-white solid (111 mg, 48%). ^1^H NMR (800 MHz, DMSO-*d*_6_) δ 8.0 (dd, *J* = 8.1, 1.4 Hz, 2H), 7.9 – 7.8 (m, 1H), 7.7 (ddd, *J* = 9.4, 2.6, 1.5 Hz, 1H), 7.7 – 7.6 (m, 2H), 7.6 (t, *J* = 7.6 Hz, 2H), 7.5 – 7.4 (m, 1H). ^13^C NMR (201 MHz, DMSO-*d*_6_) δ 165.8, 163.3 (d, *J* = 245.2 Hz), 160.5, 158.7, 133.2, 132.9, 132.2 (d, *J* = 8.0 Hz), 129.0, 128.7, 125.9 (d, *J* = 10.0 Hz), 122.7, 119.1 (d, *J* = 20.1 Hz), 113.2 (d, *J* = 24.1 Hz). ^19^F NMR (471 MHz, DMSO-*d*_6_) δ -112.5 (q, *J* = 8.7 Hz, 1F). HRMS (ESI) m/z calcd for C_15_H_11_FN_3_O_2_ [M + H]^+^  284.0835, found 284.0835.

**4-Fluoro-*N*-(5-(3-fluorophenyl)-1,3,4-oxadiazol-2-yl)benzamide (2):**

Off-white solid (51 mg, 24%). ^1^H NMR (800 MHz, DMSO-*d*_6_) δ 8.1 (dd, *J* = 8.5, 5.3 Hz, 2H), 7.9 – 7.8 (m, 1H), 7.7 (d, *J* = 9.3 Hz, 1H), 7.7 (q, *J* = 7.5 Hz, 1H), 7.5 (td, *J* = 8.7, 2.4 Hz, 1H), 7.4 (t, *J* = 8.7 Hz, 2H). ^13^C NMR (201 MHz, DMSO-*d*_6_) δ 165.9 (d, *J* = 249.2 Hz), 164.9, 163.3 (d, *J* = 245.2 Hz), 160.3, 158.8, 132.3 (d, *J* = 8.0 Hz), 131.6 (d, *J* = 8.0 Hz), 129.6, 125.9 (d, *J* = 10.0 Hz), 122.7, 119.1 (d, *J* = 22.1 Hz), 116.1 (d, *J* = 22.1 Hz), 113.2 (d, *J* = 24.1 Hz). ^19^F NMR (471 MHz, DMSO-*d*_6_) δ -107.9 (s, 1F), -112.5 (q, *J* = 8.4 Hz, 1F). HRMS (ESI) m/z calcd for C_15_H_10_F_2_N_3_O_2_ [M + H]^+^  302.0741, found 302.0739.

**4-Chloro-*N*-(5-(3-fluorophenyl)-1,3,4-oxadiazol-2-yl)benzamide (3):**

Off-white solid (46 mg, 23%). ^1^H NMR (800 MHz, DMSO-*d*_6_) δ 8.1 – 7.9 (m, 2H), 7.8 (d, *J* = 7.7 Hz, 1H), 7.7 (d, *J* = 9.2 Hz, 1H), 7.7 (d, *J* = 7.1 Hz, 1H), 7.6 – 7.6 (m, 2H), 7.5 (t, *J* = 8.8 Hz, 1H). ^13^C NMR (201 MHz, DMSO-*d*_6_) δ 165.2, 163.3 (d, *J* = 245.2 Hz), 160.2, 158.7, 138.2, 132.2 (d, *J* = 8.0 Hz), 131.8, 130.6, 129.1, 125.9 (d, *J* = 10.0 Hz), 122.7, 119.1 (d, *J* = 22.1 Hz), 113.2 (d, *J* = 24.1 Hz). ^19^F NMR (471 MHz, DMSO-*d*_6_) δ -112.5 (q, *J* = 8.5 Hz, 1F). HRMS (ESI) m/z calcd for C_15_H_10_ClFN_3_O_2_ [M + H]^+^ 318.0446, found 318.0445.

**4-Bromo-*N*-(5-(3-fluorophenyl)-1,3,4-oxadiazol-2-yl)benzamide (4):**

Off-white solid (55 mg, 31%). ^1^H NMR (800 MHz, DMSO-*d*_6_) δ 8.0 – 8.0 (m, 2H), 7.8 (d, *J* = 7.8 Hz, 1H), 7.8 – 7.7 (m, 2H), 7.7 – 7.7 (m, 1H), 7.7 (q, *J* = 7.4 Hz, 1H), 7.5 – 7.4 (m, 1H). ^13^C NMR (201 MHz, DMSO-*d*_6_) δ 165.2, 163.3 (d, *J* = 245.2 Hz), 160.2, 158.7, 132.2 (d, *J* = 8.0 Hz), 132.0, 130.8, 127.2, 125.9 (d, *J* = 10.0 Hz), 122.7, 119.1 (d, *J* = 22.1 Hz), 113.2 (d, *J* = 24.1 Hz). ^19^F NMR (471 MHz, DMSO-*d*_6_) δ -112.5 (td, *J* = 9.1, 5.8 Hz, 1F). HRMS (ESI) m/z calcd for C_15_H_10_BrFN_3_O_2_ [M + H]^+^ 361.9940, found 361.9940.

***N*-(5-(3-Fluorophenyl)-1,3,4-oxadiazol-2-yl)-4-iodobenzamide (5):**

Off-white solid (54 mg, 33%). ^1^H NMR (800 MHz, DMSO-*d*_6_) δ 8.0 – 7.9 (m, 2H), 7.8 – 7.8 (m, 3H), 7.7 (d, *J* = 9.3 Hz, 1H), 7.7 – 7.6 (m, 1H), 7.5 (t, *J* = 8.6 Hz, 1H). ^13^C NMR (201 MHz, DMSO-*d*_6_) δ 165.5, 163.3 (d, *J* = 245.2 Hz), 160.2, 158.6, 137.9, 132.4, 132.2 (d, *J* = 8.0 Hz), 130.5, 125.8 (d, *J* = 10.0 Hz), 122.7, 119.1 (d, *J* = 22.1 Hz), 113.2 (d, *J* = 24.1 Hz), 101.2. ^19^F NMR (471 MHz, DMSO-*d*_6_) δ -112.5 (q, *J* = 8.9 Hz, 1F). HRMS (ESI) m/z calcd for C_15_H_10_FIN_3_O_2_ [M + H]^+^ 409.9802, found 409.9800.

***N*-(5-(3-Fluorophenyl)-1,3,4-oxadiazol-2-yl)-4-(trifluoromethyl)benzamide (6):**

Off-white solid (53 mg, 29%). ^1^H NMR (800 MHz, DMSO-*d*_6_) δ 8.3 – 8.2 (m, 2H), 8.0 – 7.9 (m, 2H), 7.8 – 7.8 (m, 1H), 7.7 (ddd, *J* = 9.3, 2.6, 1.5 Hz, 1H), 7.7 (td, *J* = 8.0, 5.7 Hz, 1H), 7.5 (td, *J* = 8.6, 2.7 Hz, 1H). ^13^C NMR (201 MHz, DMSO-*d*_6_) δ 165.1, 163.3 (d, *J* = 245.2 Hz), 160.2, 158.6, 136.9, 132.9 (q, *J* = 32.2 Hz), 132.3 (d, *J* = 8.0 Hz), 129.6, 125.9, 125.8 (d, *J* = 10.0 Hz), 124.8 (q, *J* = 271.4 Hz), 122.8, 119.1 (d, *J* = 22.1 Hz), 113.2 (d, *J* = 24.1 Hz). ^19^F NMR (471 MHz, DMSO-*d*_6_) δ -62.8 (s, 3F), -112.5 (td, *J* = 9.1, 5.2 Hz, 1F). HRMS (ESI) m/z calcd for C_16_H_10_F_4_N_3_O_2_ [M + H]^+^ 352.0709, found 352.0706.

**3-Fluoro-*N*-(5-(3-fluorophenyl)-1,3,4-oxadiazol-2-yl)benzamide (7):**

Off-white solid (40 mg, 19%). ^1^H NMR (800 MHz, DMSO-*d*_6_) δ 7.9 – 7.9 (m, 1H), 7.8 (dd, *J* = 19.1, 8.8 Hz, 2H), 7.7 (d, *J* = 9.3 Hz, 1H), 7.7 – 7.7 (m, 1H), 7.6 (q, *J* = 7.4 Hz, 1H), 7.5 (dtd, *J* = 23.6, 8.6, 2.5 Hz, 2H). ^13^C NMR (201 MHz, DMSO-*d*_6_) δ 164.8, 163.3 (d, *J* = 68.3 Hz), 162.1 (d, *J* = 68.3 Hz), 160.2, 158.7, 135.3, 132.2 (d, *J* = 8.0 Hz), 131.2 (d, *J* = 10.0 Hz), 125.8 (d, *J* = 8.0 Hz), 124.9, 122.7, 120.1 (d, *J* = 20.1 Hz), 119.1 (d, *J* = 20.1 Hz), 115.5 (d, *J* = 22.1 Hz), 113.3 (d, *J* = 26.1 Hz). ^19^F NMR (471 MHz, DMSO-*d*_6_) δ -112.5 (q, *J* = 8.7 Hz, 1F), -113.5 (q, *J* = 8.2, 7.8 Hz, 1F). HRMS (ESI) m/z calcd for C_15_H_10_F_2_N_3_O_2_ [M + H]^+^  302.0741, found 302.0744.

**3-Chloro-*N*-(5-(3-fluorophenyl)-1,3,4-oxadiazol-2-yl)benzamide (8):**

Off-white solid (50 mg, 25%). ^1^H NMR (800 MHz, DMSO-*d*_6_) δ 8.1 (s, 1H), 8.0 (d, *J* = 7.8 Hz, 1H), 7.8 (d, *J* = 7.7 Hz, 1H), 7.7 (t, *J* = 8.2 Hz, 2H), 7.7 (q, *J* = 7.4 Hz, 1H), 7.6 (t, *J* = 7.9 Hz, 1H), 7.5 – 7.5 (m, 1H). ^13^C NMR (201 MHz, DMSO-*d*_6_) δ 164.8, 163.3 (d, *J* = 245.2 Hz), 160.3, 158.6, 135.0, 133.9, 132.9, 132.3 (d, *J* = 8.0 Hz), 130.9, 128.5, 127.4, 125.8 (d, *J* = 10.0 Hz), 122.8, 119.1 (d, *J* = 22.1 Hz), 113.2 (d, *J* = 24.1 Hz). ^19^F NMR (471 MHz, DMSO-*d*_6_) δ -112.5 (q, *J* = 8.5 Hz, 1F). HRMS (ESI) m/z calcd for C_15_H_10_ClFN_3_O_2_ [M + H]^+^ 318.0446, found 318.0448.

**3-Bromo-*N*-(5-(3-fluorophenyl)-1,3,4-oxadiazol-2-yl)benzamide (9):**

Off-white solid (46 mg, 26%). ^1^H NMR (800 MHz, DMSO-*d*_6_) δ 8.2 (s, 1H), 8.0 (d, *J* = 7.7 Hz, 1H), 7.8 (d, *J* = 8.0 Hz, 1H), 7.8 – 7.8 (m, 1H), 7.7 – 7.7 (m, 1H), 7.7 (q, *J* = 7.5 Hz, 1H), 7.5 (t, *J* = 7.9 Hz, 1H), 7.5 (td, *J* = 8.7, 2.4 Hz, 1H). ^13^C NMR (201 MHz, DMSO-*d*_6_) δ 164.5, 163.3 (d, *J* = 245.2 Hz), 160.2, 158.6, 135.8, 135.2, 132.2 (d, *J* = 8.0 Hz), 131.4, 131.1, 127.8, 125.8 (d, *J* = 10.0 Hz), 122.7, 122.2, 119.1 (d, *J* = 22.1 Hz), 113.2 (d, *J* = 24.1 Hz). ^19^F NMR (471 MHz, DMSO-*d*_6_) δ -112.4 – -112.5 (m, 1F). HRMS (ESI) m/z calcd for C_15_H_10_BrFN_3_O_2_ [M + H]^+^ 361.9940, found 361.9938.

***N*-(5-(3-Fluorophenyl)-1,3,4-oxadiazol-2-yl)-3-(trifluoromethyl)benzamide (10):**

Off-white solid (49 mg, 27%). ^1^H NMR (800 MHz, DMSO-*d*_6_) δ 8.5 – 8.2 (m, 2H), 8.1 – 7.9 (m, 1H), 7.9 – 7.6 (m, 4H), 7.5 – 7.4 (m, 1H). ^13^C NMR (201 MHz, DMSO-*d*_6_) δ 164.9, 163.3 (d, *J* = 245.2 Hz), 160.1, 158.7, 134.1, 132.8, 132.2, 130.3, 130.0 (q, *J* = 32.2 Hz), 129.6, 125.8, 125.3, 124.9 (q, *J* = 271.4 Hz), 122.7, 119.1 (d, *J* = 22.1 Hz), 113.2 (d, *J* = 24.1 Hz). ^19^F NMR (471 MHz, DMSO-*d*_6_) δ -62.5 (s, 3F), -112.5 (q, *J* = 9.3, 5.7 Hz, 1F). HRMS (ESI) m/z calcd for C_16_H_10_F_4_N_3_O_2_ [M + H]^+^ 352.0709, found 352.0711.

**3,5-Difluoro-*N*-(5-(3-fluorophenyl)-1,3,4-oxadiazol-2-yl)benzamide (11):**

Off-white solid (42 mg, 21%). ^1^H NMR (800 MHz, DMSO-*d*_6_) δ 7.8 – 7.8 (m, 1H), 7.8 – 7.7 (m, 3H), 7.7 (td, *J* = 8.0, 5.9 Hz, 1H), 7.5 (t, *J* = 9.1 Hz, 1H), 7.5 (td, *J* = 8.6, 2.5 Hz, 1H). ^13^C NMR (201 MHz, DMSO-*d*_6_) δ 164.2, 163.3 (d, *J* = 245.2 Hz), 163.2 (d, *J* = 247.2 Hz), 159.9, 158.6, 136.7, 132.3 (d, *J* = 8.0 Hz), 125.7 (d, *J* = 10.0 Hz), 122.8, 119.2 (d, *J* = 20.1 Hz), 113.3 (d, *J* = 24.1 Hz), 112.2 (dd, *J* = 28.1 Hz), 108.7 (t, *J* = 50.2 Hz). ^19^F NMR (471 MHz, DMSO-*d*_6_) δ -109.6 (s, 2F), -112.5 (td, *J* = 9.1, 5.7 Hz, 1F). HRMS (ESI) m/z calcd for C_15_H_9_F_3_N_3_O_2_ [M + H]^+^ 320.0647, found 320.0646.

**3-Chloro-4-fluoro-*N*-(5-(3-fluorophenyl)-1,3,4-oxadiazol-2-yl)benzamide (12):**

Off-white solid (63 mg, 33%). ^1^H NMR (800 MHz, DMSO-*d*_6_) δ 8.3 (dd, *J* = 7.1, 2.2 Hz, 1H), 8.1 (dt, *J* = 5.7, 2.5 Hz, 1H), 7.8 (d, *J* = 7.8 Hz, 1H), 7.7 (dt, *J* = 21.8, 8.4 Hz, 2H), 7.6 (t, *J* = 8.8 Hz, 1H), 7.5 (dt, *J* = 8.6, 4.3 Hz, 1H). ^13^C NMR (201 MHz, DMSO-*d*_6_) δ 164.1, 163.3 (d, *J* = 245.2 Hz), 160.9 (d, *J* = 253.3 Hz), 159.9, 158.6, 132.2 (d, *J* = 8.0 Hz), 131.4, 130.8, 130.2 (d, *J* = 8.0 Hz), 125.8 (d, *J* = 10.0 Hz), 122.7, 120.5 (d, *J* = 18.1 Hz), 119.2 (d, *J* = 20.1 Hz), 117.7 (d, *J* = 22.1 Hz), 113.2 (d, *J* = 24.1 Hz). ^19^F NMR (471 MHz, DMSO-*d*_6_) δ -111.1 (s, 1F), -112.5 (q, *J* = 8.9 Hz, 1F). HRMS (ESI) m/z calcd for C_15_H_9_ClF_2_N_3_O_2_ [M + H]^+^ 336.0351, found 336.0351.

**4-Chloro-3-fluoro-*N*-(5-(3-fluorophenyl)-1,3,4-oxadiazol-2-yl)benzamide (13):**

Off-white solid (51 mg, 27%). ^1^H NMR (800 MHz, DMSO-*d*_6_) δ 8.1 – 8.0 (m, 1H), 7.9 – 7.9 (m, 1H), 7.8 (dd, *J* = 14.5, 7.4 Hz, 2H), 7.7 – 7.6 (m, 2H), 7.5 (d, *J* = 9.1 Hz, 1H). ^13^C NMR (201 MHz, DMSO-*d*_6_) δ 164.4, 163.3 (d, *J* = 245.2 Hz), 159.9, 158.7, 158.0 (d, *J* = 247.2 Hz), 134.1, 132.2, 131.4, 126.0, 125.7, 124.8 (d, *J* = 18.1 Hz), 124.7, 122.7, 119.2 (d, *J* = 22.1 Hz), 117.1 (d, *J* = 24.1 Hz), 113.2 (d, *J* = 24.1 Hz). ^19^F NMR (471 MHz, DMSO-*d*_6_) δ -112.5 (q, *J* = 8.2 Hz, 1F), -116.2 (s, 1F). HRMS (ESI) m/z calcd for C_15_H_9_ClF_2_N_3_O_2_ [M + H]^+^ 336.0351, found 336.0348.

**3-Bromo-4-fluoro-*N*-(5-(3-fluorophenyl)-1,3,4-oxadiazol-2-yl)benzamide (14):**

Off-white solid (50 mg, 29%). ^1^H NMR (800 MHz, DMSO-*d*_6_) δ 8.4 (d, *J* = 4.1 Hz, 1H), 8.2 – 8.0 (m, 1H), 7.8 (d, *J* = 7.7 Hz, 1H), 7.7 – 7.6 (m, 2H), 7.5 (t, *J* = 8.6 Hz, 1H), 7.5 – 7.4 (m, 1H). ^13^C NMR (201 MHz, DMSO-*d*_6_) δ 164.0, 163.3 (d, *J* = 245.2 Hz), 162.0 (d, *J* = 251.2 Hz), 160.0, 158.7, 134.3, 132.2 (d, *J* = 8.0 Hz), 131.2, 130.8 (d, *J* = 8.0 Hz), 125.8 (d, *J* = 10.0 Hz), 122.7, 119.2 (d, *J* = 22.1 Hz), 117.4 (d, *J* = 24.1 Hz), 113.2 (d, *J* = 24.1 Hz), 108.8 (d, *J* = 20.1 Hz). ^19^F NMR (471 MHz, DMSO-*d*_6_) δ -103.1 (s, 1F), -112.5 (q, *J* = 8.2 Hz, 1F). HRMS (ESI) m/z calcd for C_15_H_9_BrF_2_N_3_O_2_ [M + H]^+^ 379.9846, found 379.9849.

**4-Bromo-3-fluoro-*N*-(5-(3-fluorophenyl)-1,3,4-oxadiazol-2-yl)benzamide (15):**

Off-white solid (36 mg, 21%). ^1^H NMR (800 MHz, DMSO-*d*_6_) δ 8.0 (d, *J* = 9.6 Hz, 1H), 7.9 (t, *J* = 7.7 Hz, 1H), 7.8 – 7.8 (m, 1H), 7.8 – 7.8 (m, 1H), 7.7 (dt, *J* = 21.8, 8.3 Hz, 2H), 7.5 (t, *J* = 8.7 Hz, 1H). ^13^C NMR (201 MHz, DMSO-*d*_6_) δ 164.5, 163.3 (d, *J* = 245.2 Hz), 159.9, 159.2 (d, *J* = 245.2 Hz), 158.7, 134.8, 134.3, 132.2 (d, *J* = 8.0 Hz), 126.2, 125.8 (d, *J* = 8.0 Hz), 122.7, 119.2(d, *J* = 22.1 Hz), 116.8 (d, *J* = 24.1 Hz), 113.7 (d, *J* = 22.1 Hz), 113.2 (d, *J* = 24.1 Hz). ^19^F NMR (471 MHz, DMSO-*d*_6_) δ -108.2 (t, *J* = 8.3 Hz, 1F), -112.5 (q, *J* = 8.7 Hz, 1F). HRMS (ESI) m/z calcd for C_15_H_9_BrF_2_N_3_O_2_ [M + H]^+^ 379.9846, found 379.9846.

**4-Fluoro-*N*-(5-(3-fluorophenyl)-1,3,4-oxadiazol-2-yl)-3-(trifluoromethyl)benzamide (16, HSGN-2241):**

Off-white solid (55 mg, 31%). ^1^H NMR (800 MHz, DMSO-*d*_6_) δ 8.4 (ddd, *J* = 42.4, 16.9, 7.1 Hz, 2H), 7.8 (dd, *J* = 17.5, 7.7 Hz, 1H), 7.7 (dt, *J* = 22.1, 7.6 Hz, 3H), 7.5 (dt, *J* = 16.9, 8.3 Hz, 1H). ^13^C NMR (201 MHz, DMSO-*d*_6_) δ 164.3, 163.3 (d, *J* = 245.2 Hz), 162.3 (d, *J* = 259.3 Hz), 159.8, 158.8, 136.2, 132.2, 130.4, 128.2, 125.8, 123.3 (d, *J* = 271.4 Hz), 122.7, 119.2 (d, *J* = 10.0 Hz), 118.2 (d, *J* = 10.0 Hz), 117.5 (q, *J* = 32.2 Hz), 113.2 (d, *J* = 24.1 Hz). ^19^F NMR (471 MHz, DMSO-*d*_6_) δ -61.4 (d, *J* = 12.8 Hz, 3F), -111.1 (s, 1F), -112.5 (td, *J* = 9.3, 5.8 Hz, 1F). HRMS (ESI) m/z calcd for C_16_H_9_F_5_N_3_O_2_ [M + H]^+^ 370.0615, found 370.0615.

**4-Chloro-*N*-(5-(3-fluorophenyl)-1,3,4-oxadiazol-2-yl)-3-(trifluoromethyl)benzamide (17, HSGN-2263):**

Off-white solid (48 mg, 28%). ^1^H NMR (800 MHz, DMSO-*d*_6_) δ 8.5 (d, *J* = 2.1 Hz, 1H), 8.3 (dd, *J* = 8.4, 2.1 Hz, 1H), 8.0 – 7.9 (m, 1H), 7.8 – 7.8 (m, 1H), 7.7 – 7.6 (m, 2H), 7.5 (td, *J* = 8.6, 2.6 Hz, 1H). ^13^C NMR (201 MHz, DMSO-*d*_6_) δ 164.5, 163.3 (d, *J* = 245.2 Hz), 159.7, 158.8, 135.5, 134.3, 132.8, 132.6, 132.3 (d, *J* = 10.0 Hz), 128.1, 127.4 (d, *J* = 30.2 Hz), 125.8 (d, *J* = 8.0 Hz), 123.6 (d, *J* = 271.4 Hz), 122.8, 119.2 (d, *J* = 22.1 Hz), 113.3 (d, *J* = 24.1 Hz). ^19^F NMR (471 MHz, DMSO-*d*_6_) δ -62.5 (s, 3F), -112.5 (td, *J* = 9.4, 5.8 Hz, 1F). HRMS (ESI) m/z calcd for C_16_H_9_ClF_4_N_3_O_2_ [M + H]^+^ 386.0319, found 386.0316.

**3-Fluoro-*N*-(5-(3-fluorophenyl)-1,3,4-oxadiazol-2-yl)-5-(trifluoromethyl)benzamide (18):**

Off-white solid (47 mg, 27%). ^1^H NMR (800 MHz, DMSO-*d*_6_) δ 8.3 (s, 1H), 8.2 – 8.1 (m, 1H), 8.0 (d, *J* = 8.4 Hz, 1H), 7.8 – 7.8 (m, 1H), 7.7 – 7.6 (m, 2H), 7.5 – 7.4 (m, 1H). ^13^C NMR (201 MHz, DMSO-*d*_6_) δ 164.2, 163.3 (d, *J* = 245.2 Hz), 163.0 (d, *J* = 247.2 Hz), 159.7, 158.8, 137.1, 132.3 (d, *J* = 8.0 Hz), 131.9 (q, *J* = 34.2 Hz), 125.7 (d, *J* = 8.0 Hz), 124.1 (q, *J* = 273.4 Hz), 122.8, 121.7, 120.0 (d, *J* = 24.1 Hz), 119.2 (d, *J* = 22.1 Hz), 117.3 (d, *J* = 26.1 Hz), 113.3 (d, *J* = 24.1 Hz). ^19^F NMR (471 MHz, DMSO-*d*_6_) δ -62.6 (s, 3F), -110.6 (t, *J* = 8.7 Hz, 1F), -112.5 (td, *J* = 8.9, 5.5 Hz, 1F). HRMS (ESI) m/z calcd for C_16_H_9_F_5_N_3_O_2_ [M + H]^+^ 370.0615, found 370.0612.

**2-Chloro-4-fluoro-*N*-(5-(3-fluorophenyl)-1,3,4-oxadiazol-2-yl)benzamide (19):**

Off-white solid (34 mg, 18%). ^1^H NMR (800 MHz, DMSO-*d*_6_) δ 7.8 – 7.8 (m, 2H), 7.7 – 7.6 (m, 2H), 7.6 – 7.5 (m, 1H), 7.5 – 7.4 (m, 1H), 7.4 – 7.3 (m, 1H). ^13^C NMR (201 MHz, DMSO-*d*_6_) δ 164.1, 163.9 (d, *J* = 251.2 Hz), 163.3 (d, *J* = 245.2 Hz), 160.2, 157.9, 132.3, 132.2 (d, *J* = 10.0 Hz), 131.8 (d, *J* = 10.0 Hz), 131.6, 125.8 (d, *J* = 10.0 Hz), 122.7, 119.1(d, *J* = 21.1 Hz), 117.8 (d, *J* = 24.1 Hz), 115.0 (d, *J* = 22.1 Hz), 113.2 (d, *J* = 24.1 Hz). ^19^F NMR (471 MHz, DMSO-*d*_6_) δ -108.7 (q, *J* = 7.9 Hz, 1F), -112.5 – -112.6 (m, 1F). HRMS (ESI) m/z calcd for C_15_H_9_ClF_2_N_3_O_2_ [M + H]^+^ 336.0351, found 336.0350.

**4-Chloro-2-fluoro-*N*-(5-(3-fluorophenyl)-1,3,4-oxadiazol-2-yl)benzamide (20):**

Off-white solid (40 mg, 21%). ^1^H NMR (800 MHz, DMSO-*d*_6_) δ 7.8 – 7.8 (m, 2H), 7.7 – 7.6 (m, 2H), 7.6 (d, *J* = 10.1 Hz, 1H), 7.5 – 7.4 (m, 2H). ^13^C NMR (201 MHz, DMSO-*d*_6_) δ 163.3 (d, *J* = 245.2 Hz), 162.2, 160.5 (d, *J* = 255.3 Hz), 160.2, 158.0, 137.9 (d, *J* = 10.0 Hz), 132.2 (d, *J* = 8.0 Hz), 132.0, 125.8 (d, *J* = 8.0 Hz), 125.4, 122.7, 122.0 (d, *J* = 14.1 Hz), 119.1 (d, *J* = 20.1 Hz), 117.5 (d, *J* = 26.1 Hz), 113.2 (d, *J* = 24.1 Hz). ^19^F NMR (471 MHz, DMSO-*d*_6_) δ -111.6 (s, 1F), -112.5 – -112.5 (m, 1F). HRMS (ESI) m/z calcd for C_15_H_9_ClF_2_N_3_O_2_ [M + H]^+^ 336.0351, found 336.0353.

**2-Bromo-4-fluoro-*N*-(5-(3-fluorophenyl)-1,3,4-oxadiazol-2-yl)benzamide (21):**

Off-white solid (38 mg, 22%). ^1^H NMR (800 MHz, DMSO-*d*_6_) δ 7.8 – 7.8 (m, 1H), 7.7 (t, *J* = 7.3 Hz, 1H), 7.7 (d, *J* = 8.8 Hz, 1H), 7.7 – 7.7 (m, 2H), 7.5 (t, *J* = 8.7 Hz, 1H), 7.4 (t, *J* = 8.6 Hz, 1H). ^13^C NMR (201 MHz, DMSO-*d*_6_) δ 164.9, 163.6 (d, *J* = 253.3 Hz), 163.3 (d, *J* = 245.2 Hz), 160.2, 157.9, 133.8, 132.3 (d, *J* = 10.0 Hz), 131.6 (d, *J* = 10.0 Hz), 125.8 (d, *J* = 8.0 Hz), 122.7, 120.8 (d, *J* = 24.1 Hz), 120.4 (d, *J* = 10.0 Hz), 119.1 (d, *J* = 22.1 Hz), 115.4 (d, *J* = 22.1 Hz), 113.2 (d, *J* = 24.1 Hz). ^19^F NMR (471 MHz, DMSO-*d*_6_) δ -109.0 (q, *J* = 7.8 Hz, 1F), -112.5 (s, 1F). HRMS (ESI) m/z calcd for C_15_H_9_BrF_2_N_3_O_2_ [M + H]^+^ 379.9846, found 379.9850.

**4-Bromo-2-fluoro-*N*-(5-(3-fluorophenyl)-1,3,4-oxadiazol-2-yl)benzamide (22):**

Off-white solid (50 mg, 29%). ^1^H NMR (800 MHz, DMSO-*d*_6_) δ 7.8 – 7.8 (m, 1H), 7.8 – 7.7 (m, 2H), 7.7 – 7.6 (m, 2H), 7.6 – 7.6 (m, 1H), 7.5 (t, *J* = 8.5 Hz, 1H). ^13^C NMR (201 MHz, DMSO-*d*_6_) δ 163.3 (d, *J* = 245.2 Hz), 162.4, 160.4 (d, *J* = 255.3 Hz), 160.2, 158.1, 132.3, 132.2 (d, *J* = 8.0 Hz), 128.3, 126.1 (d, *J* = 10.0 Hz), 125.8 (d, *J* = 10.0 Hz), 122.8, 122.4 (d, *J* = 12.1 Hz), 120.3 (d, *J* = 26.1 Hz), 119.1 (d, *J* = 20.1 Hz), 113.2 (d, *J* = 24.1 Hz). ^19^F NMR (471 MHz, DMSO-*d*_6_) δ -111.7 (t, *J* = 8.9 Hz, 1F), -112.5 (s, 1F). HRMS (ESI) m/z calcd for C_15_H_9_BrF_2_N_3_O_2_ [M + H]^+^ 379.9846, found 379.9847.

**2-Fluoro-*N*-(5-(3-fluorophenyl)-1,3,4-oxadiazol-2-yl)-4-iodobenzamide (23):**

Off-white solid (32 mg, 25%). ^1^H NMR (800 MHz, DMSO-*d*_6_) δ 7.8 (d, *J* = 9.6 Hz, 1H), 7.8 – 7.8 (m, 1H), 7.8 – 7.7 (m, 1H), 7.7 – 7.6 (m, 2H), 7.5 (t, *J* = 7.8 Hz, 1H), 7.5 (t, *J* = 8.5 Hz, 1H). ^13^C NMR (201 MHz, DMSO-*d*_6_) δ 163.3 (d, *J* = 245.2 Hz), 162.6, 160.2, 159.9 (d, *J* = 257.3 Hz), 158.0, 134.1, 132.2 (d, *J* = 8.0 Hz), 132.0, 125.8, 125.7, 125.6, 122.7, 122.6 (d, *J* = 14.1 Hz), 119.1 (d, *J* = 20.1 Hz), 113.2 (d, *J* = 24.1 Hz), 99.6 (d, *J* = 8.0 Hz). ^19^F NMR (471 MHz, DMSO-*d*_6_) δ -112.5 (q, *J* = 8.8, 8.4 Hz, 2F). HRMS (ESI) m/z calcd for C_15_H_10_FIN_3_O_2_ [M + H]^+^ 409.9802, found 409.9805.

**4-Fluoro-*N*-(5-(4-fluorophenyl)-1,3,4-oxadiazol-2-yl)-3-(trifluoromethyl)benzamide (24):**

Off-white solid (56 mg, 32%). ^1^H NMR (800 MHz, DMSO-*d*_6_) δ 8.4 (d, *J* = 6.9 Hz, 1H), 8.4 – 8.4 (m, 1H), 8.0 (t, *J* = 6.5 Hz, 2H), 7.7 (t, *J* = 9.6 Hz, 1H), 7.4 (t, *J* = 8.5 Hz, 2H). ^13^C NMR (201 MHz, DMSO-*d*_6_) δ 165.1 (d, *J* = 249.2 Hz), 164.4, 162.3 (d, *J* = 261.3 Hz), 160.1, 158.6, 136.1 (d, *J* = 10.0 Hz), 130.3, 129.2 (d, *J* = 10.0 Hz), 128.2, 123.3 (q, *J* = 271.4 Hz), 120.4, 118.2 (d, *J* = 22.1 Hz), 117.4 (q, *J* = 44.2 Hz), 117.1 (d, *J* = 22.1 Hz). ^19^F NMR (471 MHz, DMSO-*d*_6_) δ -61.5 (d, *J* = 13.0 Hz, 3F), -108.8 (s, 1F), -111.1 (s, 1F). HRMS (ESI) m/z calcd for C_16_H_9_F_5_N_3_O_2_ [M + H]^+^ 370.0615, found 370.0612.

**4-Fluoro-*N*-(5-(2-fluorophenyl)-1,3,4-oxadiazol-2-yl)-3-(trifluoromethyl)benzamide (25):**

Off-white solid (33 mg, 19%). ^1^H NMR (800 MHz, DMSO-*d*_6_) δ 8.5 (d, *J* = 6.7 Hz, 1H), 8.4 (t, *J* = 6.6 Hz, 1H), 8.0 (t, *J* = 7.6 Hz, 1H), 7.8 – 7.6 (m, 2H), 7.5 – 7.4 (m, 2H). ^13^C NMR (201 MHz, DMSO-*d*_6_) δ 164.1, 162.3 (d, *J* = 259.3 Hz), 160.2 (d, *J* = 255.3 Hz), 158.7, 157.4, 136.2 (d, *J* = 10.0 Hz), 134.4 (d, *J* = 8.0 Hz), 130.3, 129.6, 128.2, 125.7, 123.3 (q, *J* = 271.4 Hz), 118.2 (d, *J* = 20.1 Hz), 117.5 (d, *J* = 20.1 Hz), 117.2 (q, *J* = 34.2 Hz) 112.1 (d, *J* = 12.1 Hz). ^19^F NMR (471 MHz, DMSO-*d*_6_) δ -61.4 (d, *J* = 13.1 Hz, 3F), -111.0 (s, 1F), -112.0 (q, *J* = 11.7, 5.9 Hz, 1F). HRMS (ESI) m/z calcd for C_16_H_9_F_5_N_3_O_2_ [M + H]^+^ 370.0615, found 370.0616.

**4-Fluoro-*N*-(5-phenyl-1,3,4-oxadiazol-2-yl)-3-(trifluoromethyl)benzamide (26):**

Off-white solid (37 mg, 22%). ^1^H NMR (800 MHz, DMSO-*d*_6_) δ 8.5 (d, *J* = 6.7 Hz, 1H), 8.4 – 8.4 (m, 1H), 8.0 (d, *J* = 7.1 Hz, 2H), 7.7 (t, *J* = 9.7 Hz, 1H), 7.6 – 7.6 (m, 3H). ^13^C NMR (201 MHz, DMSO-*d*_6_) δ 164.5, 162.3 (d, *J* = 259.3 Hz), 160.7, 158.6, 136.2 (d, *J* = 10.0 Hz), 132.2, 130.4, 129.8, 128.2, 126.5, 123.7, 123.3 (q, *J* = 271.4 Hz), 118.2 (d, *J* = 20.1 Hz), 117.4 (q, *J* = 46.2 Hz). ^19^F NMR (471 MHz, DMSO-*d*_6_) δ -61.4 (d, *J* = 12.8 Hz, 3F), -111.1 (s, 1F). HRMS (ESI) m/z calcd for C_16_H_10_F_4_N_3_O_2_ [M + H]^+^ 352.0709, found 352.0707.

**4-Fluoro-*N*-(5-(thiophen-2-yl)-1,3,4-oxadiazol-2-yl)-3-(trifluoromethyl)benzamide (27):**

Off-white solid (48 mg, 28%). ^1^H NMR (800 MHz, DMSO-*d*_6_) δ 8.4 (d, *J* = 6.8 Hz, 1H), 8.4 (dd, *J* = 7.0, 3.6 Hz, 1H), 7.9 (d, *J* = 4.9 Hz, 1H), 7.8 (s, 1H), 7.7 (t, *J* = 9.7 Hz, 1H), 7.3 (s, 1H). ^13^C NMR (201 MHz, DMSO-*d*_6_) δ 164.2, 162.3 (d, *J* = 259.3 Hz), 158.0, 157.3, 136.1 (d, *J* = 10.0 Hz), 131.4, 130.3, 130.1, 129.0, 128.2, 124.7, 123.3 (q, *J* = 273.4 Hz), 118.2 (d, *J* = 20.1 Hz), 117.4 (q, *J* = 46.2 Hz). ^19^F NMR (471 MHz, DMSO-*d*_6_) δ -61.5 (d, *J* = 12.9 Hz, 3F), -111.0 (s, 1F). HRMS (ESI) m/z calcd for C_14_H_8_F_4_N_3_O_2_S [M + H]^+^ 358.0273, found 358.0273.

***N*-(5-(2,4-Dimethylthiazol-5-yl)-1,3,4-oxadiazol-2-yl)-4-fluoro-3-(trifluoromethyl)benzamide (28):**

Off-white solid (42 mg, 23%). ^1^H NMR (800 MHz, DMSO-*d*_6_) δ 8.6 – 8.4 (m, 1H), 8.4 (dt, *J* = 5.5, 2.8 Hz, 1H), 7.7 (t, *J* = 9.5 Hz, 1H), 2.7 (s, 3H), 2.7 (s, 3H). ^13^C NMR (201 MHz, DMSO-*d*_6_) δ 168.0, 163.9, 162.3 (d, *J* = 259.3 Hz), 157.8, 156.2, 155.0, 136.2, 130.1, 128.2, 123.3 (q, *J* = 273.4 Hz), 118.2 (d, *J* = 20.1 Hz), 117.4 (q, *J* = 38.2 Hz), 113.7, 19.3, 17.1. ^19^F NMR (471 MHz, DMSO-*d*_6_) δ -61.4 (d, *J* = 12.9 Hz, 3F), -110.9 (s, 1F). HRMS (ESI) m/z calcd for C_15_H_11_F_4_N_4_O_2_S [M + H]^+^ 387.0539, found 387.0538.

**4-Fluoro-*N*-(5-(pyrazin-2-yl)-1,3,4-oxadiazol-2-yl)-3-(trifluoromethyl)benzamide (29):**

Off-white solid (49 mg, 29%). ^1^H NMR (800 MHz, DMSO-*d*_6_) δ 9.3 (d, *J* = 1.5 Hz, 1H), 8.9 – 8.7 (m, 2H), 8.5 (dd, *J* = 6.9, 2.3 Hz, 1H), 8.4 (ddd, *J* = 8.5, 4.7, 2.3 Hz, 1H), 7.7 (dd, *J* = 10.5, 8.7 Hz, 1H). ^13^C NMR (201 MHz, DMSO-*d*_6_) δ 163.6, 162.4 (d, *J* = 259.3 Hz), 159.4, 158.7, 147.1, 145.3, 143.5, 139.4, 136.3 (d, *J* = 10.0 Hz), 130.1, 128.3, 123.3 (q, *J* = 273.4 Hz), 118.3 (d, *J* = 20.1 Hz), 117.4 (q, *J* = 38.2 Hz). ^19^F NMR (471 MHz, DMSO-*d*_6_) δ -61.4 (d, *J* = 12.9 Hz, 3F), -110.8 (s, 1F). HRMS (ESI) m/z calcd for C_14_H_8_F_4_N_5_O_2_ [M + H]^+^ 354.0614, found 354.0617.

**4-Fluoro-*N*-(5-(pyridin-2-yl)-1,3,4-oxadiazol-2-yl)-3-(trifluoromethyl)benzamide (30):**

Off-white solid (52 mg, 31%). ^1^H NMR (800 MHz, DMSO-*d*_6_) δ 8.8 (ddd, *J* = 4.7, 1.8, 1.0 Hz, 1H), 8.5 (dd, *J* = 6.9, 2.3 Hz, 1H), 8.4 (ddd, *J* = 8.6, 4.8, 2.3 Hz, 1H), 8.2 – 8.1 (m, 1H), 8.0 (td, *J* = 7.7, 1.7 Hz, 1H), 7.7 – 7.7 (m, 1H), 7.6 (ddd, *J* = 7.6, 4.8, 1.2 Hz, 1H). ^13^C NMR (201 MHz, DMSO-*d*_6_) δ 163.7, 162.3 (d, *J* = 259.3 Hz), 160.4, 159.0, 150.6, 143.1, 138.1, 136.2 (d, *J* = 10.0 Hz), 130.2, 128.2, 126.5, 123.3 (q, *J* = 273.4 Hz), 122.8, 118.3 (d, *J* = 20.1 Hz), 117.3 (q, *J* = 38.2 Hz). ^19^F NMR (471 MHz, DMSO-*d*_6_) δ -61.4 (d, *J* = 12.9 Hz, 3F), -110.9 (s, 1F). HRMS (ESI) m/z calcd for C_15_H_9_F_4_N_4_O_2_ [M + H]^+^ 353.0662, found 353.0658.

**4-Fluoro-*N*-(5-(pyridin-3-yl)-1,3,4-oxadiazol-2-yl)-3-(trifluoromethyl)benzamide (31):**

Off-white solid (44 mg, 26%). ^1^H NMR (800 MHz, DMSO-*d*_6_) δ 9.1 (d, *J* = 2.2 Hz, 1H), 8.8 (dd, *J* = 4.8, 1.7 Hz, 1H), 8.5 (dd, *J* = 7.0, 2.3 Hz, 1H), 8.4 (ddd, *J* = 8.6, 4.7, 2.3 Hz, 1H), 8.3 (dt, *J* = 7.9, 2.0 Hz, 1H), 7.7 (dd, *J* = 10.5, 8.7 Hz, 1H), 7.6 (ddd, *J* = 8.0, 4.8, 0.8 Hz, 1H). ^13^C NMR (201 MHz, DMSO-*d*_6_) δ 164.1, 162.3 (d, *J* = 259.3 Hz), 158.9, 152.7, 147.2, 136.2 (d, *J* = 10.0 Hz), 134.0, 130.3, 128.2, 124.7, 123.3 (q, *J* = 273.4 Hz), 120.3, 118.2 (d, *J* = 22.1 Hz), 117.4 (q, *J* = 38.2 Hz). ^19^F NMR (471 MHz, DMSO-*d*_6_) δ -61.4 (d, *J* = 12.9 Hz, 3F), -111.0 (s, 1F). HRMS (ESI) m/z calcd for C_15_H_9_F_4_N_4_O_2_ [M + H]^+^ 353.0662, found 353.0663.

**4-Fluoro-*N*-(5-(pyridin-4-yl)-1,3,4-oxadiazol-2-yl)-3-(trifluoromethyl)benzamide (32):**

Off-white solid (55 mg, 33%). ^1^H NMR (800 MHz, DMSO-*d*_6_) δ 8.9 – 8.8 (m, 2H), 8.5 (dd, *J* = 6.9, 2.3 Hz, 1H), 8.4 (ddd, *J* = 8.6, 4.7, 2.3 Hz, 1H), 7.9 – 7.8 (m, 2H), 7.7 (dd, *J* = 10.5, 8.7 Hz, 1H). ^13^C NMR (201 MHz, DMSO-*d*_6_) δ 164.0, 162.4 (d, *J* = 259.3 Hz), 159.2, 151.3, 136.2 (d, *J* = 10.0 Hz), 130.8, 130.2, 128.2, 123.3 (q, *J* = 273.4 Hz), 120.0, 118.2 (d, *J* = 22.1 Hz), 117.4 (q, *J* = 38.2 Hz). ^19^F NMR (471 MHz, DMSO-*d*_6_) δ -61.5 (d, *J* = 12.9 Hz, 3F), -110.9 (s, 1F). HRMS (ESI) m/z calcd for C_15_H_9_F_4_N_4_O_2_ [M + H]^+^ 353.0662, found 353.0665.

**II. Biological Analysis:**

**Table S1.** Bacterial strains, sources and characteristics

| Bacterial Strain | Source | Characteristics |
| --- | --- | --- |
| *Staphylococcus aureus* ATCC 25923 | ATCC | Methicillin susceptible. Reference strain for antibiotic susceptibility testing |
| MRSA USA 300 | ATCC/BEI resources | Methicillin resistant. Most prevalent MRSA clinical infection strain in the U.S |
| MRSA ATCC 33592 | William Wuest, Emory University | Methicillin resistant, Gentamicin resistant. SCCmec: Type III |
| *Enterococcus faecalis* ATCC 29212 | ATCC | Reference strain for antibiotic susceptibility testing. |
| *Enterococcus faecalis* ATCC 51575 (VRE) | ATCC | Presence of *vanB*.  Resistant to gentamicin, streptomycin, and vancomycin. Sensitive to teicoplanin. |
| *Enterococcus faecium* ATCC 700221 (VRE) | ATCC | Presence of *vanA*.  Resistant to vancomycin and teicoplanin |
| *Listeria monocytogenes* ATCC 19115 | ATCC | Reference strain for antibiotic susceptibility testing. |

ATCC = American Type Culture Collection

**Table S2.** MRSA clinical isolates

| Strain | PFGE Pattern / MLST Type | spa Type eGenomics (Ridom) | SCCmec | Other Genetic Characterization | Additional Non MRSA resistance (dru Type) | Oxacillin MIC in μg/mL |
| --- | --- | --- | --- | --- | --- | --- |
| ARLG 1561 | USA 300 | 1 | IVa | ACME+, pvl genes+ | Ery | NT |
| ARLG 1567 | USA 300 | 1 | IVa | ACME+, pvl genes+ | Ery, Cipro | NT |
| ARLG 1568 | USA 300 | 1 | IVa | ACME+, pvl genes+, MupA gene+ | Mup, Ery, Cipro | NT |
| ARLG 1569 | USA 300 | 1 | IVa | ACME+, pvl genes+, MupA gene+ | Mup, Ery, Cipro, Clinda | NT |
| ARLG 1570 | USA 300 | 1 | IVa | ACME+, pvl genes+, MupA gene+ | Mup, Ery, Cipro, Clinda | NT |
| ARLG-1663 | ST239 | 351 (t030) | 3A.1.4 | ccrC+, dcs+, Hg-J+, mecl+ | (dt10a) | 512 |
| ARLG-1649 | ST1312 | 351 (t030) | 3A.1.4 | ccrC+, dcs+, Hg-J+, mecl+ | (dt10g) | 256 |
| ARLG-1664 | ST239 | 351 (t030) | 16691 | ccrC+, Hg-J+, mecl+ | (dt9x) | 256 |

* ACME = arginine catabolic mobile element, PFGE = Pulsed field gel electrophoresis, MLST = Multilocus sequence typing, pvl = panton valentin leucocidin, Ery = Erythromycin, Mup = Mupirocin, Cipro = Ciprofloxacin, Clinda = Constitutive clindamycin resistance, ARLG = Antibacterial Resistance Leadership Group

**Table S3.** Sequence of primers used in RT-PCR.

| **Primer Name** | **Sequence (5’- 3’)** | **Source** |
| --- | --- | --- |
| eno Forward | AAACTGCCGTAGGTGACGAA | Kot et al.[1] |
| eno Reverse | TGTTTCAACAGCATCTTCAGTACCTT | Kot et al.[1] |
| ebps Forward | ACATTCAAATGACGCTCAAAACAAAAGT | Kot et al.[1] |
| ebps Reverse | CTTATCTTGAGACGCTTTATCCTCAGT | Kot et al.[1] |
| fib Forward | GAATATGGTGCACGTCCACAATT | Kot et al.[1] |
| fib Reverse | AAGATTTTGAGCTTGAATCAATTTTTGTTCTTTTT | Kot et al.[1] |
| cna Forward | GACTTACCGAAGTATGATGAAGGAAAGA | Kot et al.[1] |
| cna Reverse | ACCGTTGATGTCTGTTGTGTAGTC | Kot et al.[1] |
| icaA Forward | CAATACTATTTCGGGTGTCTTCACTCT | Kot et al.[1] |
| icaA Reverse | CAAGAAACTGCAATATCTTCGGTAATCAT | Kot et al.[1] |
| icaD Forward | TCAAGCCCAGACAGAGGGAATA | Kot et al.[1] |
| icaD Reverse | ACACGATATAGCGATAAGTGCTGTTT | Kot et al.[1] |
| rpoB Forward | CAGCTGACGAAGAAGATAGCTATGT | Kot et al.[1] |
| rpoB Reverse | ACTTCATCATCCATGAAACGACCAT | Kot et al.[1] |

**Table S4.** Sources of reagents used in biochemical assays.

| **Reagent Name** | **Source** |
| --- | --- |
| Linezolid | Chem-Impex International, Wood Dale, IL, USA |
| Vancomycin HCl | Gold Biotechnology, St. Louis, MO, USA |
| Ciprofloxacin | Sigma Aldrich, St. Louis, MO, USA |
| Daptomycin | AK Scientific, Union City, CA, USA |
| DiSC3(5) (3,3'-Dipropylthiadicarbocyanine Iodide) | Thermo Fisher Scientific, Waltham, MA, USA |
| Potassium Chloride | Sigma Aldrich, St. Louis, MO, USA |
| Potassium-binding benzofuran isophthalate (PBFI) | Sigma Aldrich, St. Louis, MO, USA |
| Sodium Acetate | Sigma Aldrich, St. Louis, MO, USA |
| acetonitrile/methanol/300 mM ammonium acetate | Sigma Aldrich, St. Louis, MO, USA |

**Figure S1**. Effects of **HSGN-2241** on membrane permeability in *S. aureus* at 2X MIC, 5X MIC, and 10X MIC concentrations using SYTOX green dye. Increase in fluorescence indicates permeability. Bithionol (4 µg/mL) is used as positive control while 1% DMSO is used as negative control. Dotted lines indicate standard deviation.

**III. ^1^H, ^13^C, and ^19^F Spectra of Analogs:**

**
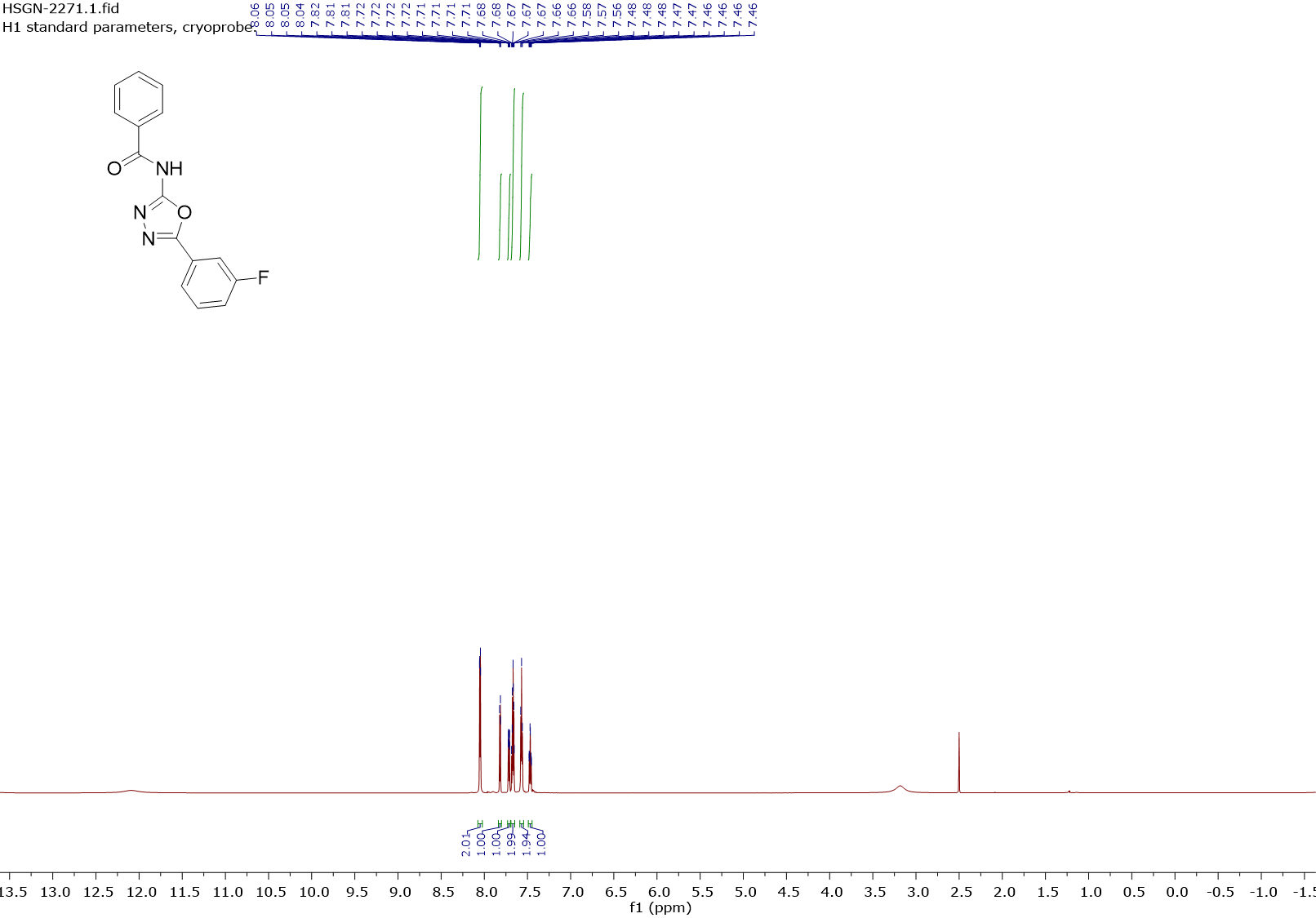
**

**
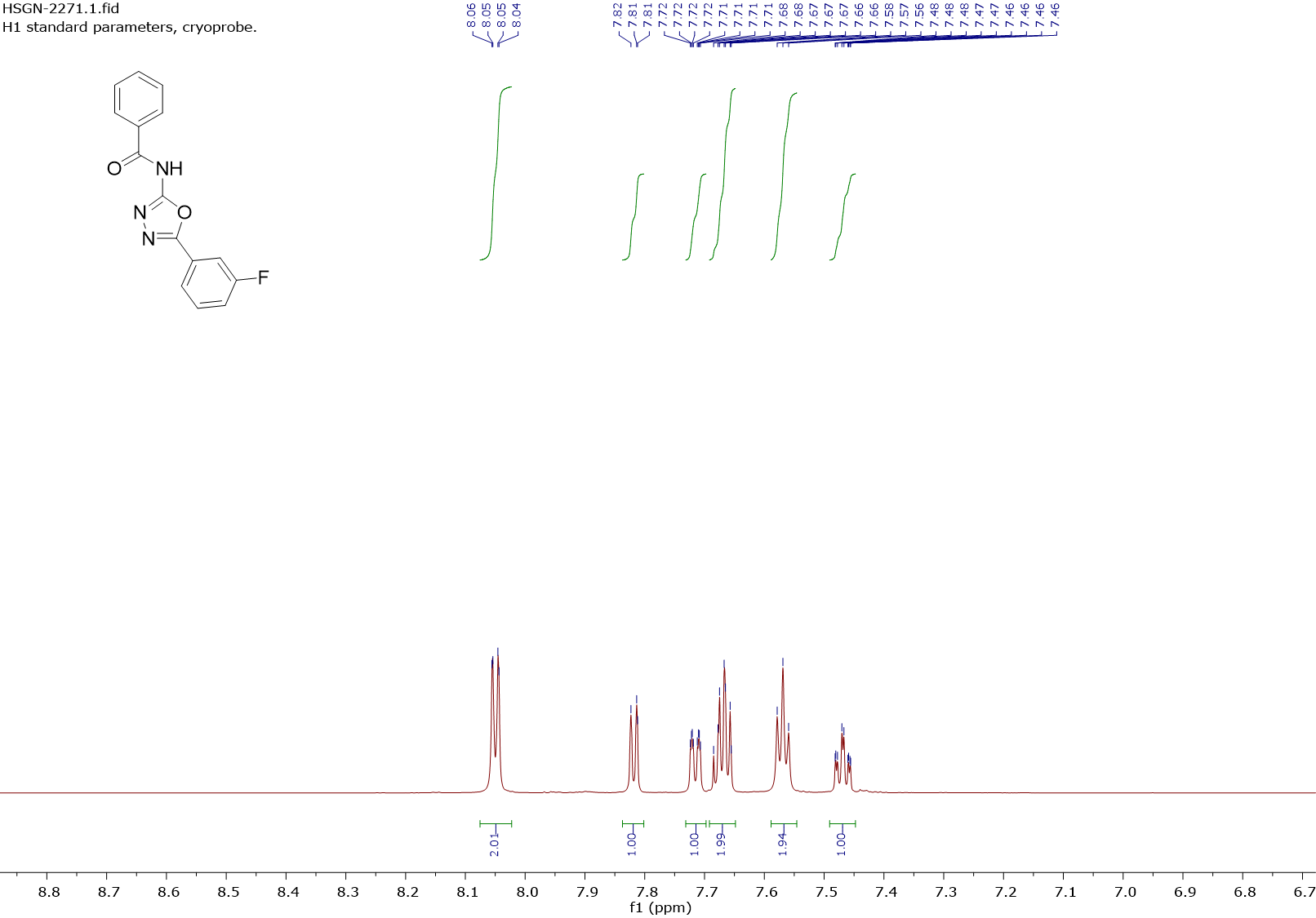
**

**
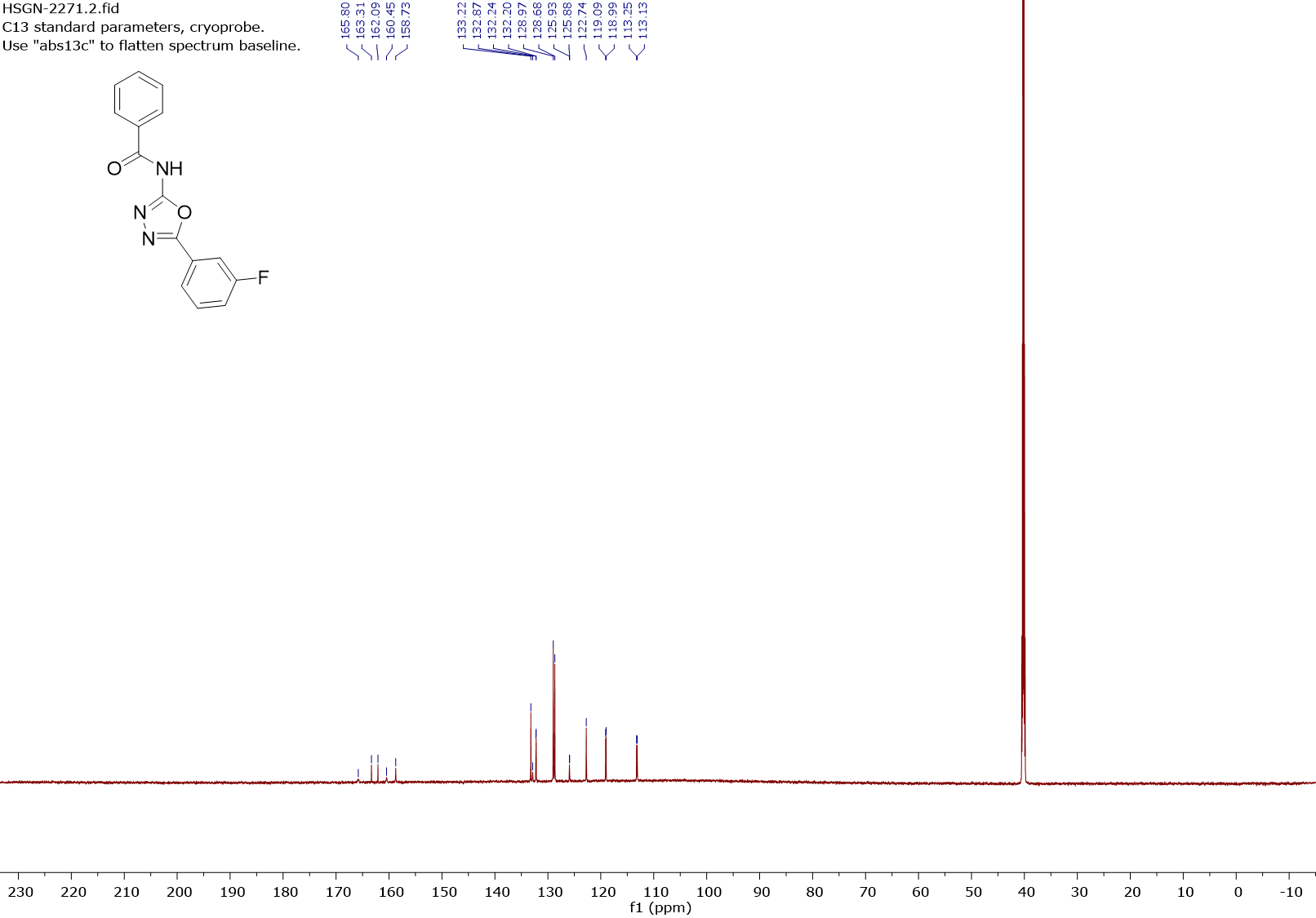
**

**
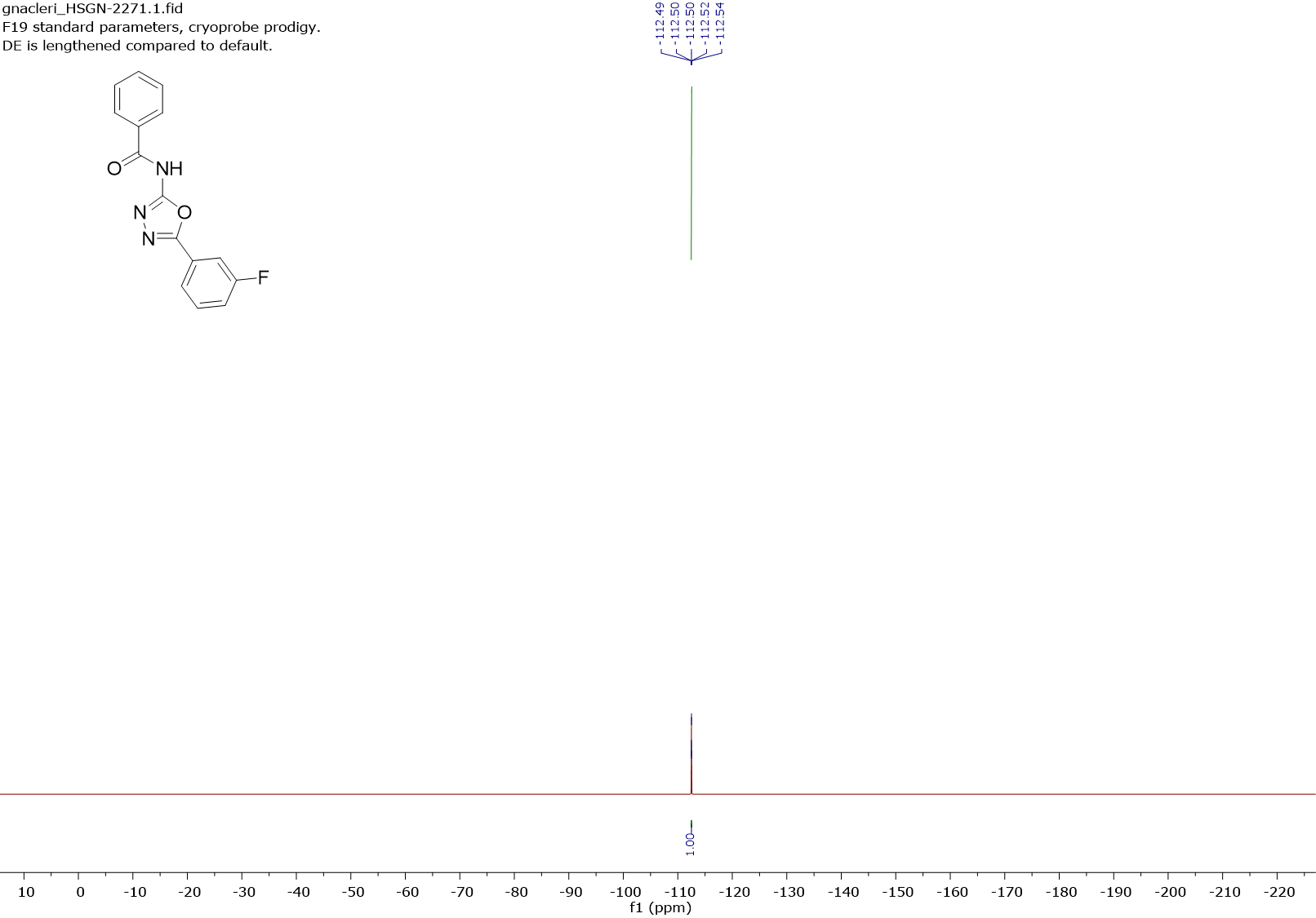
**


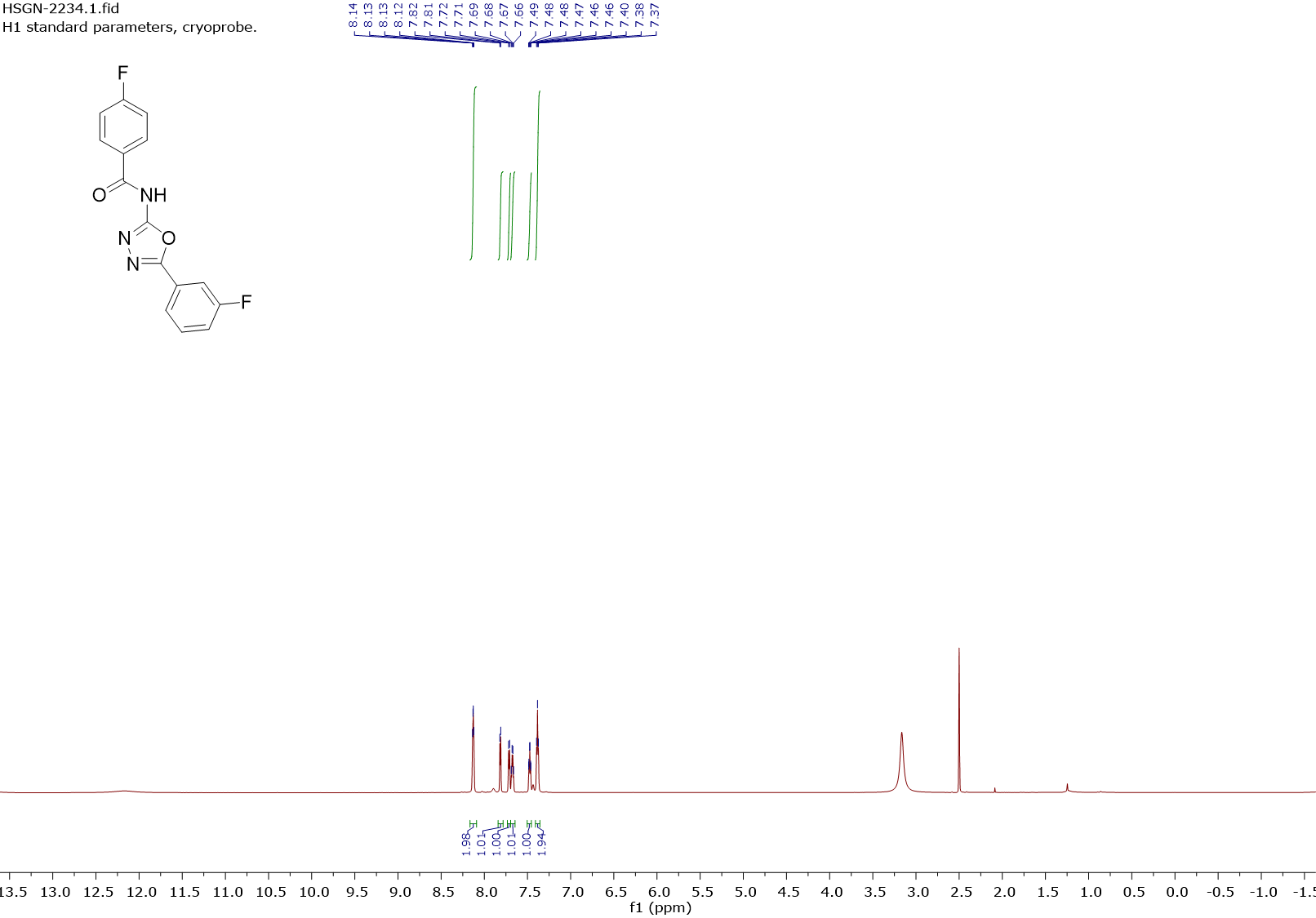


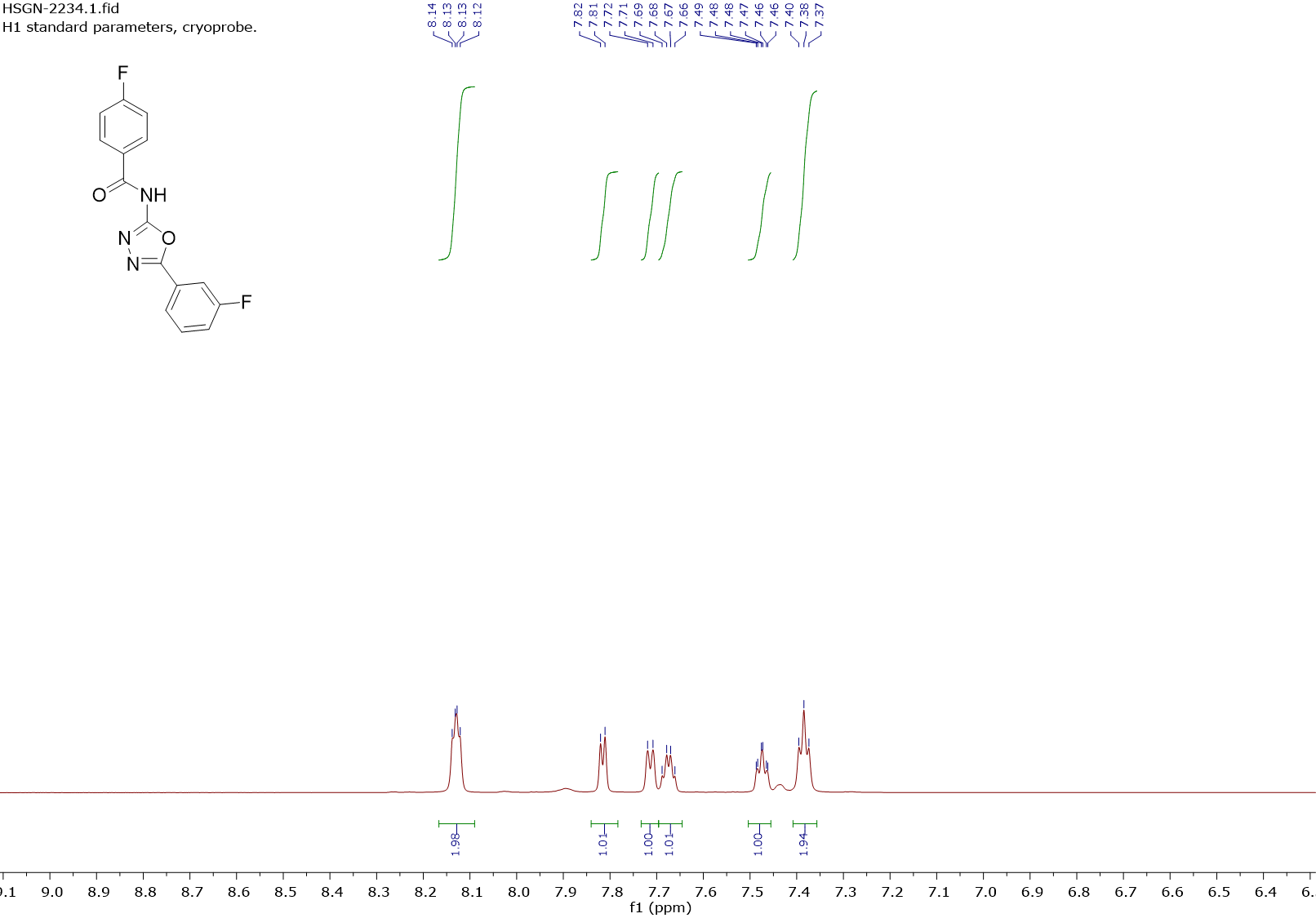


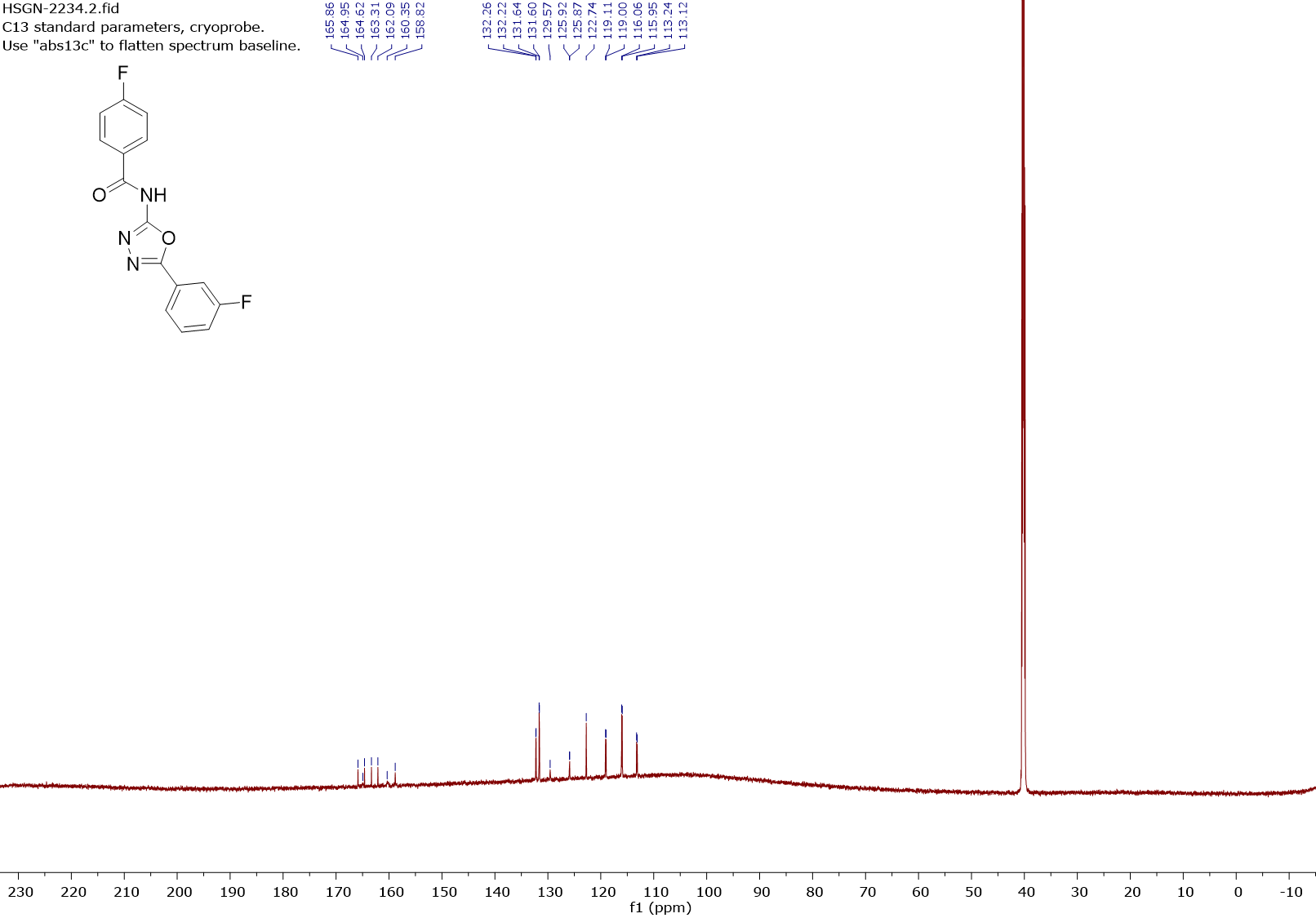


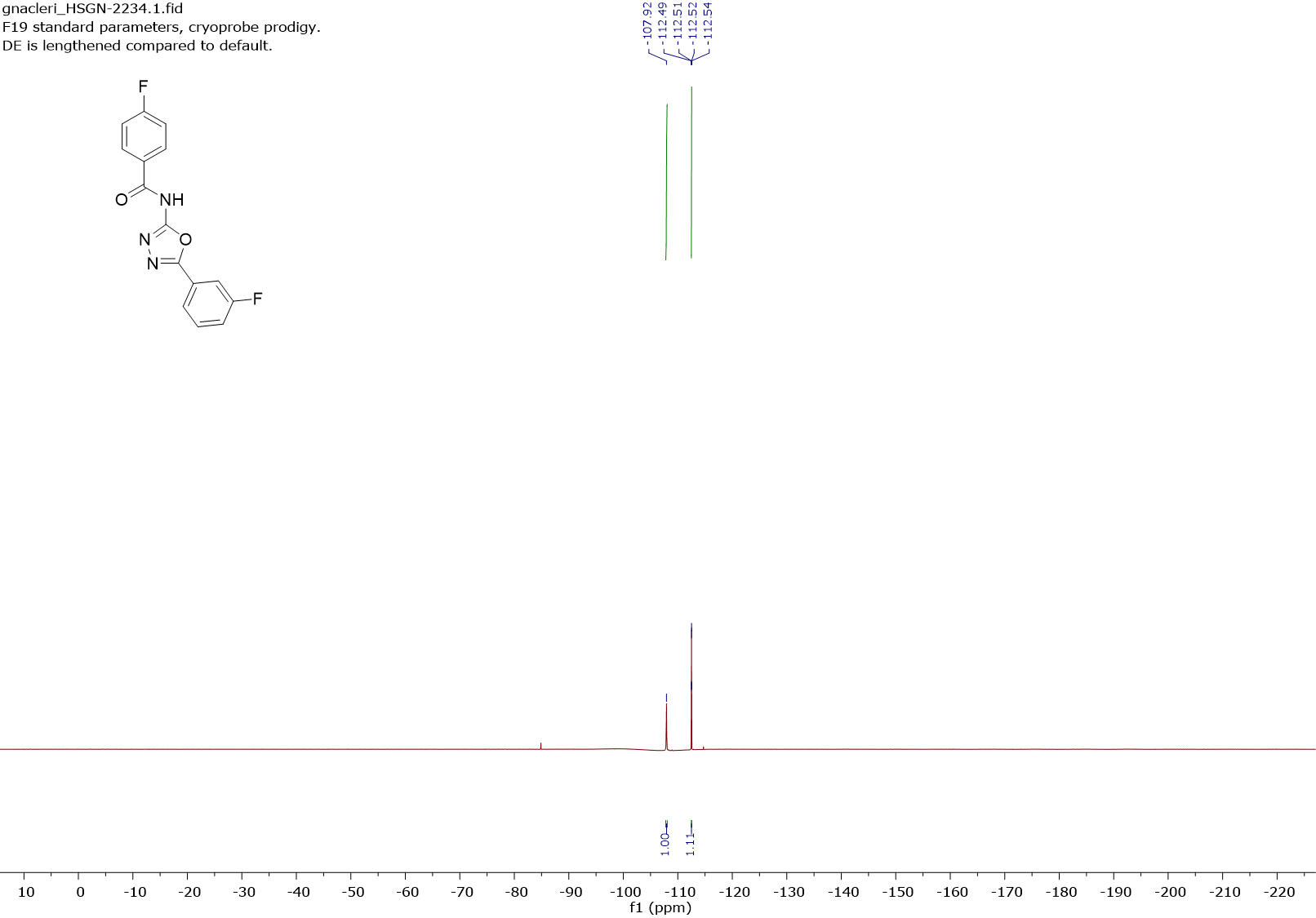


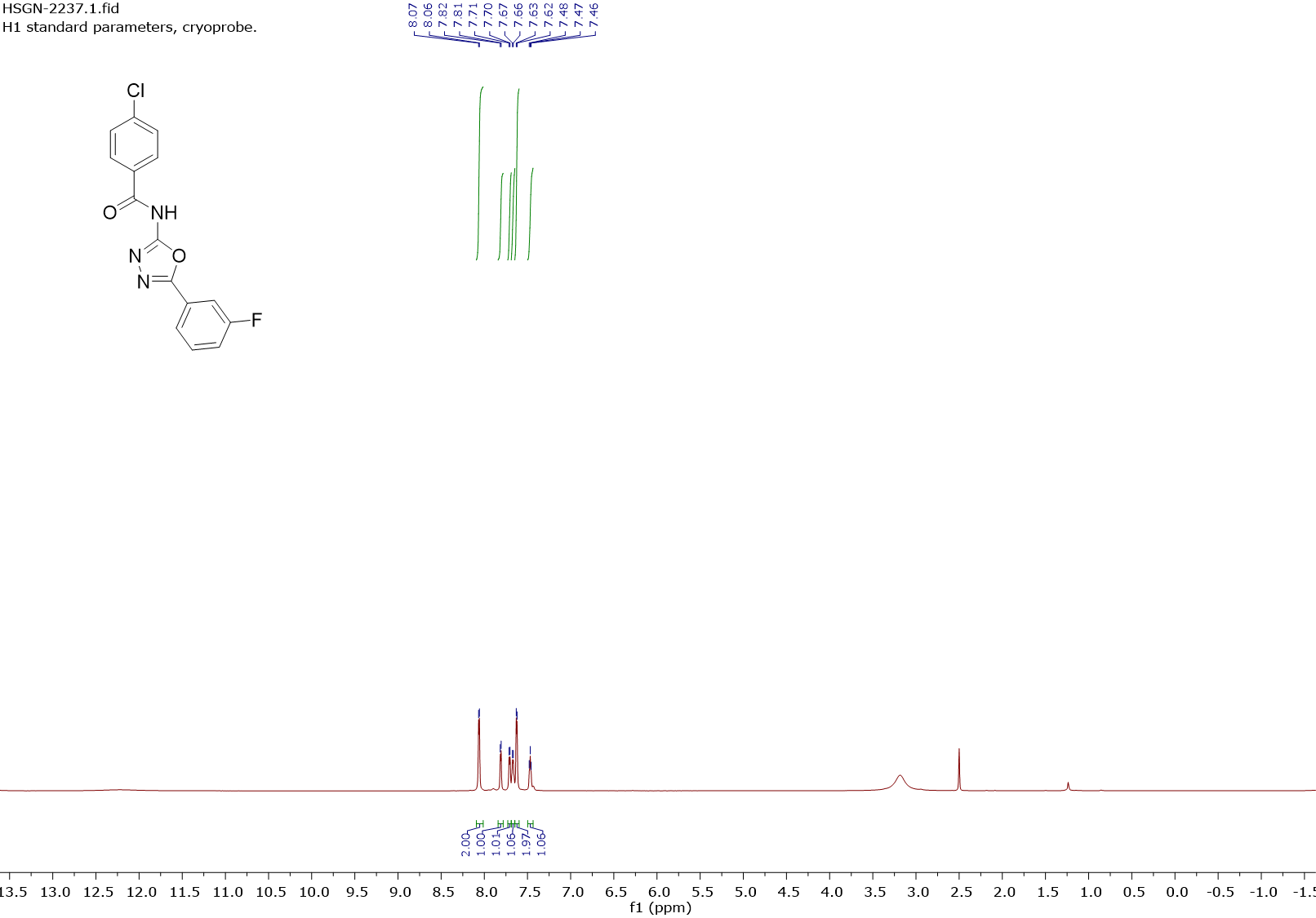


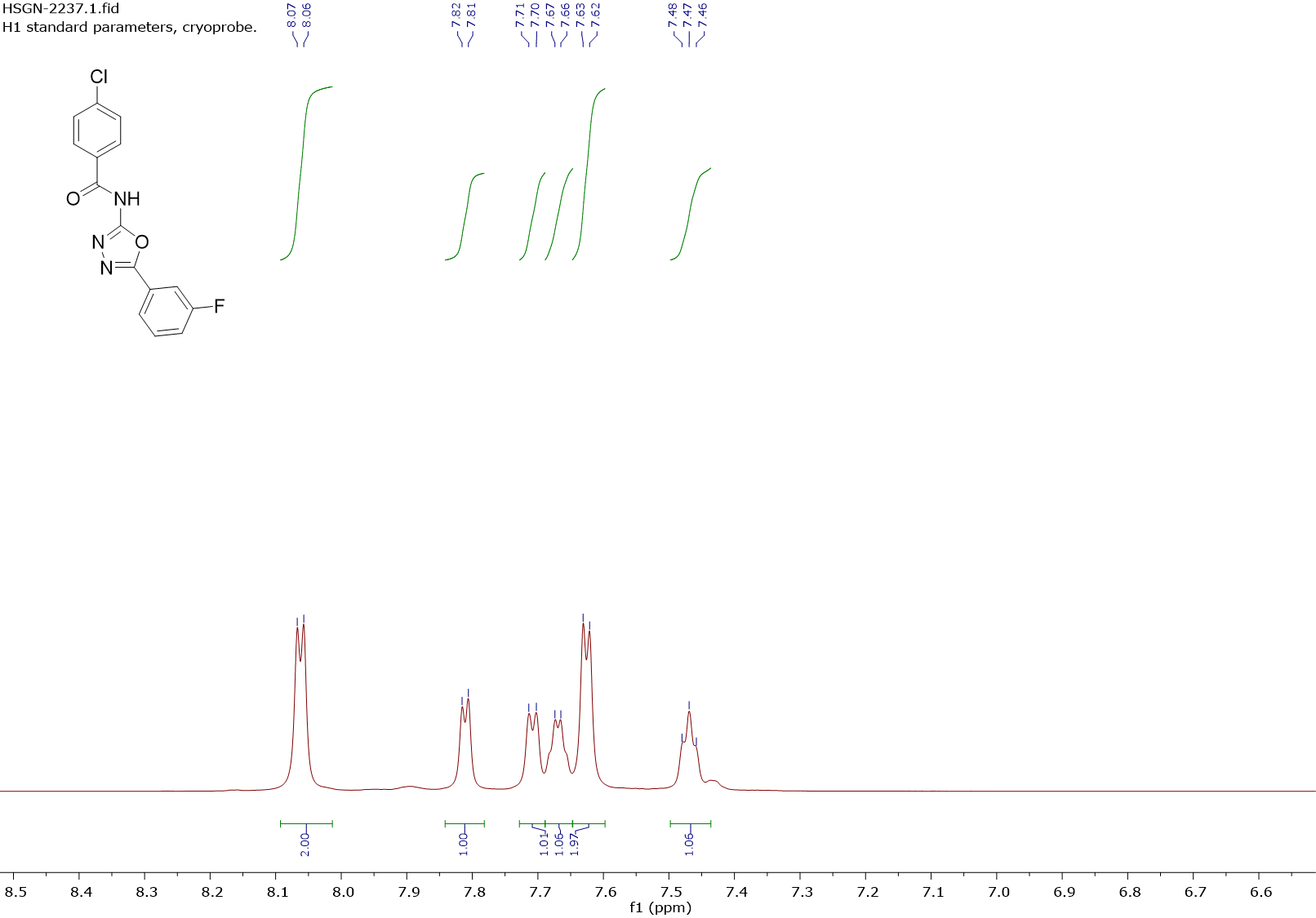


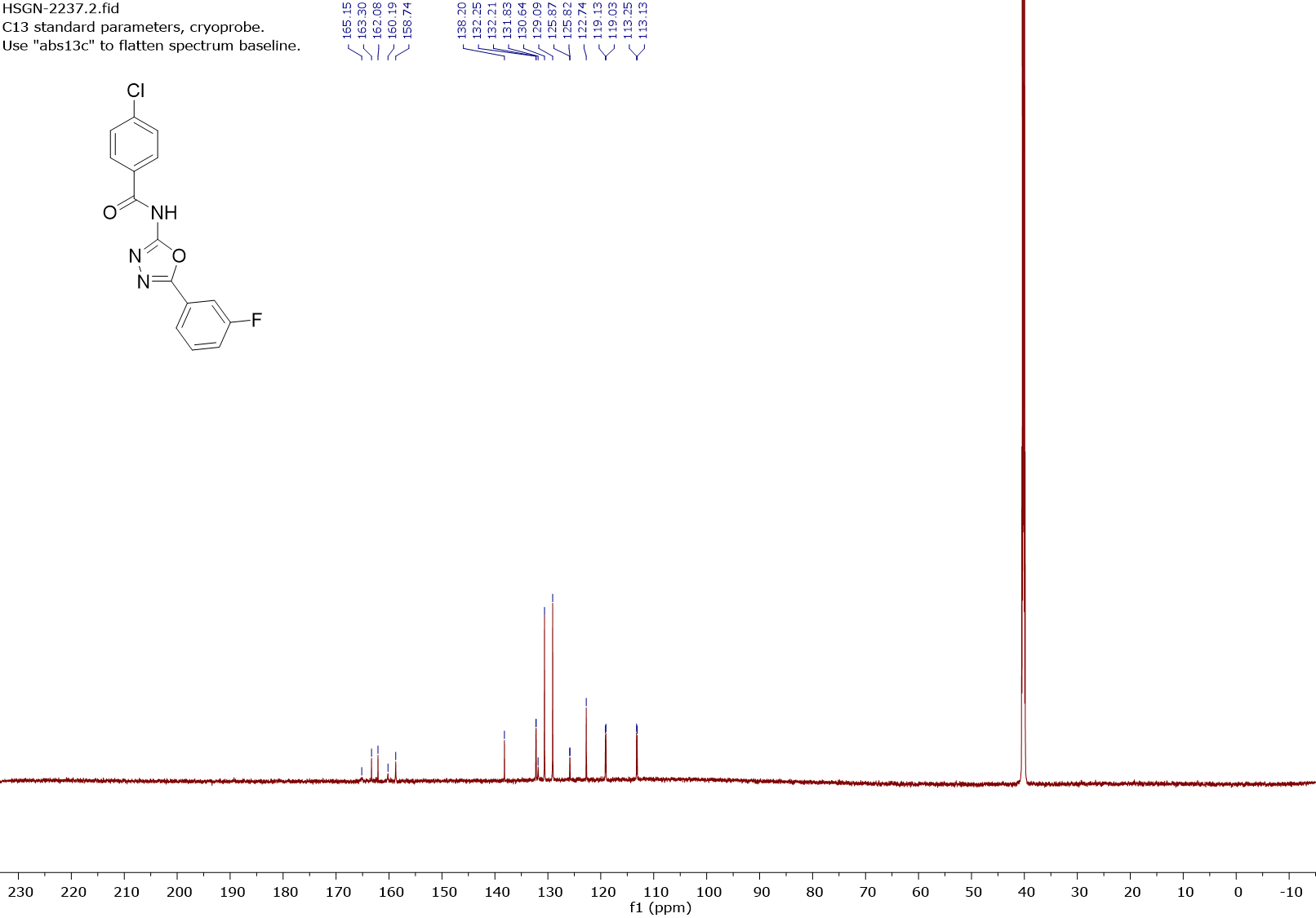


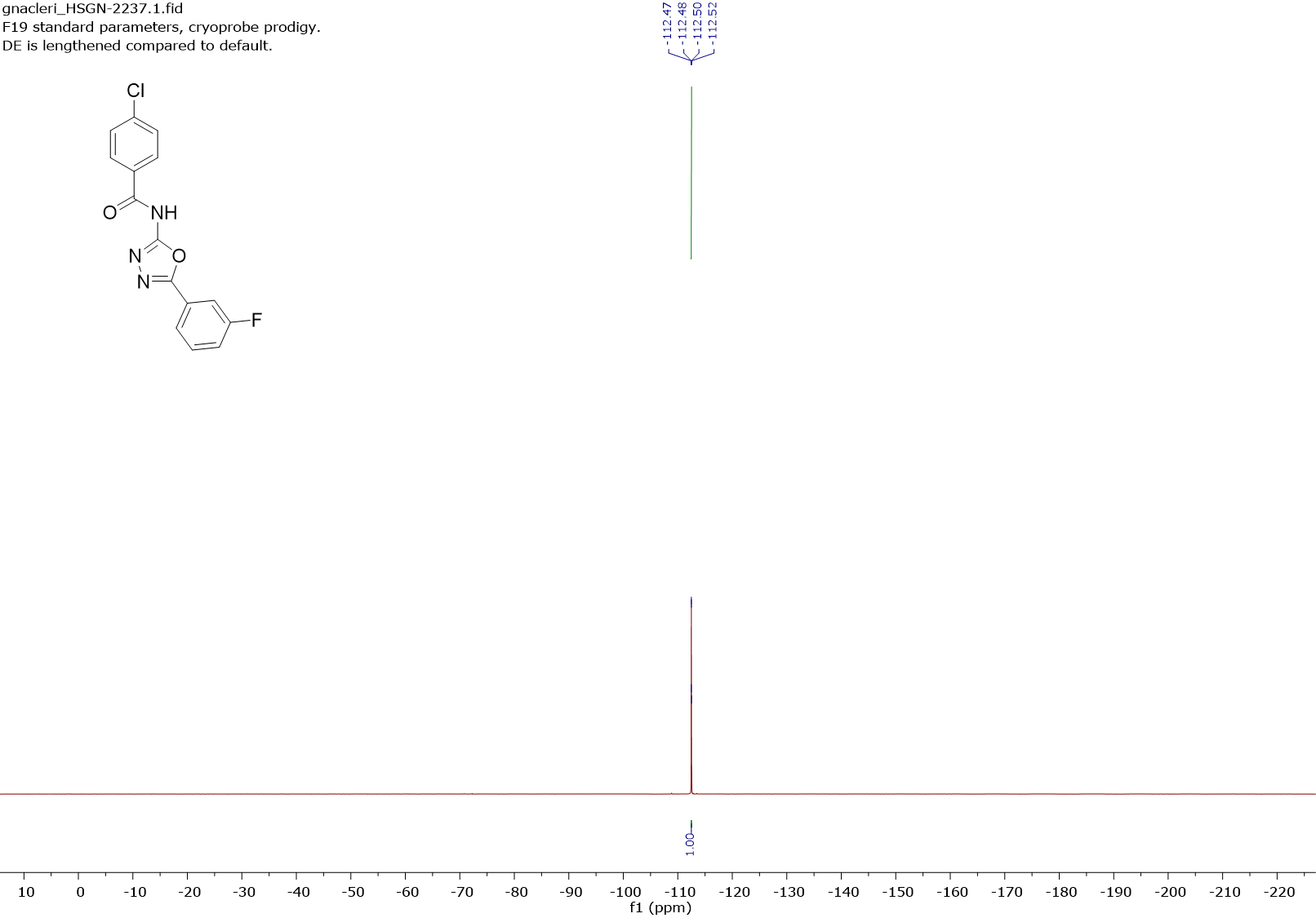


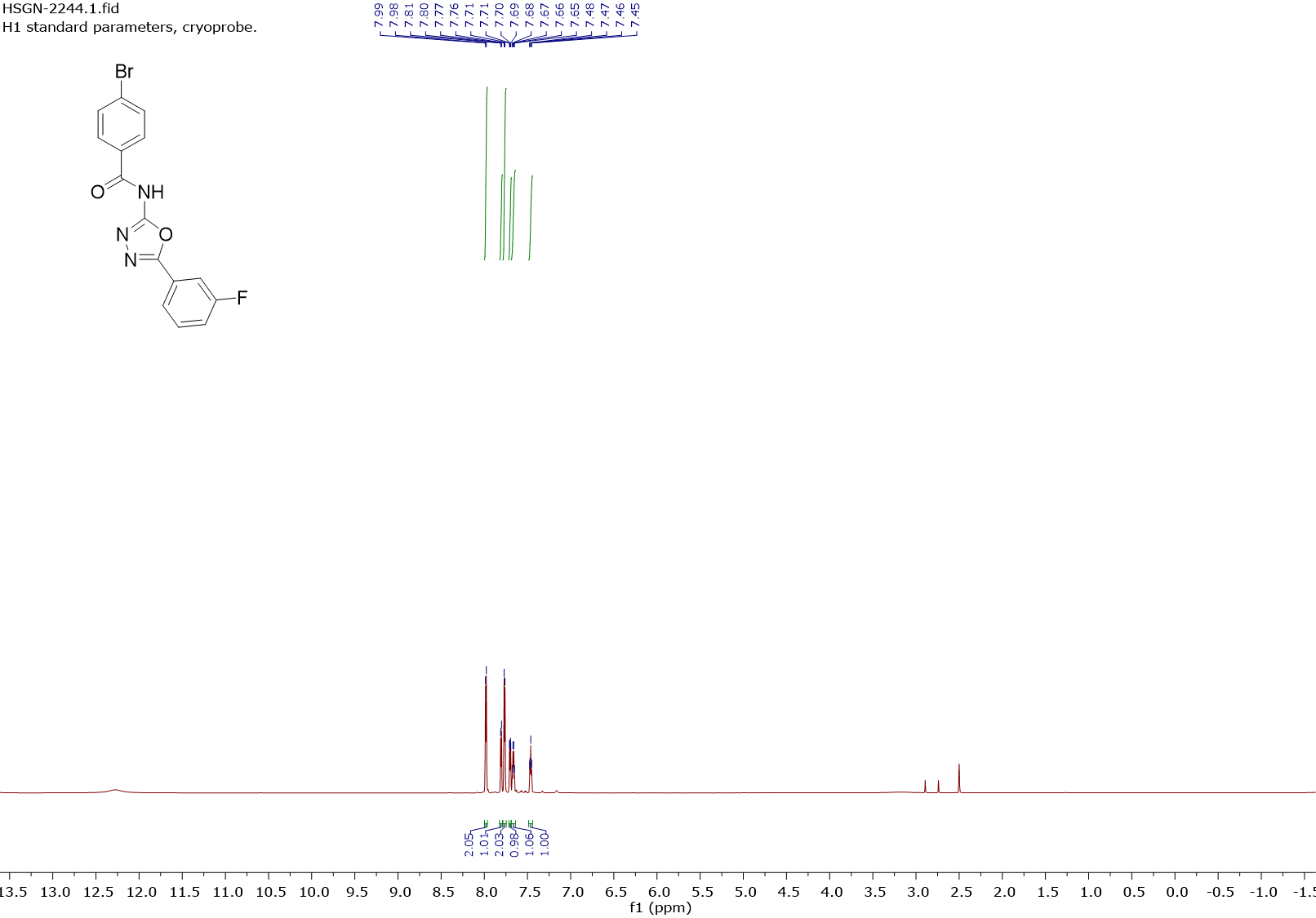


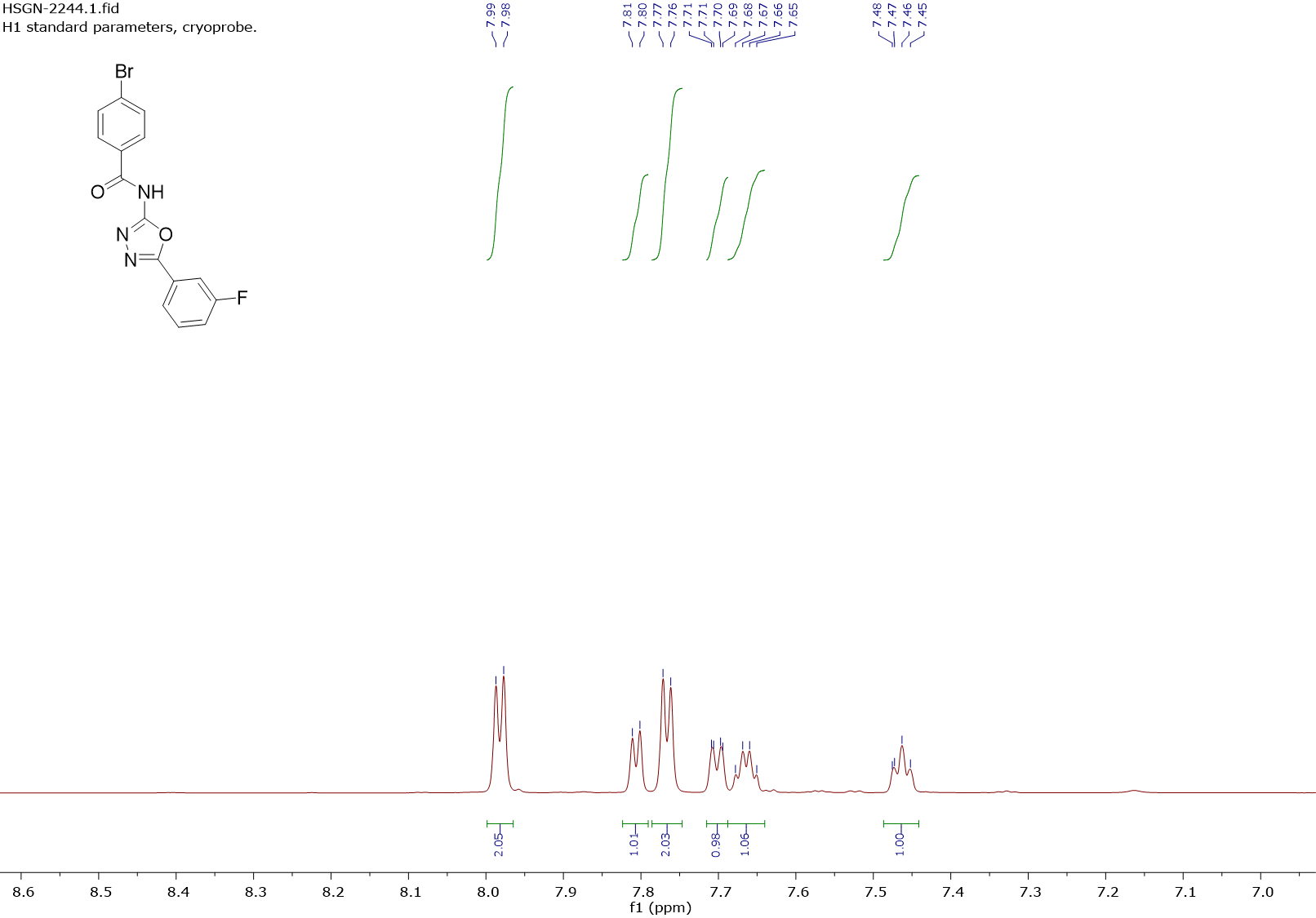


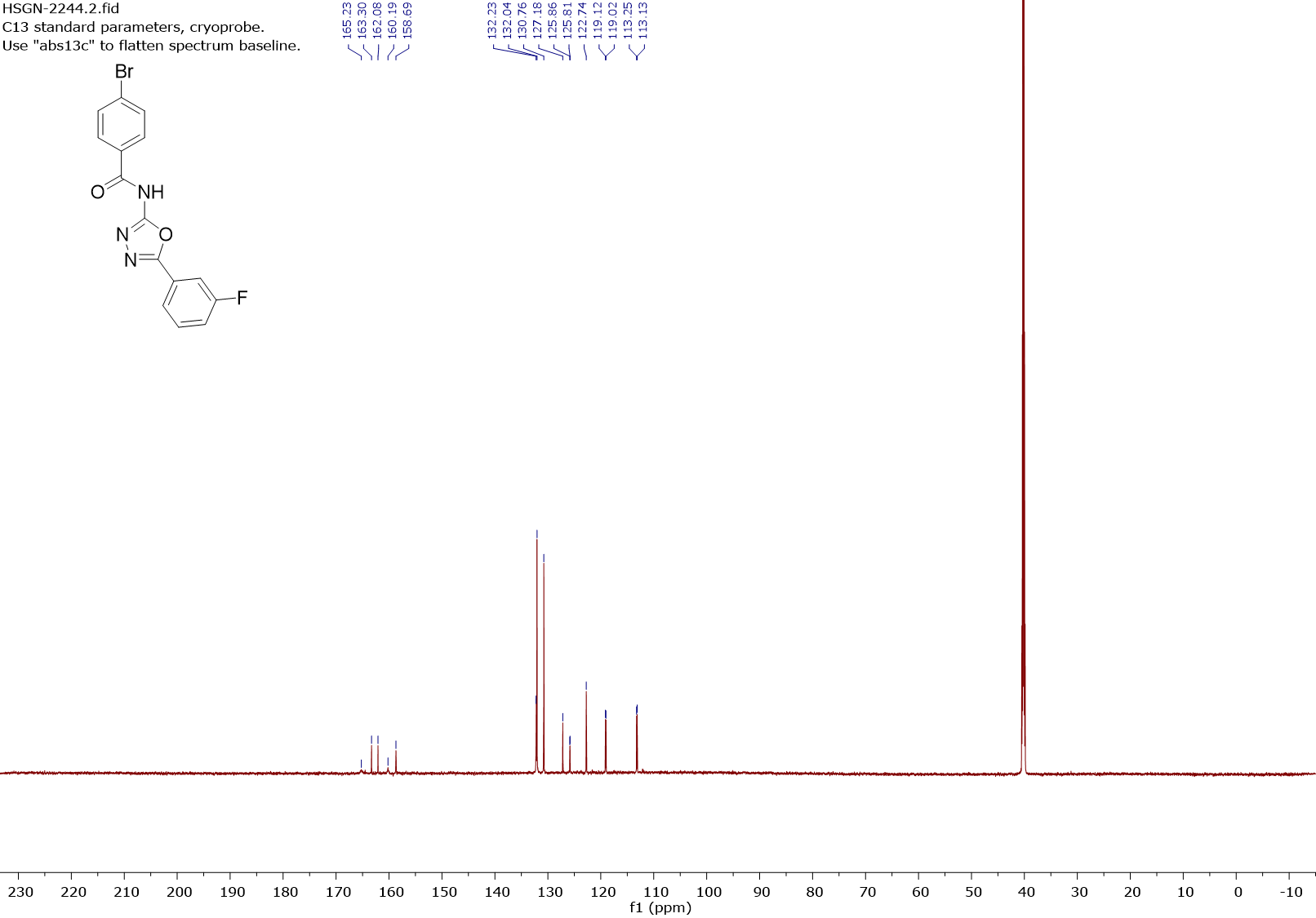


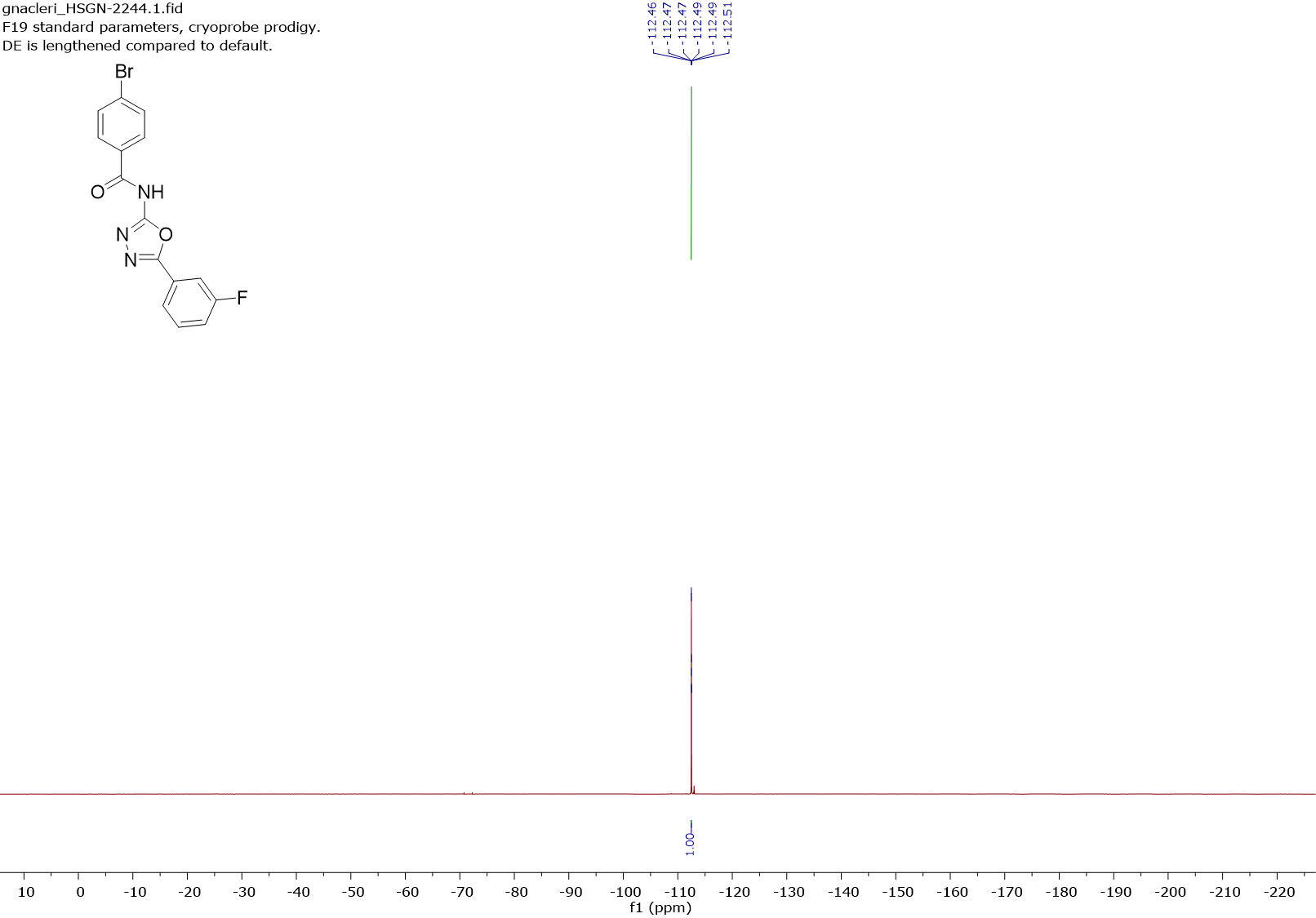


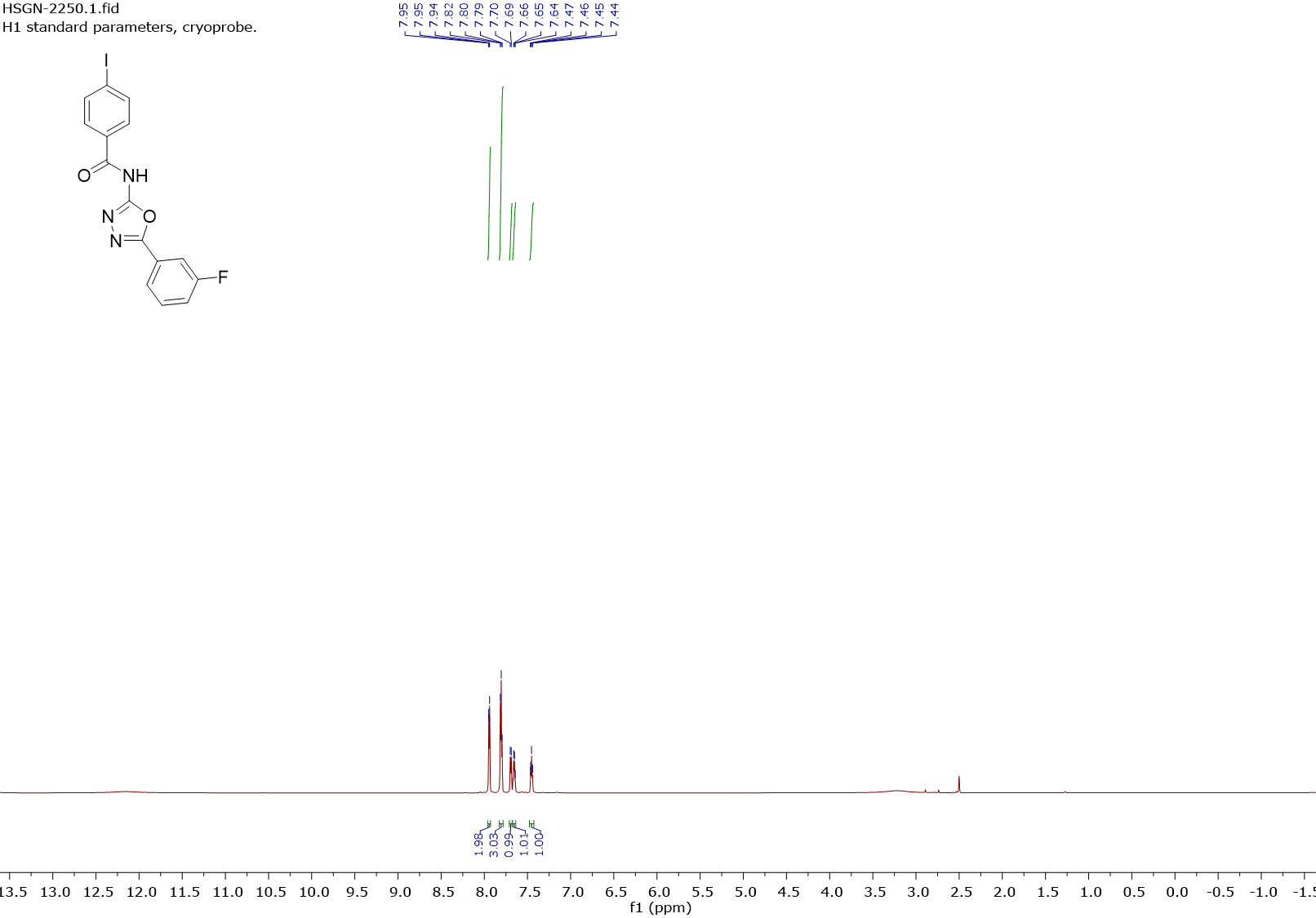


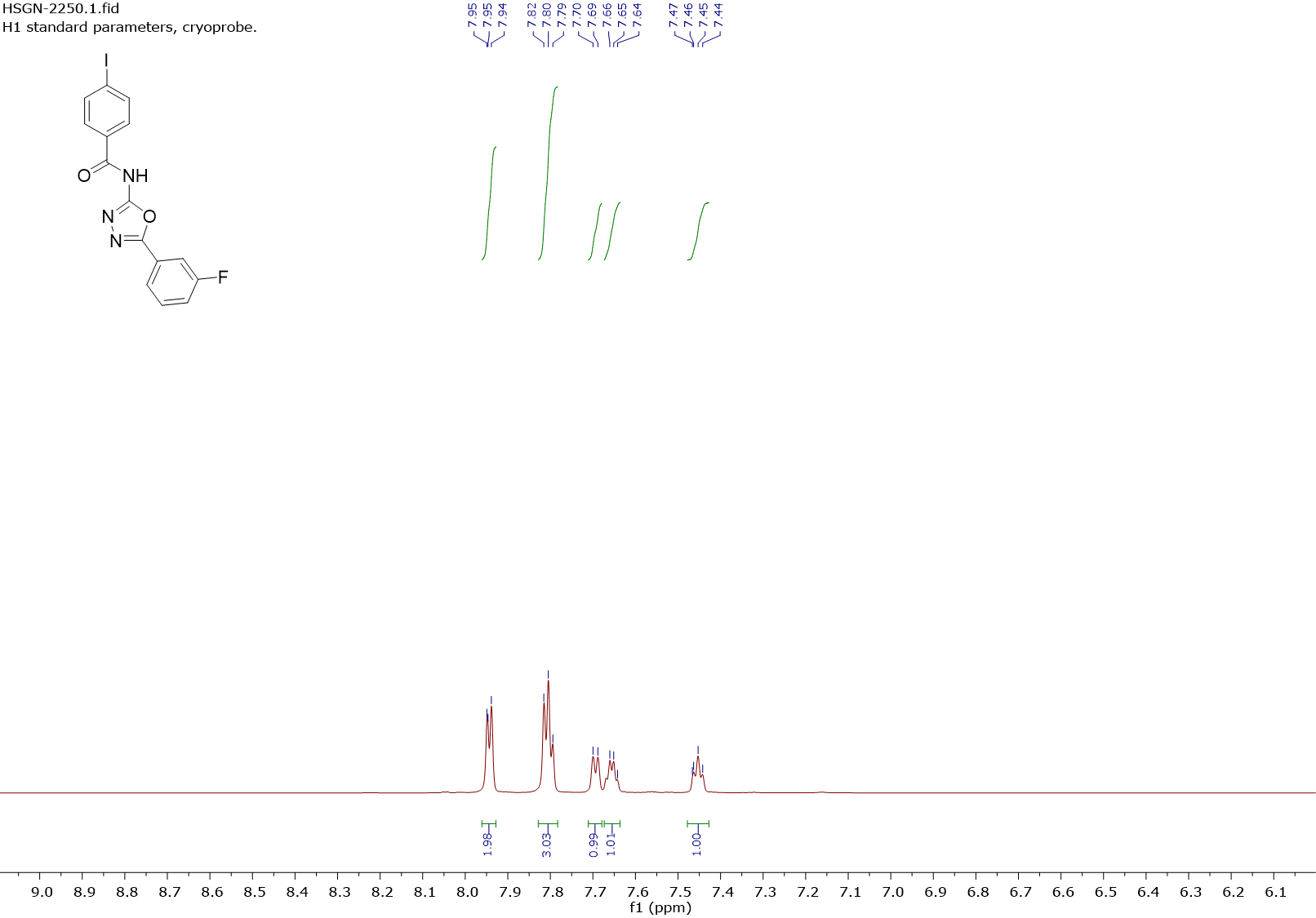


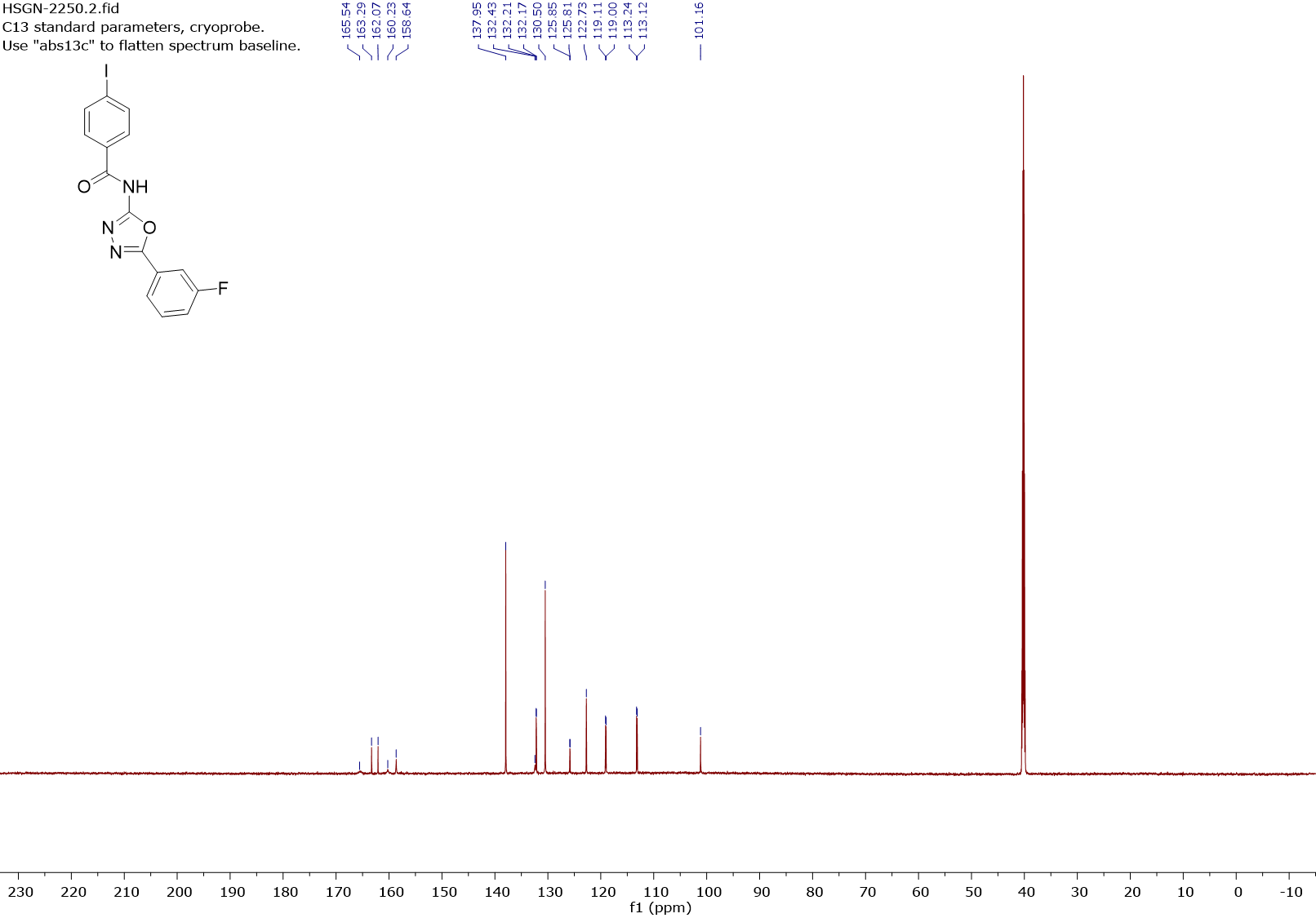


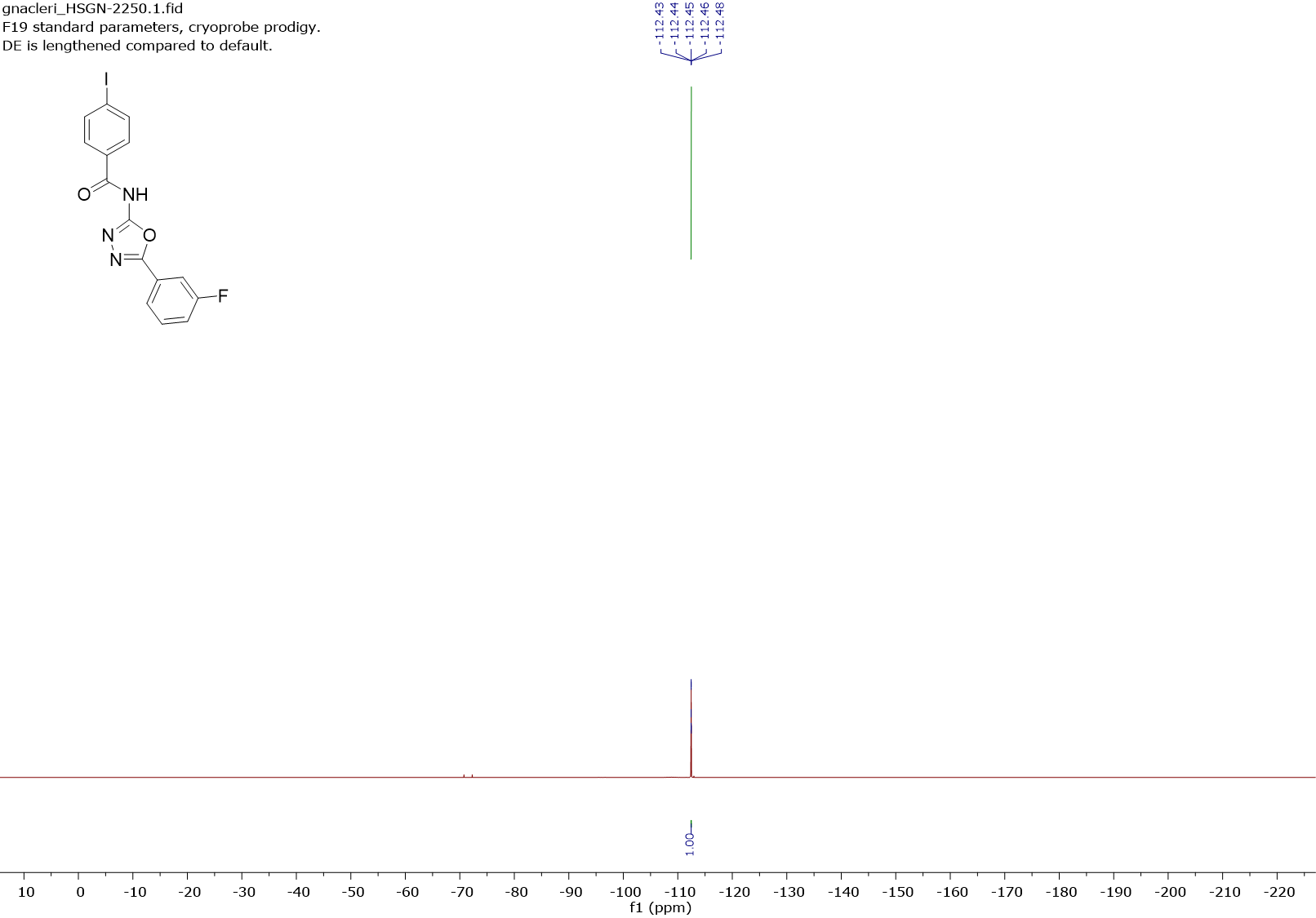


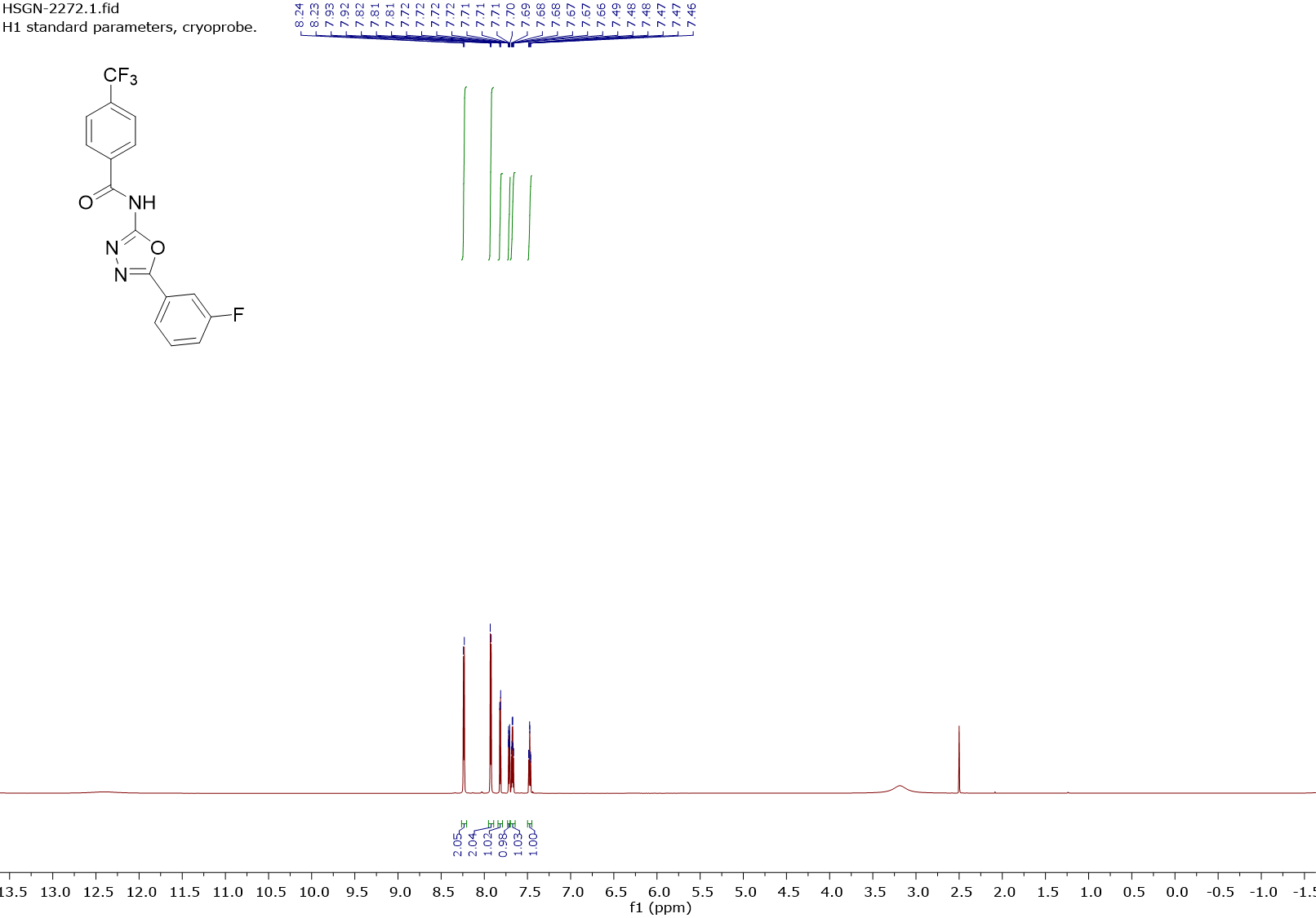

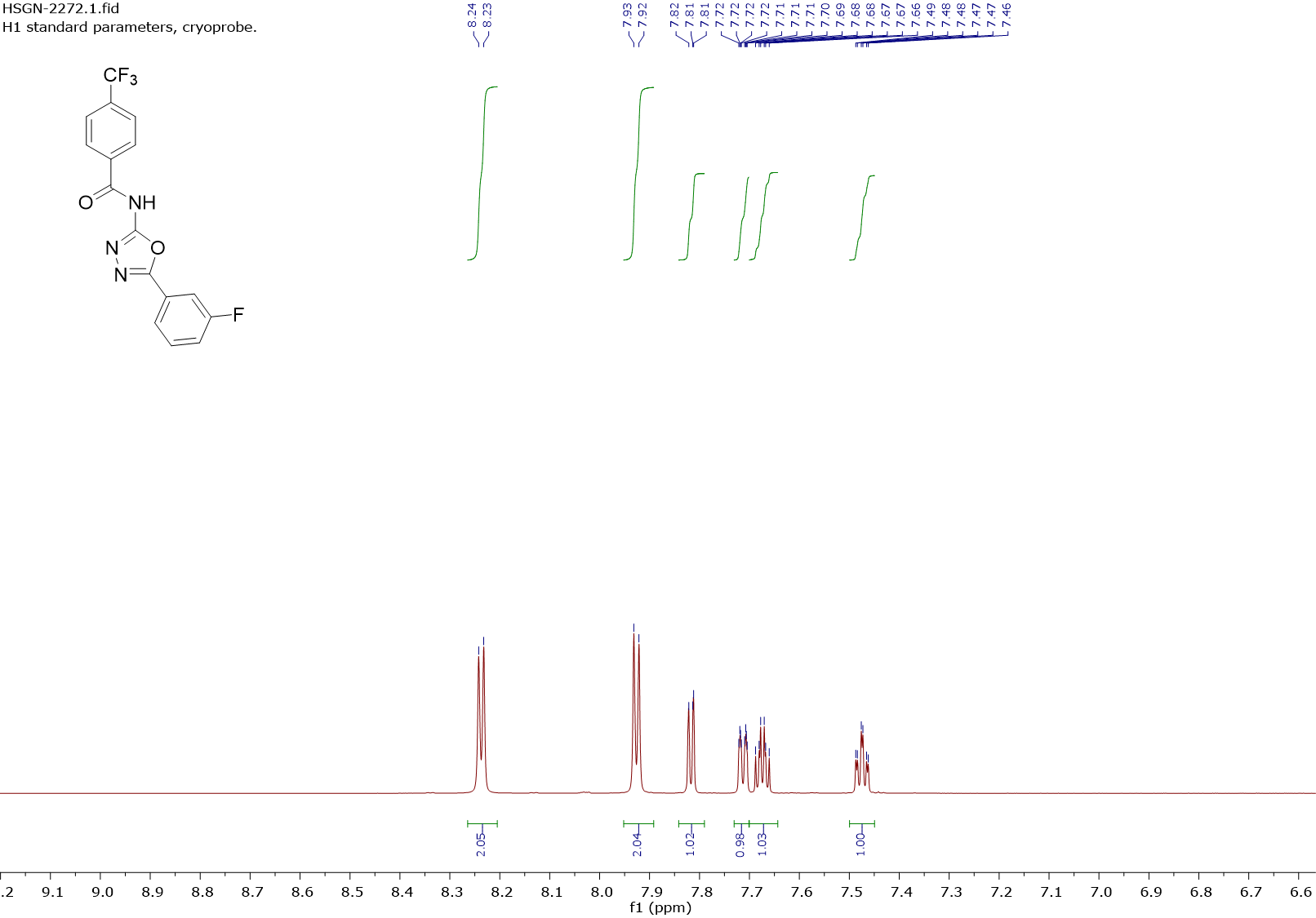


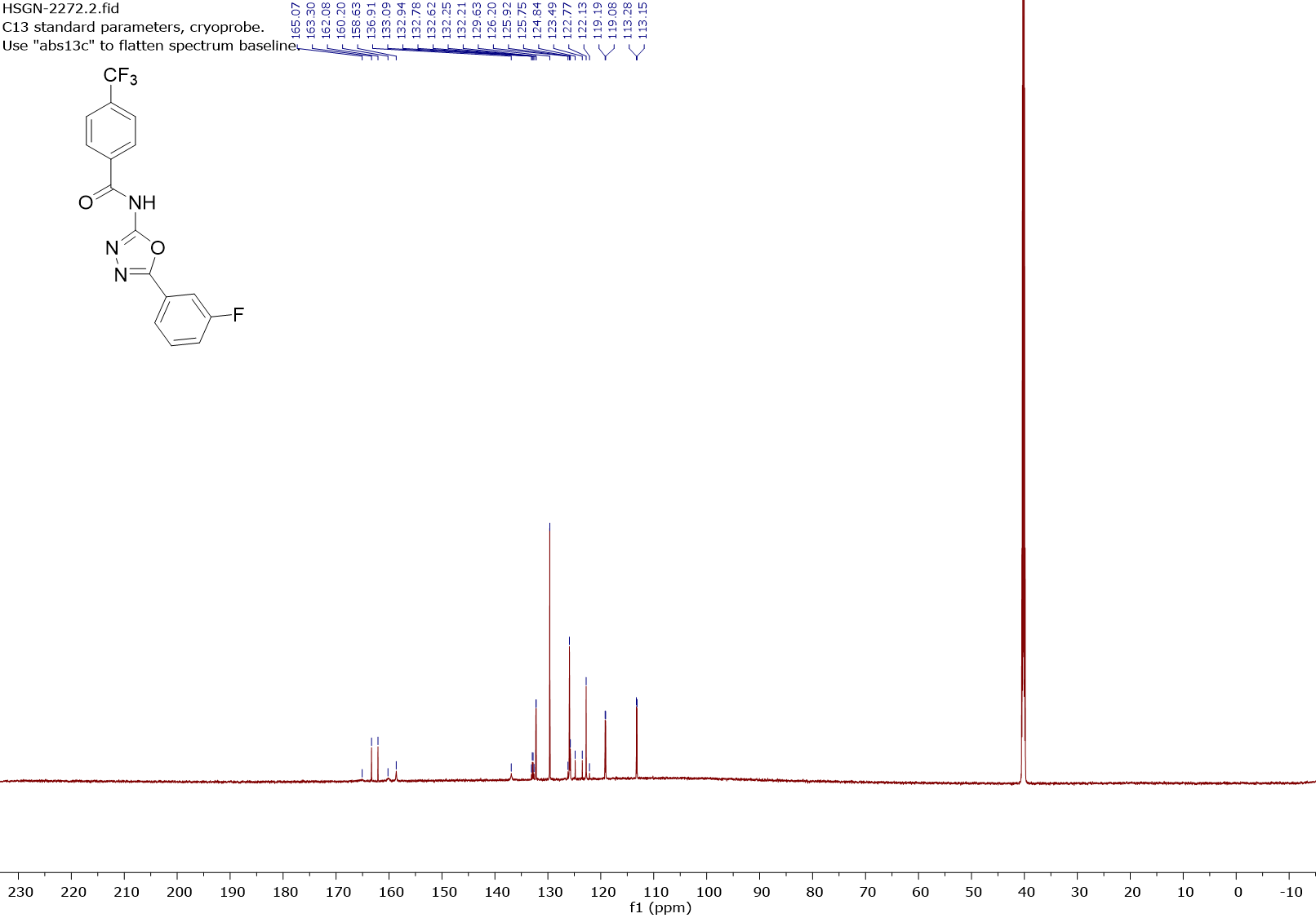


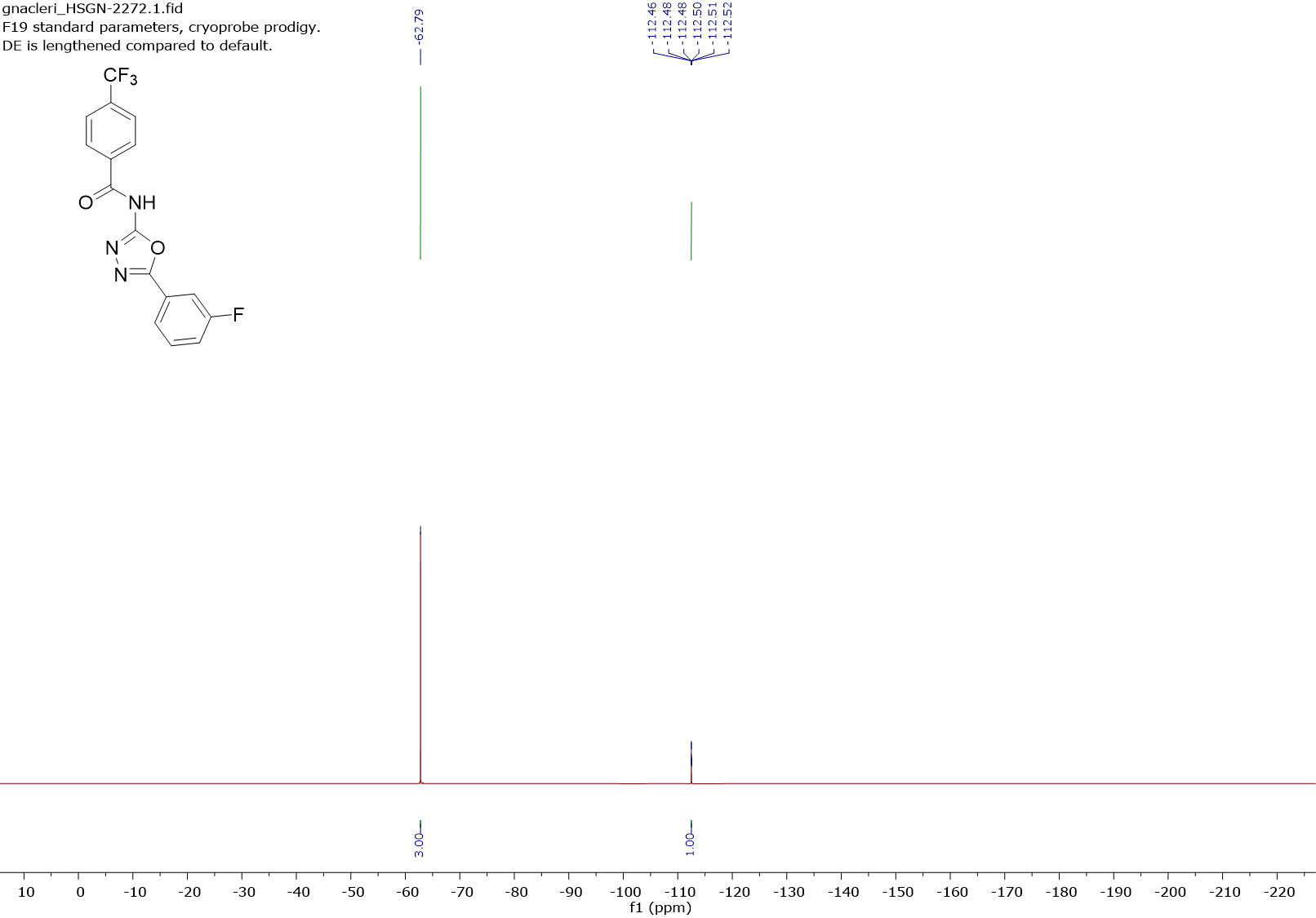


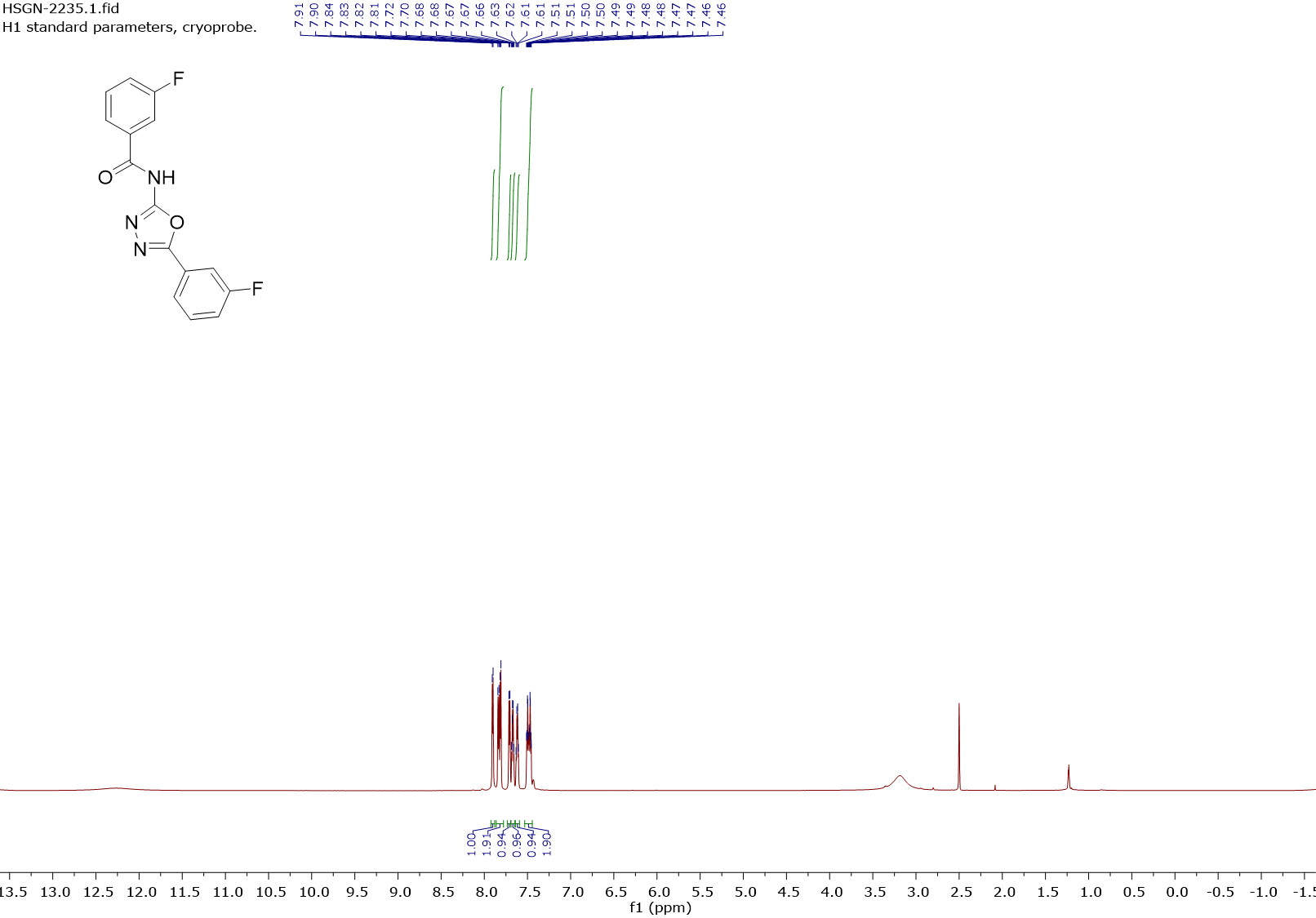


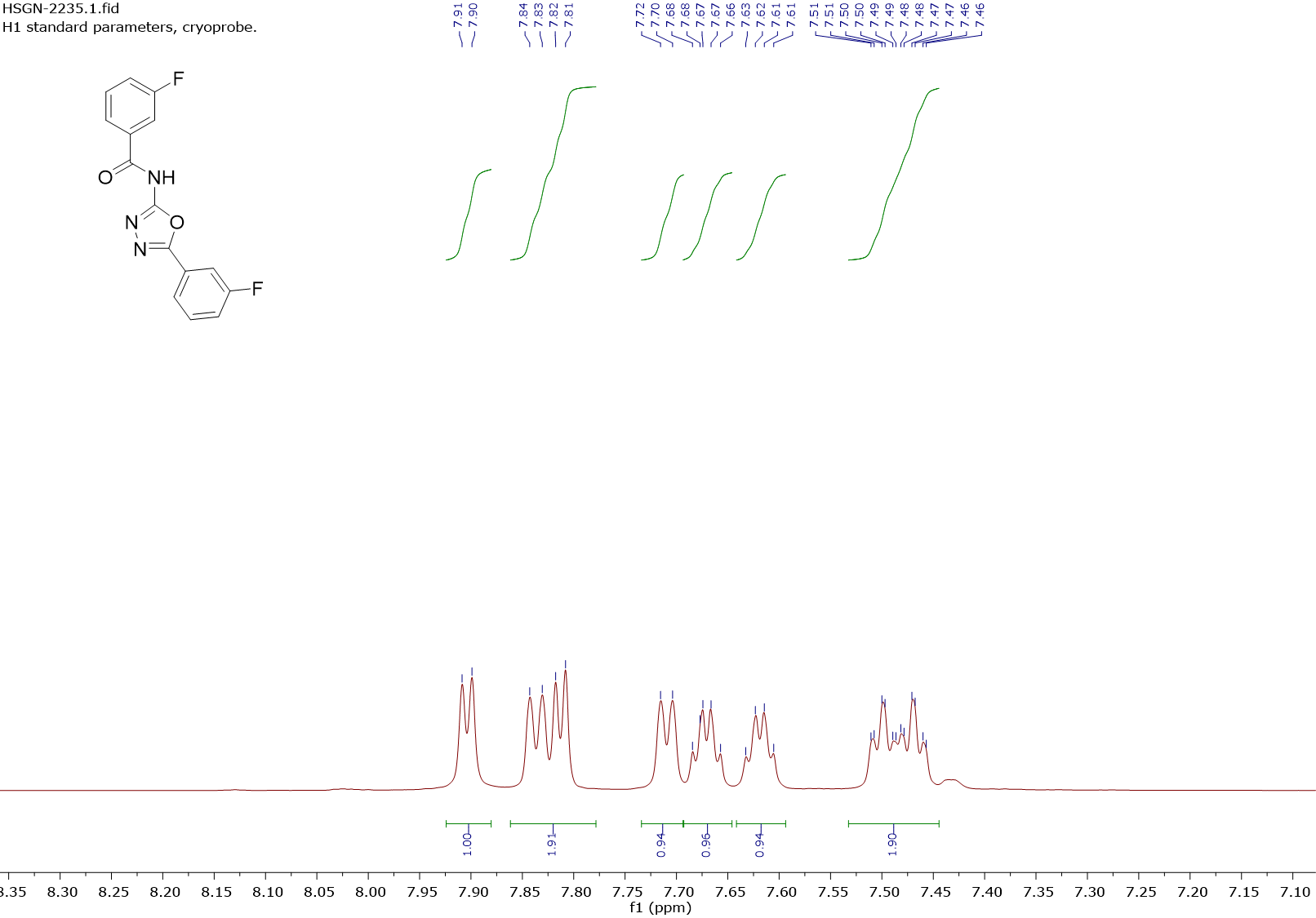


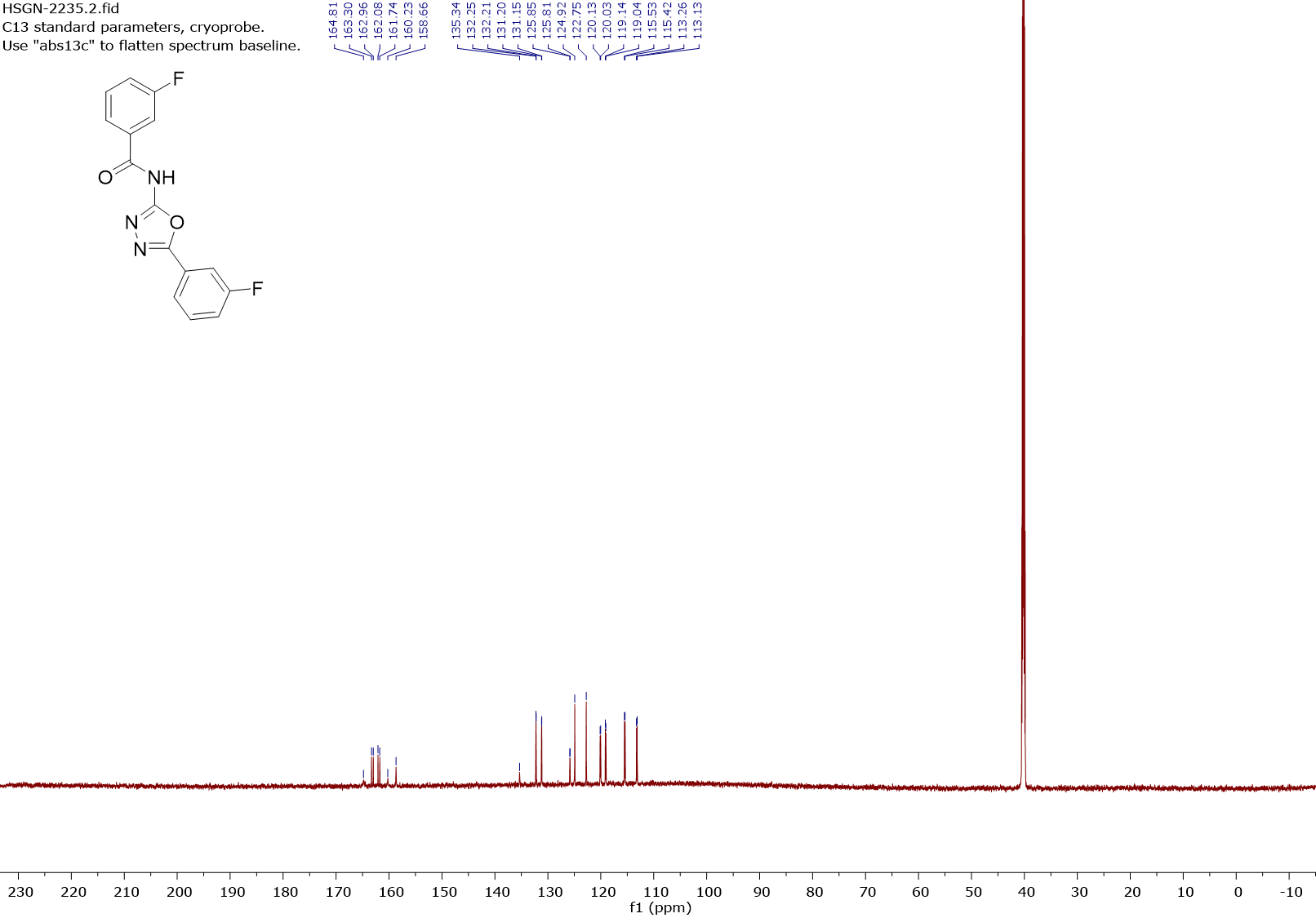


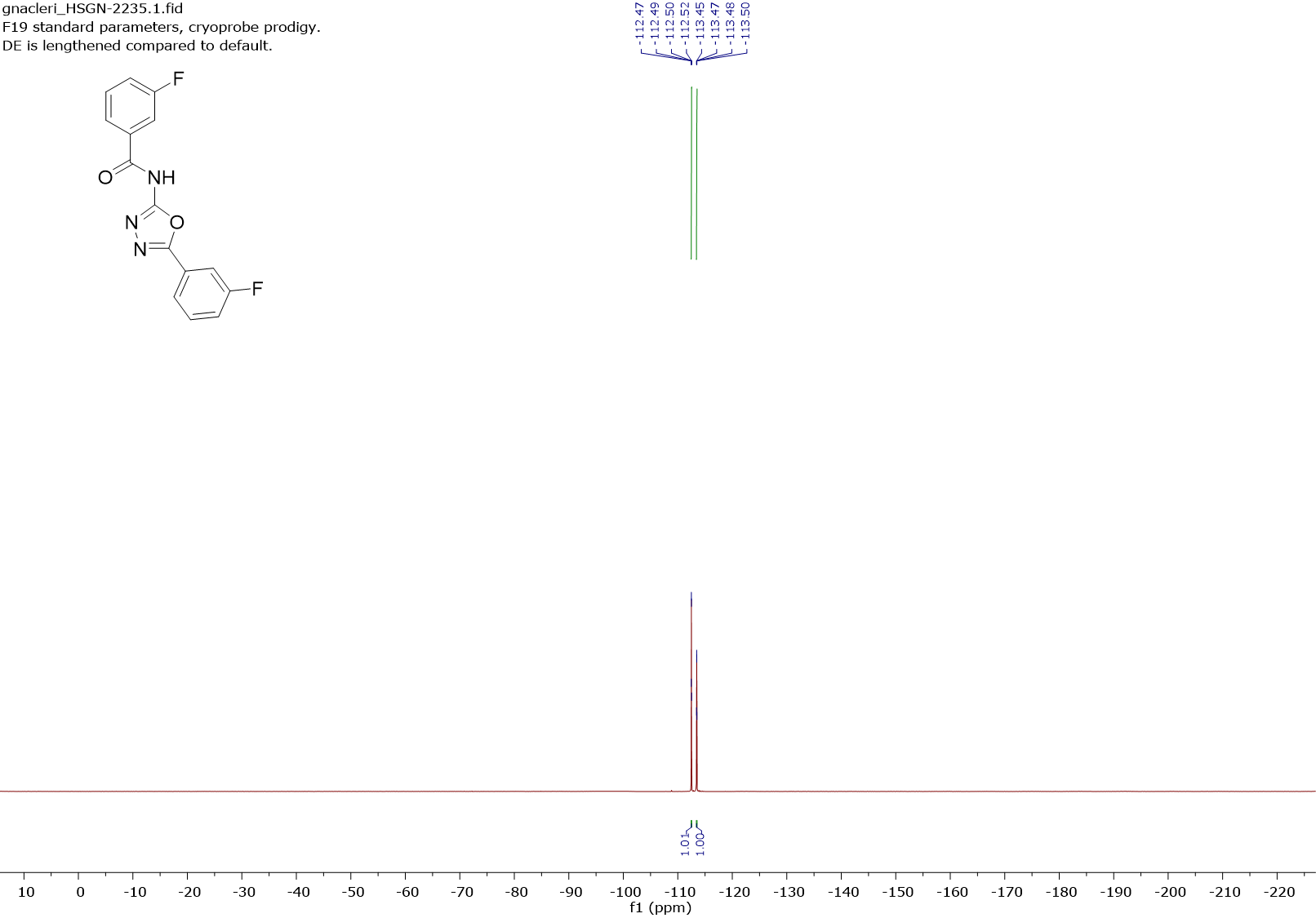


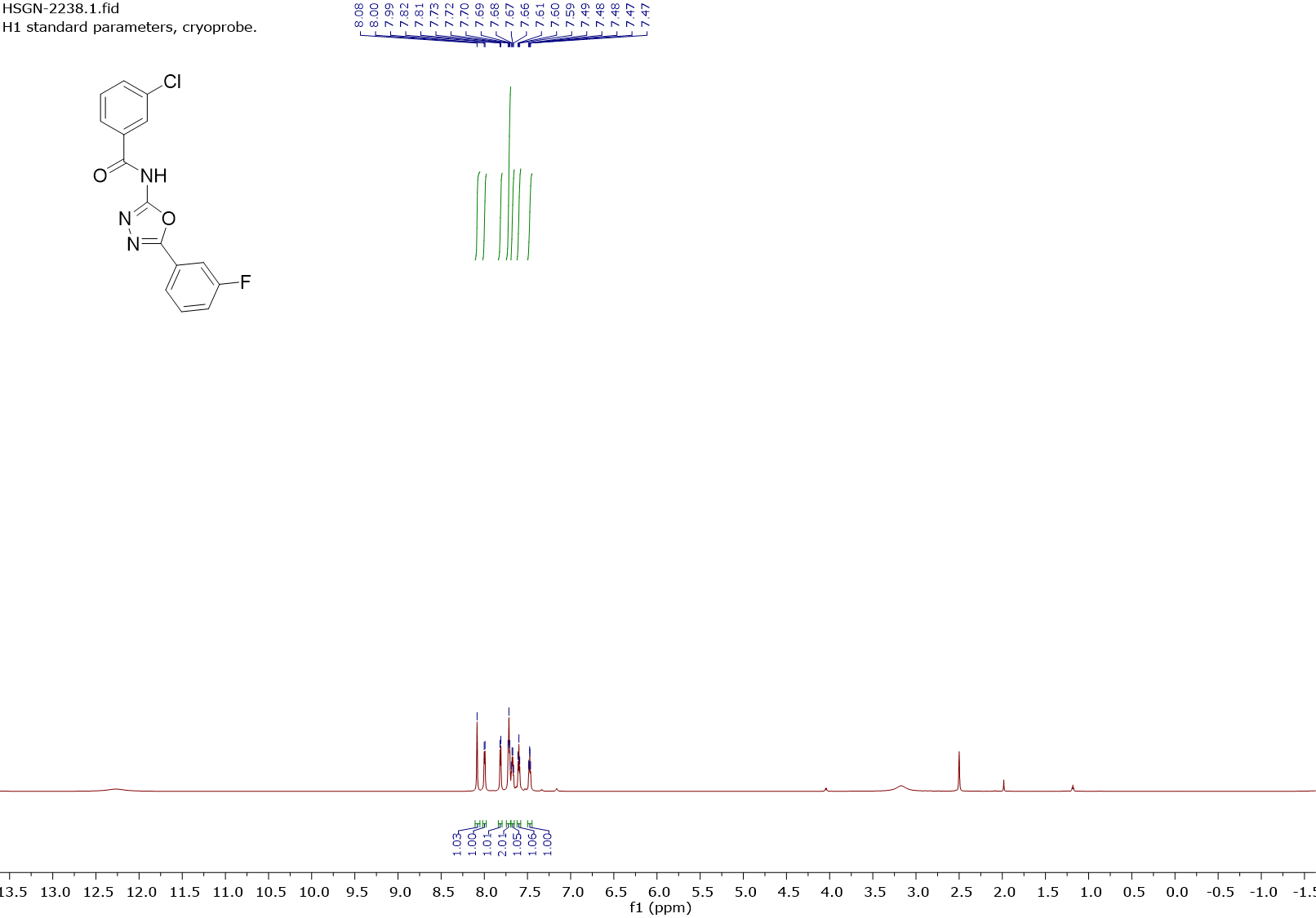


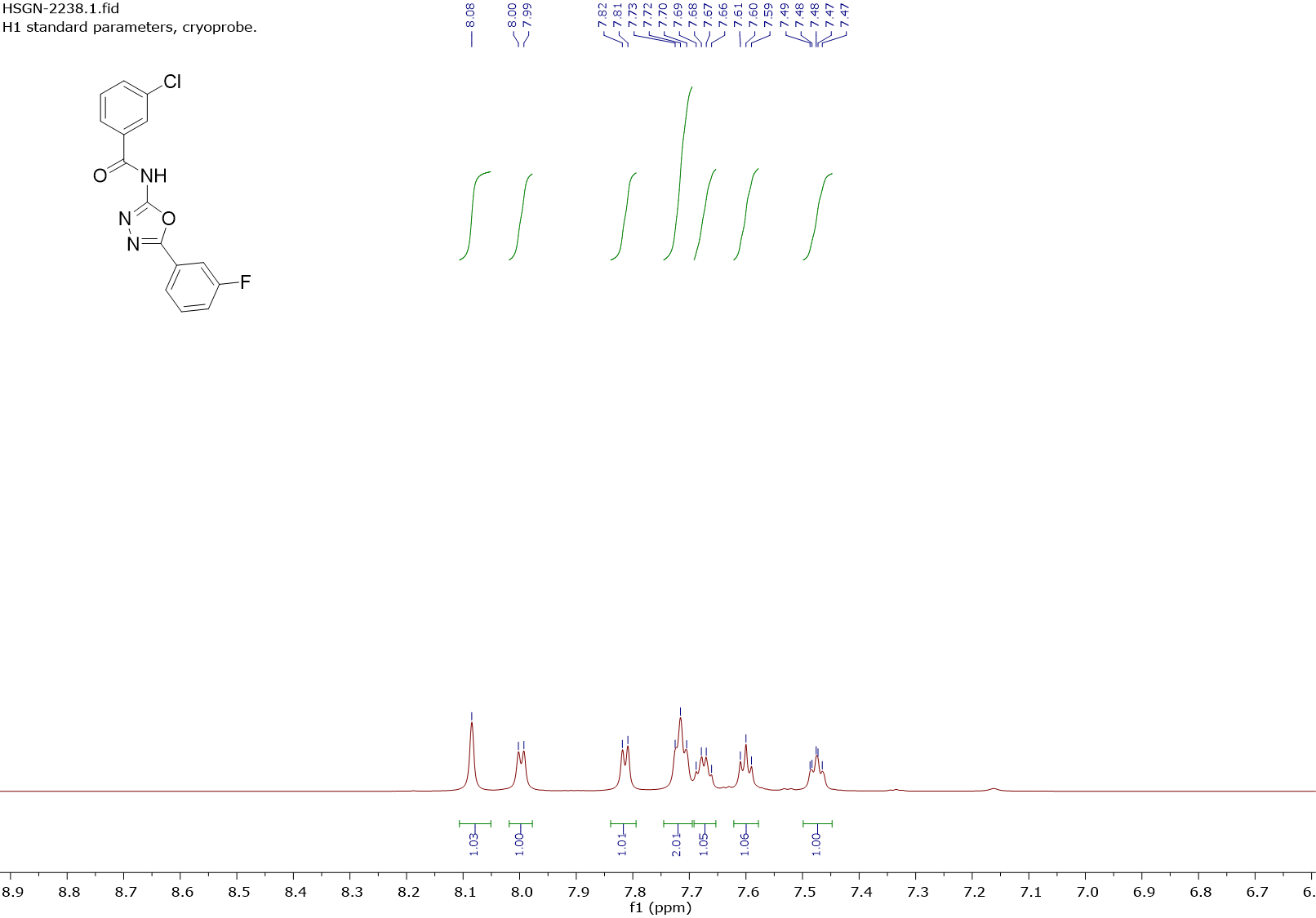


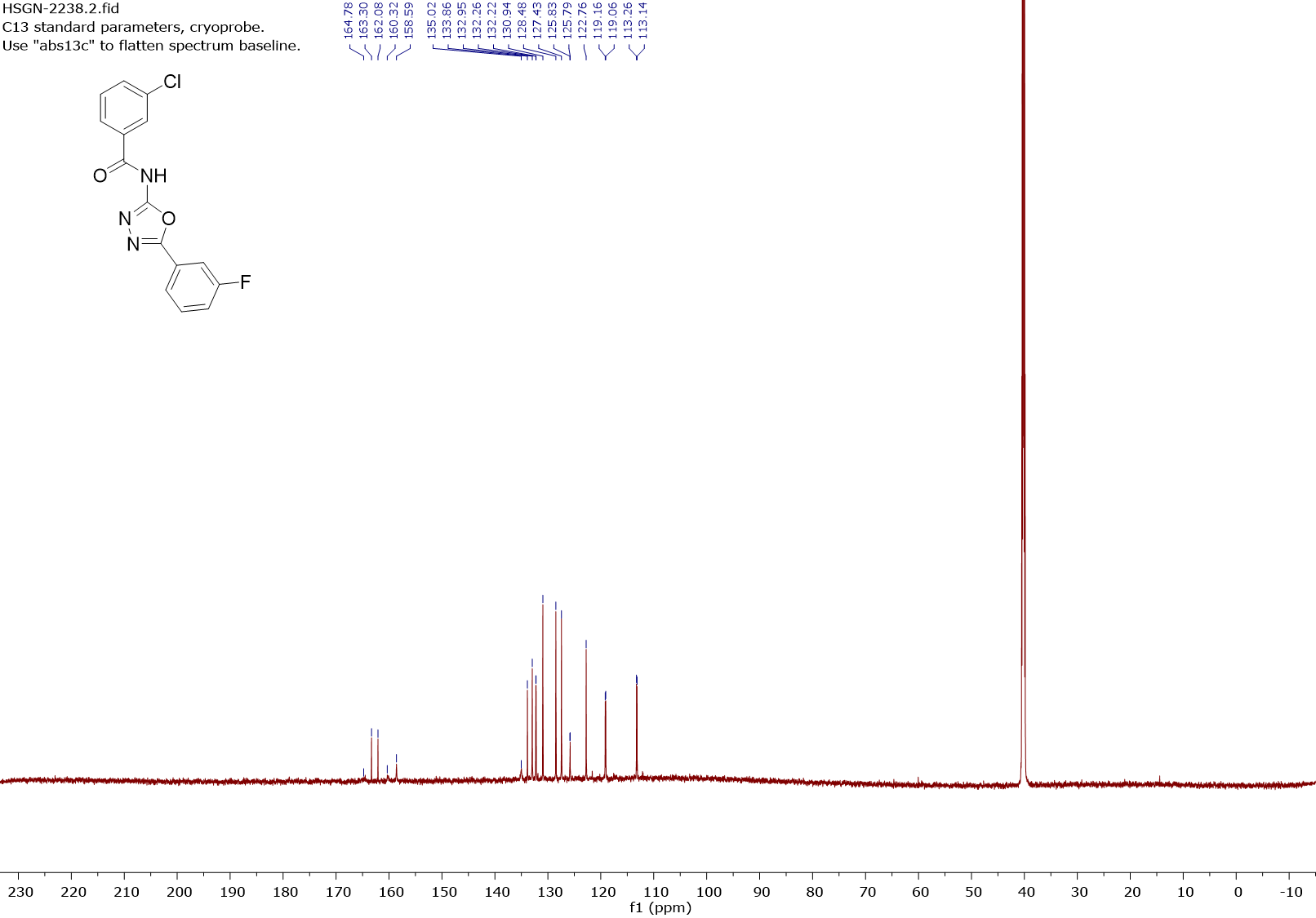


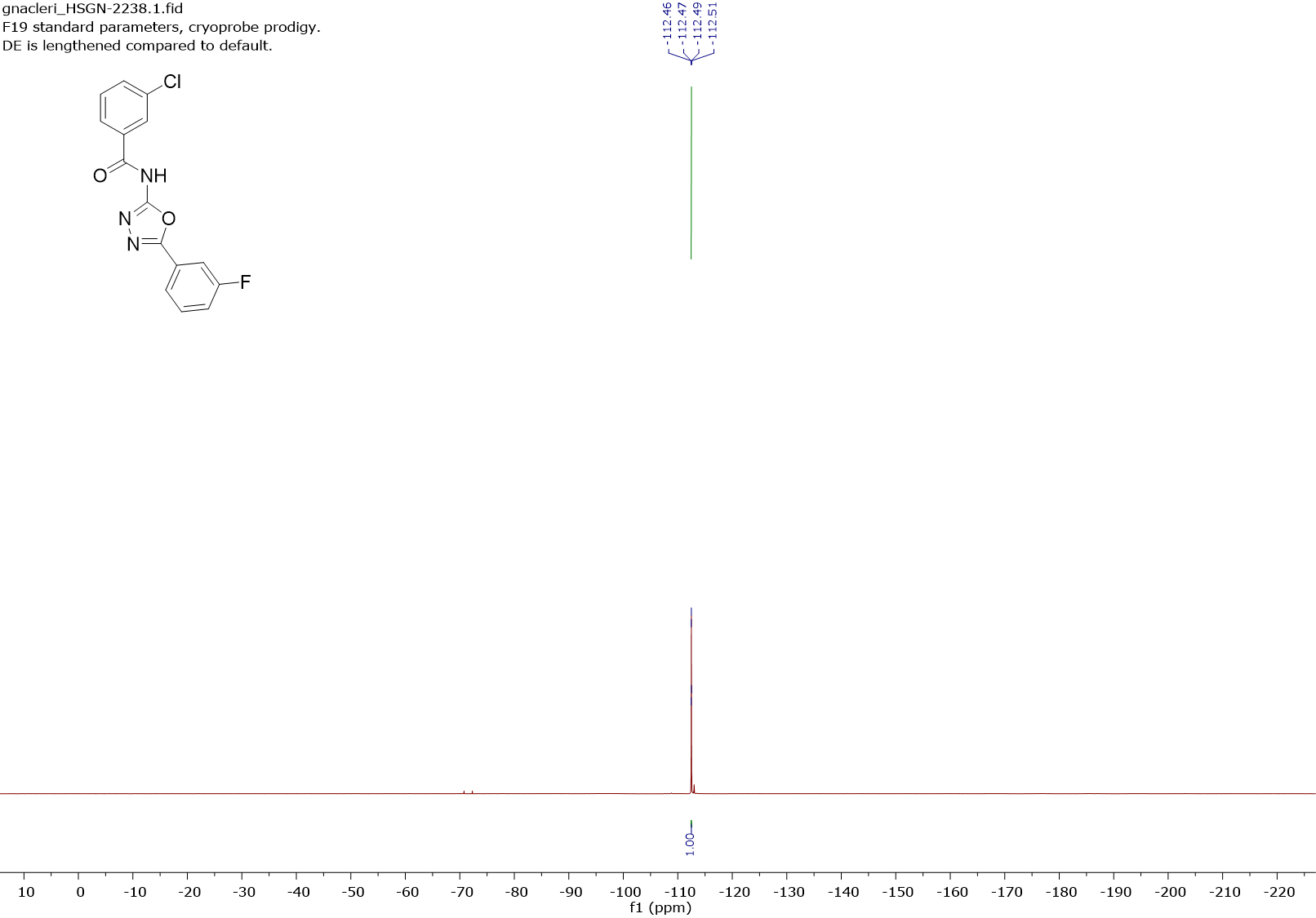


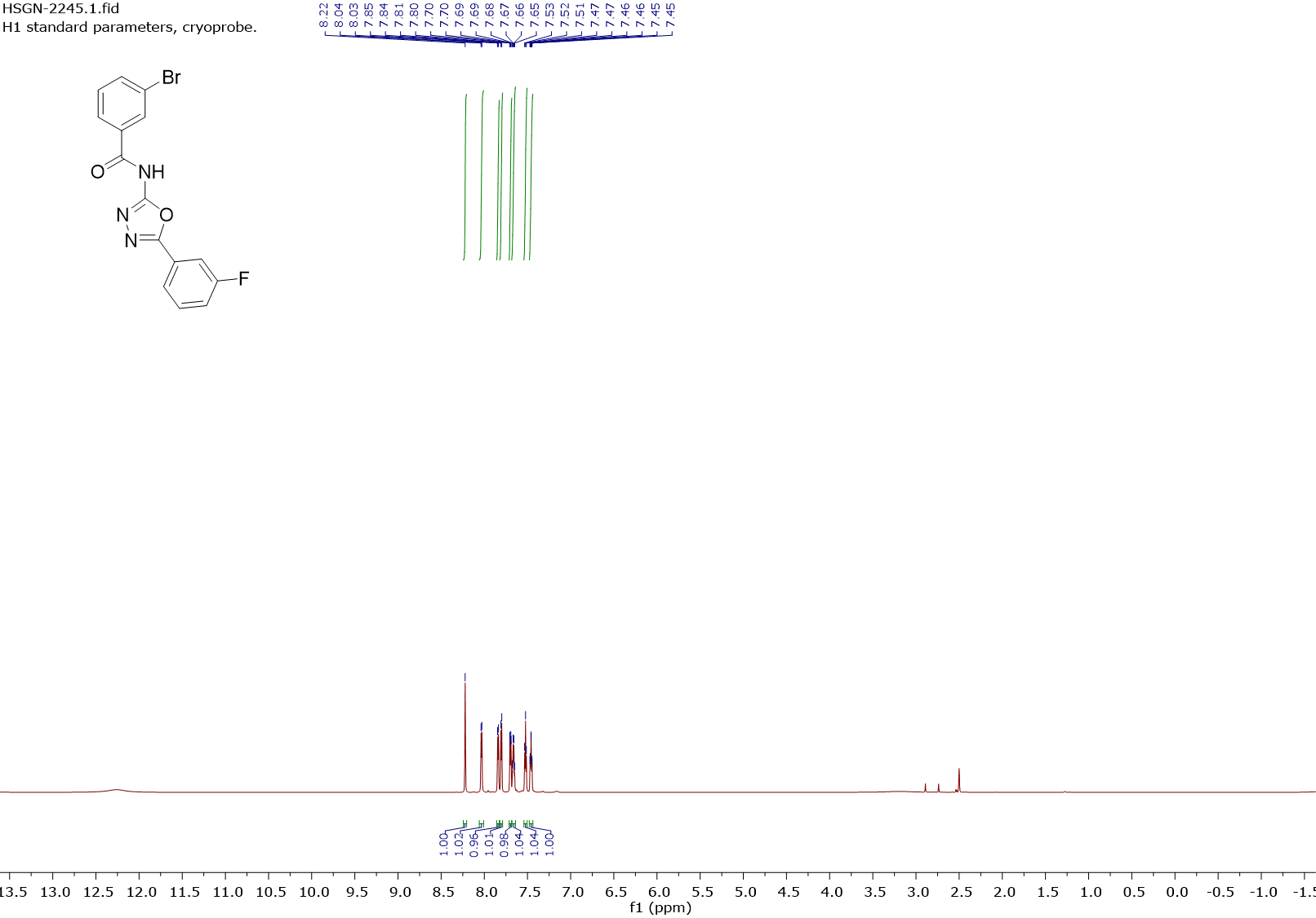


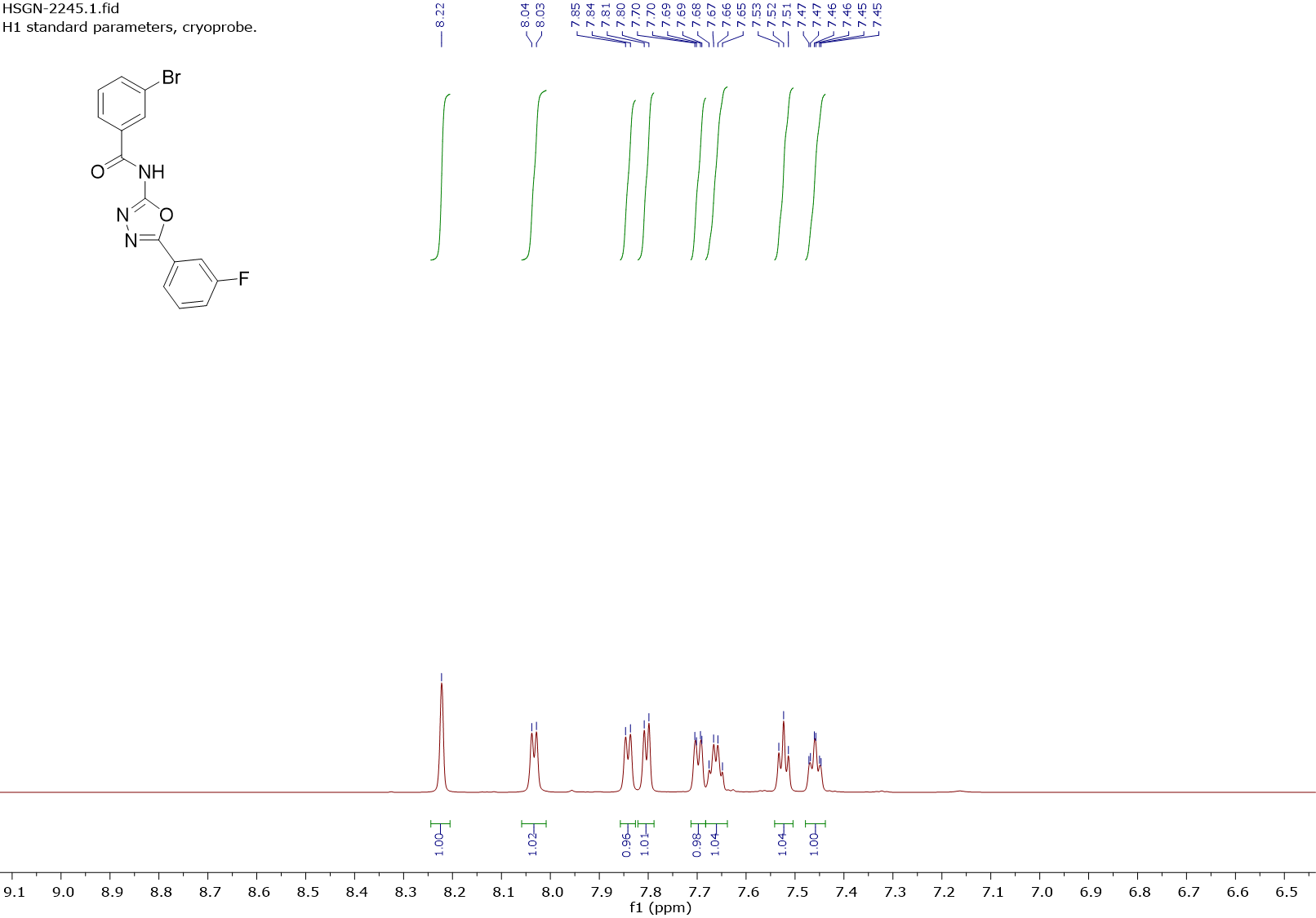


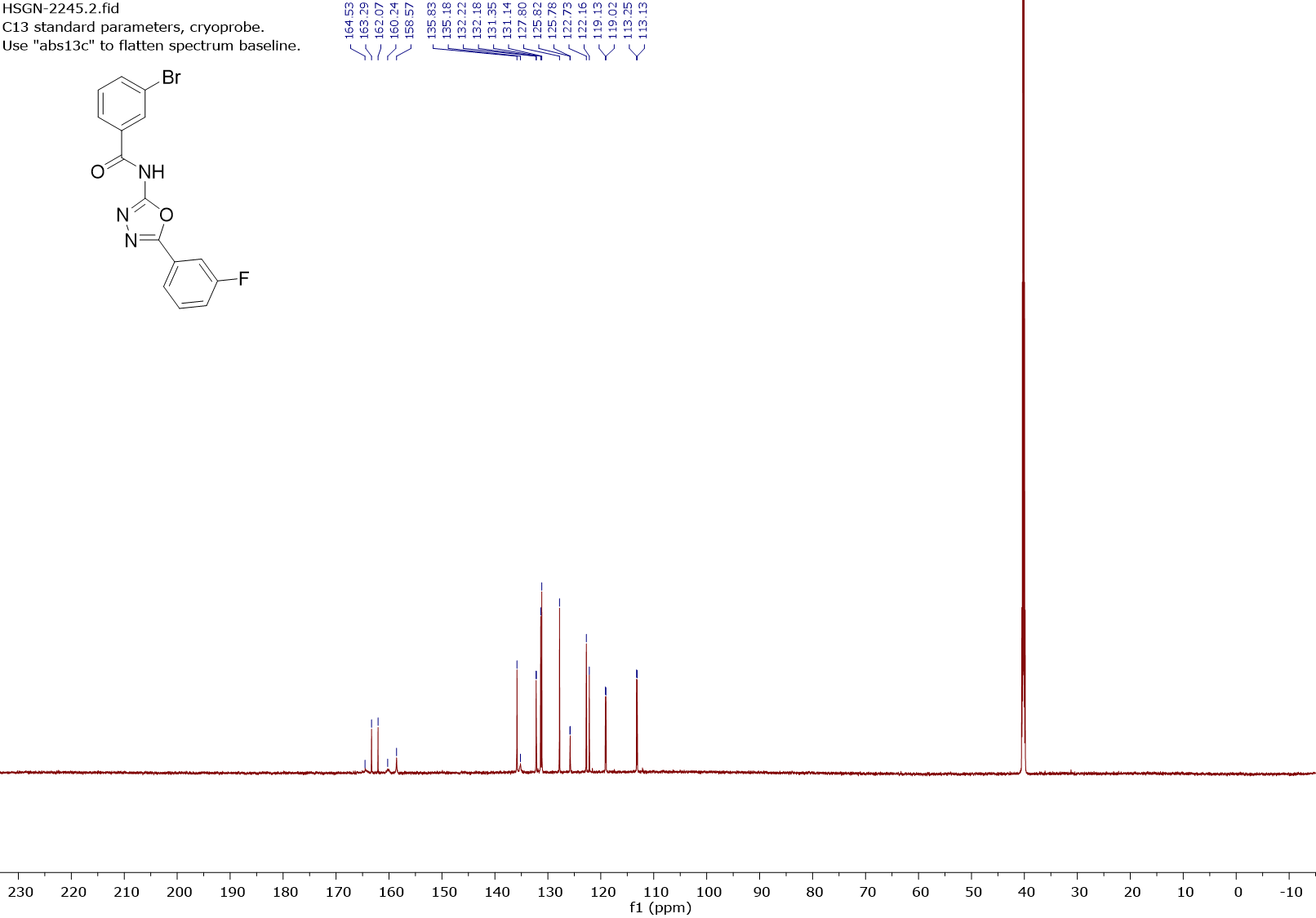


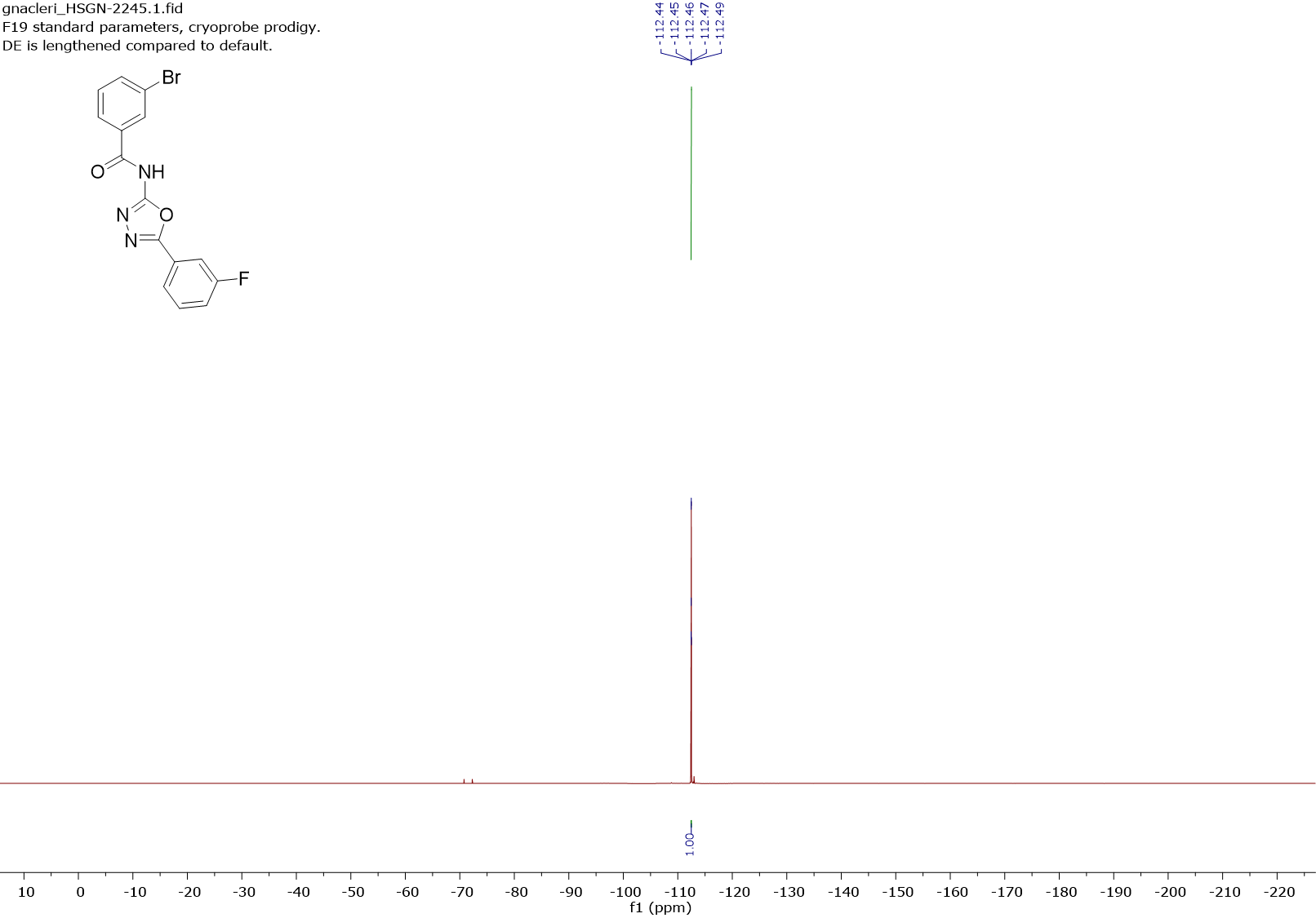


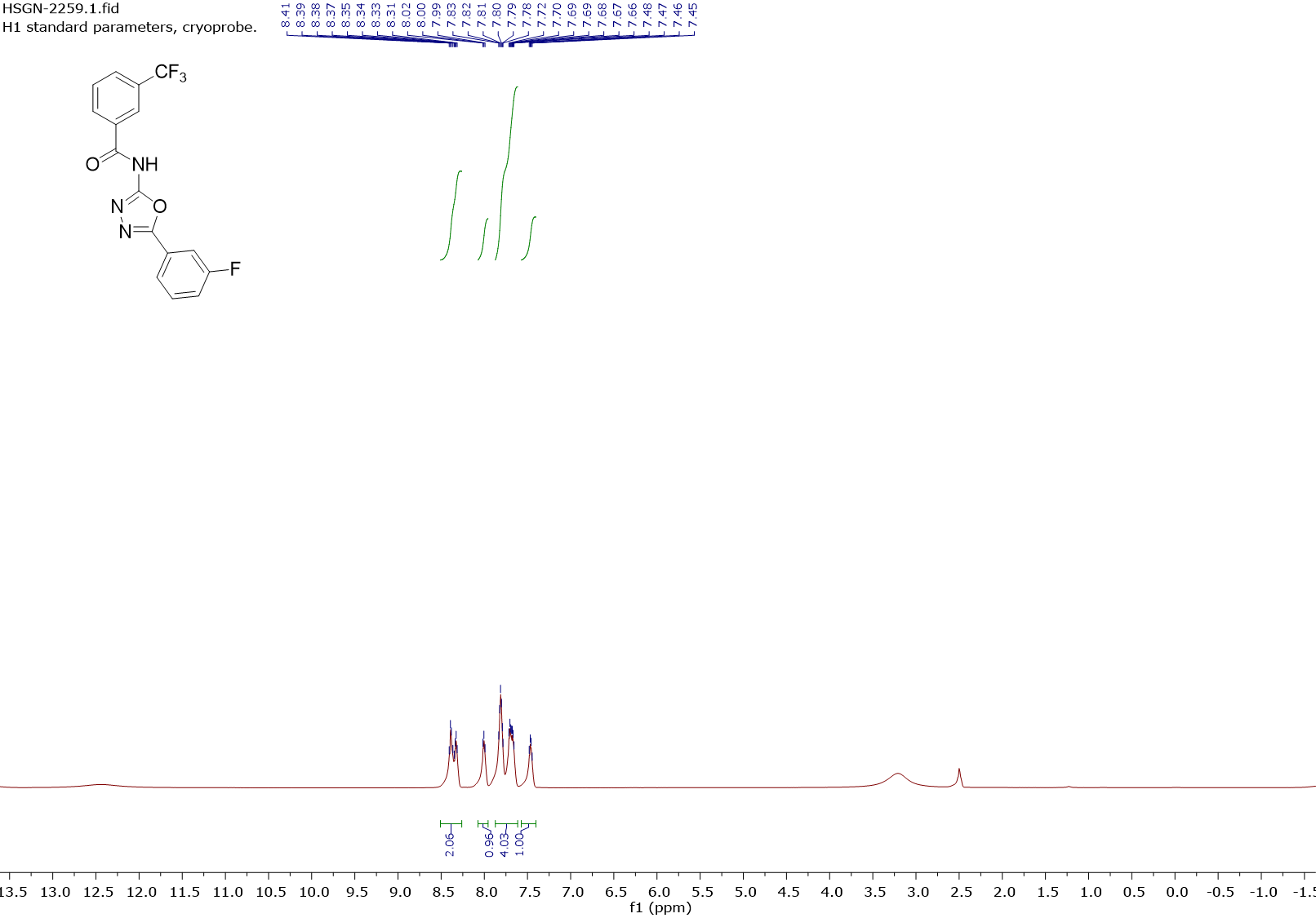


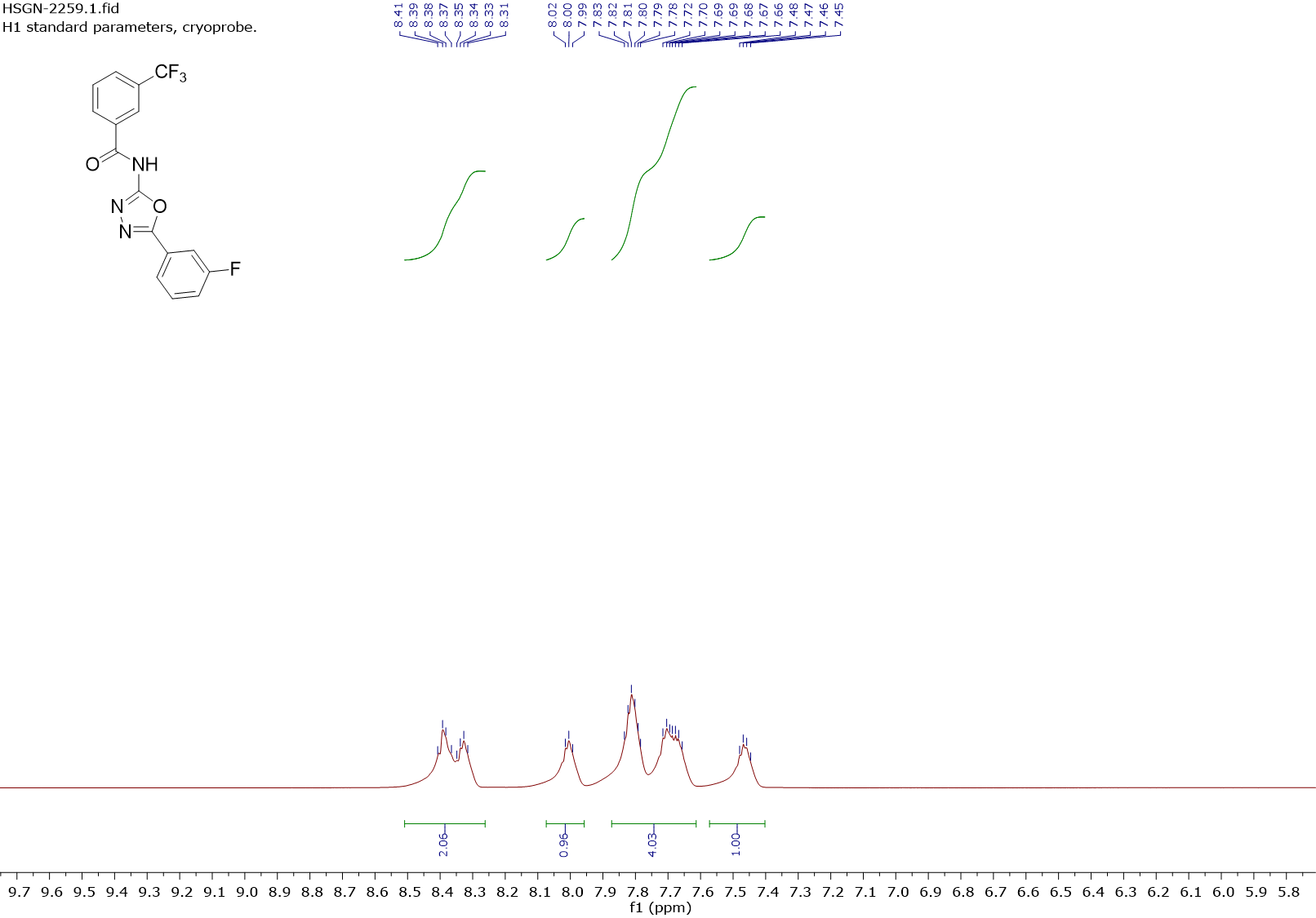


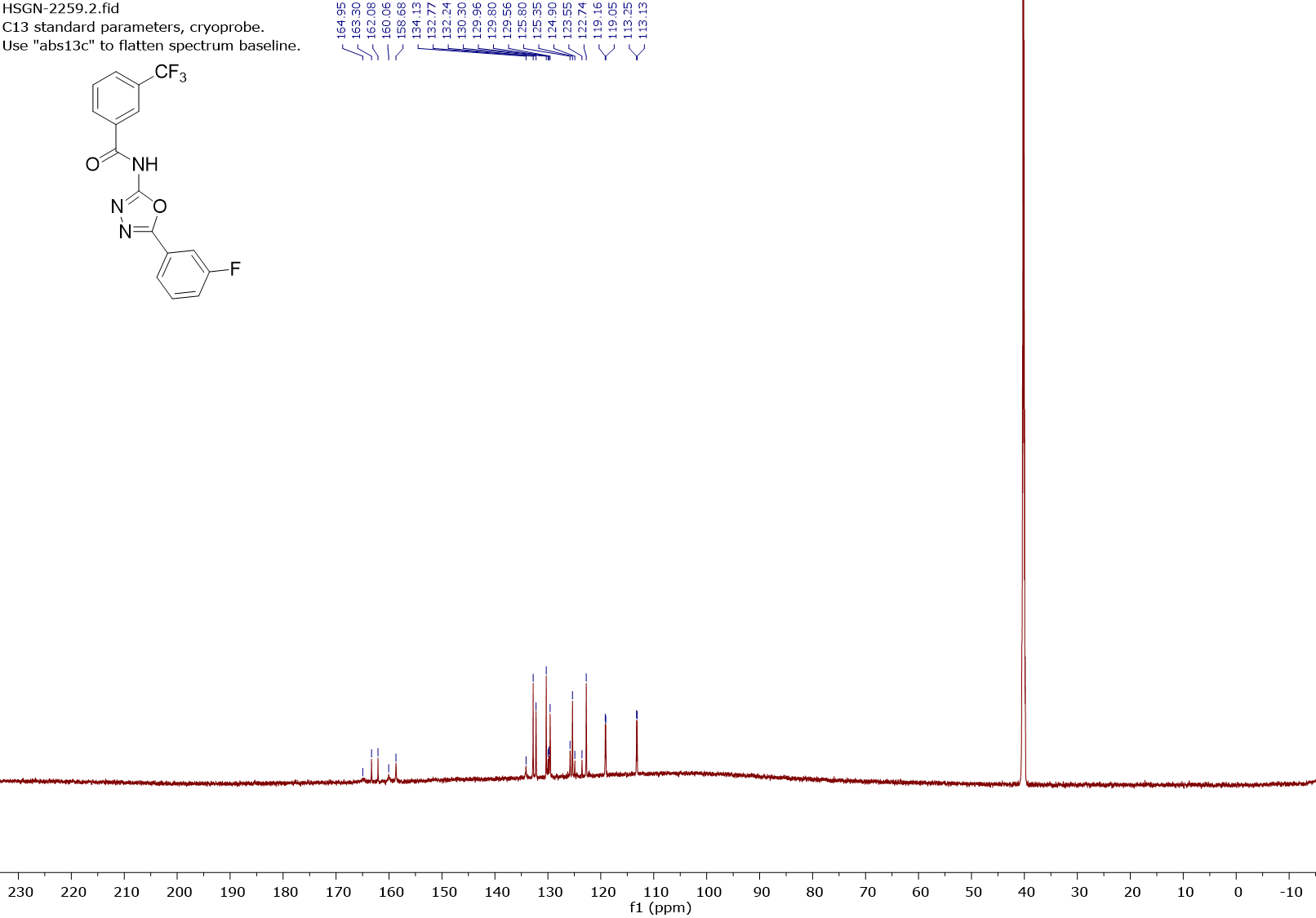


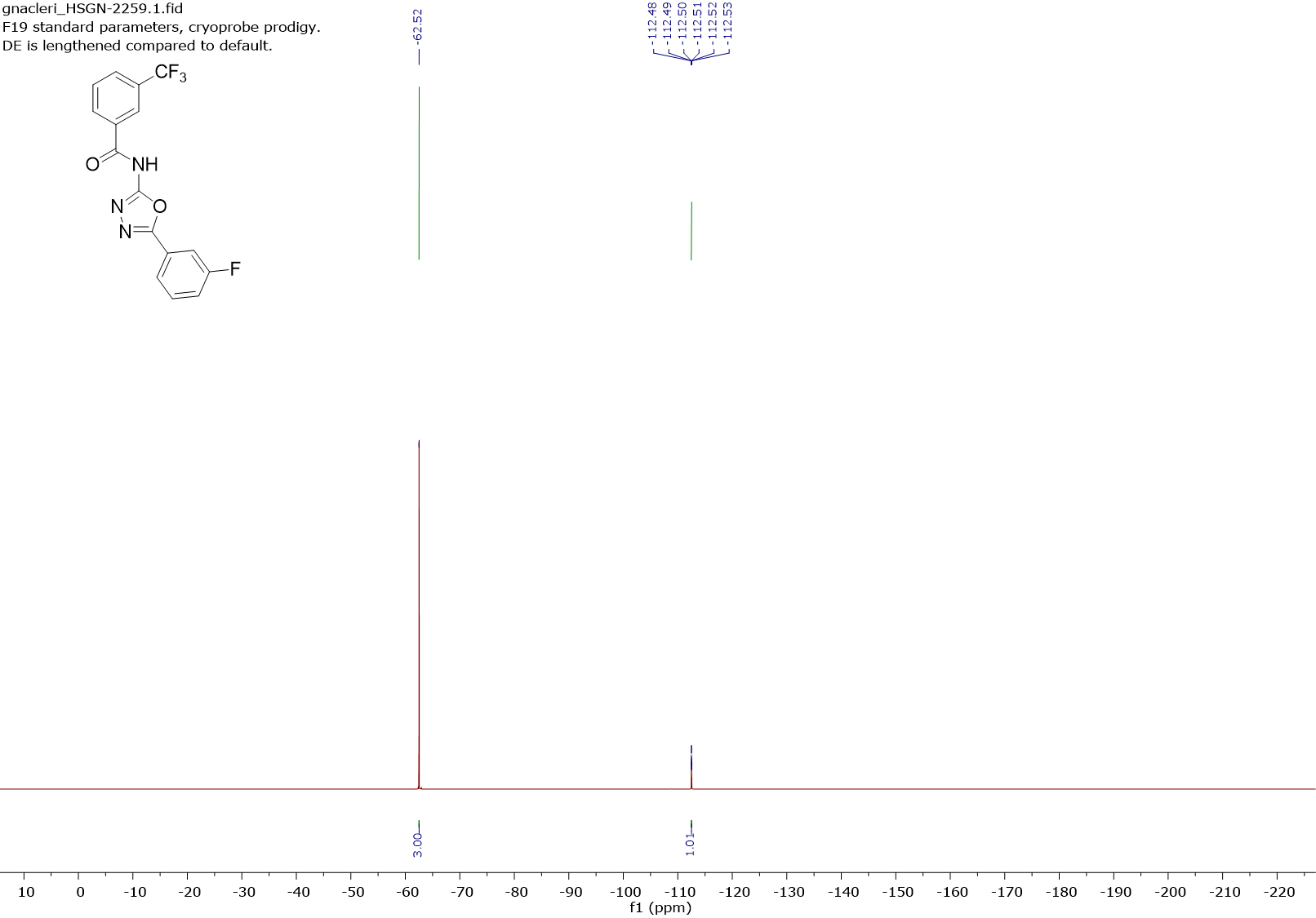


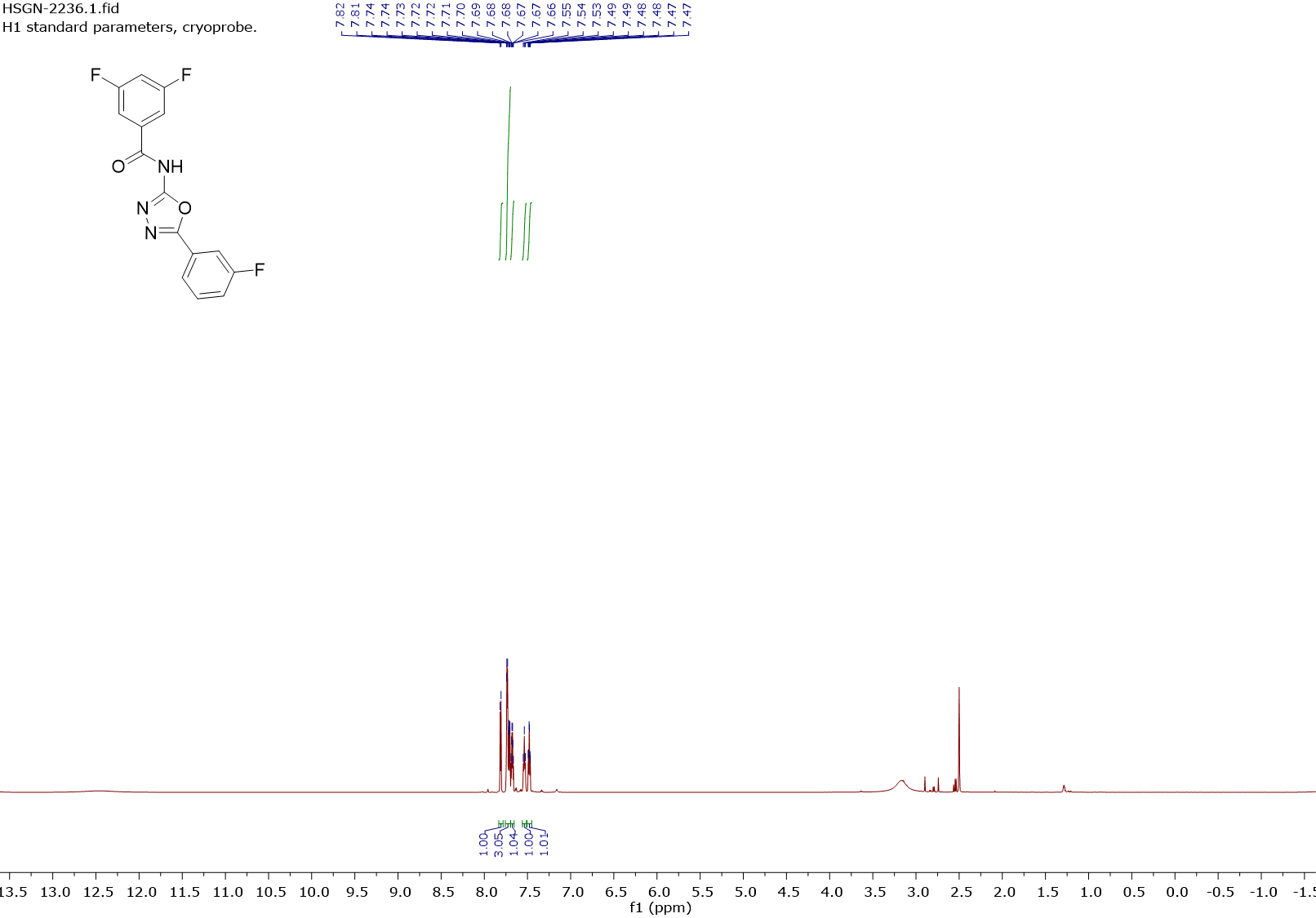


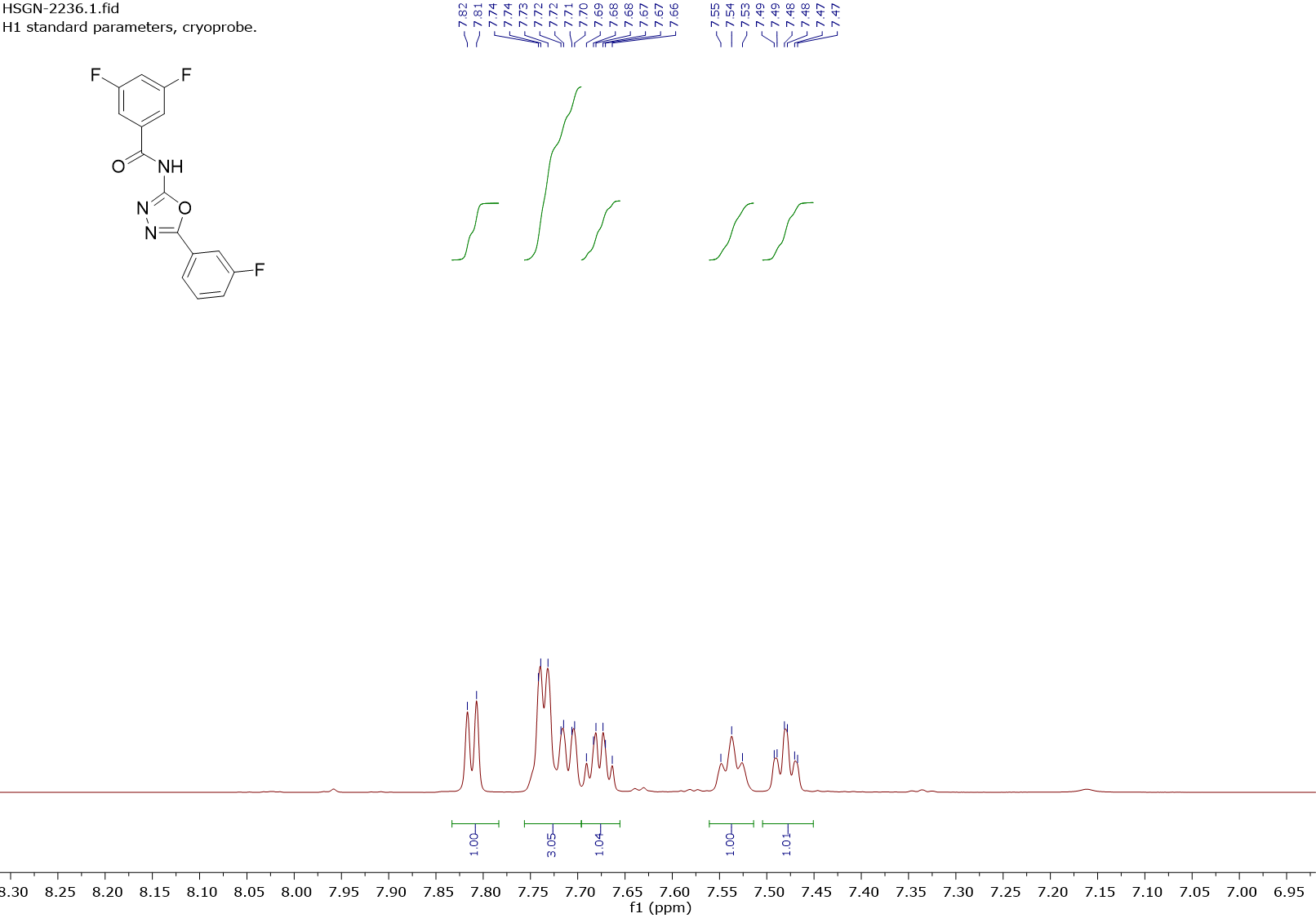


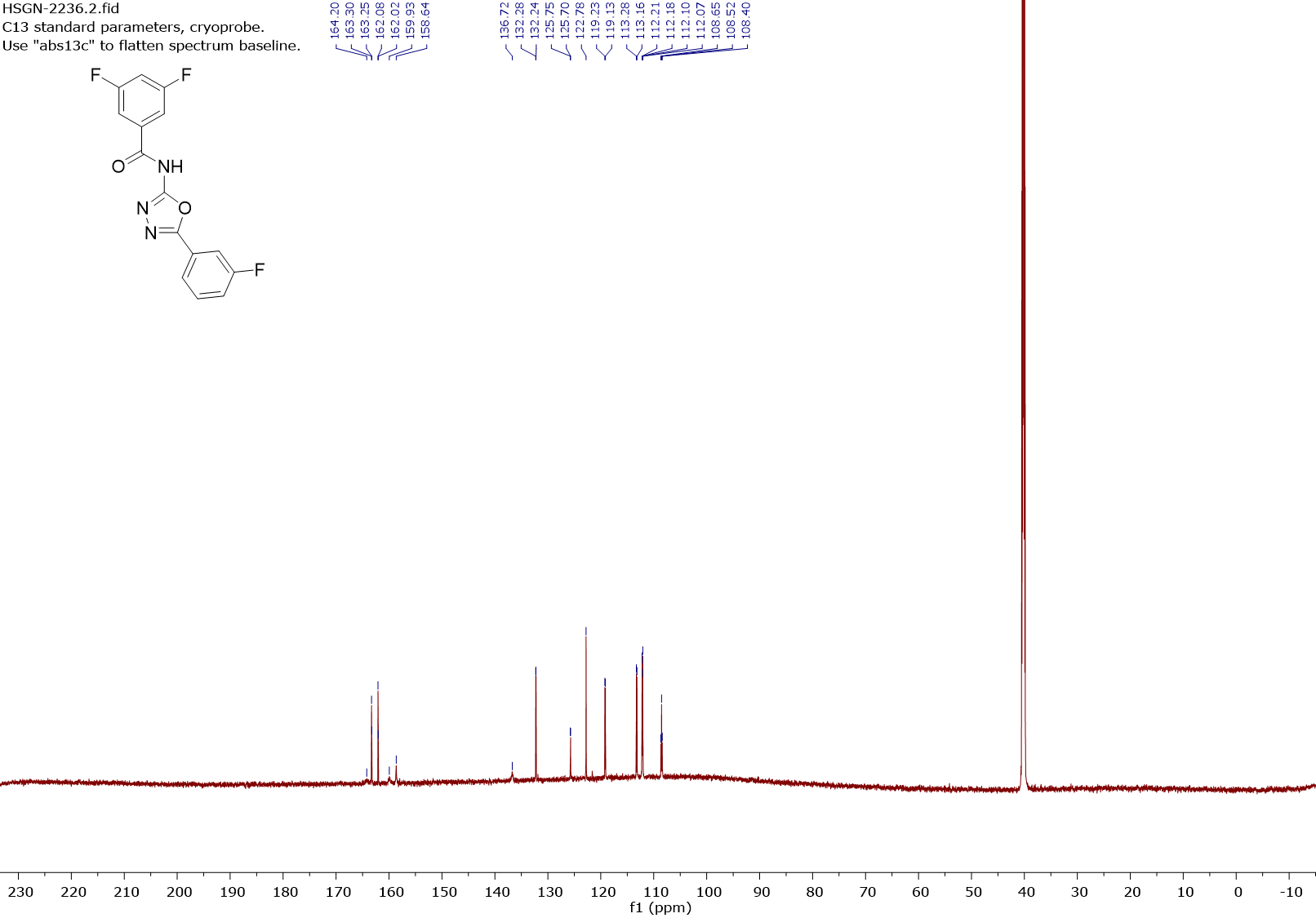


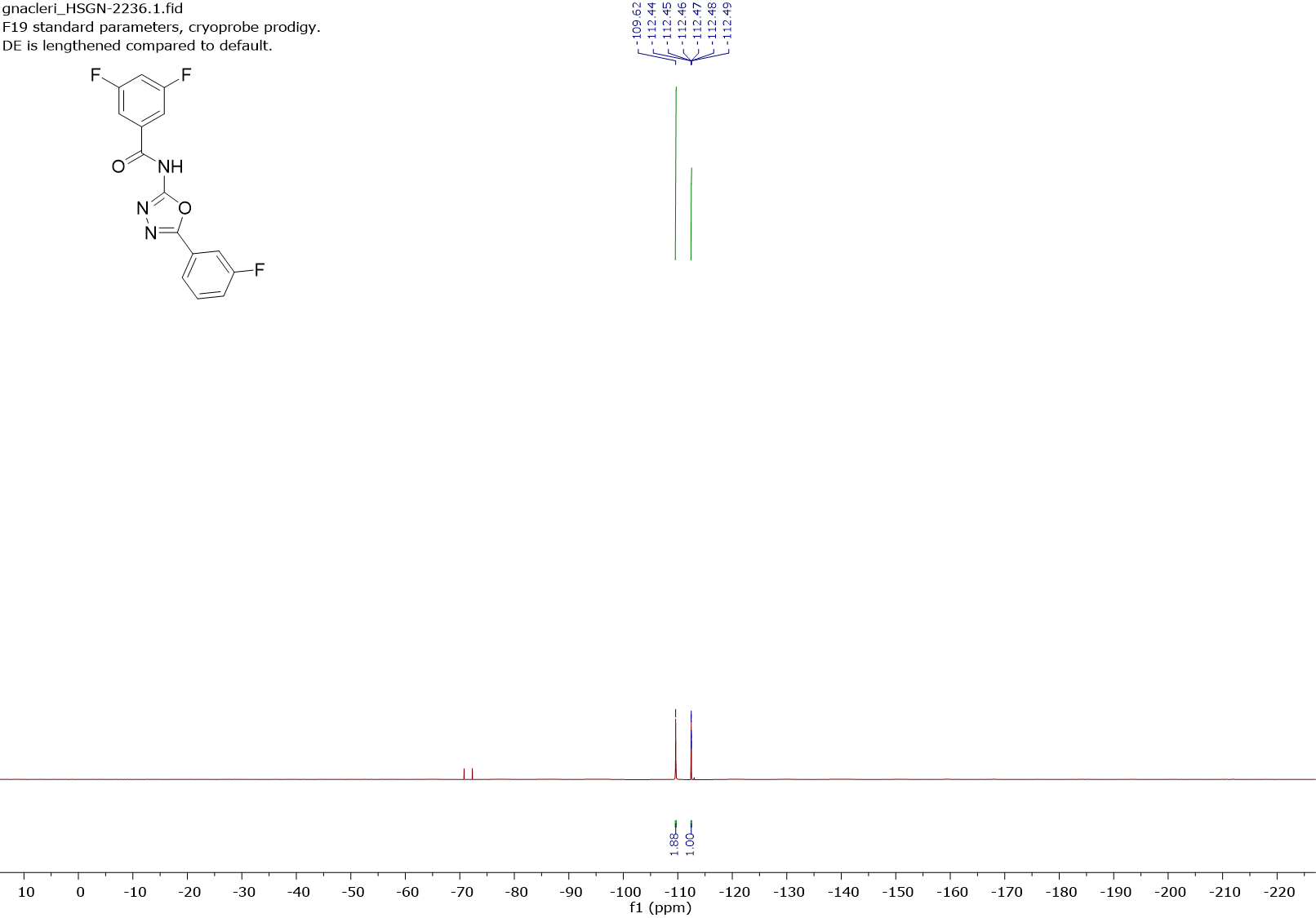


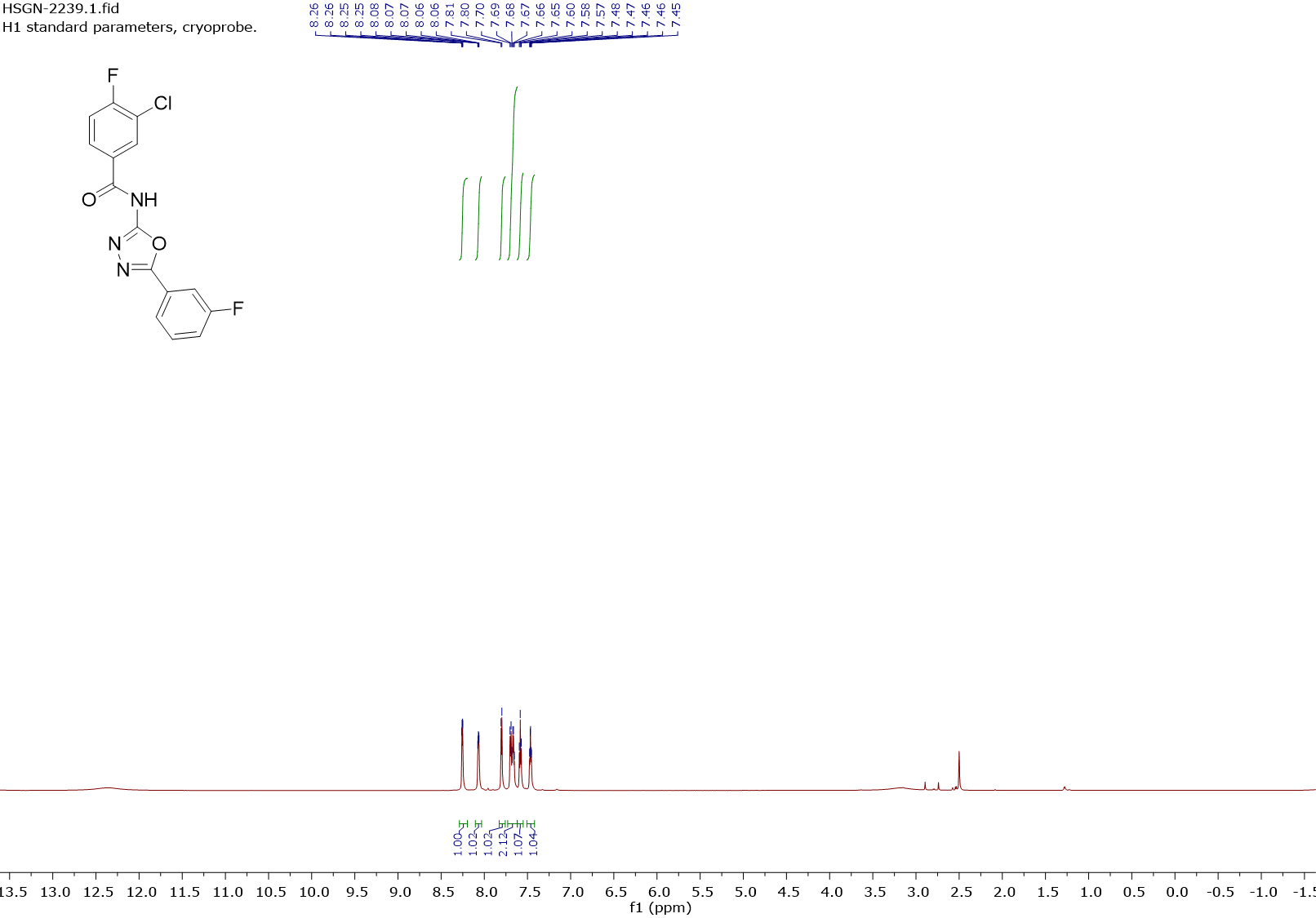


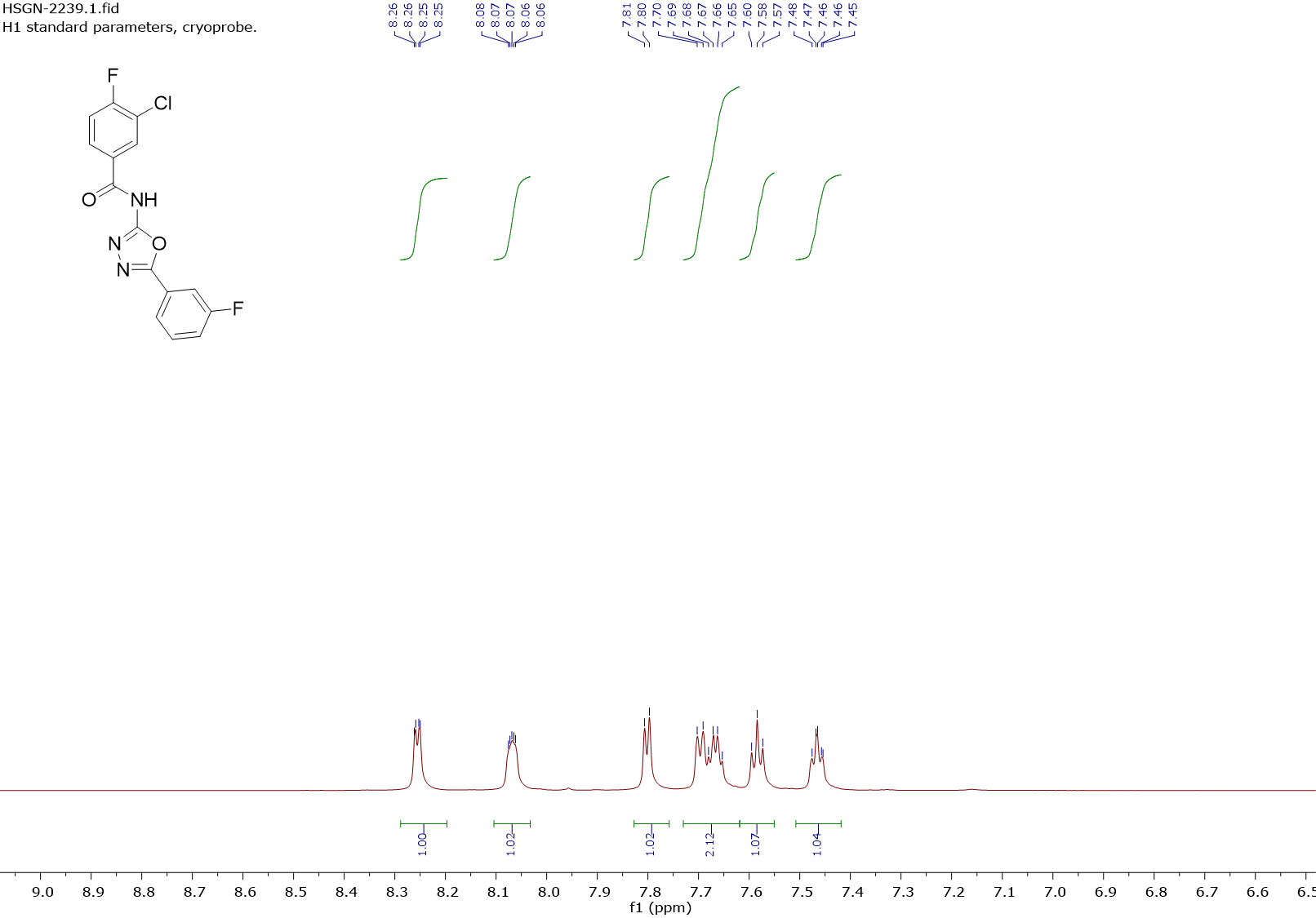


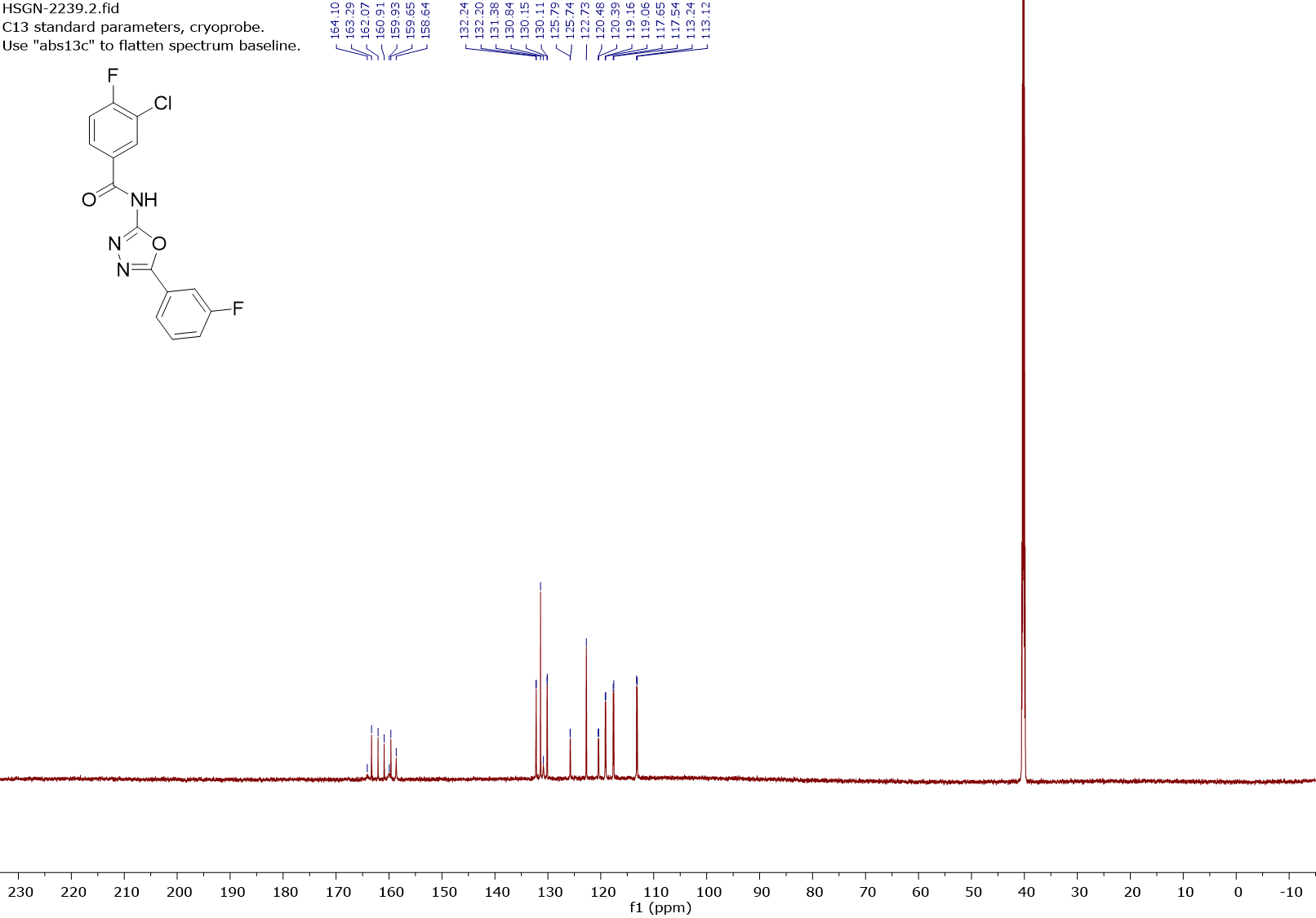


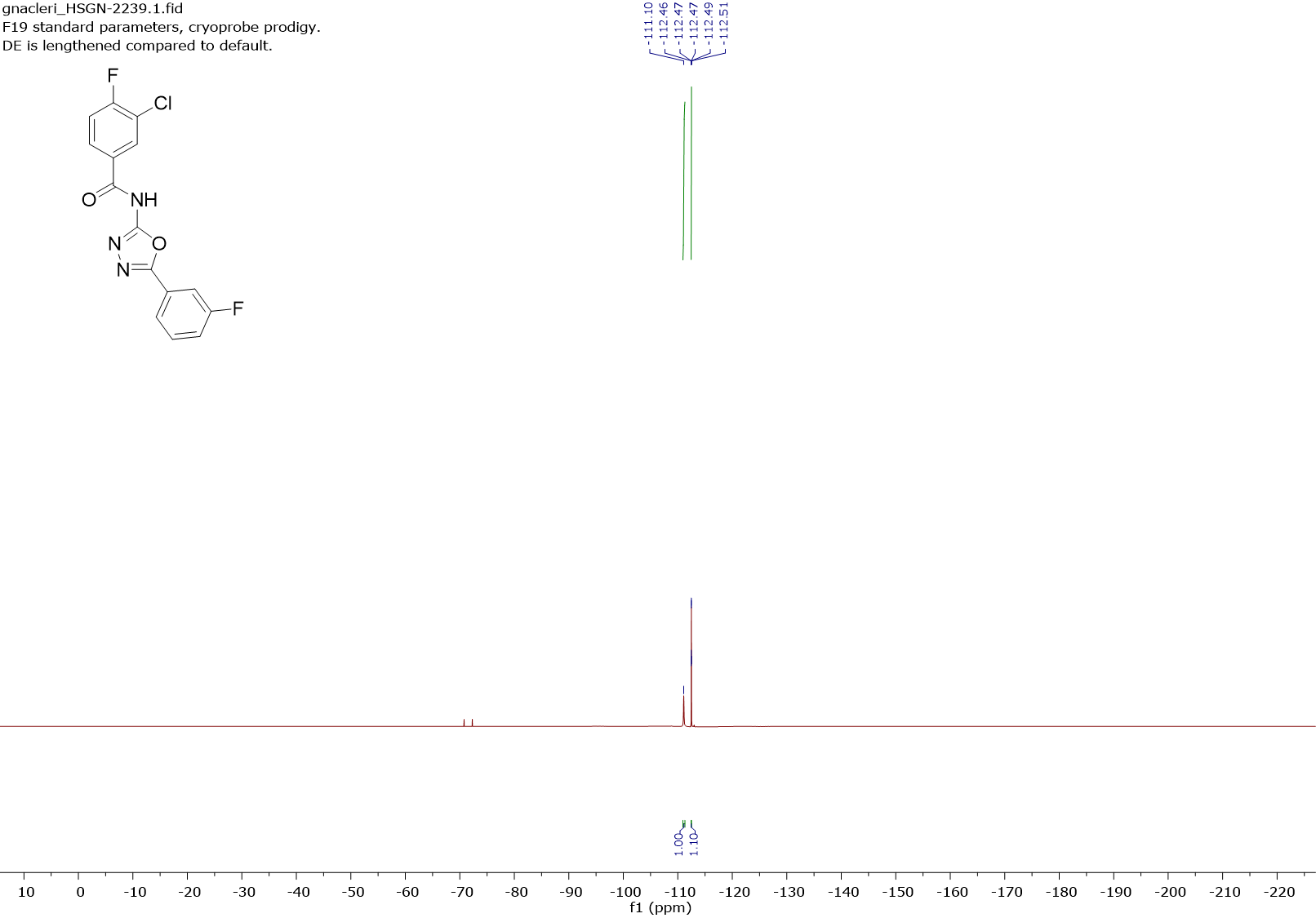


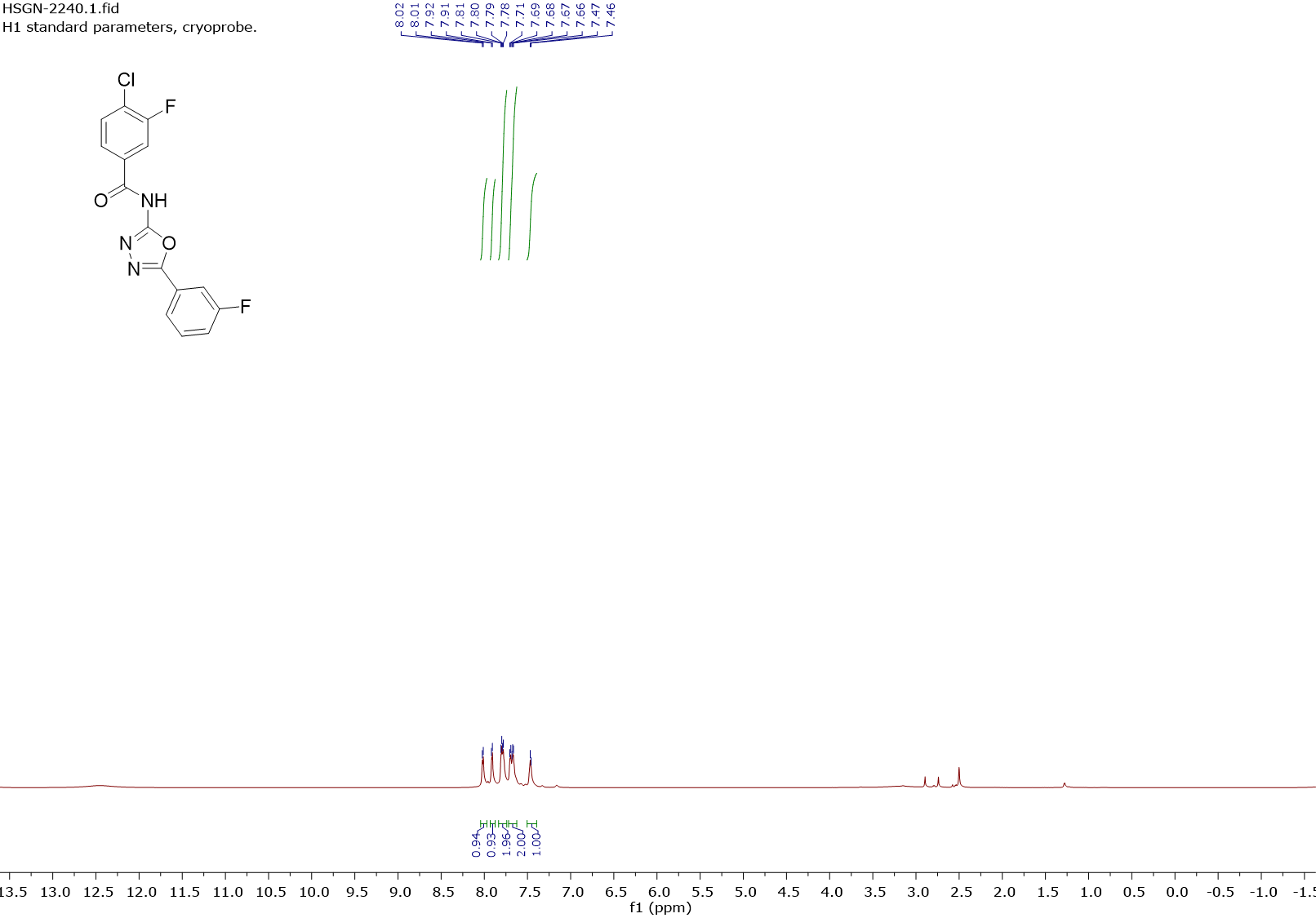


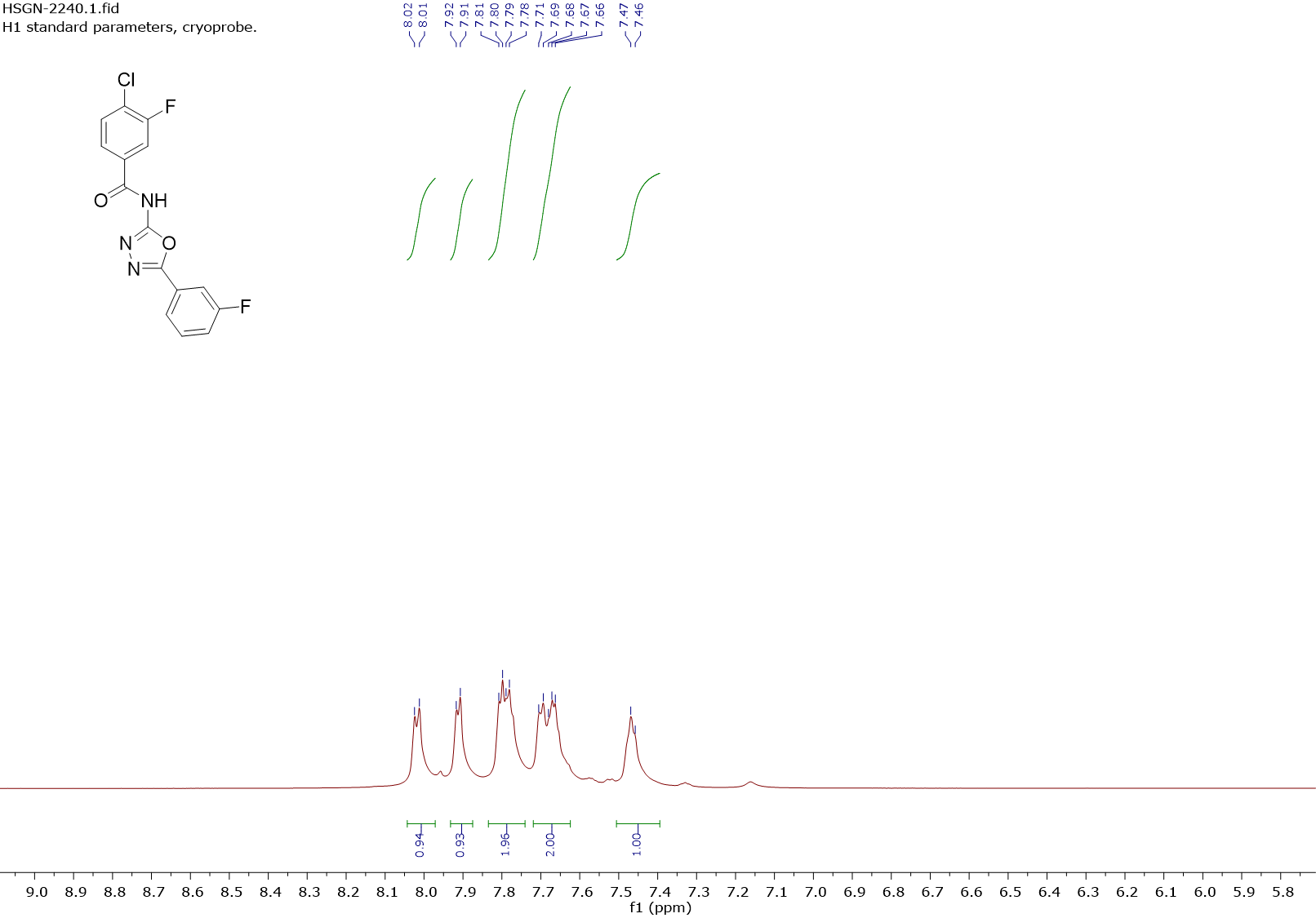


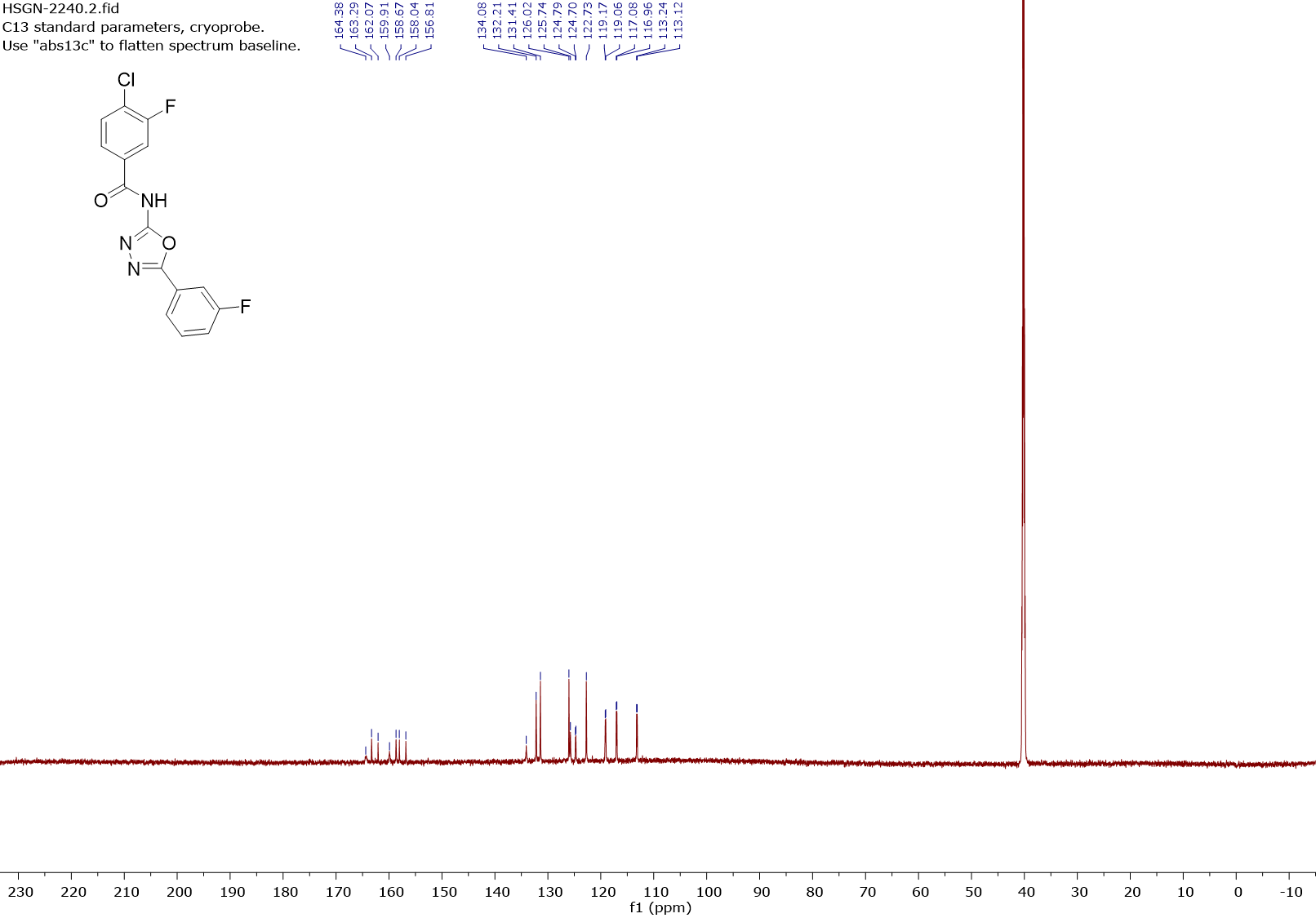


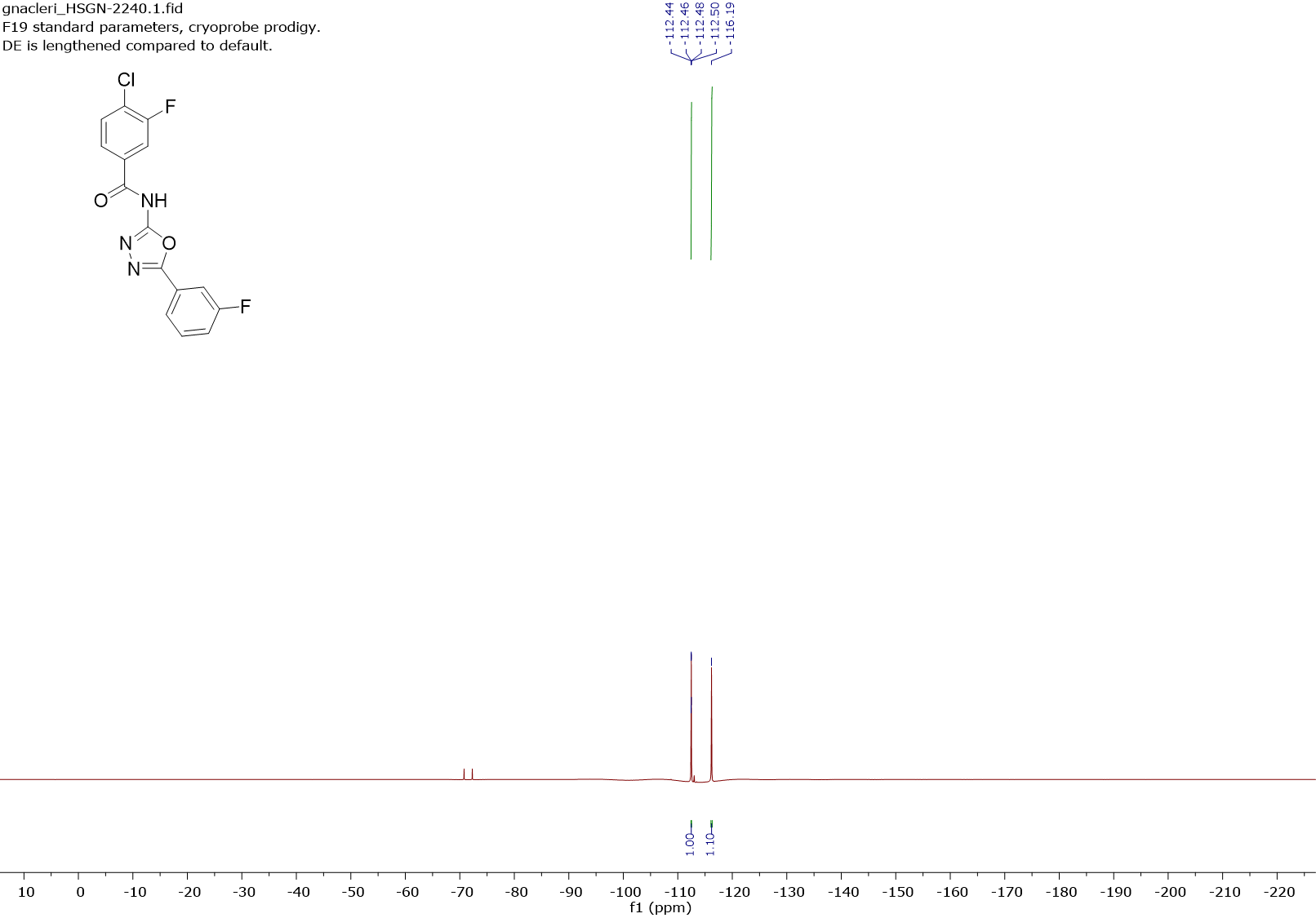


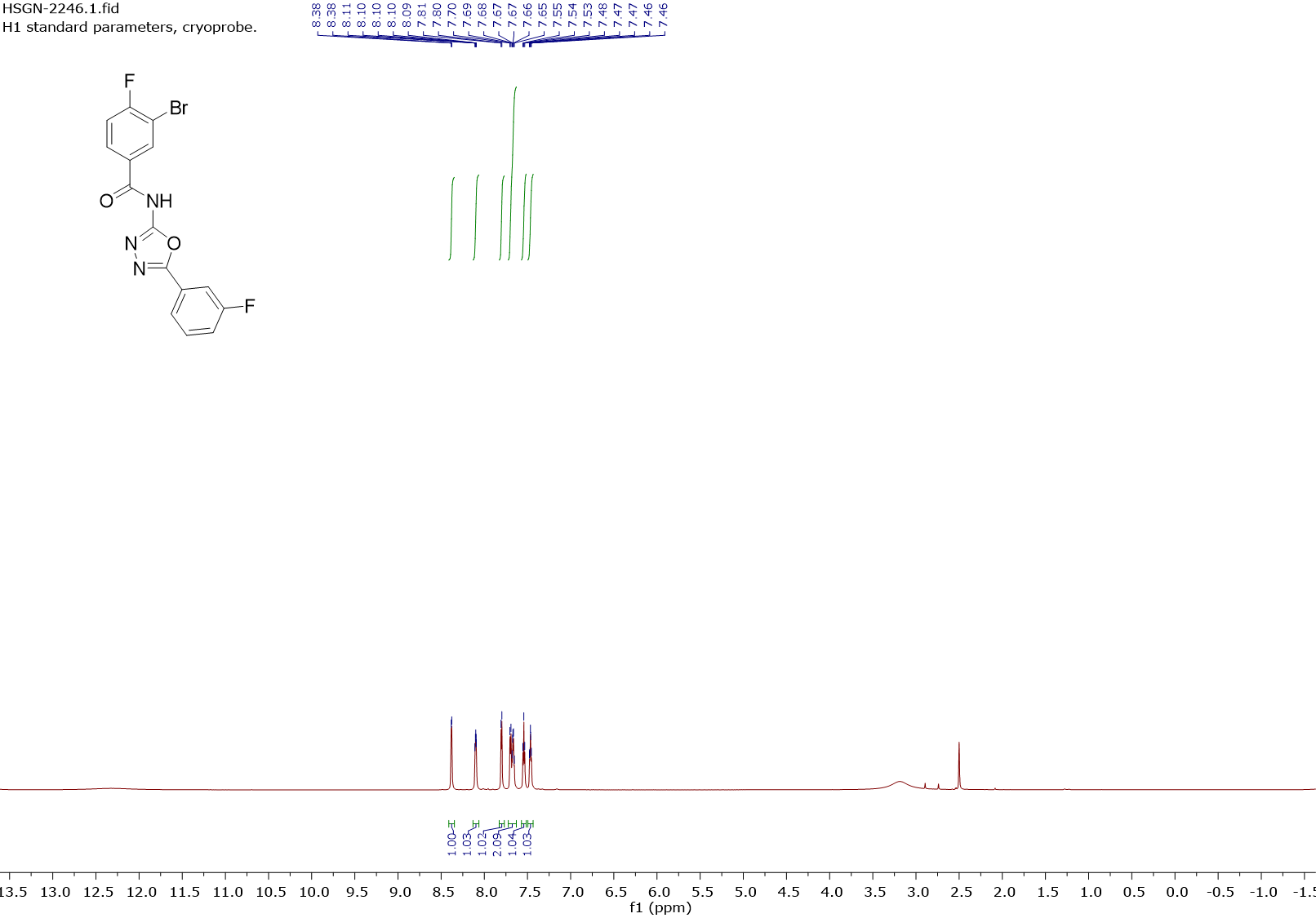


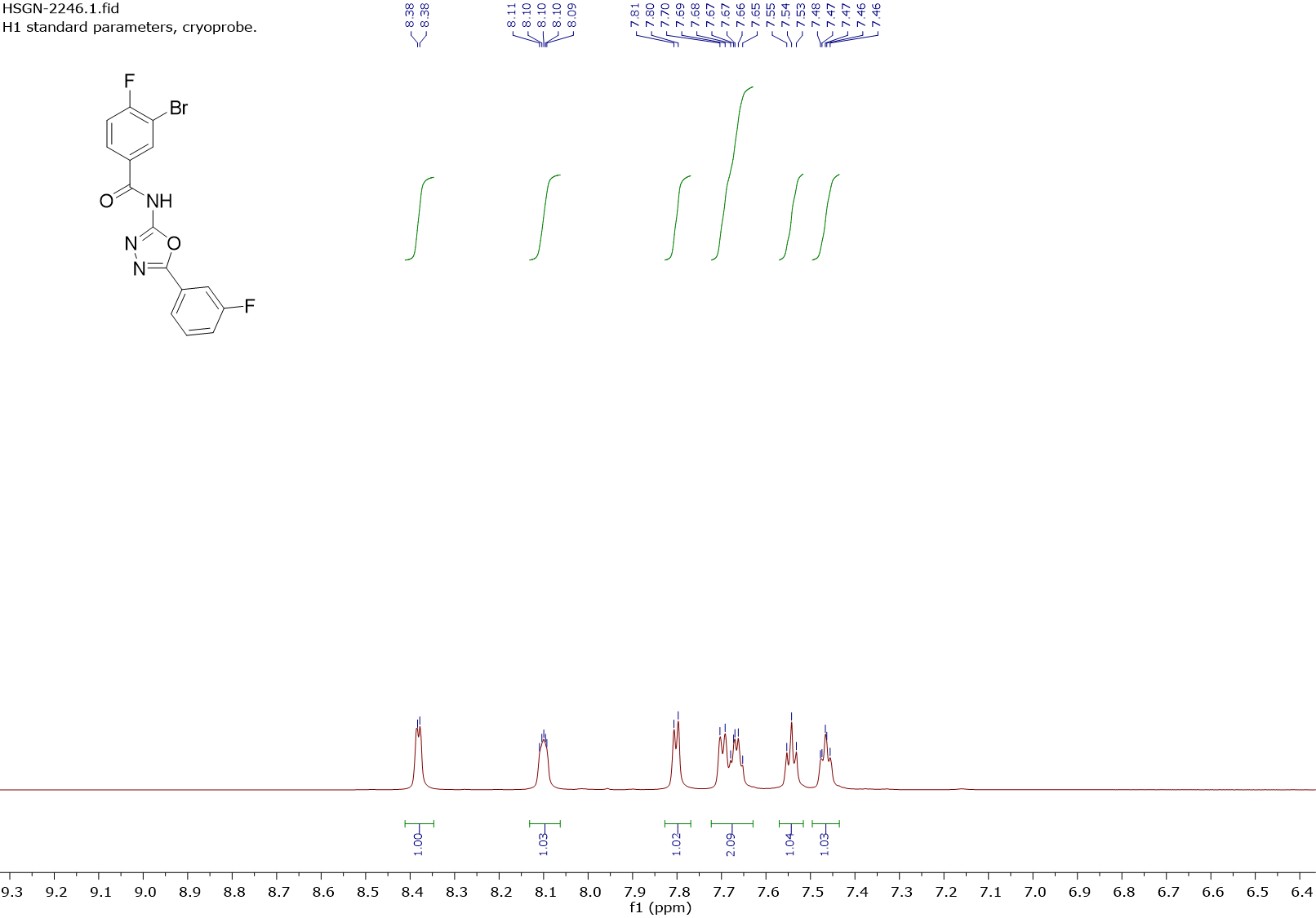


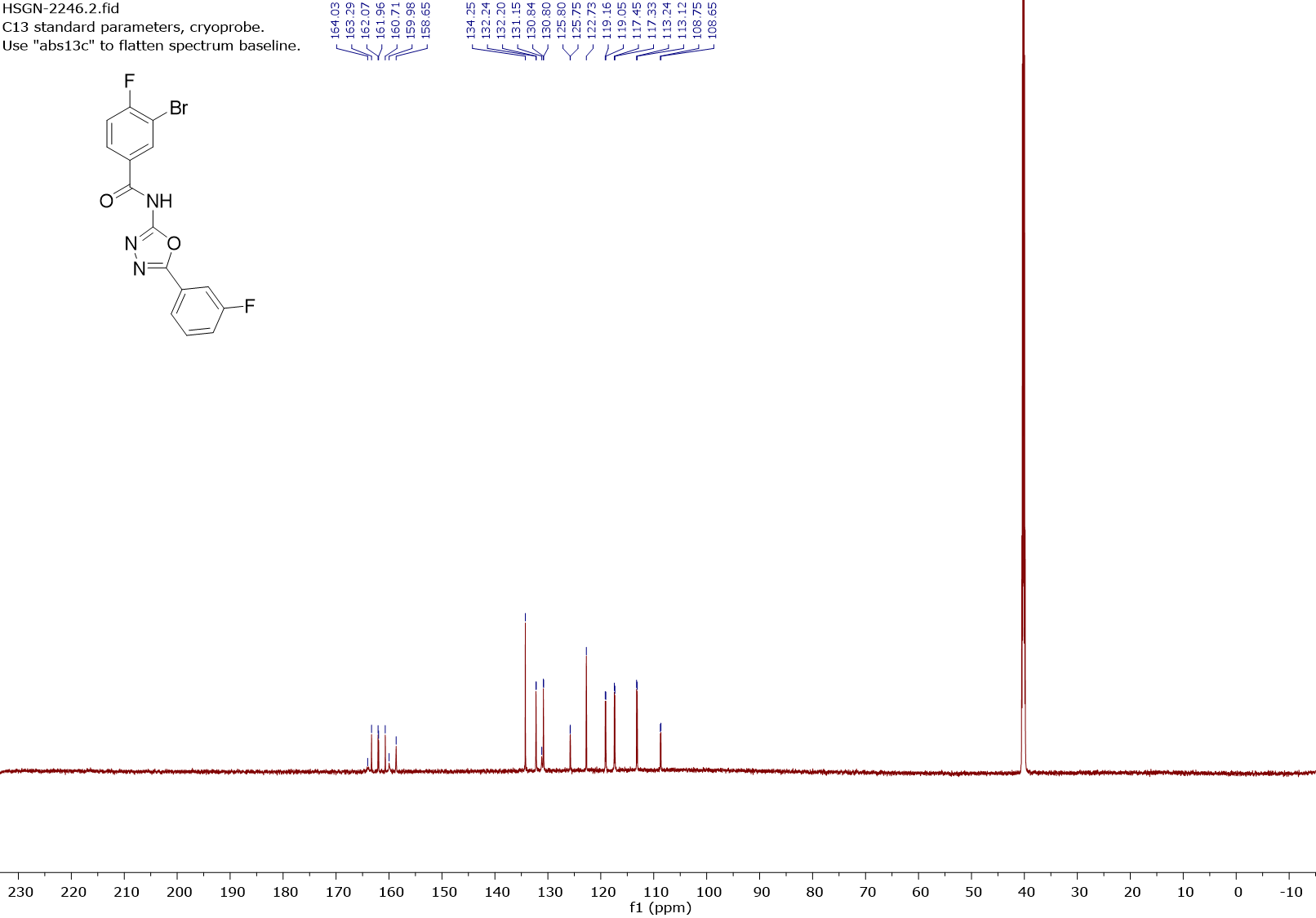


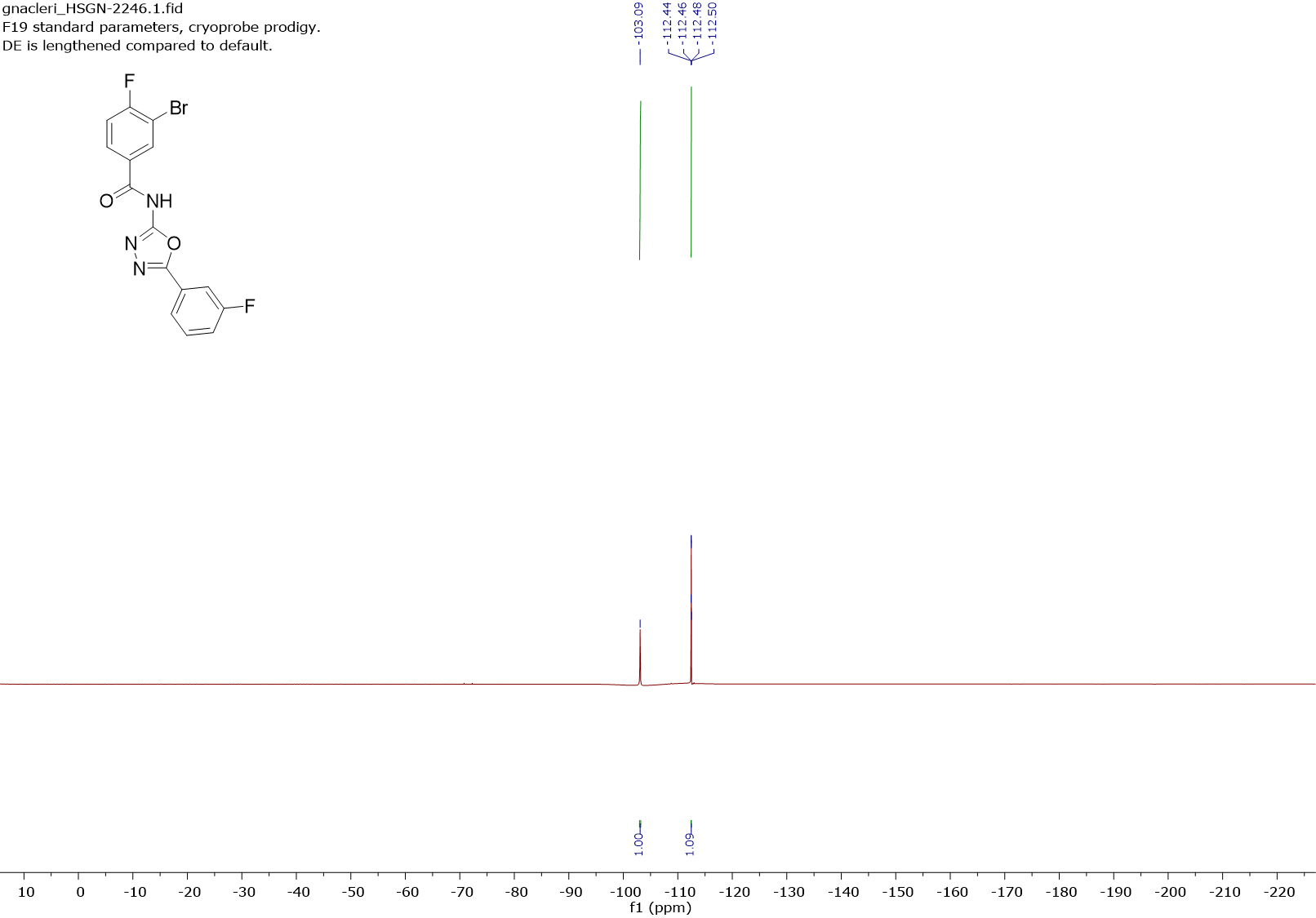


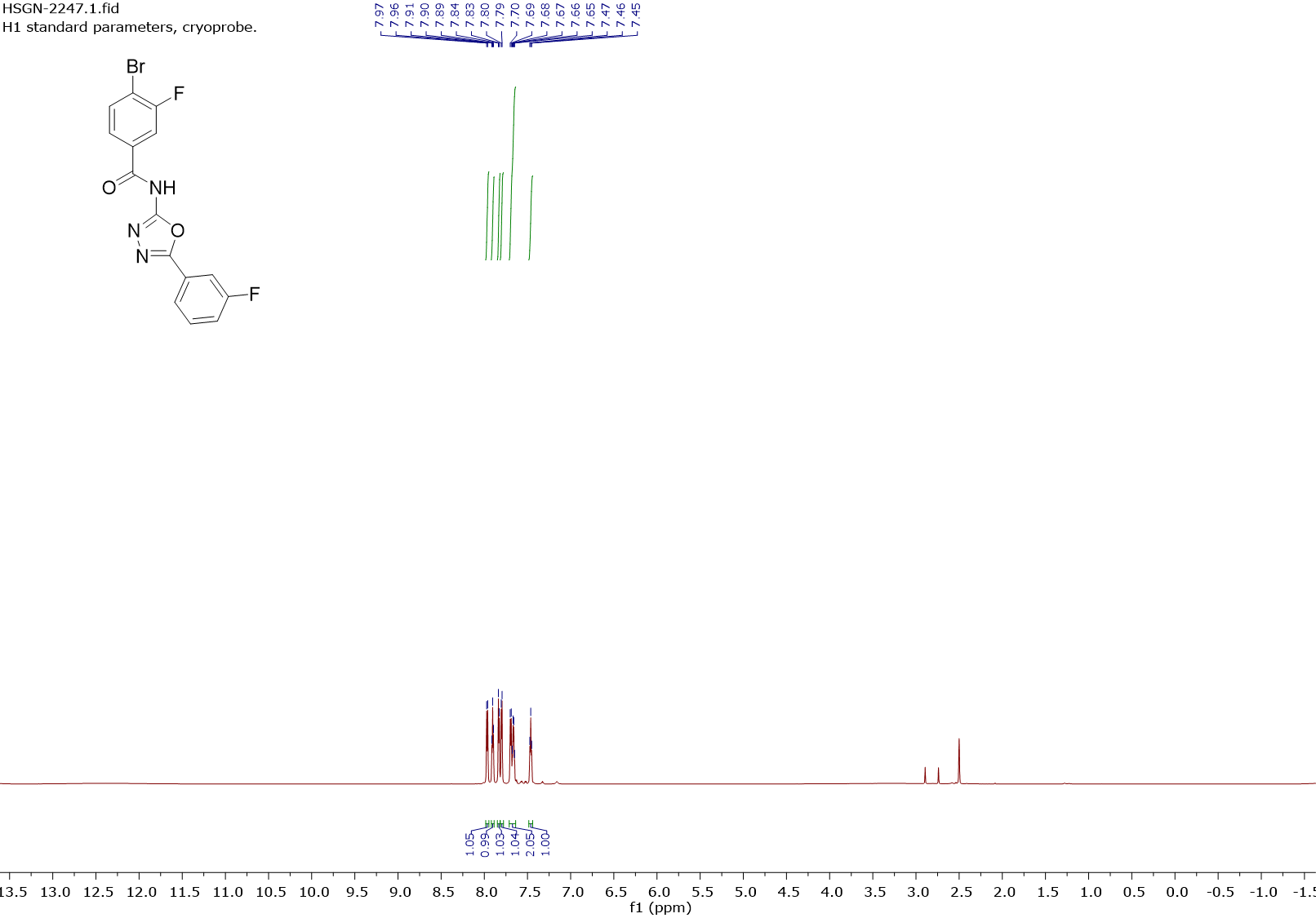


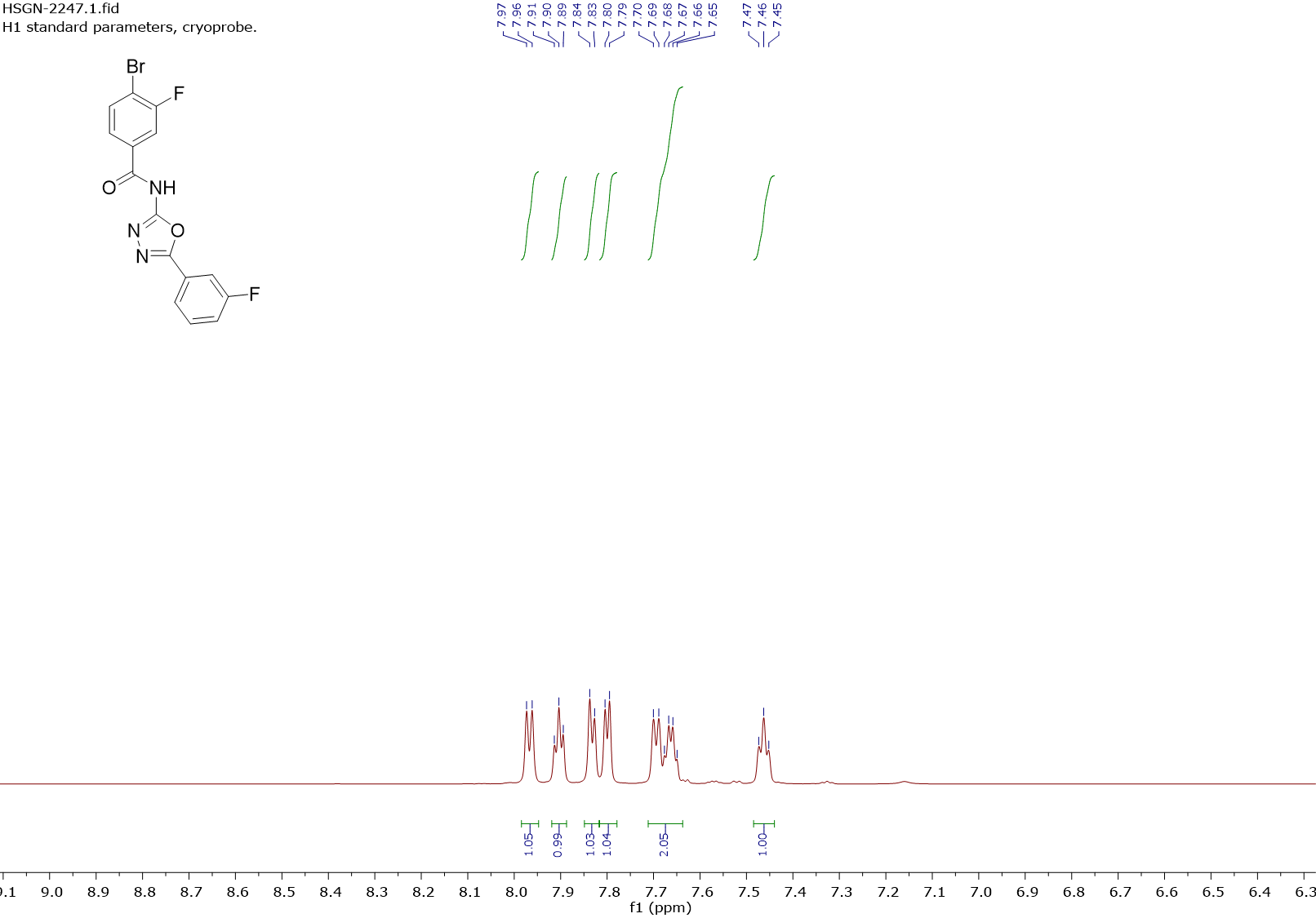


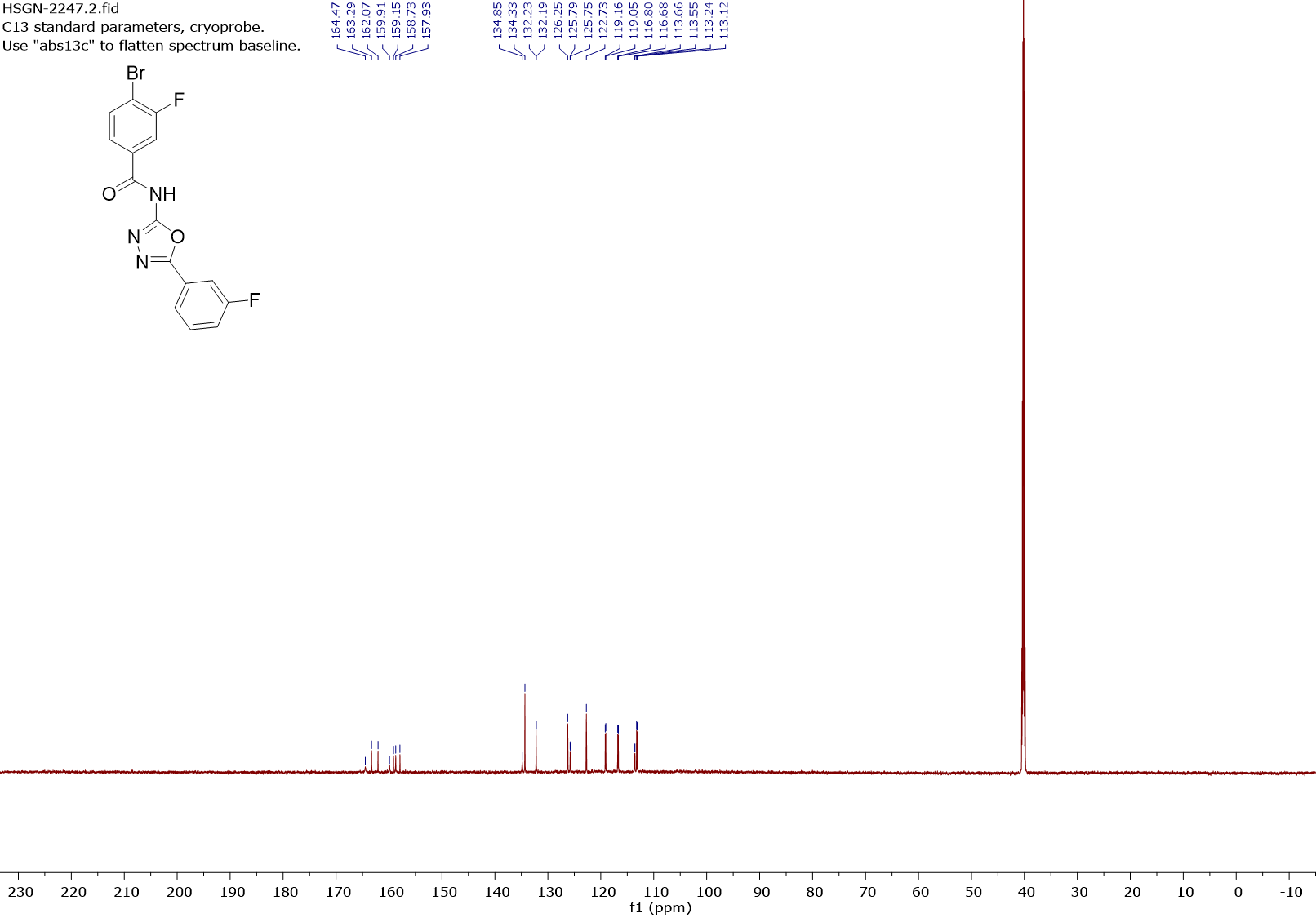


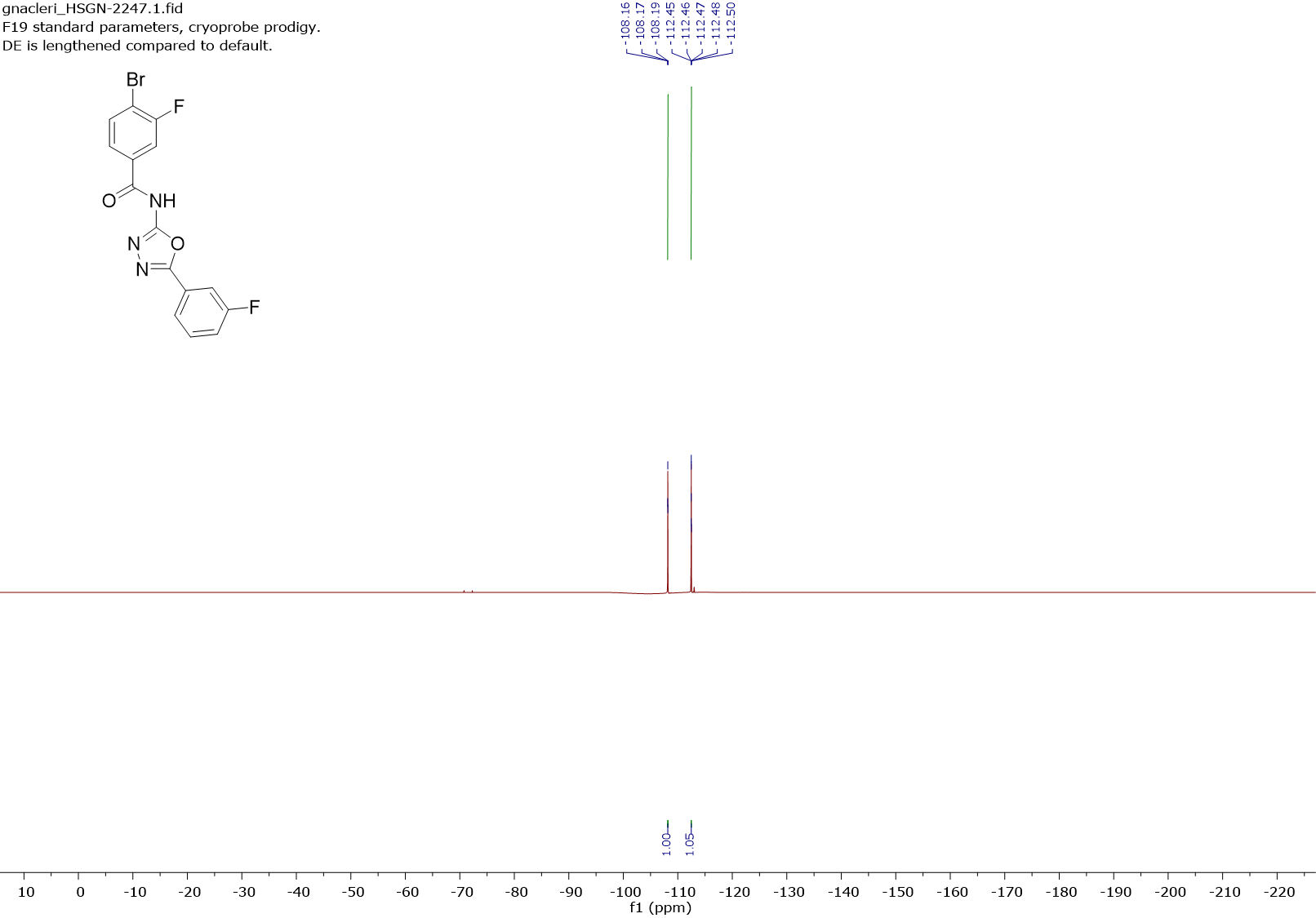


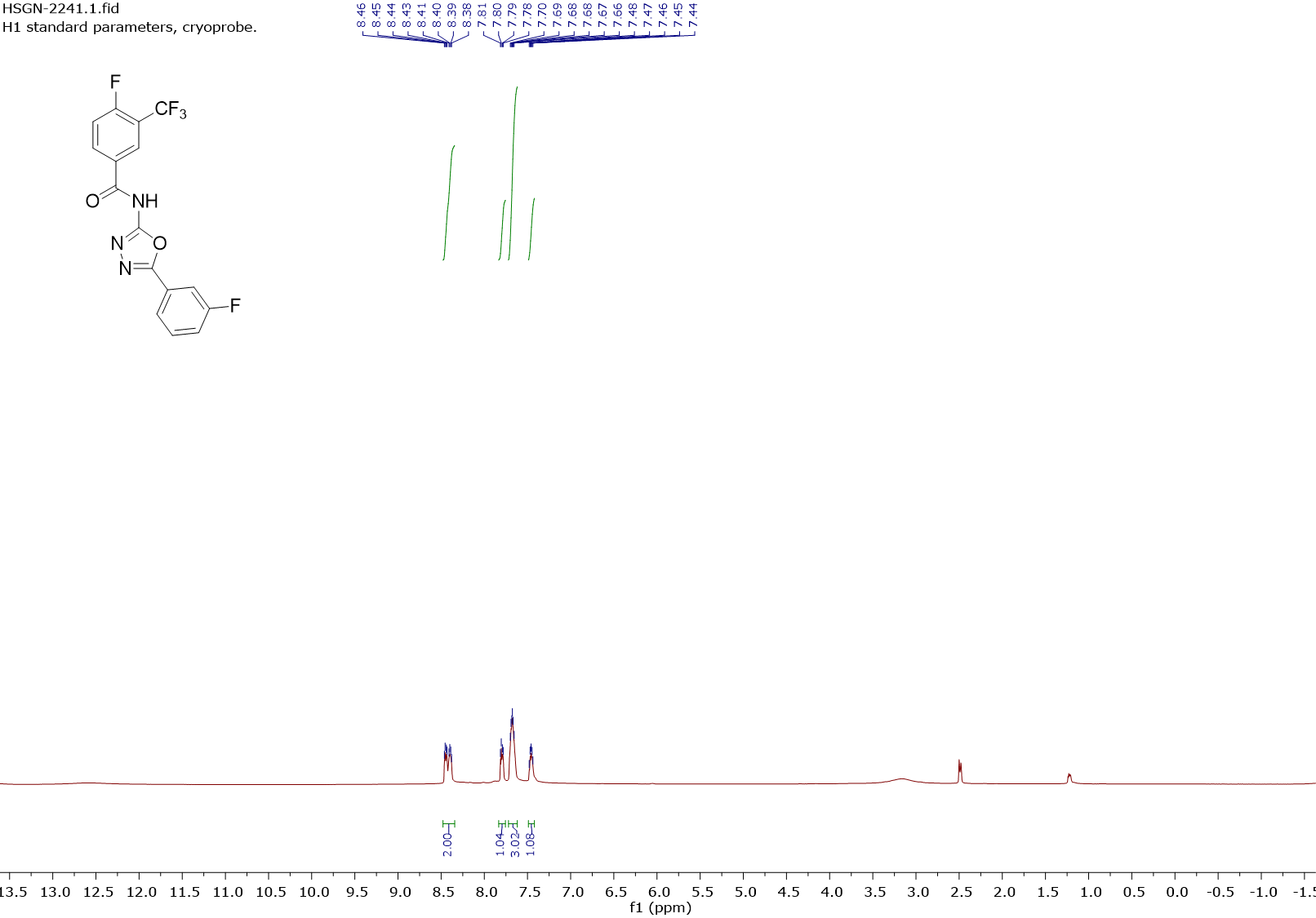


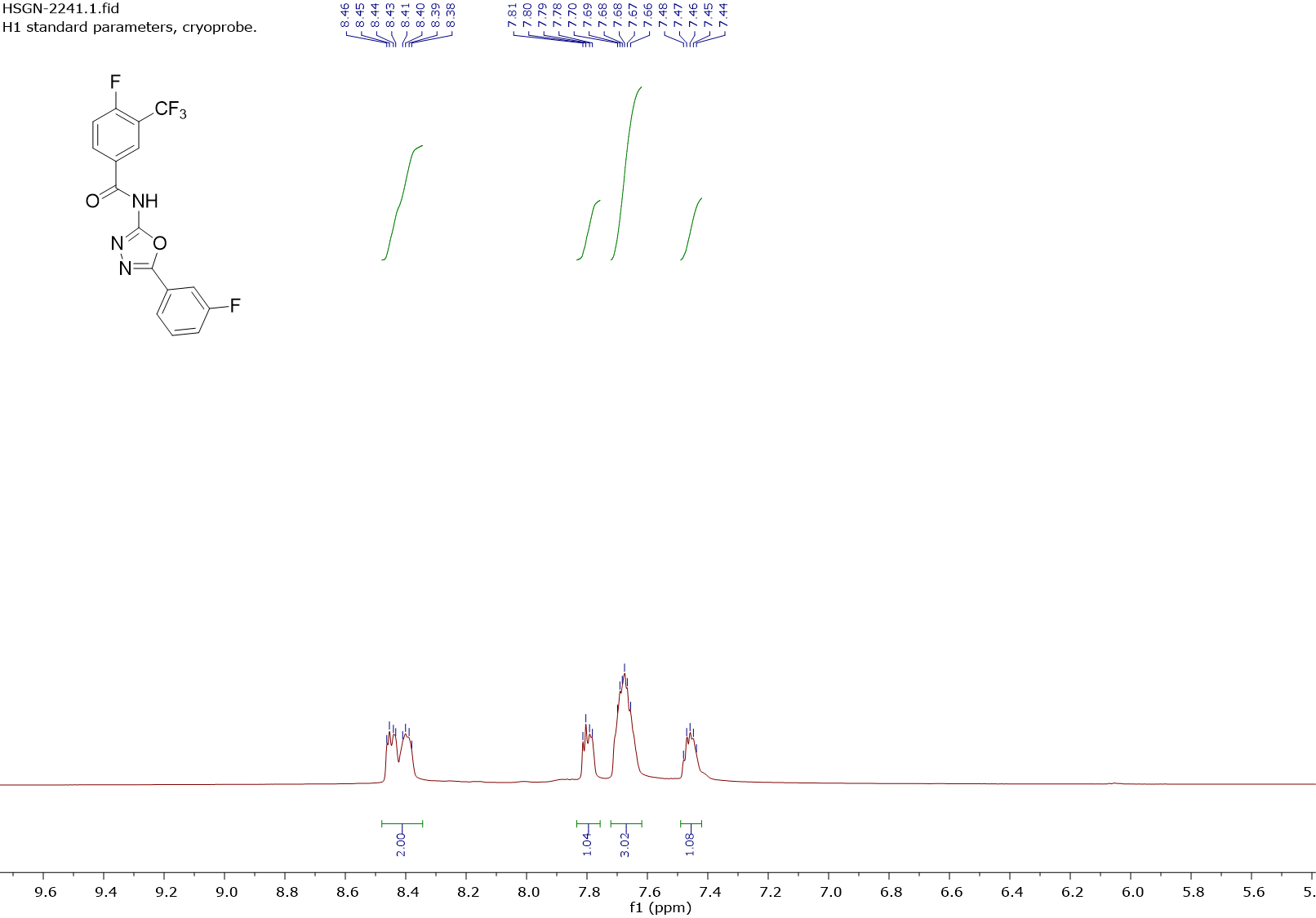


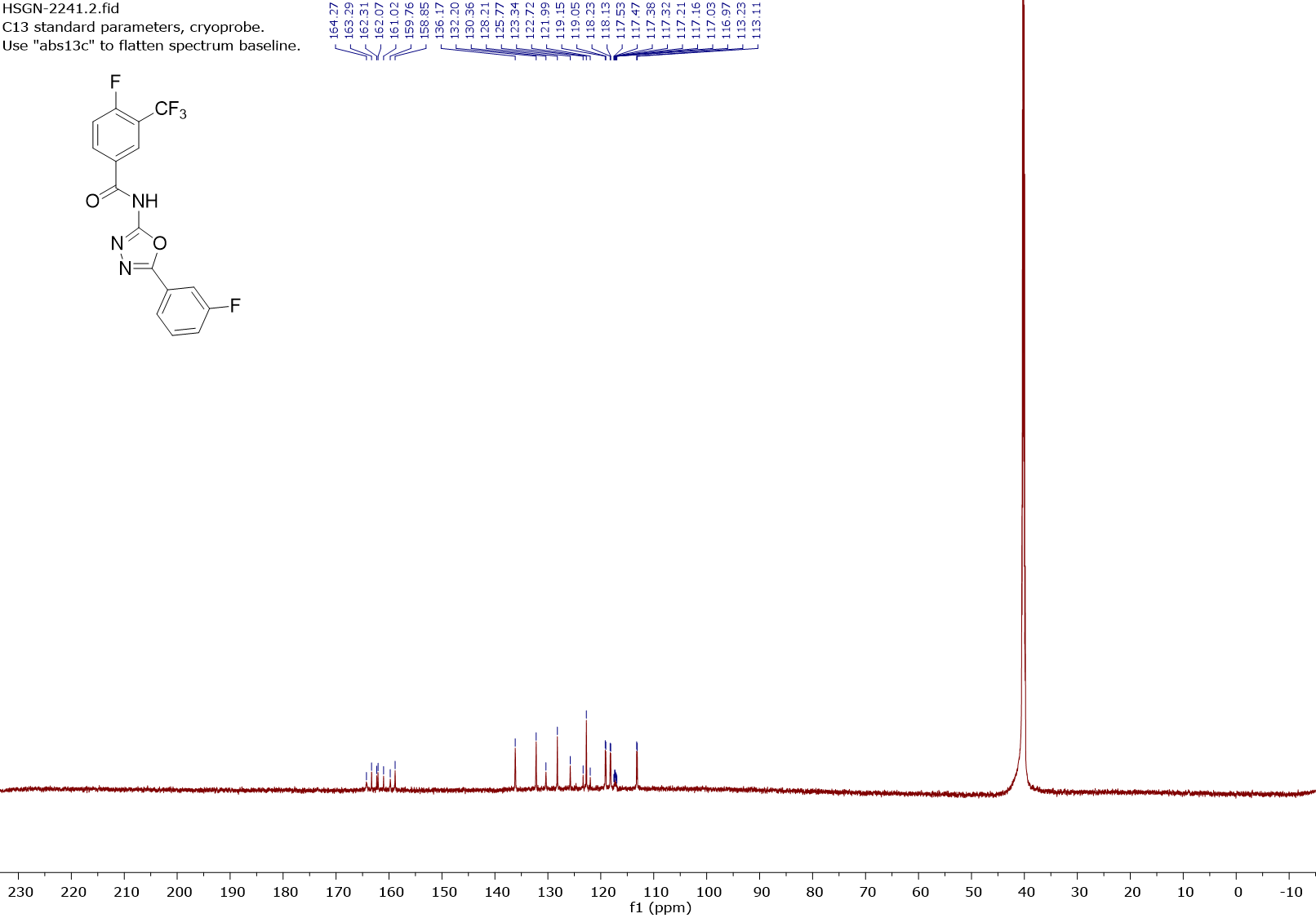


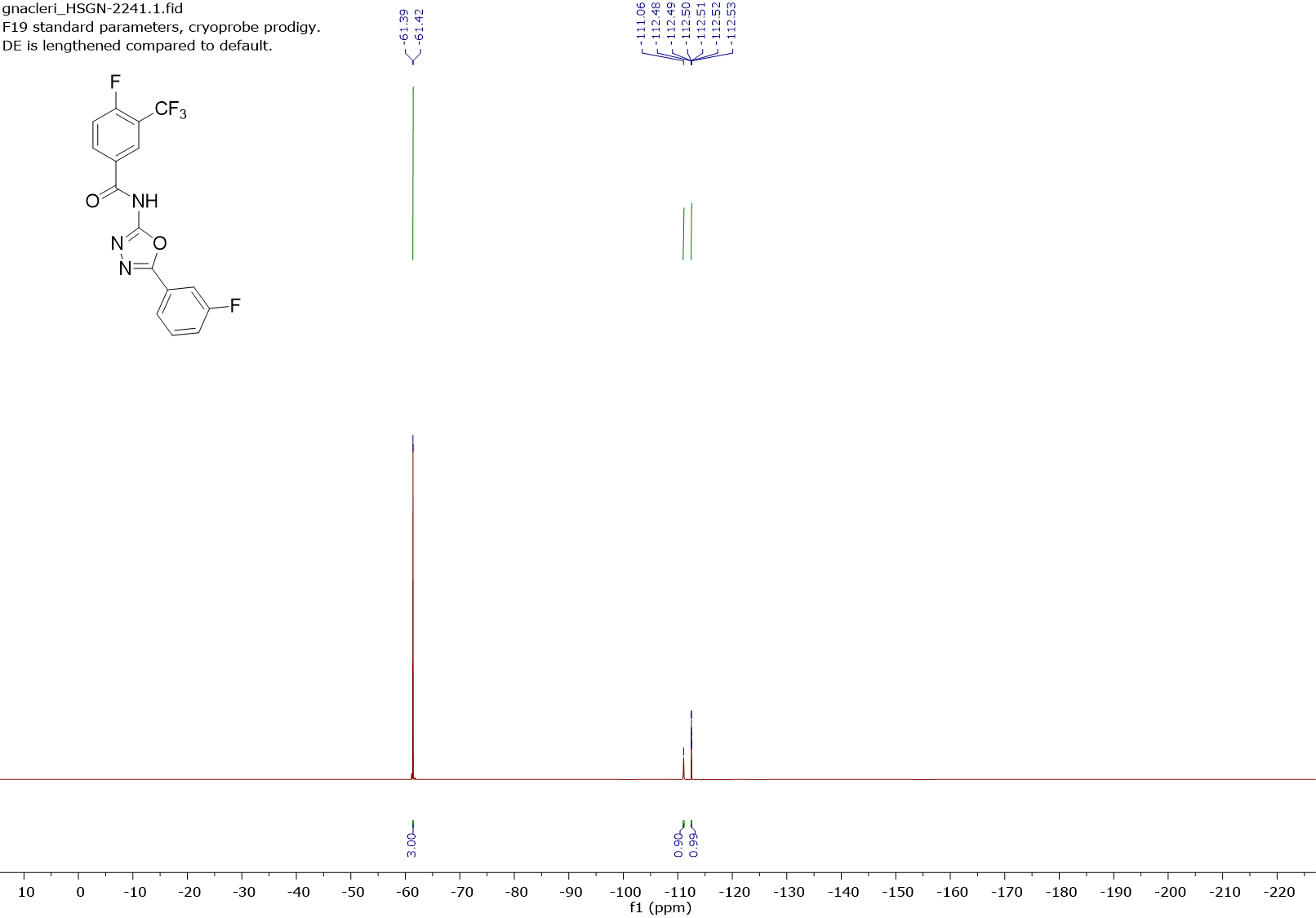


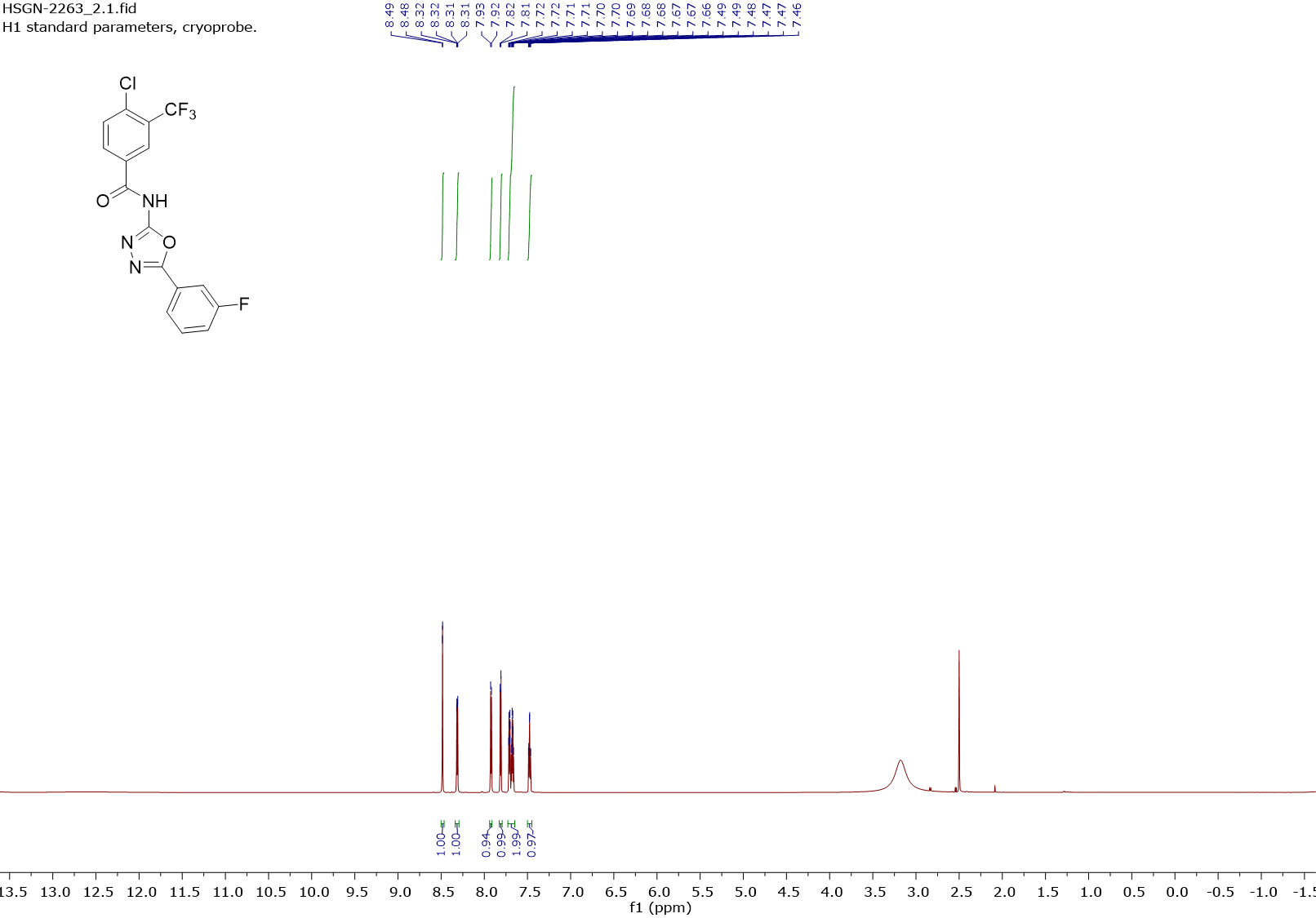


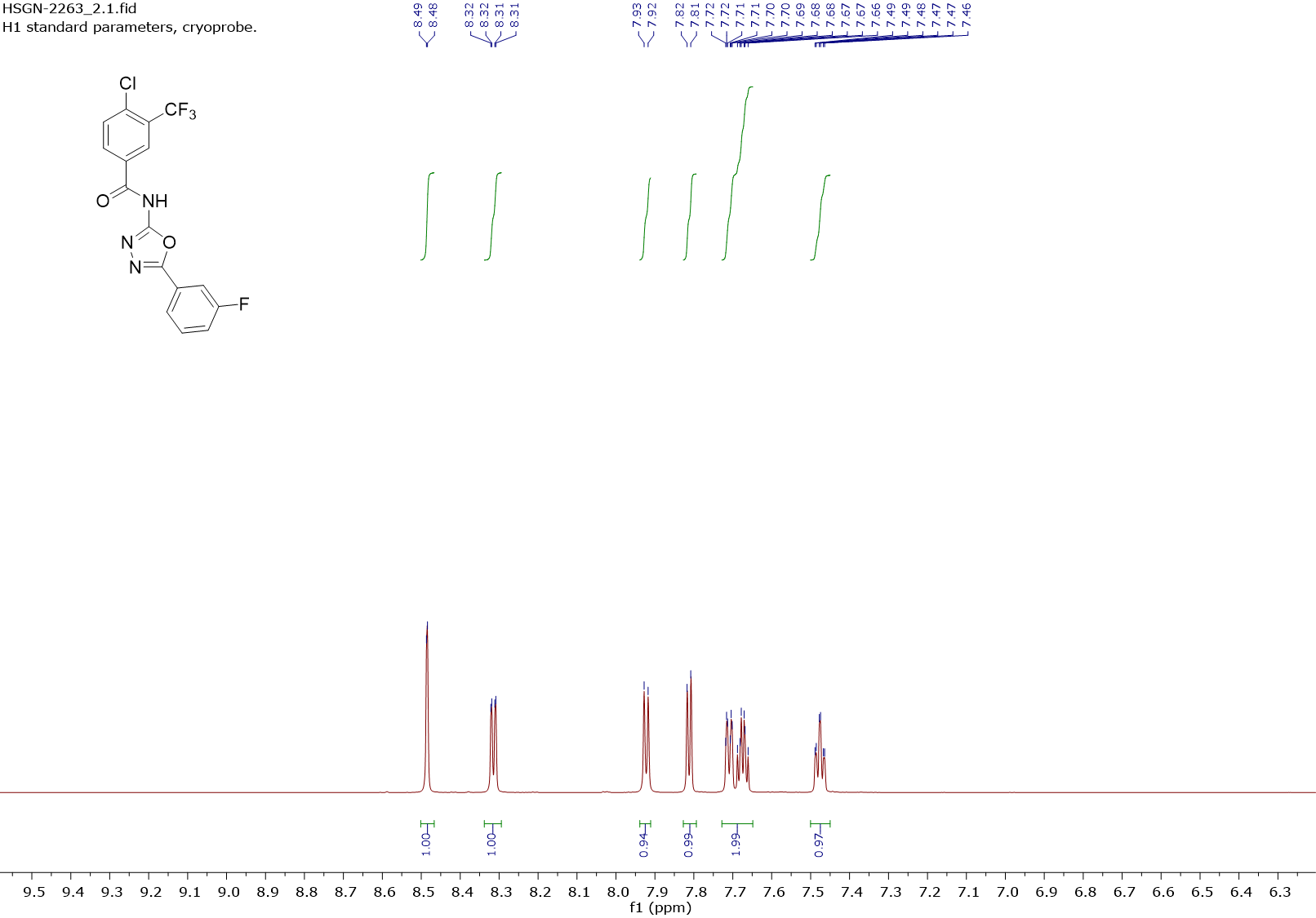


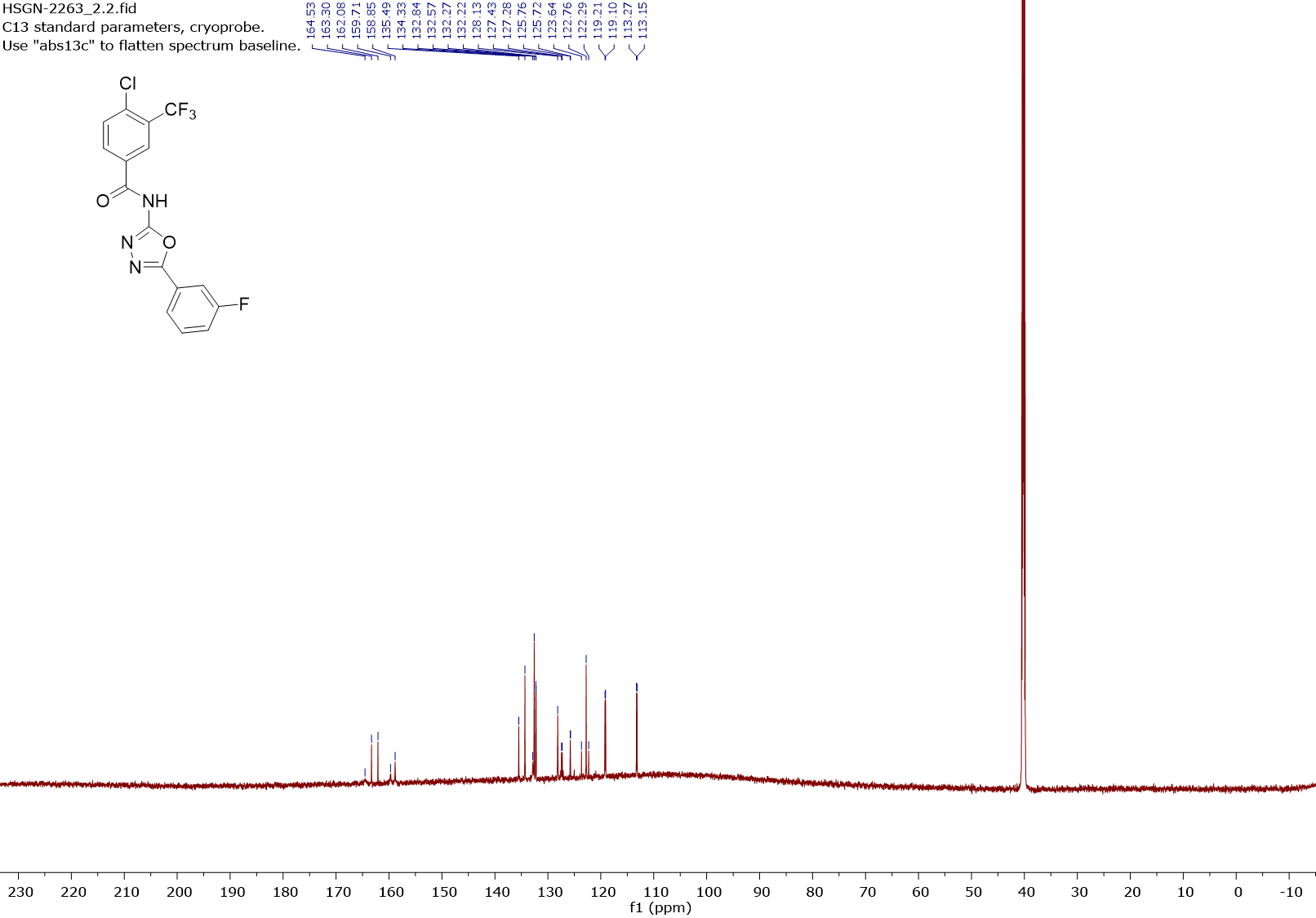


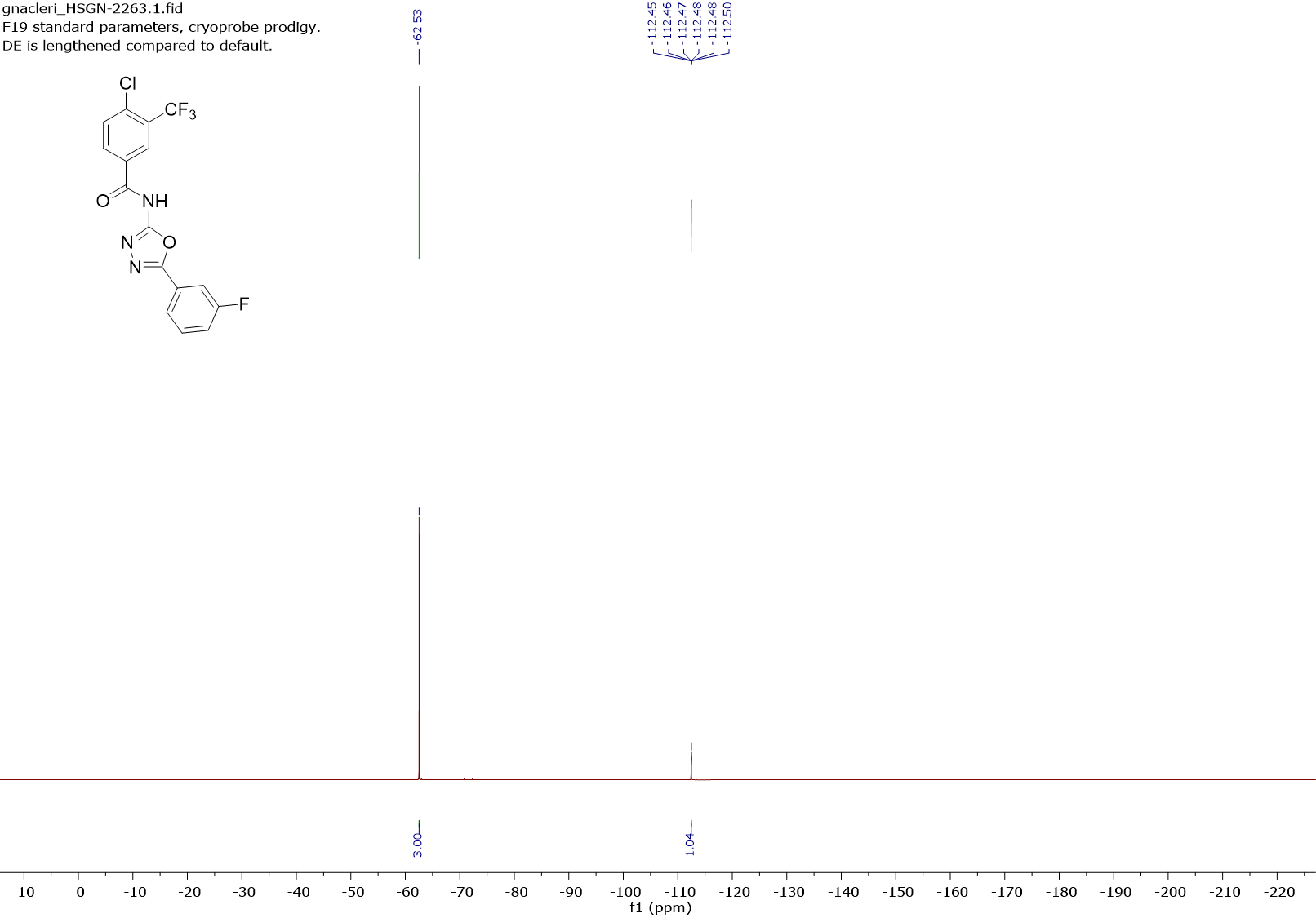


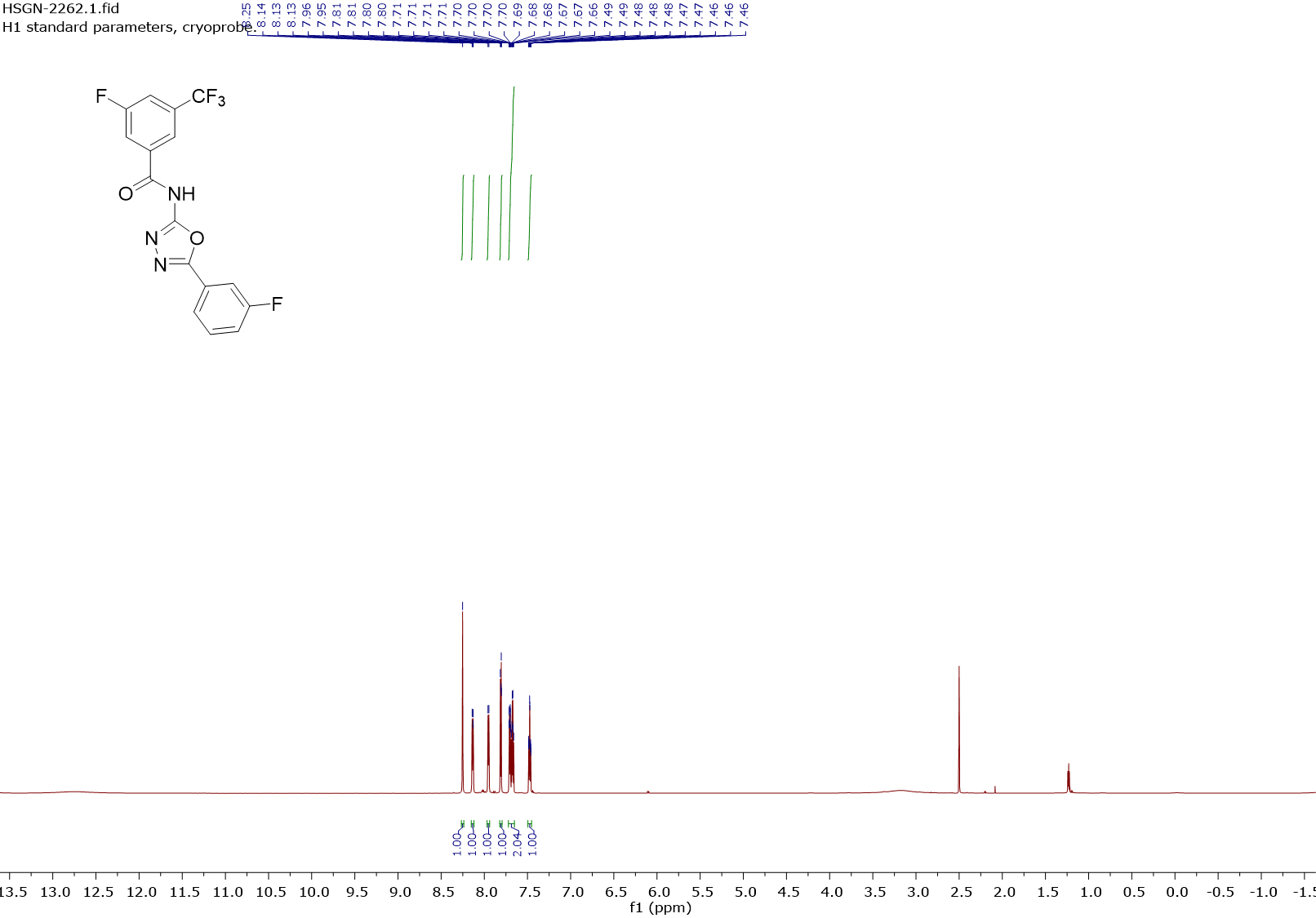


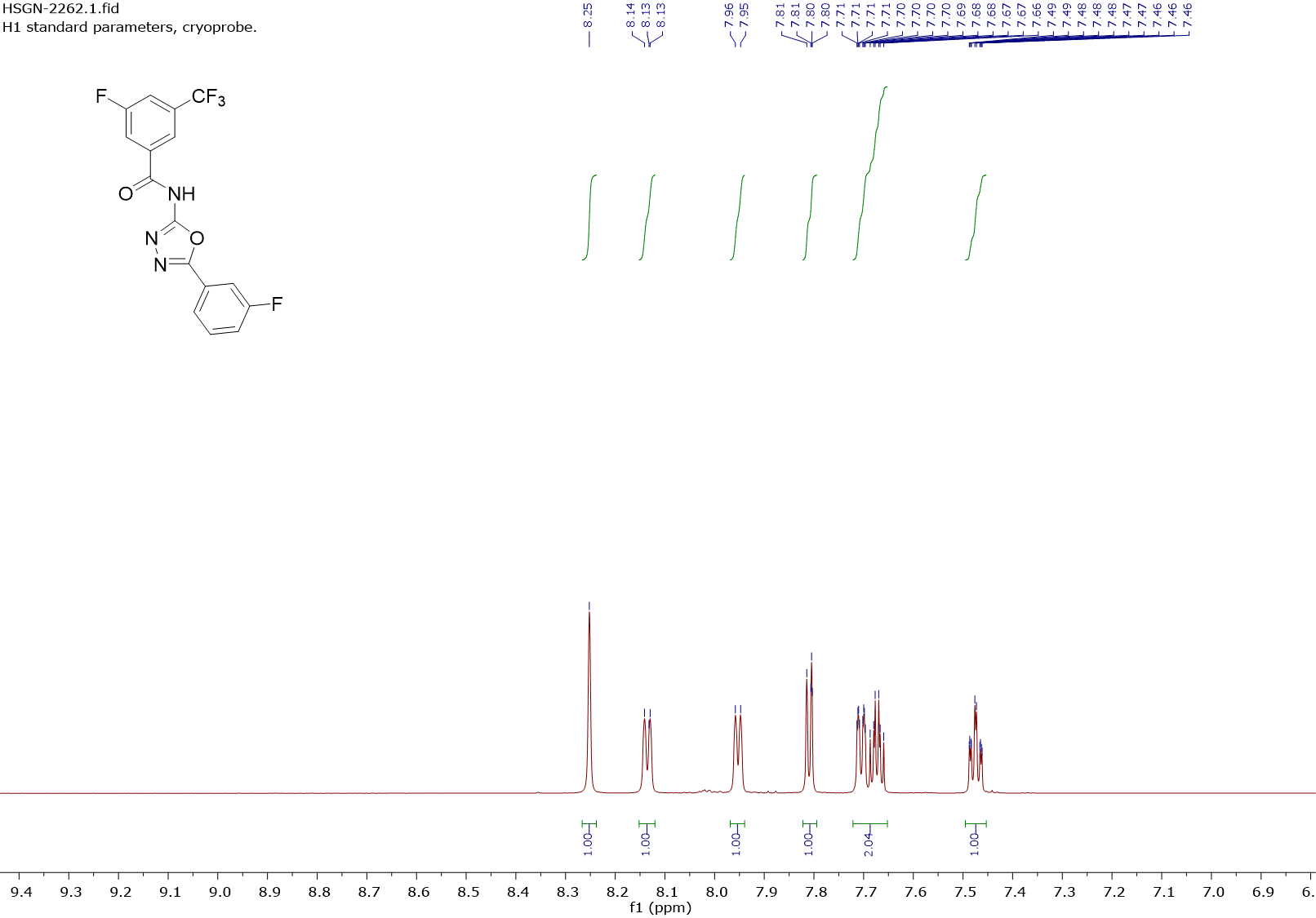


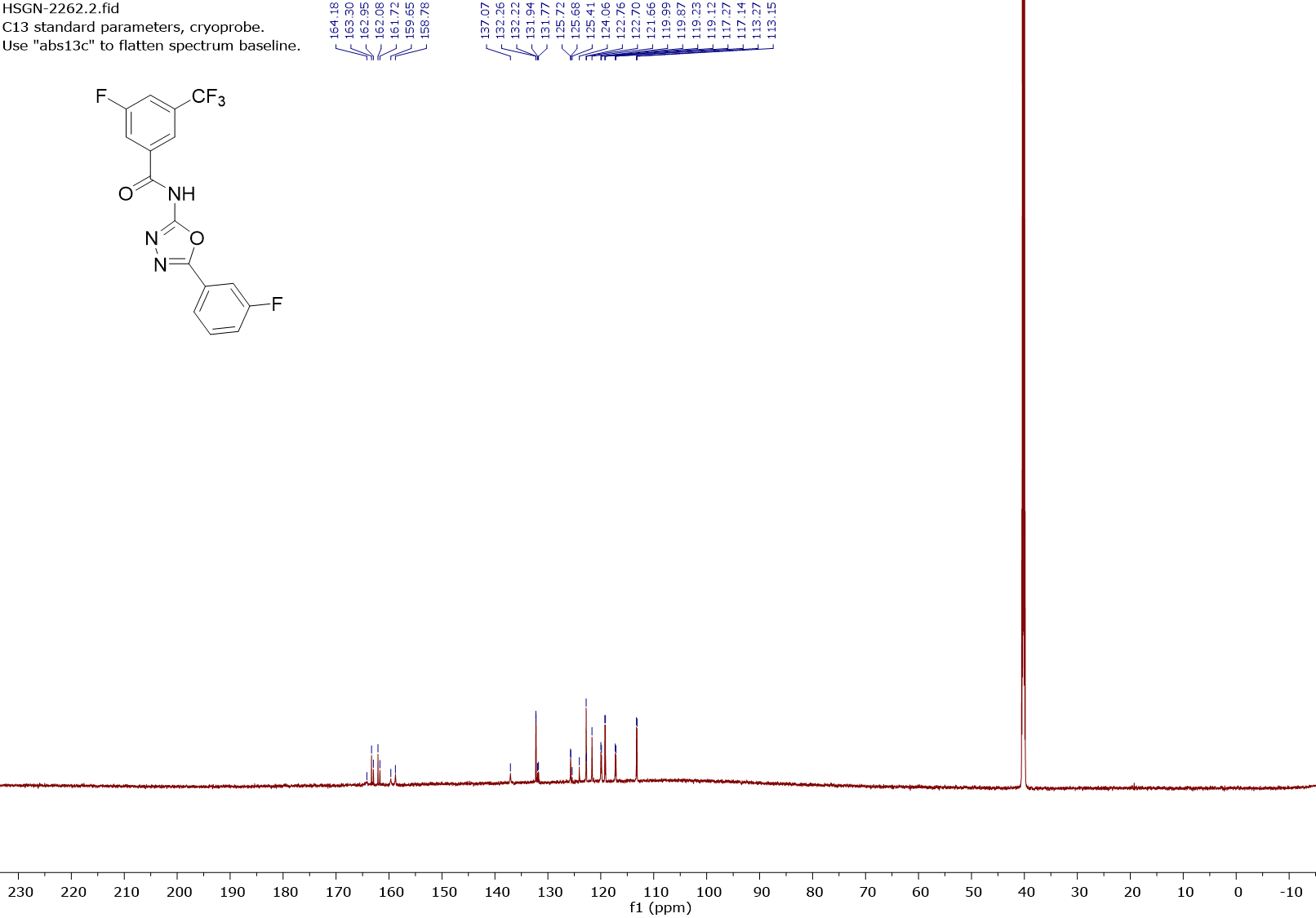


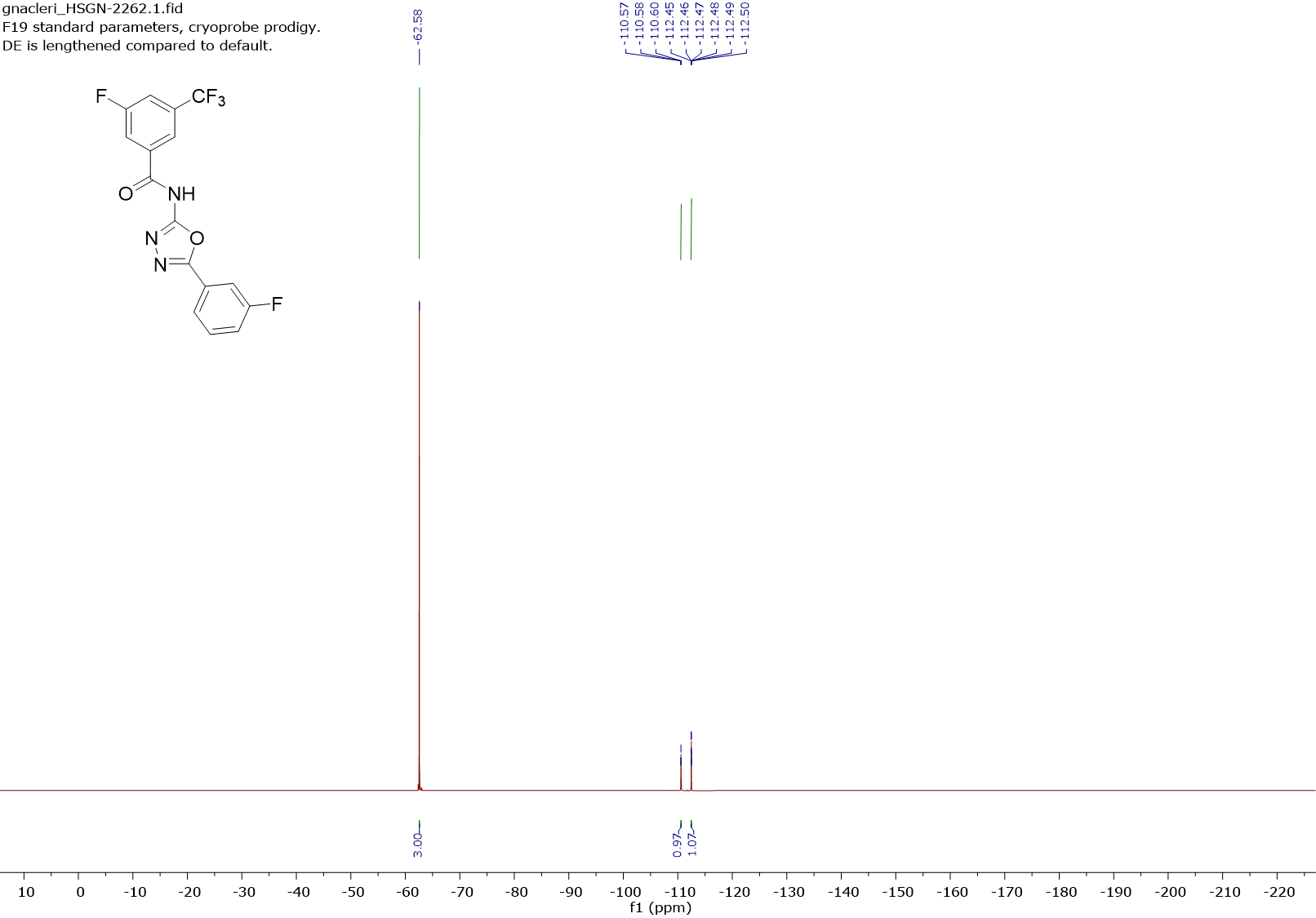


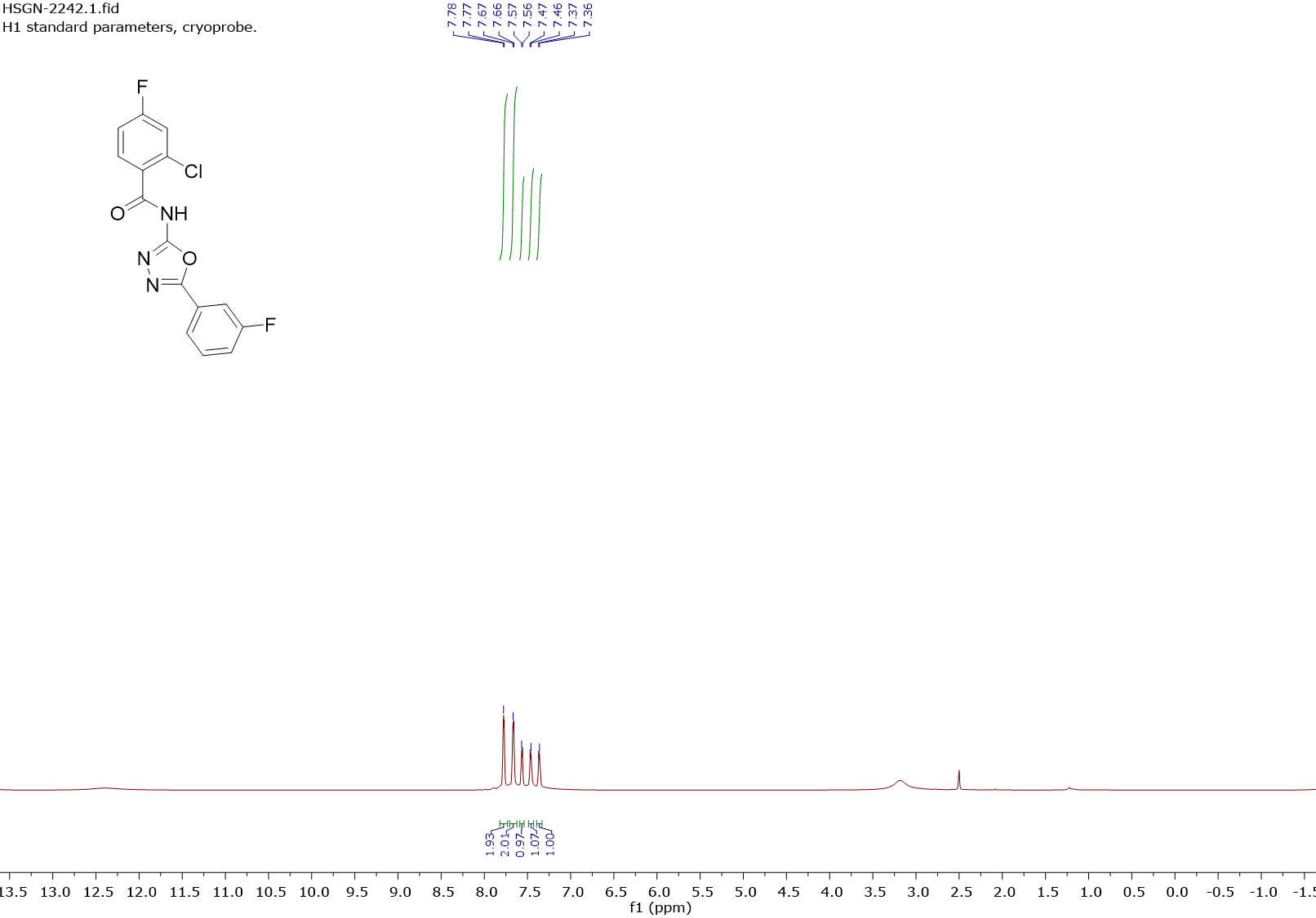


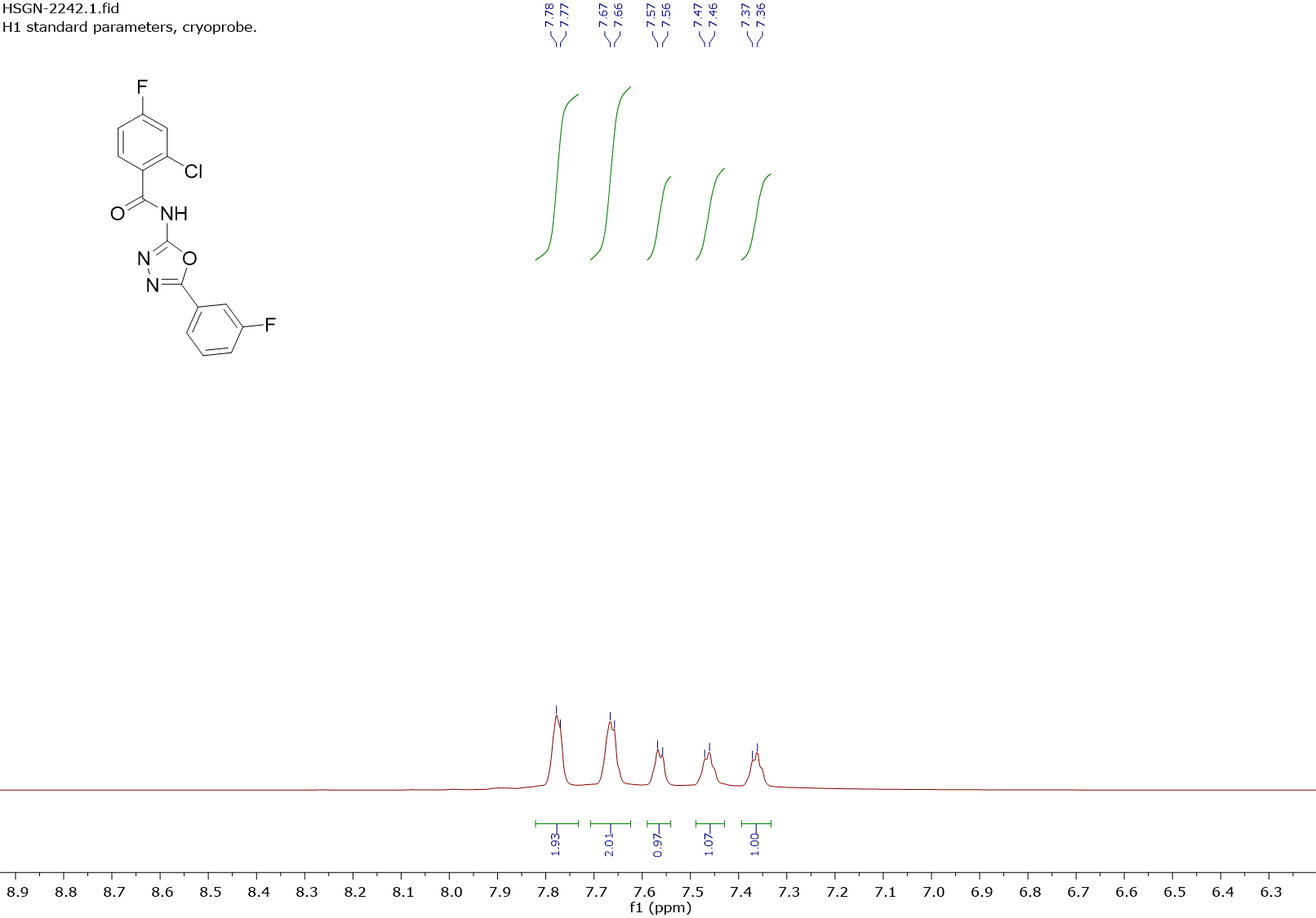


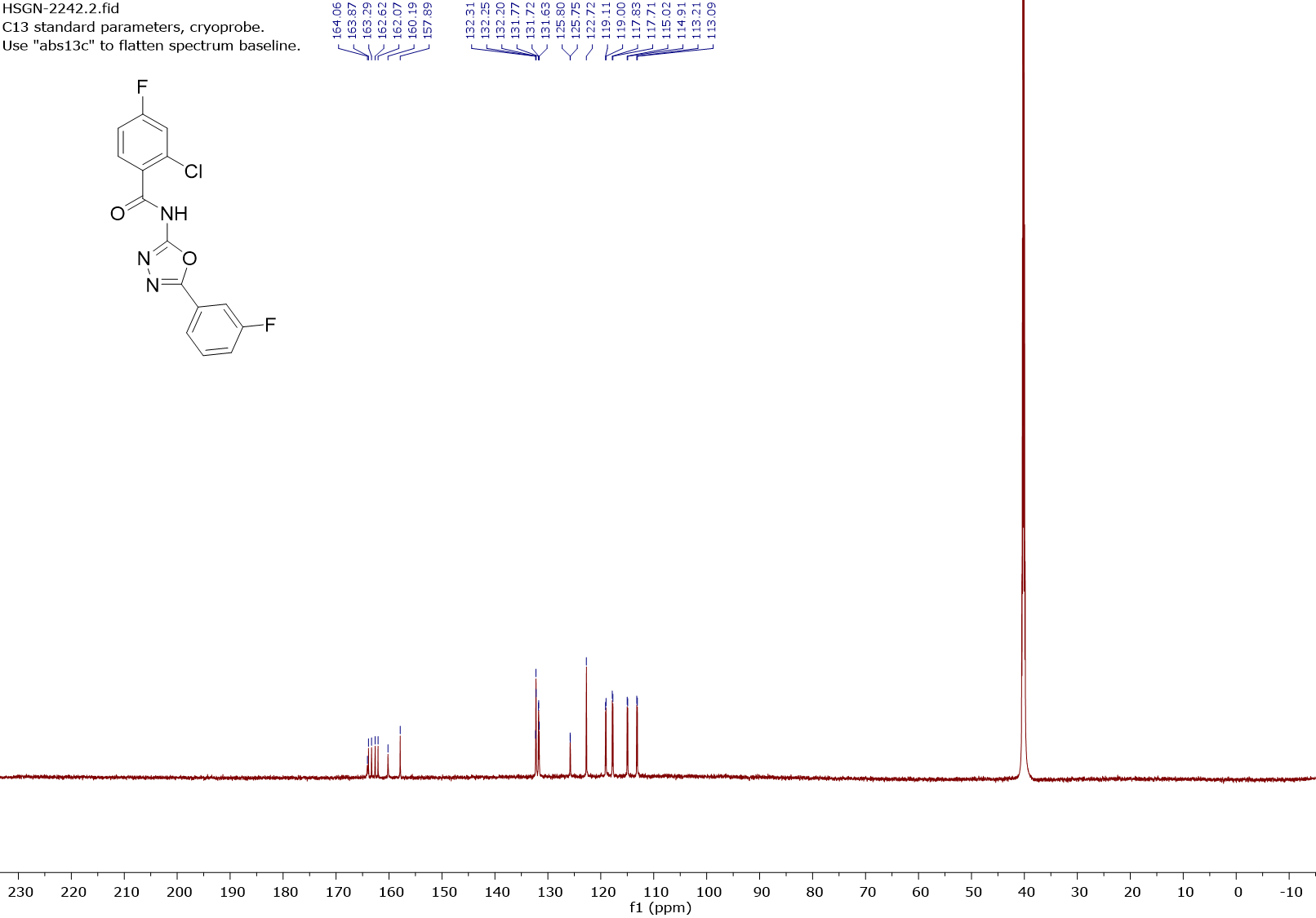


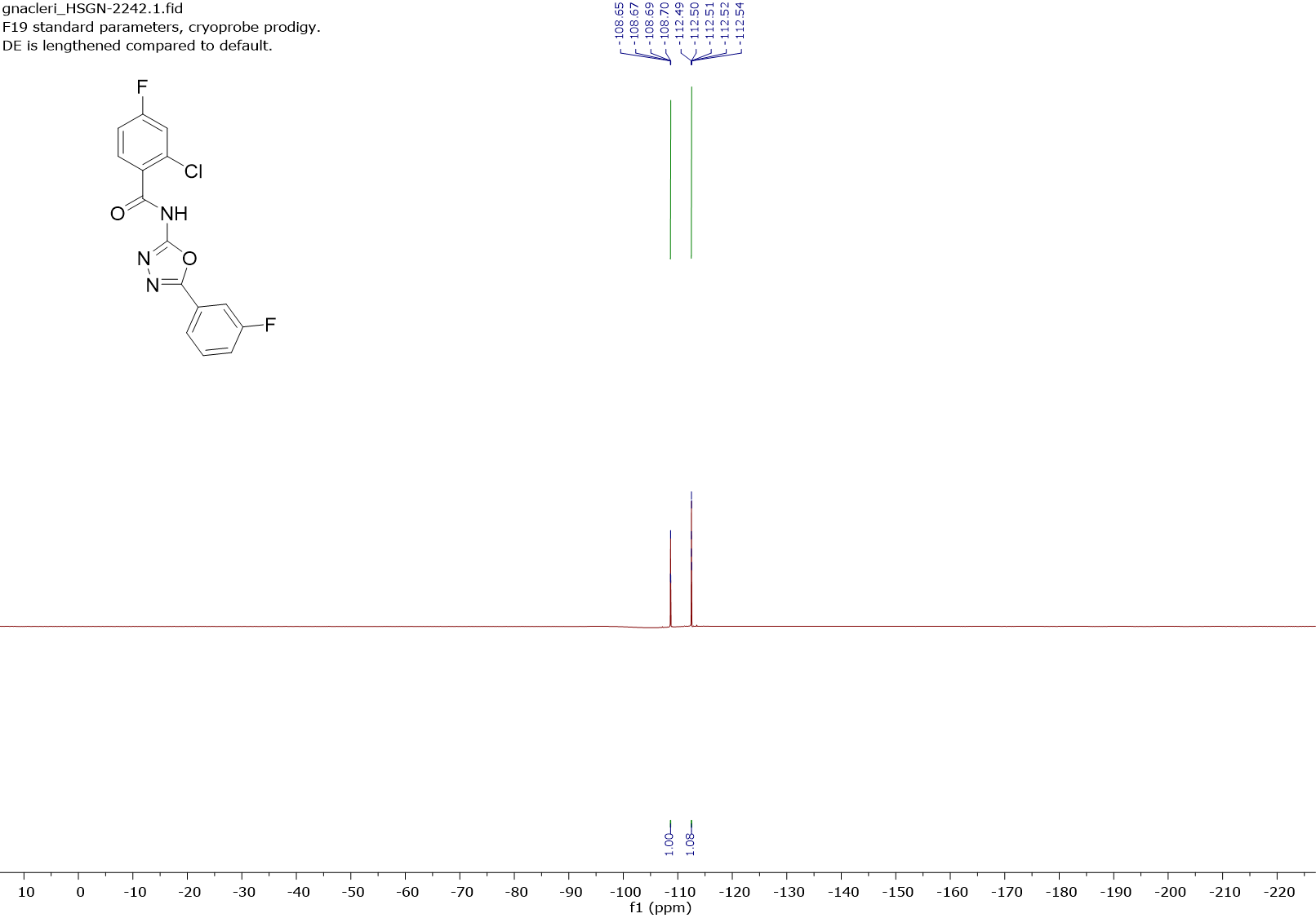


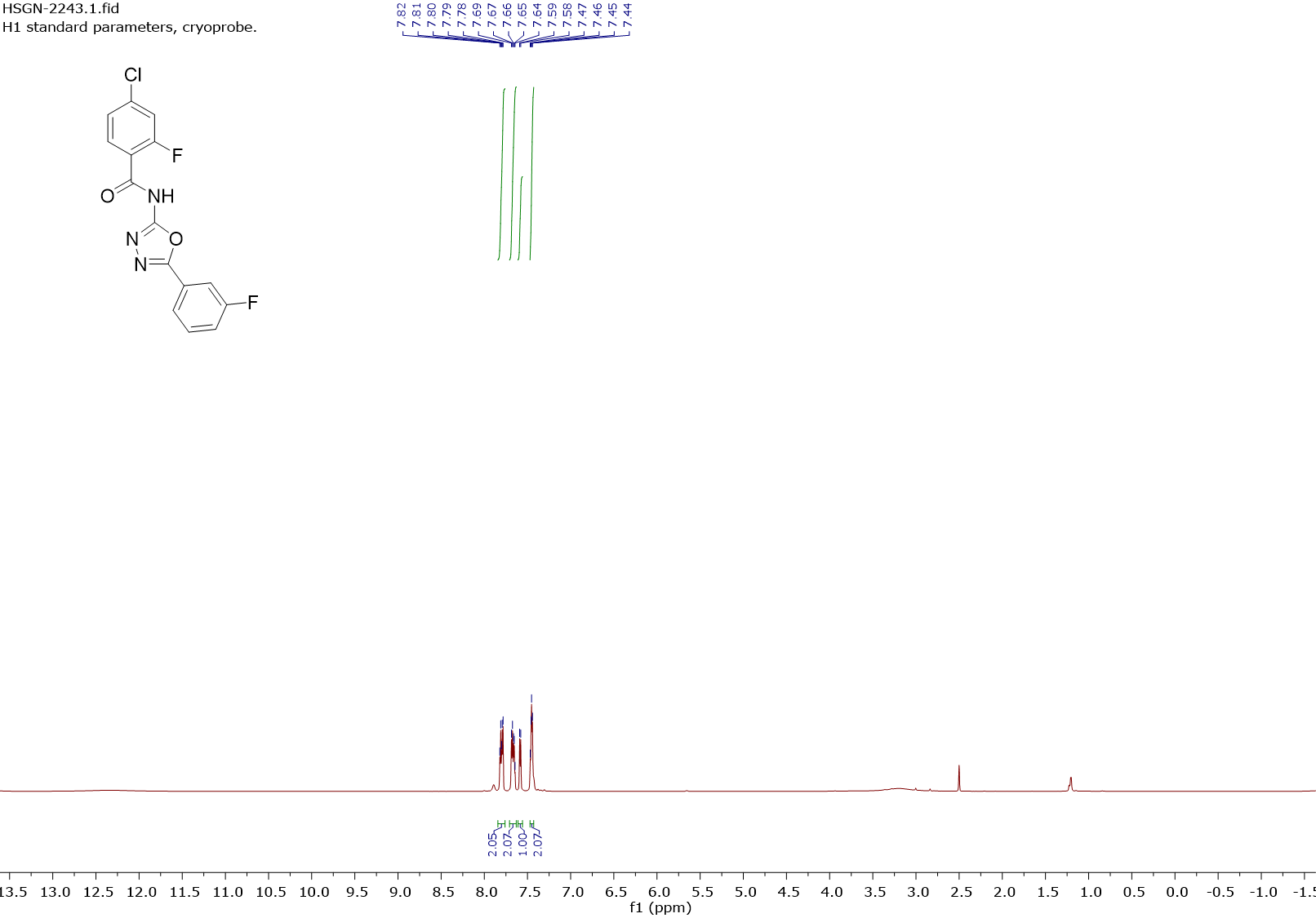


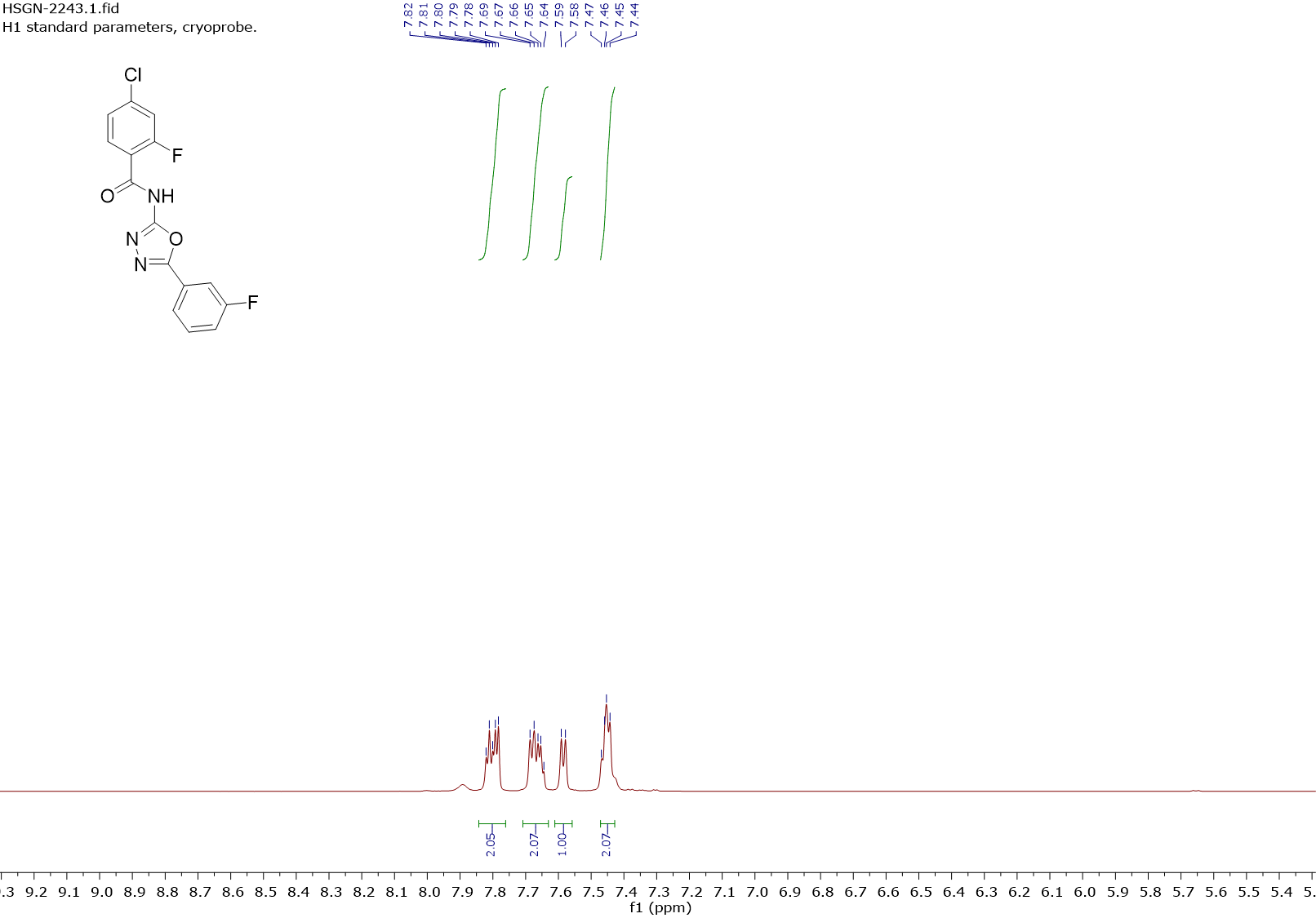


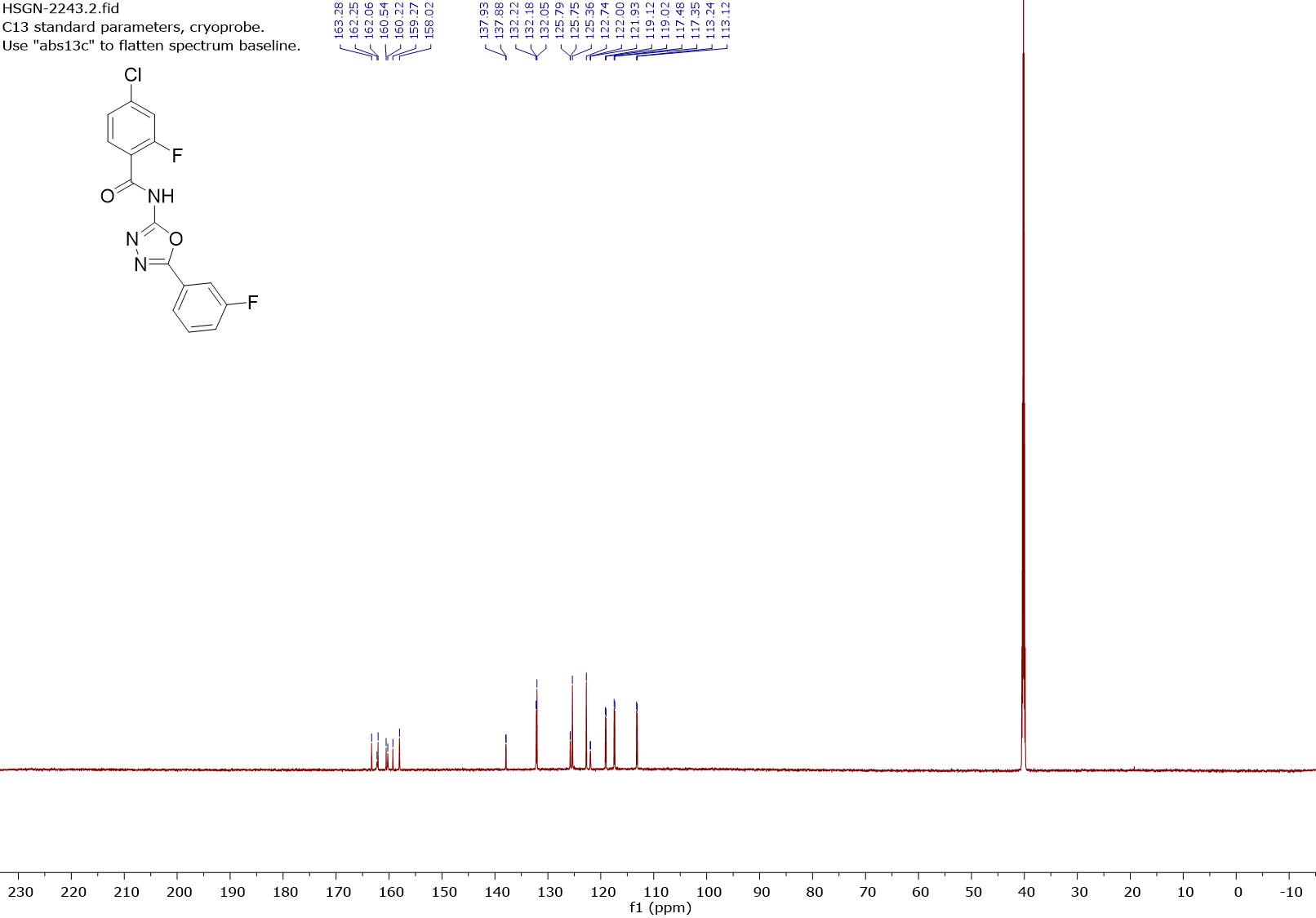


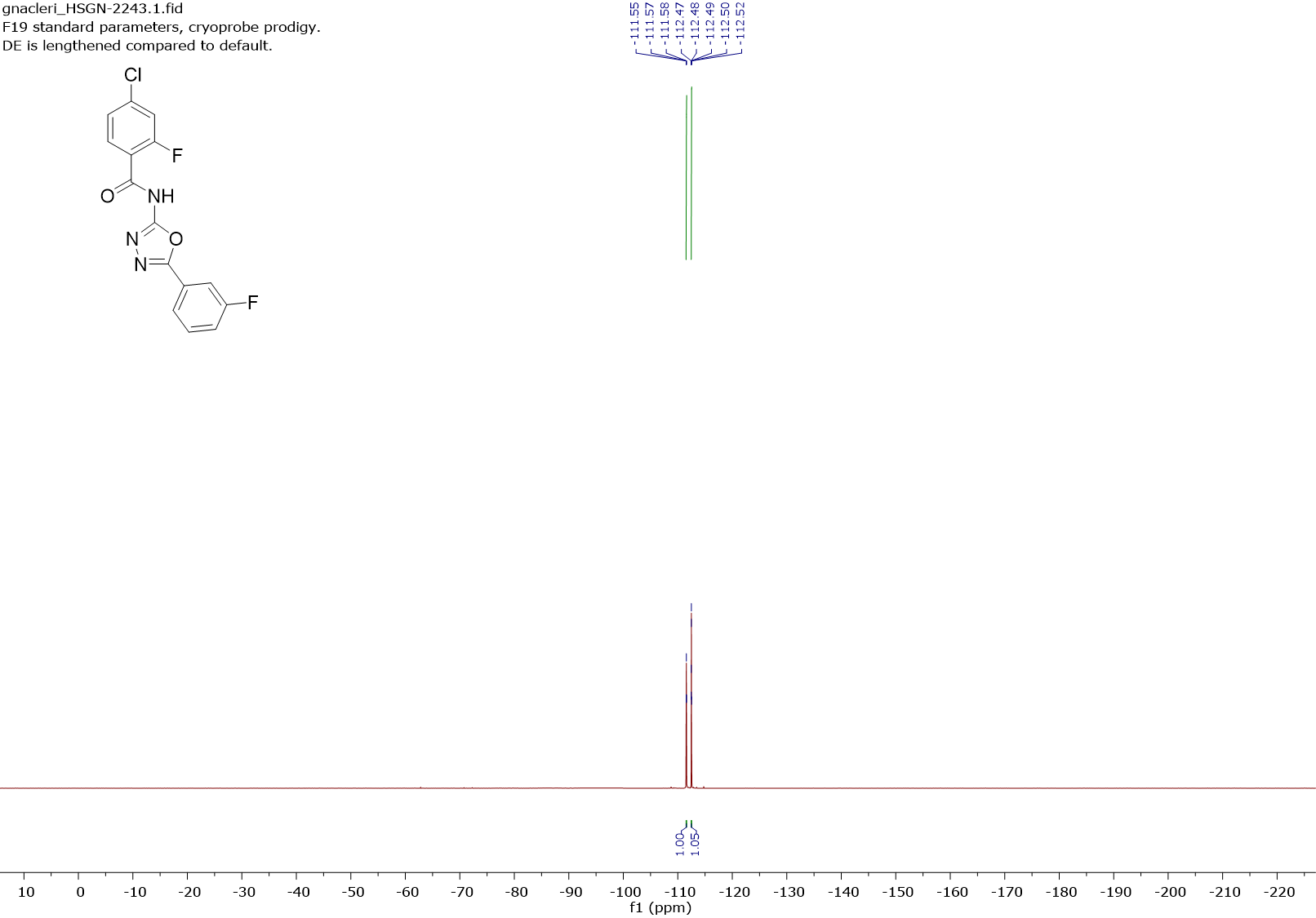


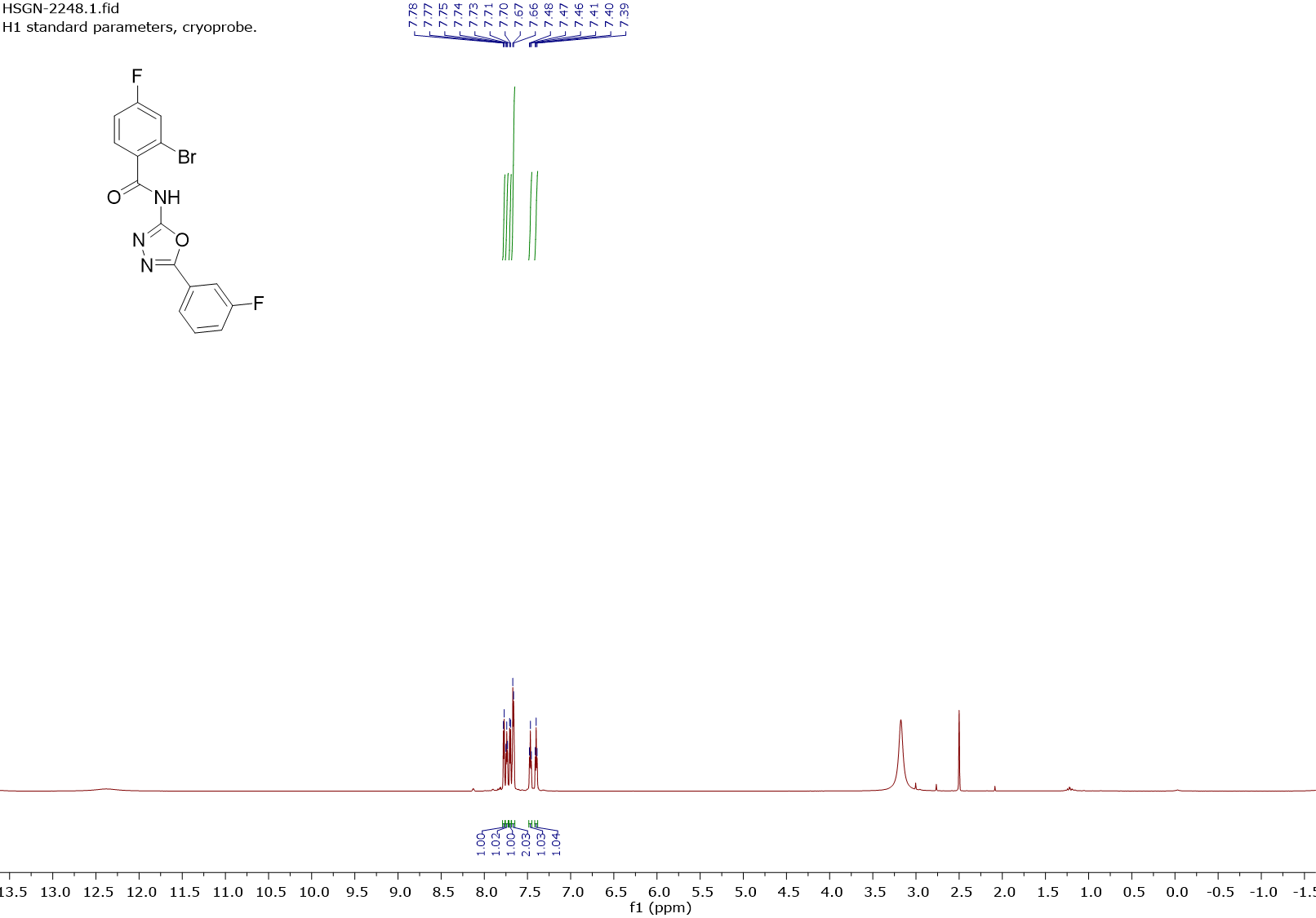


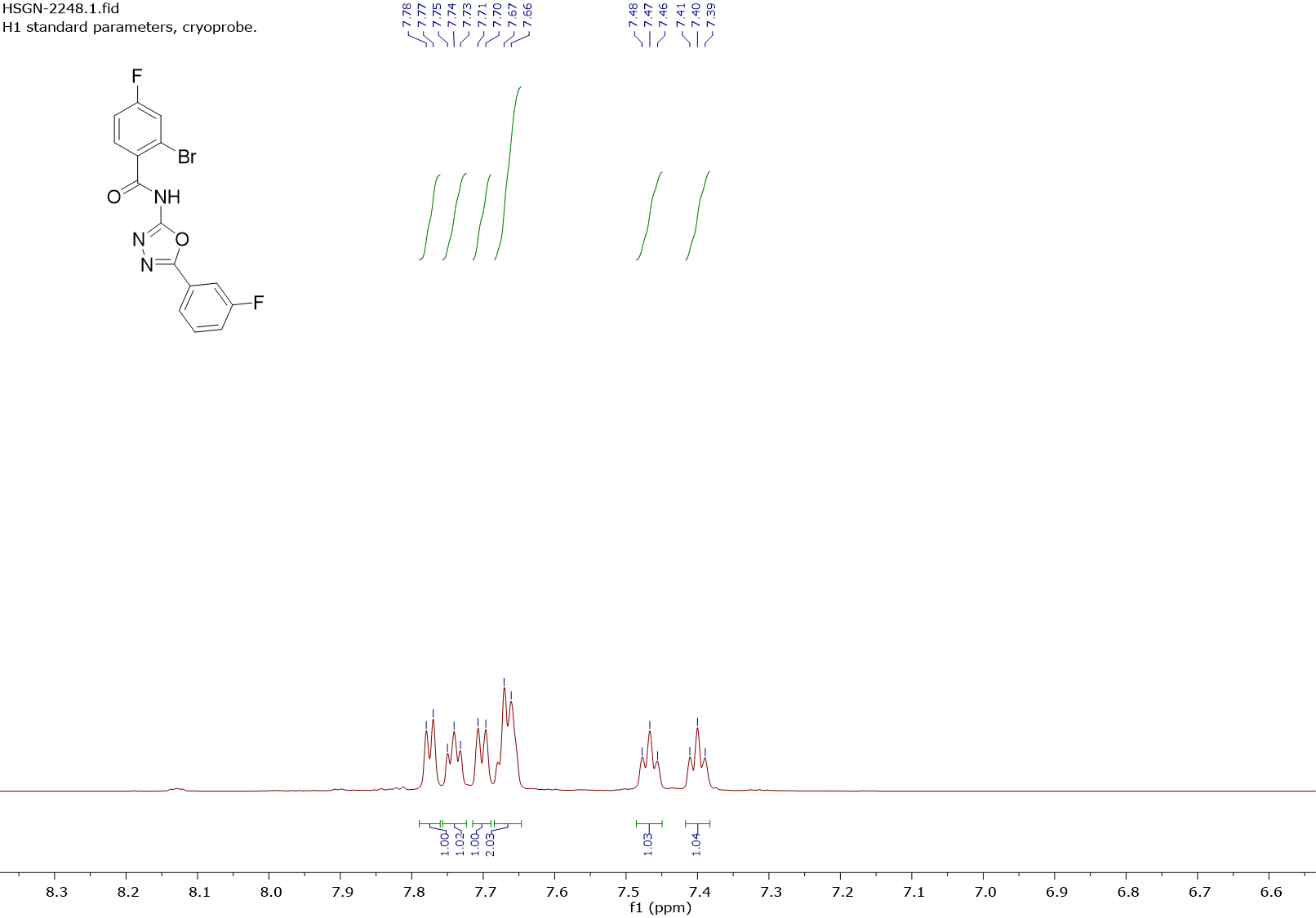


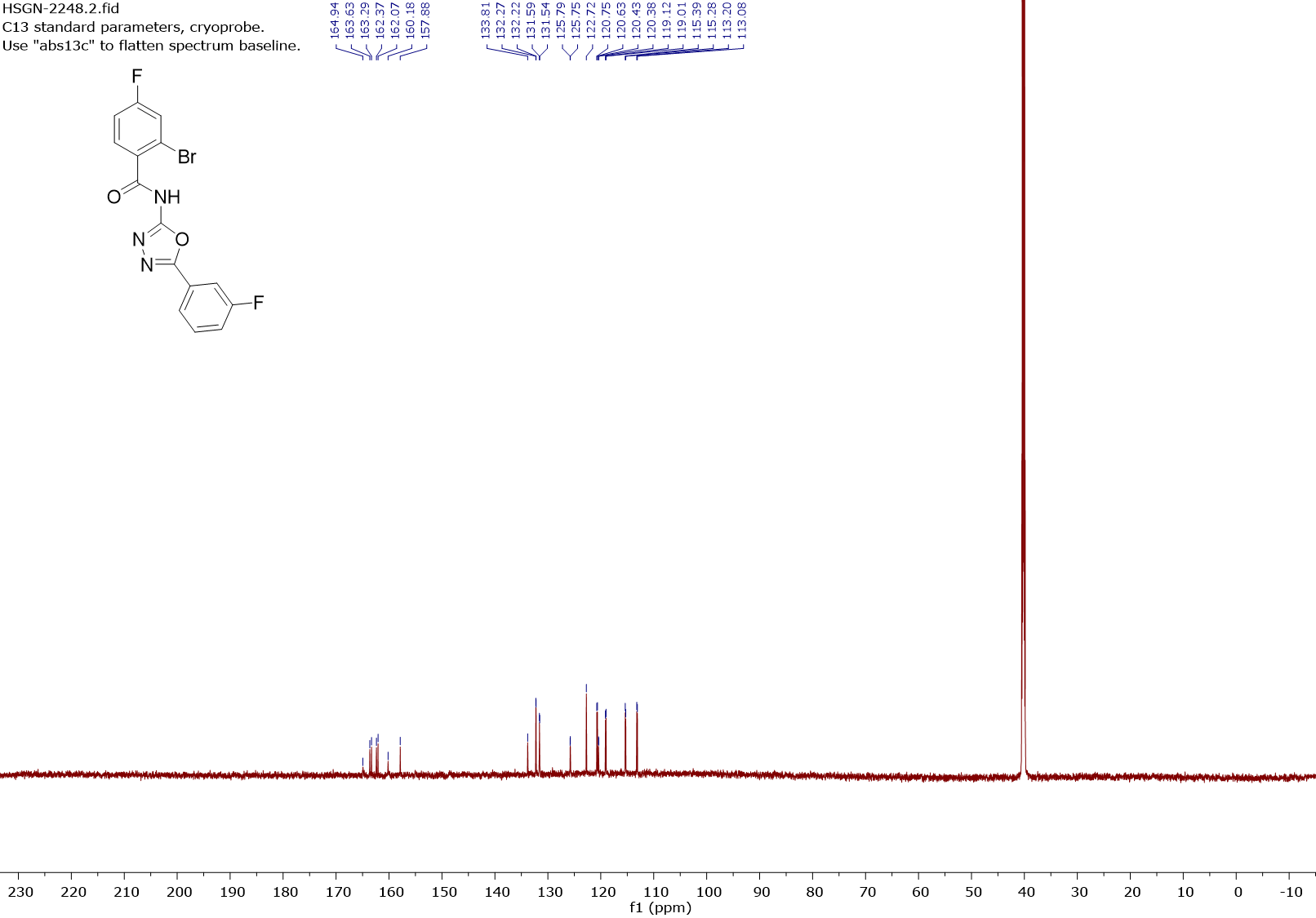


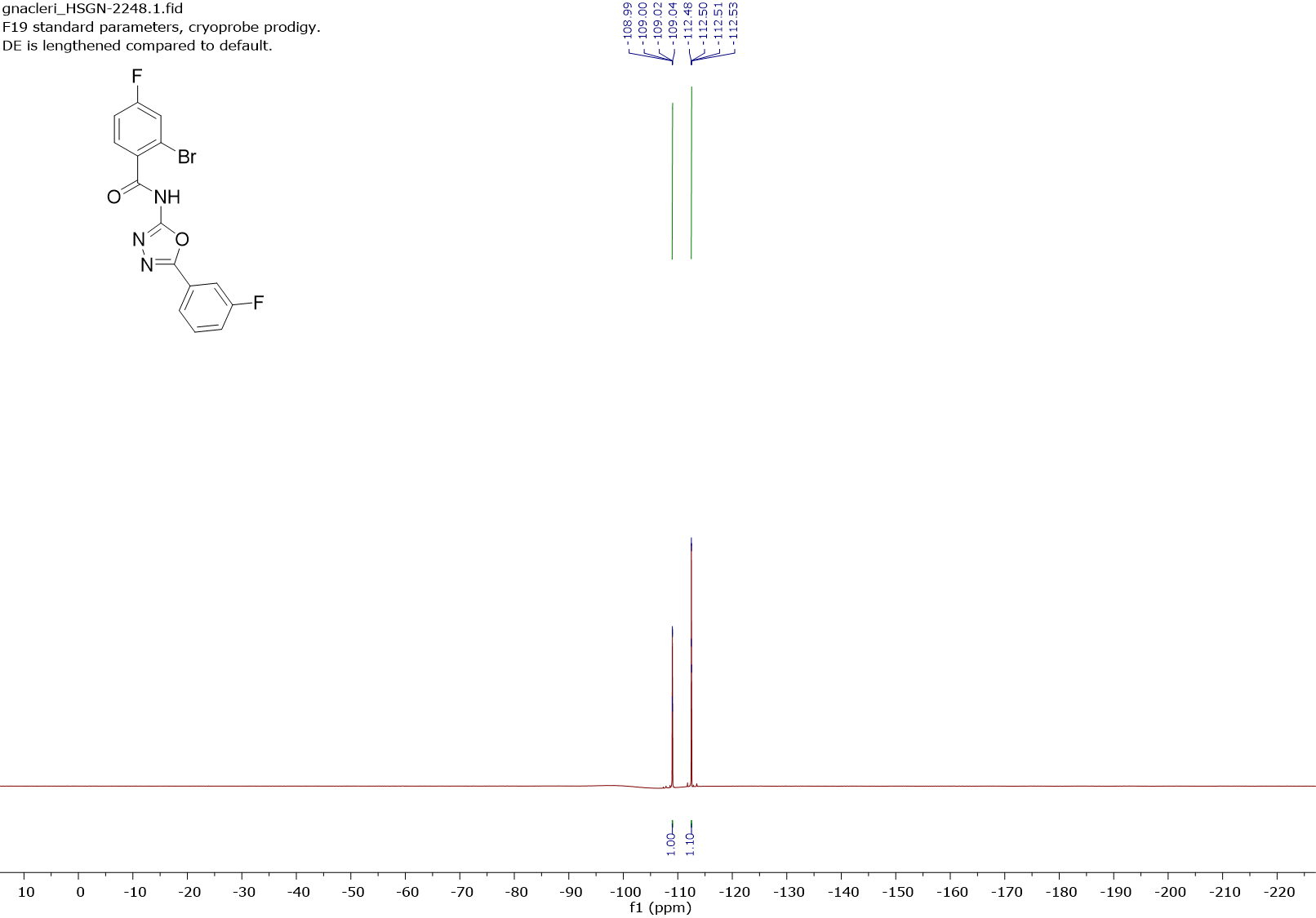


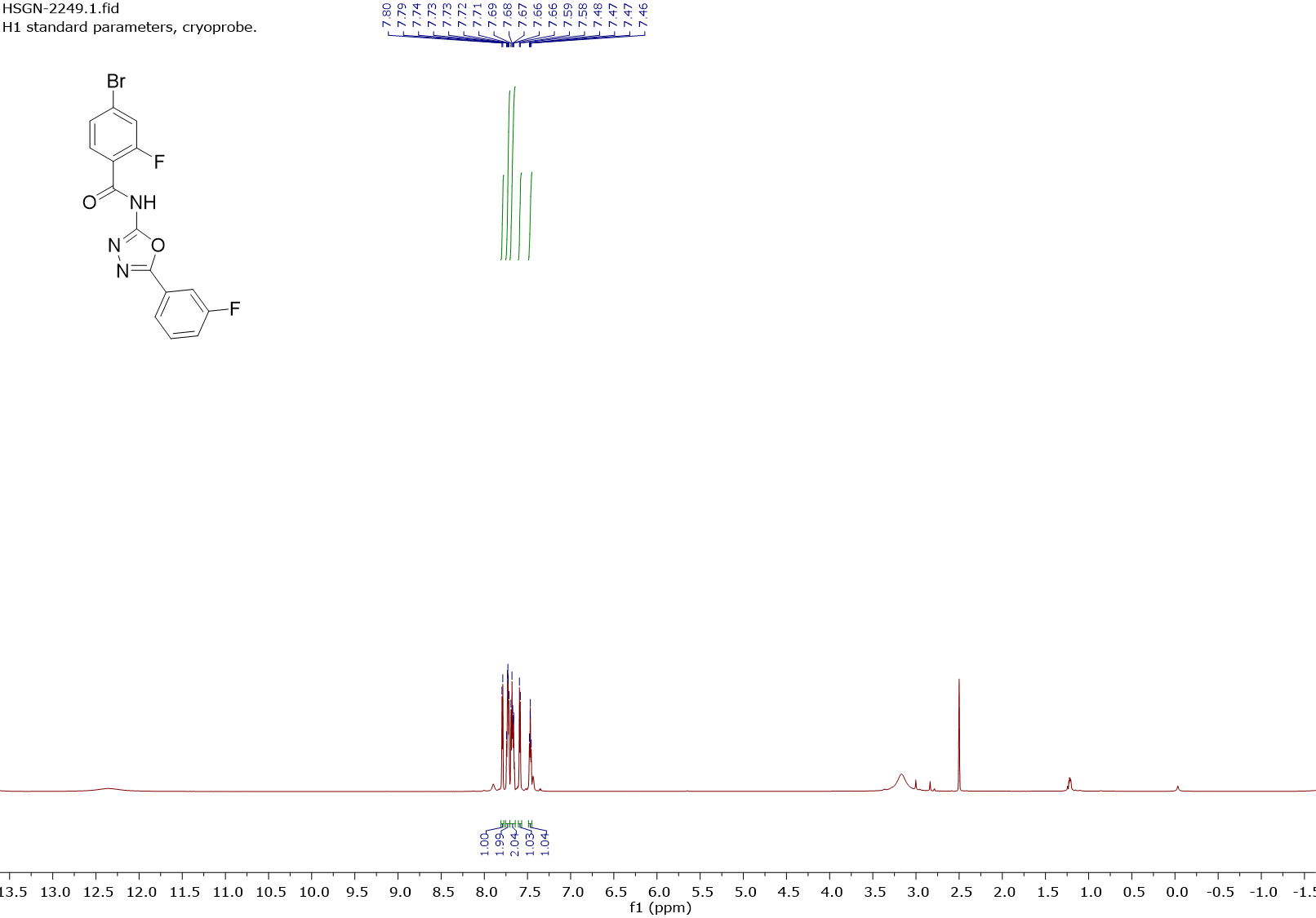


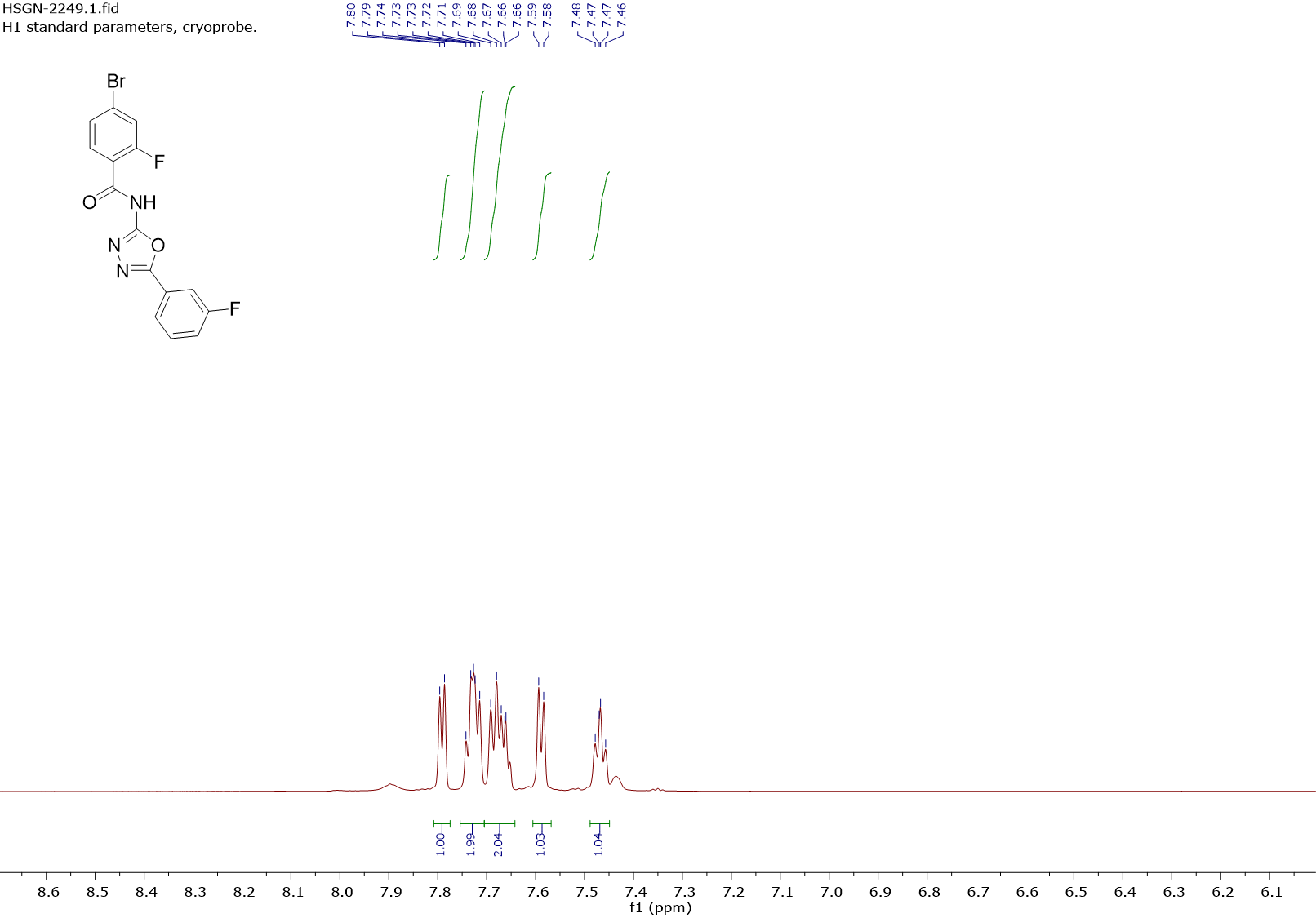


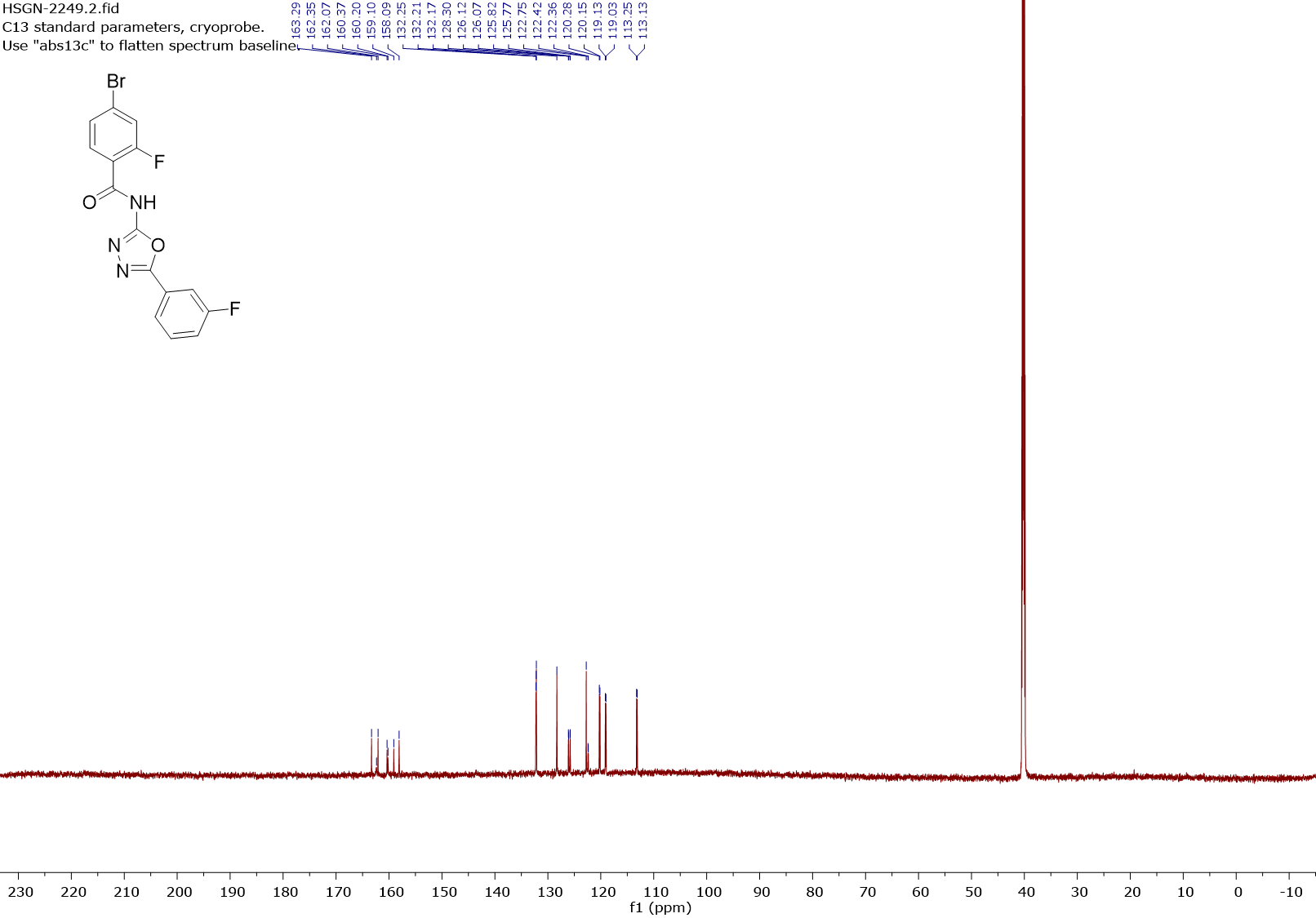


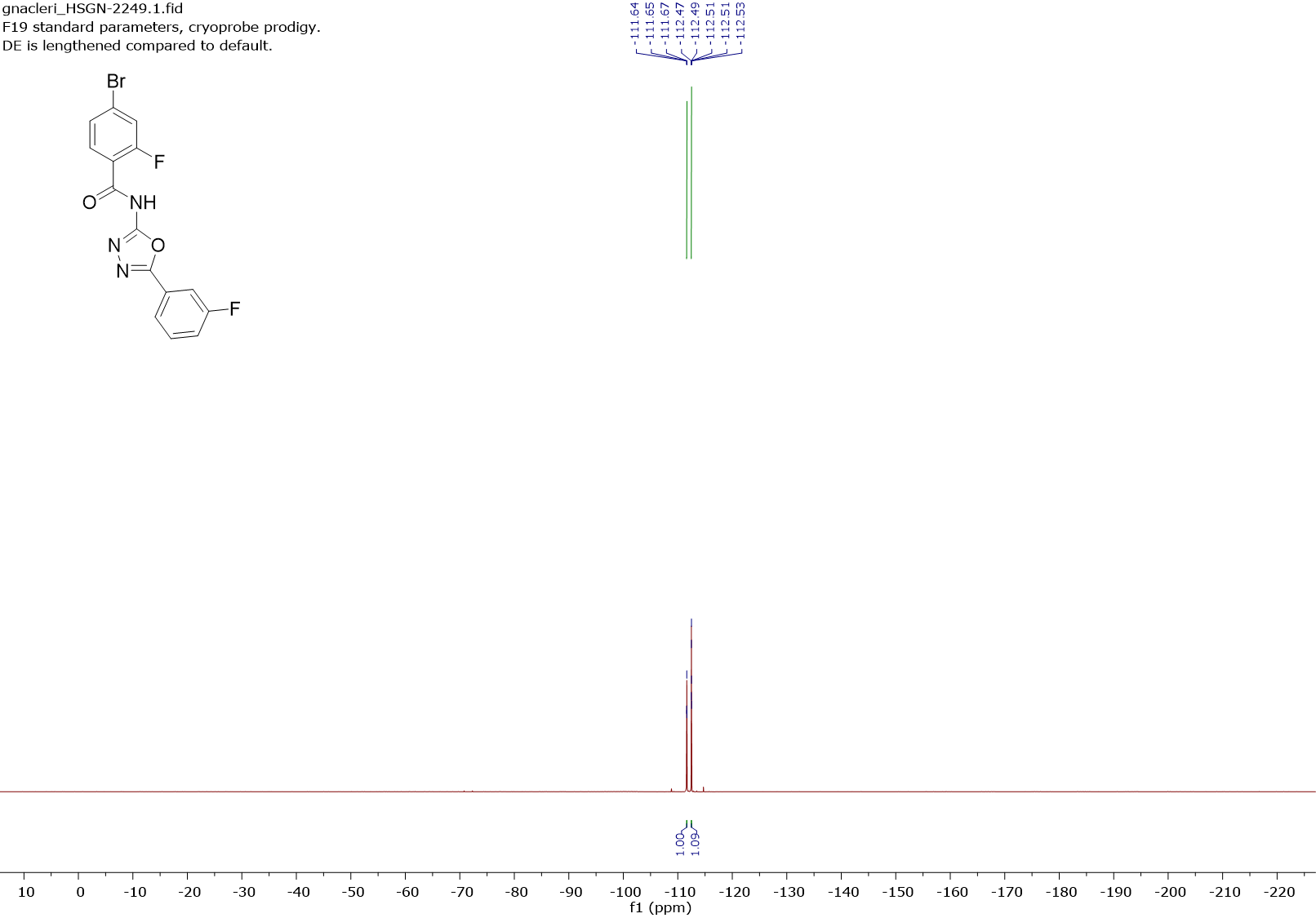


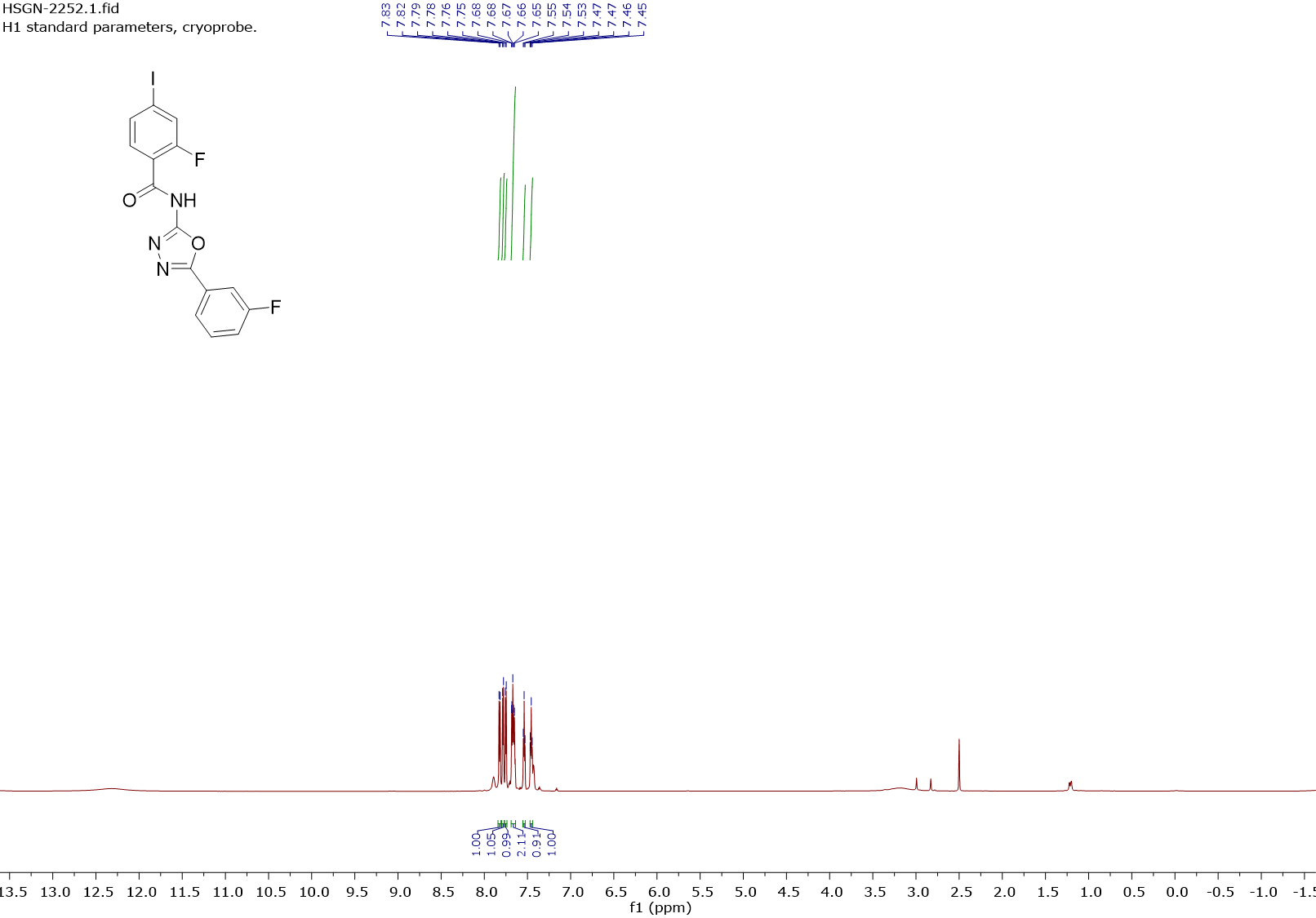


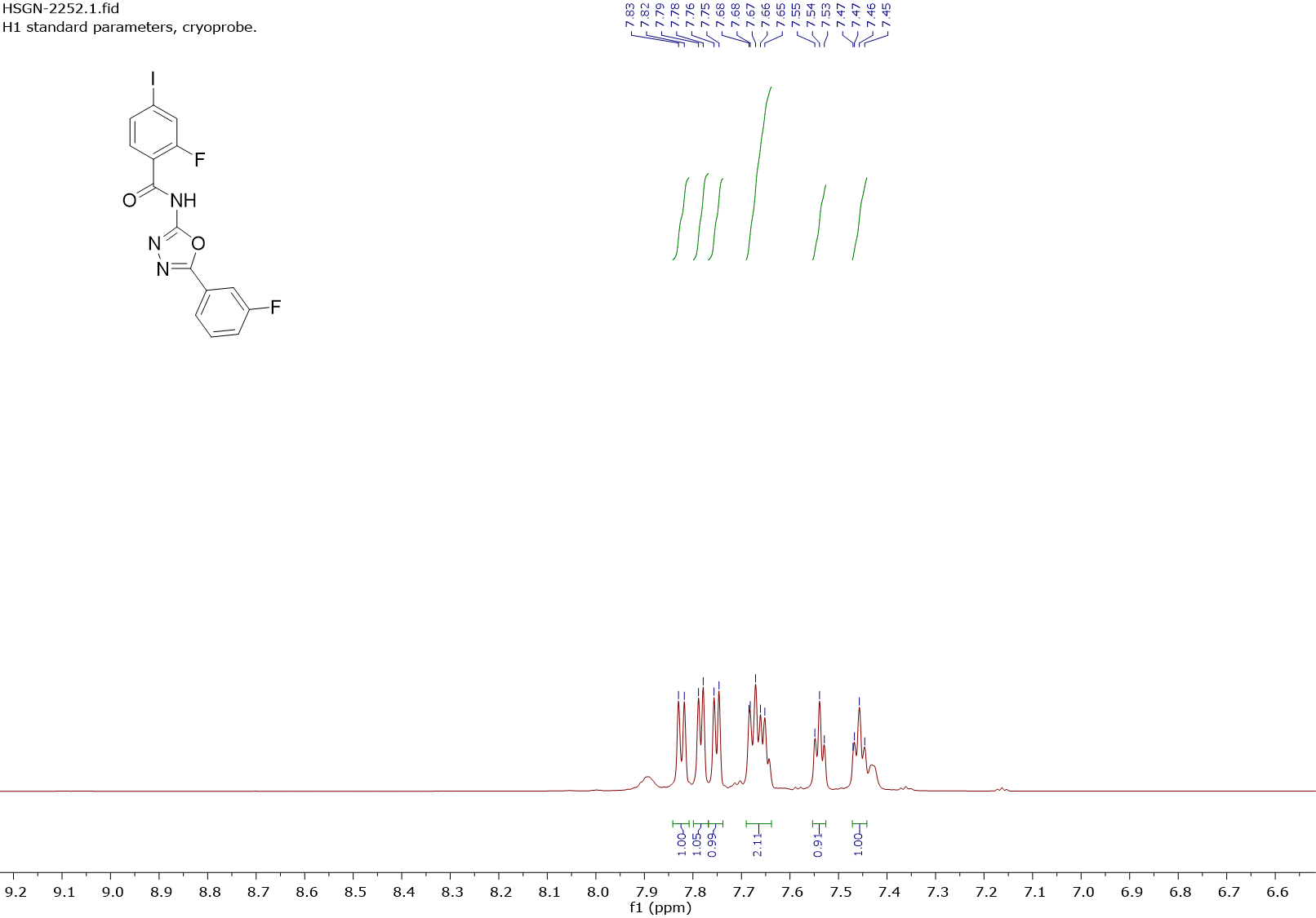


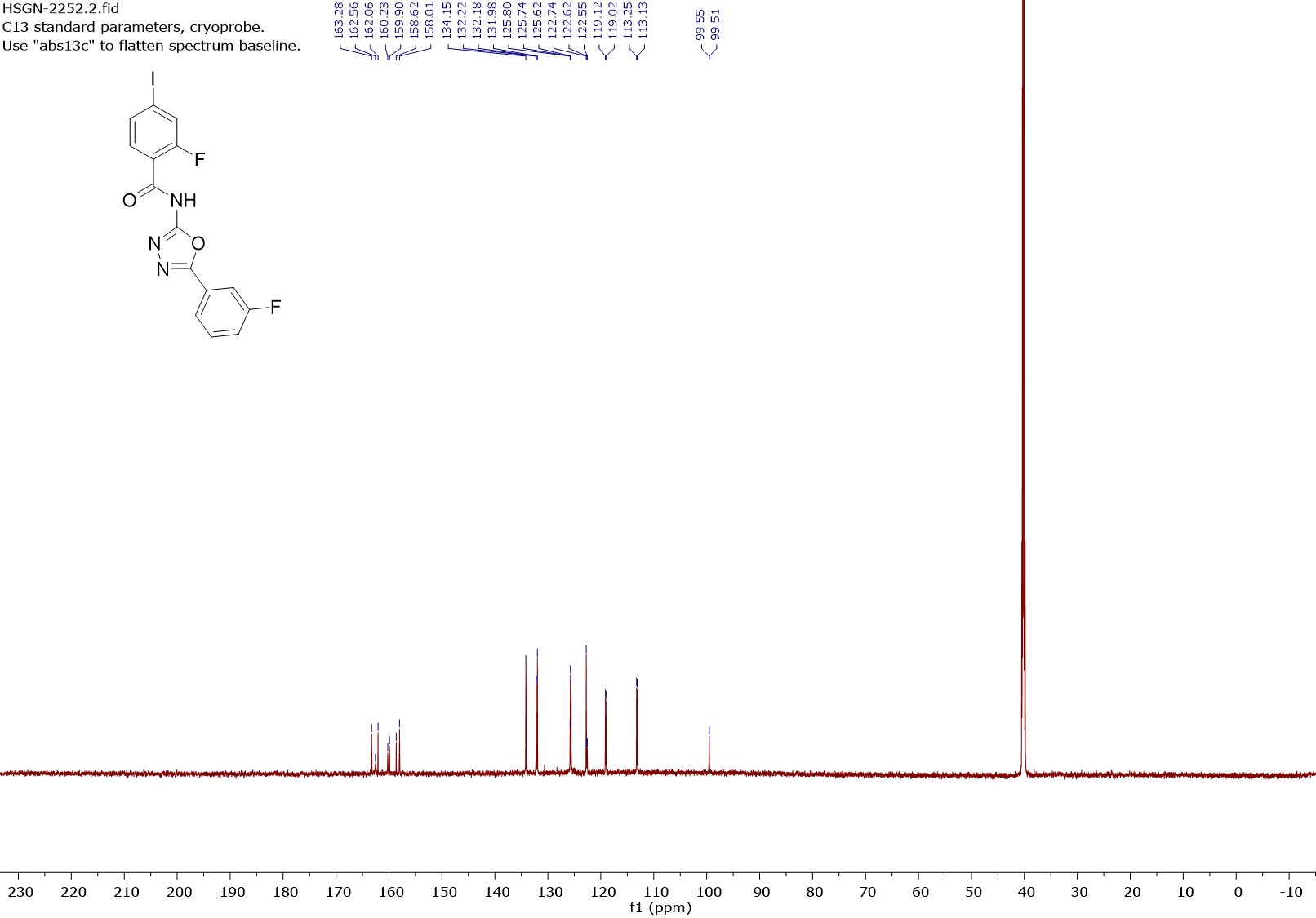


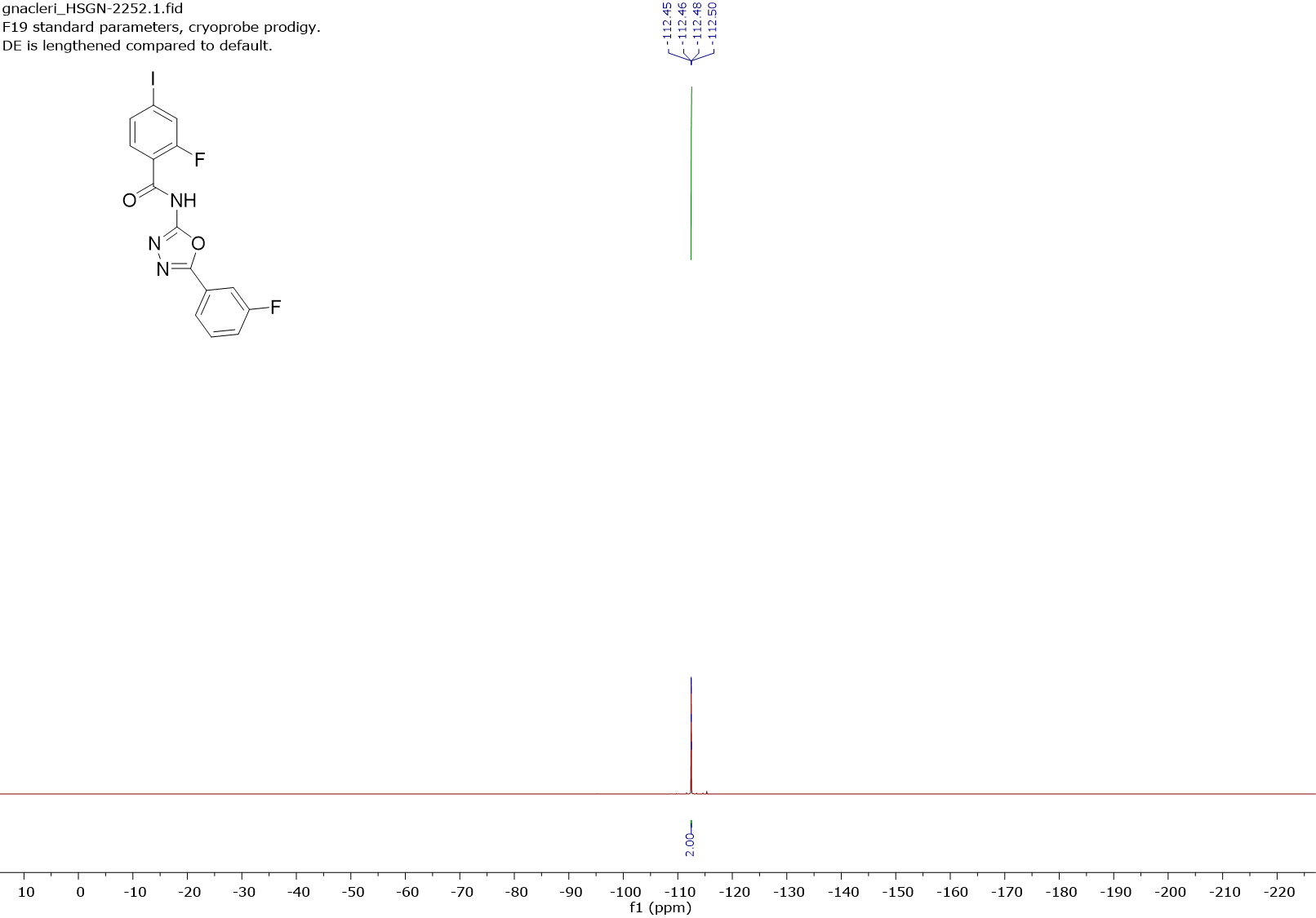


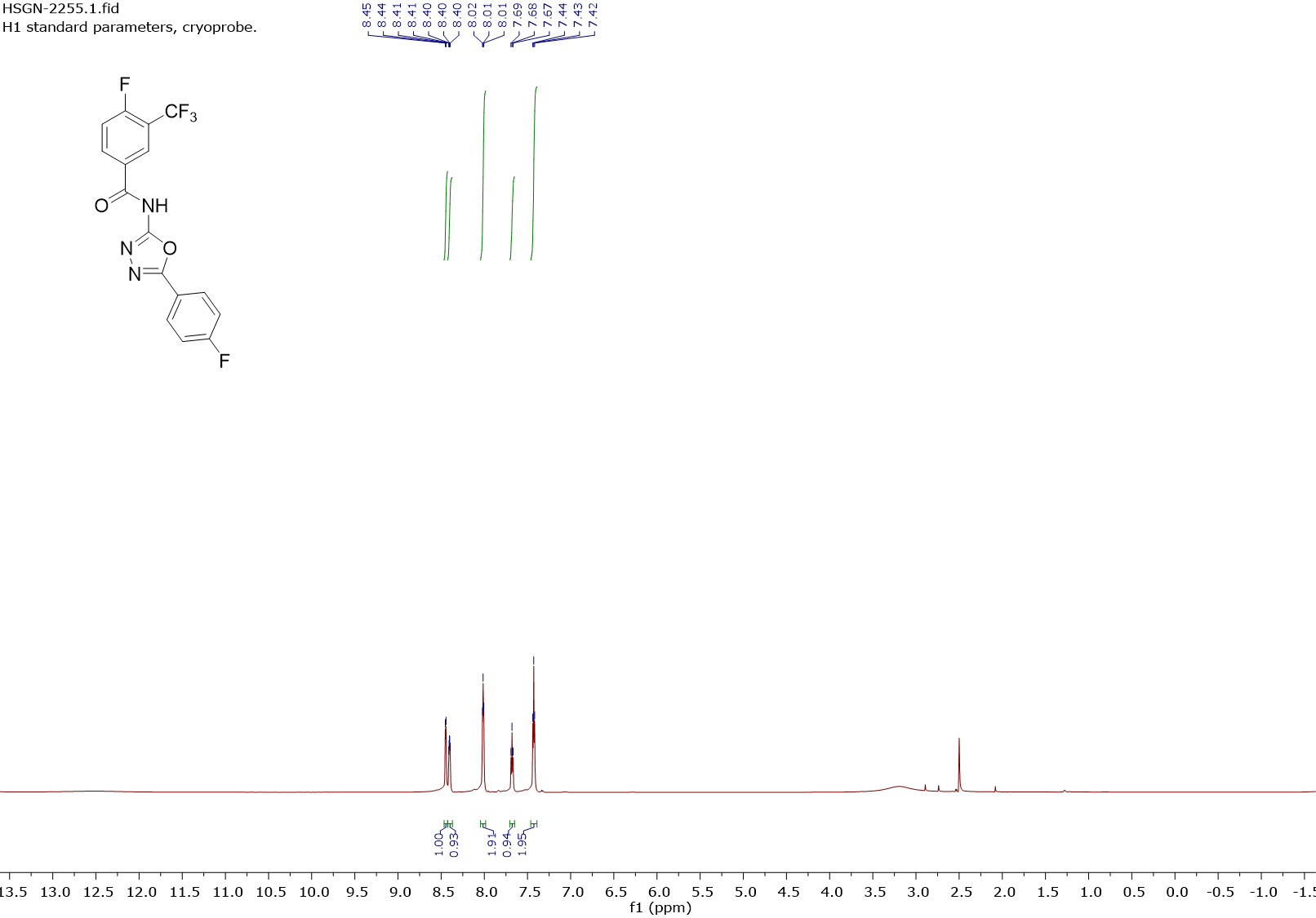


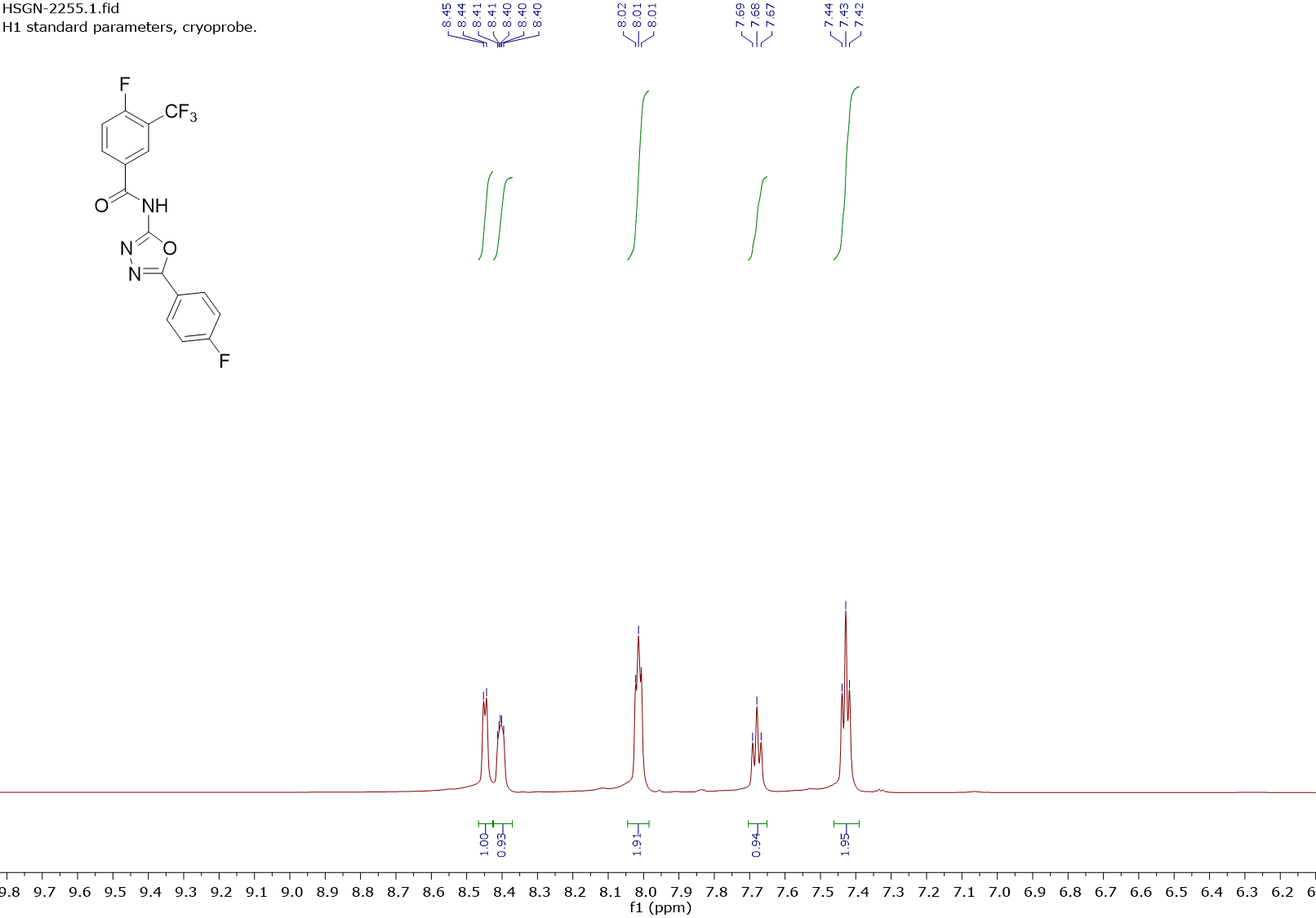


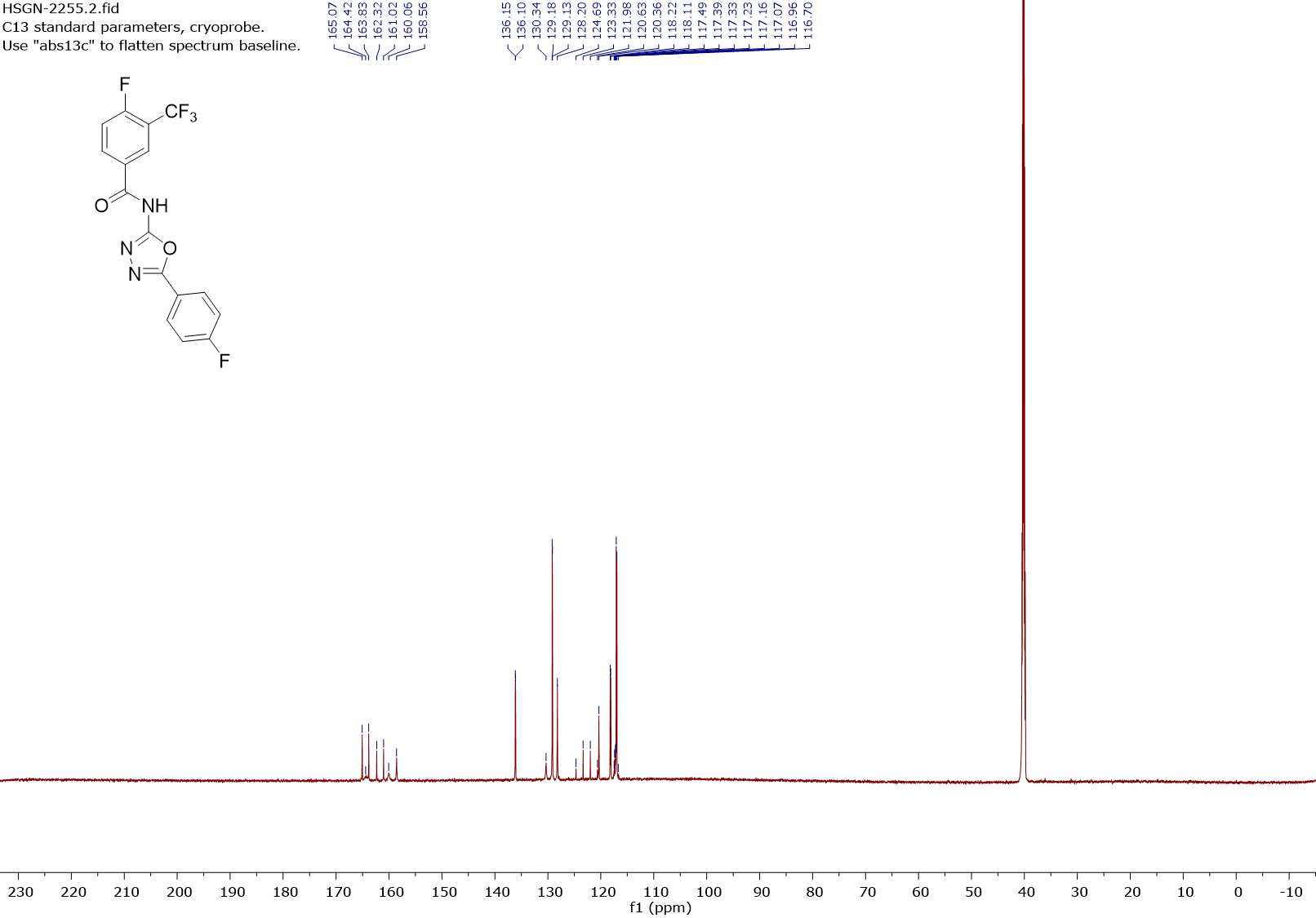


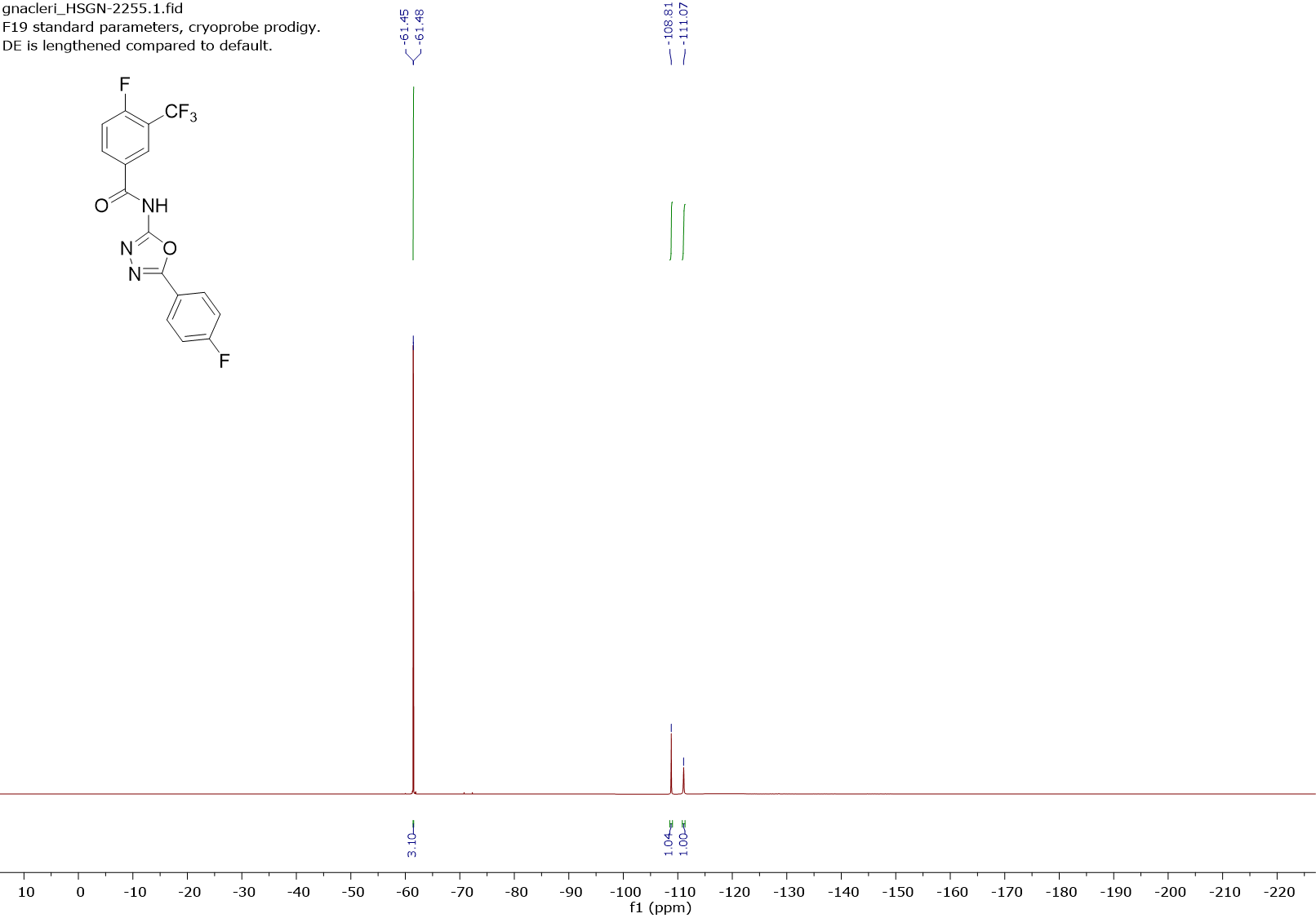


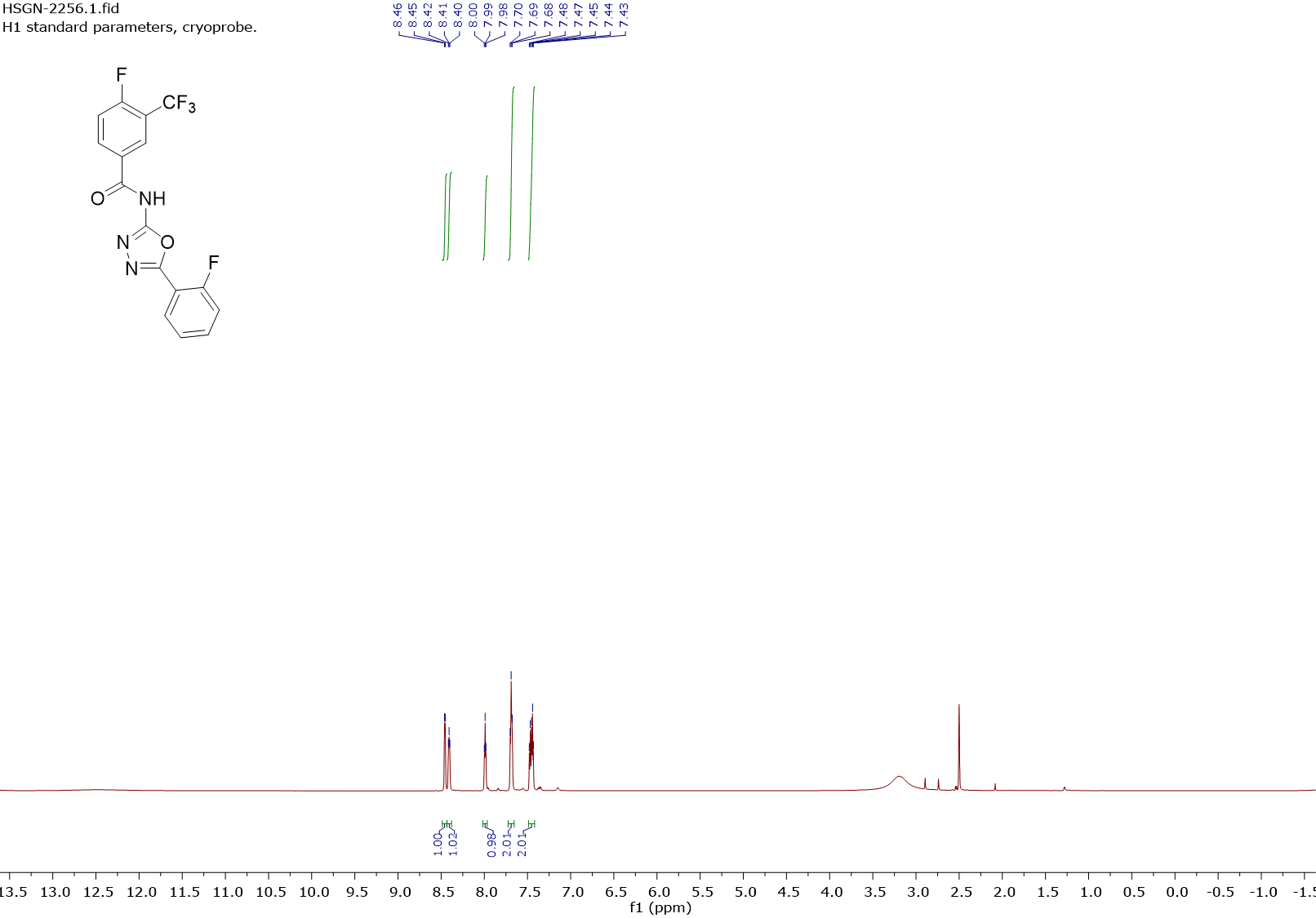


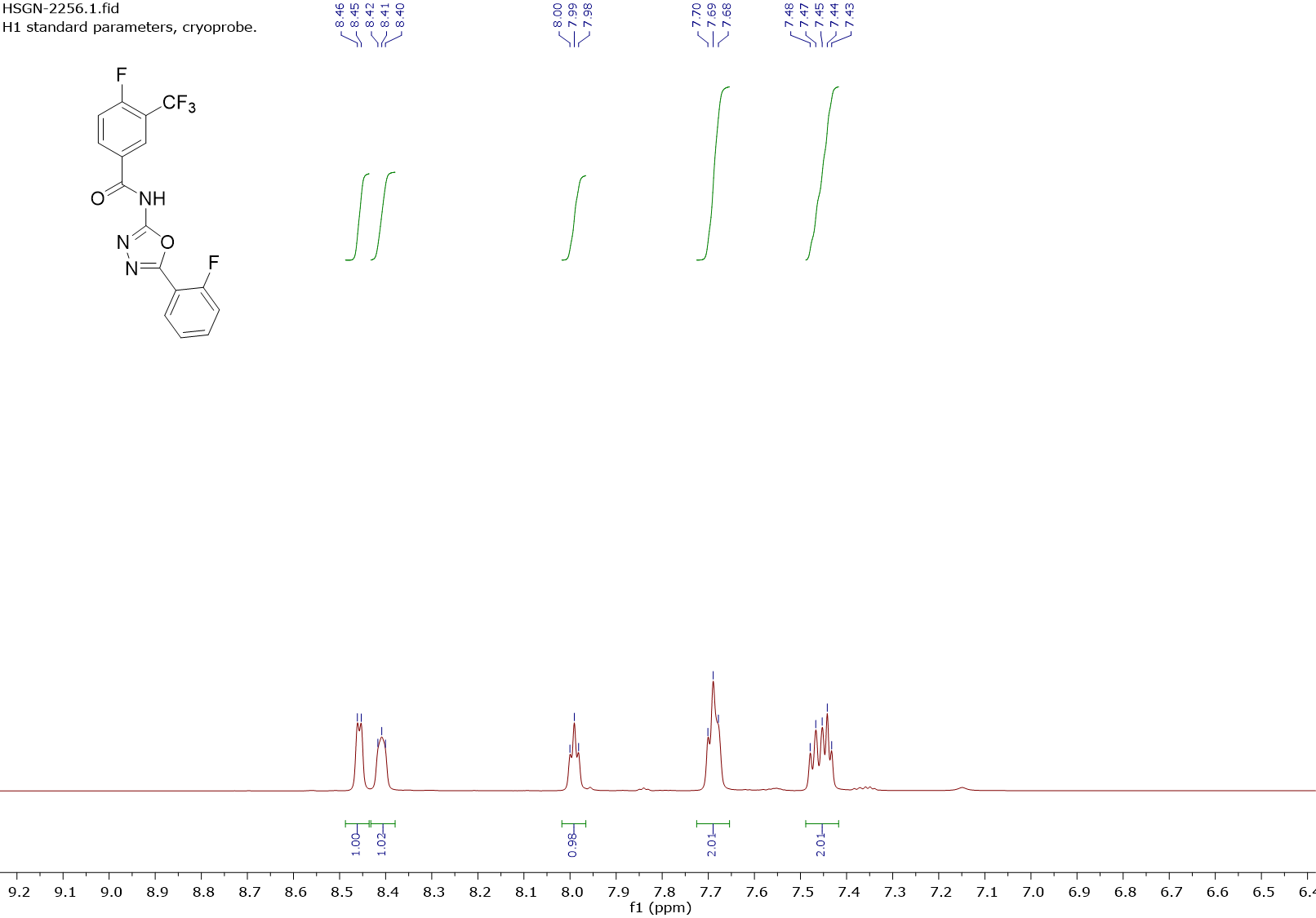


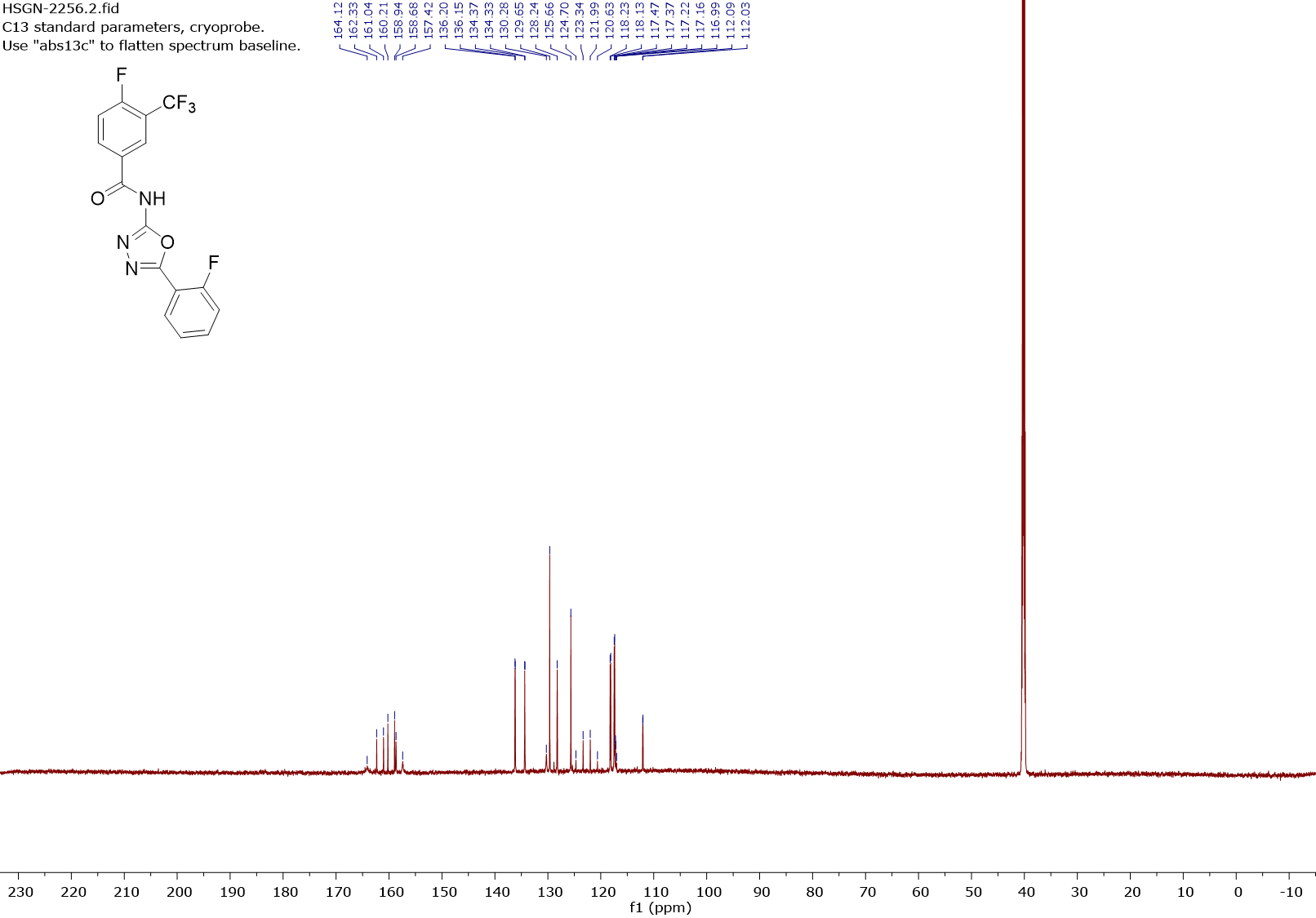


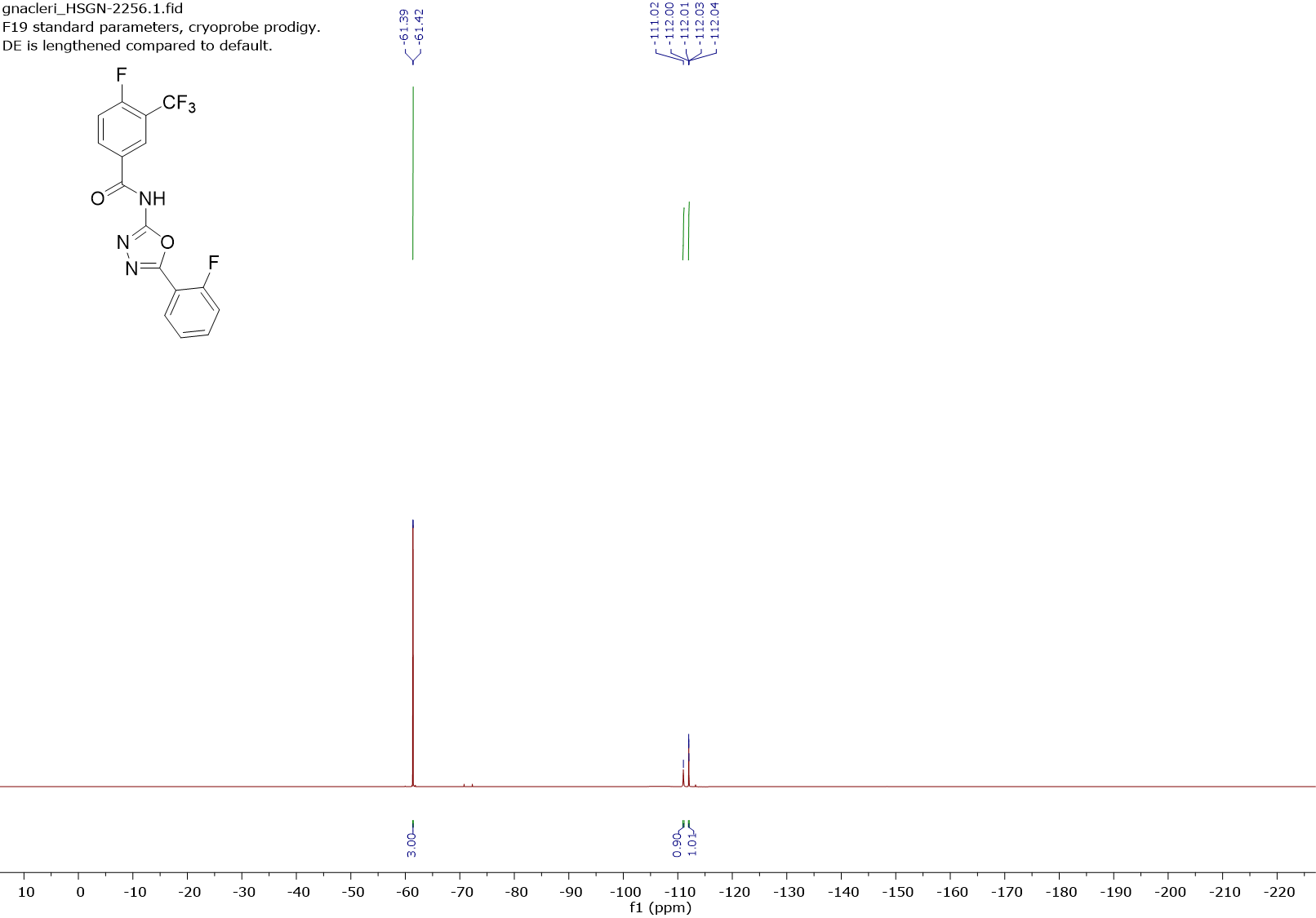


**References:**

[1] B. Kot, H. Sytykiewicz, I. Sprawka, M. Witeska, Effect of manuka honey on biofilm-associated genes expression during methicillin-resistant Staphylococcus aureus biofilm formation, Sci Rep, 10 (2020) 13552.
